# Will Trespassers Be Prosecuted or Assessed According to Their Merits? A Consilient Interpretation of Territoriality in a Group-Living Carnivore, the European Badger (*Meles meles)*

**DOI:** 10.1371/journal.pone.0132432

**Published:** 2015-07-06

**Authors:** Helga V. Tinnesand, Christina D. Buesching, Michael J. Noonan, Chris Newman, Andreas Zedrosser, Frank Rosell, David W. Macdonald

**Affiliations:** 1 Faculty of Arts and Sciences, Department of Environmental and Health Studies, Telemark University College, N-3800 Bø i Telemark, Norway; 2 Wildlife Conservation Research Unit, Dept. of Zoology, University of Oxford, The Recanati-Kaplan Centre, Tubney House, Abingdon Rd, Tubney, Abingdon, OX13 5QL, United Kingdom; 3 Department of Integrative Biology, Institute of Wildlife Biology and Game Management, University of Natural Resources and Life Sciences, Vienna, Gregor Mendel Str. 33, A-1180 Vienna, Austria; University of Veterinary Medicine Hannover, GERMANY

## Abstract

Socio-spatial interactions of Carnivores have traditionally been described using the vocabulary of territoriality and aggression, with scent marks interpreted as ‘scent fences’. Here, we investigate the role of olfactory signals in assumed territorial marking of group-living solitary foragers using European badgers *Meles meles* as a model. We presented anal gland secretions (*n* = 351) from known individuals to identifiable recipients (*n* = 187), to assess response-variation according to familiarity (own-group, neighbours, strangers) and spatial context (in-context: at a shared border; out-of-context: at an unshared border/ the main sett). Sniffing and over-marking (with subcaudal gland secretion) responses were strongest to anal gland secretions from strangers, intermediate to neighbouring-group and weakest to own-group members. Secretions from both, strangers and neighbours, were sniffed for longer than were own-group samples, although neighbour-secretion presented out-of-context evoked no greater interest than in-context. On an individual level, responses were further moderated by the relevance of individual-specific donor information encoded in the secretion, as it related to the physiological state of the responder. There was a trend bordering on significance for males to sniff for longer than did females, but without sex-related differences in the frequency of subcaudal over-marking responses, and males over-marked oestrous female secretions more than non-oestrous females. There were no age-class related differences in sniff-duration or in over-marking. Evaluating these results in the context of the Familiarity hypothesis, the Threat-level hypothesis, and the Individual advertisement hypothesis evidences that interpretations of territorial scent-marks depicting rigid and potentially agonistic discrimination between own- and foreign-group conspecifics are overly simplistic. We use our findings to advance conceptual understanding of badger socio-spatial ecology, and the general context of territoriality and group-range dynamics.

## Introduction

The socio-spatial interactions of Carnivores have conventionally been described using the vocabulary of friend or foe, manifesting through territoriality and aggression [1]. This resulted in the strict differentiation between two types of habitat use: While the term ‘home range’ refers to the area, which an animal or social group uses for its day-to-day activities, ‘territory’ refers to the area within the animal’s or group’s home range over which it has exclusive (or at least priority) use; a territory is, by definition, defended and can encompass the home range in part or completely [2]. Such socio-spatial relationships are most complex in group-living species, where individuals permit certain familiar conspecifics to co-share their territory while excluding others [3]. This involves a trade-off between costs of defence versus resource security benefits [4]. Yet, the mechanisms involved, and the role of heterogeneities in individual costs and benefits linked to participation in group defence, are still not fully understood [5]. Crucially, residents must be able to recognise, remember and discriminate other own-group members from extra-group individuals (i.e., neighbours and strangers: [6, 7]). Ability to determine individual-specific characteristics, group membership and familiarity then aids in the formation of more complex animal societies [8].

Olfactory signals are particularly effective in this context due to their latency (i.e., their persistence in the environment over time), where the signalling individual does not need to be present to convey information [9]), nor do they need to encounter conspecifics directly to gain information [10, 11]. Consequently, olfactory cues such as faeces and urine play a critical role in defining the activity range of an individual (e.g. [12] or group (e.g. [13]). Scent-matching of scent-marks to resident individuals therefore allows intruders to identify territory holders, alleviating potentially costly aggressive conflicts (e.g. [14, 15]). Scent marks are thus often referred to as a form of territorial defense and conventionally interpreted as ‘keep-out signs’ [16] or ‘scent fences’ (e.g. [17])

Game theory [18] predicts that the outcome of territorial disputes hinges on two factors: (i) the value that the defended resource has for each individual’s fitness [14], and (ii) the contestant’s fighting ability (resource holding power: [19, 20]. Accordingly, territorial animals are expected to react in a context-specific manner. The Dear Enemy Phenomenon (DEP: [6] predicts that, by evaluating the relative threat posed by interlopers, residents can direct attention towards individuals that pose the greatest risk, while being more tolerant of others (Threat-level hypothesis: [21]). Thus, when competition with neighbours is intense, residents are predicted to respond more strongly to their neighbours, or to their olfactory cues, than to strangers [8]. In situations where strangers pose the greater threat than neighbours (e.g., dispersers intent upon territory acquisition), residents will respond more strongly to them. The resulting geometry of individual- or group-territories manifests as a contiguous socio-spatial network, maintained primarily by scent-marking rather than direct aggression [14].

This conceptual framework can, however, lead to interpretive bias because it implies that all same-group conspecifics (*own-group*: *OG*) are ‘friends’, that is, conspecifics against which no aggression will be shown; whereas all individuals belonging to other groups (*extra-group*: *EG*) are ‘enemies’, potentially subject to aggressive exclusion/ repulsion [11]. This discounts social nuances, where OG-individuals may also be threatening (e.g. dominance; reproductive suppression, food competition) and EG may have positive intent (e.g. seeking mating opportunities), or benign consequences under different resource scenarios (e.g. [22]).

Of particular interest is range use and territoriality in group-living solitary foragers—typically a spatial aggregation of individuals best explained by the particular dispersion of their key resources, as described by the Resource Dispersion Hypothesis (RDH: reviewed in [23]). This social system is more common than previously understood, often involving a common denning site. Functionally, there is little basis for these spatial groups to operate in isolation. The formation of obligate social groups is determined by bottom-up drivers (sensu [24]), such as collaborative and cooperative social behaviours between group members. In RDH-based spatial-groups, these social behaviours are not predicted to be intrinsic to group formation, rather the tolerance of a limited number of conspecifics (often kin) is less costly than trying to maintain an exclusive range under key resource conditions (see [23]). Consequently the population will undergo top-down segregation into social-group units, determined by the most economic division of resources that allows necessary resource security [23, 24].

An example of an RDH-based facultative group-living, solitary forager is the European badger *Meles meles* [23], for which a consilient understanding of socio-spatial segregation and territoriality has proven challenging, as new data contradict traditional views of territorial exclusivity in this species [25]. Understanding the details of badger sociality, however, is particularly important as, in the UK, this species is implicated as a wildlife reservoir in bovine tuberculosis (bTB) breakdowns in cattle herds (reviewed in [26]). The government-implemented large-scale culling operations of badgers as bTB management strategies rely largely on the notion of territoriality by specifically targeting infected social groups [26].

Badgers rely primarily upon olfaction to gain information about their environment and conspecifics [27], and–like many carnivores–establish composite latrines containing faeces, urine, subcaudal and anal gland secretions, the location of which can remain stable over decades [28]. Bait-marking surveys [29] separate these latrines into two types: ‘hinterland latrines’, which are used exclusively by members of the resident social group, and ‘border latrines’, which are shared between neighbouring groups [30]. Since Kruuk’s formative descriptions of badger society [31], these border latrines are conventionally assumed to link up into defended perimeters, which divide group-territories via ‘scent fences’ [32]. This, however, is interpretative and a potential source of bias in the role of latrines: Simply because the bait-marking procedure [30] draws hard lines linking latrines to segregate groups, does not mean that badgers perceive the resultant ‘borders’ in such a rigid way. In actuality, there is scant evidence of active territorial defence in badgers [33], with little aggression between neighbours observed at border latrines [34]. Consequently, although the conventional ‘active territorial defence’ hypothesis [33], would interpret latrine use in badgers at territory borders as a stand-off in terms of each group's resource holding potential, signalling encounter likelihood across the boundary [32], explanations based on non-agonistic but resource-pragmatic mechanisms of range separation between groups, such as the Passive Range Exclusion Hypothesis [33] appear increasingly plausible.

New data show that badgers from different social groups meet at latrines frequently, and pass by each other amicably, proceeding into their neighbour's range [34]. Trapping records [35] and underground telemetry [36] evidence that individual badgers can reside within the main den (‘sett’) of other (primarily neighbouring) social groups for several days, with some individuals disappearing from their home sett for weeks, presumably residing with more distant groups [36]. In Ireland, Byrne *et al*. [37] observed badgers making long-distance movements, crossing the implied territories of others and likely staying temporarily in setts occupied by less familiar conspecifics. Genetic pedigree has revealed that badgers breed frequently between groups [38–40], undermining territory defence by the inference that it is ineffective. Truly co-operative behaviours are few [41–43], and hence badger groups cannot be defined as ‘eusocial’, ‘breeding’ or ‘spatial’ [25].

In a previous study, Palphramand & White [44] demonstrated that badgers are able to discriminate between faeces from OG, neighbours and strangers, but interpreted this only in the context of territoriality and DEP. The interpretative power of their study, however, was limited because they were not able to identify the sex, age or group affiliation of either the donor or recipient animal. Here, we used anal gland secretion (AGS, which coats faecal deposits: [28]) isolate to investigate the broader context of scent function in badger society.

Mustelid AGS encodes idiographic as well as nomothetic [45] parameters (e.g., group-membership and individual-specific information such as age, sex, reproductive status), independent of faecal odour cues associated with foraging success. AGS from known individuals was presented to identifiable wild recipients to assess responses to familiar and unfamiliar scent-marks, and whether these vary according to context expectation in different regions of badger group-ranges. This framework permitted to test the predictions of three different (not necessarily mutually exclusive) hypotheses:

- The **familiarity hypothesis** [6, 21], which posits that residents will react to the degree of familiarity of scent cues, unaffected by any individual-specific information encoded. Thus, corresponding with increasing familiarity, badgers would show a lesser response to OG-AGS than to neighbours or strangers.
- The **threat-level hypothesis** [21], which posits that residents will modify their response according to the perceived level of threat, implied by the spatial-context of the cue [46]. Thus, AGS from non-residents at the resident’s sett, rather than at the territory perimeter, and AGS from neighbours at unshared, as opposed to shared borders, would elicit a greater response, as these scenarios might signify territory incursion.
- The **individual advertisement hypothesis** [27], which posits that scent-marks advertise individual-specific characteristics, such as sex, age and/or reproductive status. Thus, the response of residents would be predicated upon the relevance of the encoded information, relative to their own physiological status and motivation, and largely independent of spatial context.

The findings of this study are used to advance conceptual understanding of badger socio-spatial ecology, and are placed into the general context of territoriality.

## Materials and Methods

### Study population

This study was carried out in a high-density badger population at Wytham Woods, Oxfordshire, England (51:46:26N; 1:19:19W; a designated Site of Special Scientific Interest (SSSI), owned by the University of Oxford) between 30^th^ May – 8^th^June 2012 and 3^rd^ – 15^th^ June 2013, when latrines are particularly active [29]. Since 1987, a long-term trapping programme has documented the life histories and social-group affiliations of all individuals in this population from birth until death (usually indicated by disappearance from trapping records) over seasonal trapping sessions each year [47]. The population comprised 23 social groups with ca. 182 adults and 39 cubs in 2012, and ca. 215 adults and 40 cubs in 2013 (estimated after [47]), permitting good statistical power. Social group ranges, and thus the affiliation of badgers caught at setts in each group range, were inferred from bait-marking and latrine surveys [29].

### Trapping protocol

All field work and data collection for this study was covered by our Natural England license 2014-5710-SCI-SCI and Home Office license PPL 30/2385; and all work was approved and supervised by the University of Oxford’s Zoology Ethical Review Committee. Badgers (protected in the UK under the Badger Act 1992) were caught and sedated for handling and sampling, following the methodology detailed in Macdonald *et al*. [47] as part of an ongoing population study. All trapped badgers were identifiable from a permanent individual tattoo number on the left inguinal region. In addition, all juveniles (<2 years, sexually immature) and adults (≥2 years) were fur-clipped at every trapping [48], permitting visual identification of individuals for behavioural analyses to cross-reference against their life-history records in the trapping record. Cubs (<1 year) were not clip-marked and were excluded from all analyses.

Sex, age-class (juvenile and adult), and reproductive status were recorded for each capture as ‘individual characteristics’. For adult females, oestrus was inferred from vulva condition and classified as oestrous (vulva swollen and moist), or non-oestrous (vulva flat and dry). Although all males had scrotal testes and were assumed to be in breeding condition during the field trials [49], the degree of testes descent varied between descended (testes descended into the scrotum, but lateral movement restricted) and fully descended (testes fully scrotal and mobile).

The social group affiliation of the individual (i.e. its capture site) defined its ‘status’, i.e., its group affiliation relative to the responding individual.

### Sample collection

AGS samples (*n* = 50; 22 adult males; 6 yearling males; 18 adult females; and 4 yearling females) were collected in sterile 2.0ml glass vials with Teflon lined caps by gently everting and palpating the left anal gland papilla while the badger was sedated. Secretion was stored at –20°C until used in the trials. Only samples obtained within 10 days of the scent presentation experiment were used to maintain contemporaneous context.

### Scent-presentation experiments

In total, we conducted 117 trials in 11 different social groups (for details of donors see Table 1). In each trial, a sample vial, containing approximately 0.1g of AGS, was pushed into the ground until the rim was level with the surrounding soil, and an empty control vial was placed 50cm (i.e., approximately one badger head-body length) from the sample. Vials were deployed between 18:00 and 19:00, approximately 1 hour before badger emergence. New latex gloves were worn whenever samples were handled to avoid scent-contamination. To identify sample and control vial location on the camera footage, the researcher took a photo of themselves with the in situ camera immediately after vial deployment, pointing to both vials.

**Table 1:** Scent samples used in the behavioural trials varying in levels of familiarity (own- group, neighbour, stranger), spatial-context (sett, shared border, unshared border) and sex and age class of the scent donor (n = 117).

Treatment	Location	N_Total_	N_Female Adult_	N_Female Yearling_	N_Male Adult_	N_Male Yearling_
Own-group	Sett	13	4	1	7	1
	Border	17	6	1	8	2
Neighbour	Sett	17	5	1	8	3
	Shared border	15	5	1	8	1
	Unshared border	13	5	1	6	1
Stranger	Sett	22	6	2	12	2
	Border	20	6	1	11	2

#### Familiarity treatments

AGS from three different donor categories were presented:

- OG (i.e., donor resident in the same social group where its AGS was presented; *n* = 30)
- Neighbour (i.e., donor resident in a neighbouring group to the group where its AGS was presented; *n* = 45)
- Stranger (i.e., donor resident in a social group at least 2 territories apart; *n* = 42).

AGS was presented either at the main sett or at a border latrine:

- OG presented at main sett (*n*_Own_ = 13) or at border latrine (*n*_Own_ = 17).
- Neighbour presented at main sett (*n*_Neighbour_ = 17), at a shared border latrine (*n*_Neighbour-shared_ = 15) or at a latrine on a different sector of the responder’s group’s border (i.e., not shared with that particular neighbour: *n*_Neighbour-unshared_ = 13).
- Stranger presented at main sett (*n*_Stranger_ = 22) or at border latrine (i.e., always unshared: *n*_Stranger_ = 20).

AGS from each scent donor was used only once in each category to avoid habituation. Trials were conducted at each social group for three days, with one AGS sample presented per night at each location. Treatment order was randomised.

### Behavioural Observations

Responses were recorded using infra-red ReconyxHyperFireHC600 cameras (Reconyx Inc., Wisconsin, USA; one camera per site) taking two frames per second. At each site, one camera was set up in a nearby tree, approximately 1.5m off the ground and–depending on tree-availability—between 1.5m and 3.5m distance from the sample, a minimum of 12 hours before the start of each 3-day-trial-period, and checked every morning. If badgers investigated a sample during the previous night, the respective sample and control vial were removed and a new sample and control vial presented at this location in a different spot (ca. 50cm away from previous sample) the following evening. If no badger responded, the sample and control were removed in the morning and replaced with a new control vial and a fresh sample from the same donor the following evening to maintain social-contact continuity.

#### Response measurements

A **potential response** was defined as a badger (‘responder’) approaching the sample to ≤50cm (ca. one head-body length), from where the badger would be capable of detecting the scent.

As a subset of these potential response events, an **actual response** was defined as progression to a badger positioning its nose within ≤10cm of the sample (‘sniffing’ the sample; the exact extent to which badgers may have licked samples was hard to discern consistently and was not used as a variable in the analyses, but tongue-contact with the sample was observed in some instances, indicating use of the vomero-nasal organ: [50]).

For each response, (i) the number of times an individual sniffed the sample (potential response = 0 sniffing; actual response ≥ 1 sniffing) was recorded; (ii) the sniff-duration in seconds; (iii) the number of times an individual responded with subcaudal-marking within a 50cm radius of the sample (noting that badgers cannot scent-mark with AGS unless they defecate); and (iv) whether this subcaudal marking involved **over-marking** on top of the sample, or **proximity-marking** within 50cm of the sample [51] were recorded for each actual response.

### Statistical analyses

To test the predictions of the familiarity-, threat-level-, and individual advertisement hypotheses, the entire dataset (all responses regardless of identifiability of responder; *n* = 351; for details of individuals participating in the experiments see S5 Table) was first evaluated with analyses of variance (ANOVA).

The relative importance of individual-specific characteristics of the scent donor (as established during the recent trapping session when AGS was collected) on sniff-duration and over-marking responses was then analysed using an information-theoretic (IT) approach employing Akaike information criteria on the complete dataset (*n* = 351; see [52]).

To further understanding of potential individual advertisement information encoded in AGS, IT-modelling was then expanded to investigate the influence of individual responder characteristics (age-class, sex, relative group-affiliation) as well as their interaction with scent-donor characteristics on sniff-duration and over-marking responses (restricting the dataset to those observations where the responder could be identified individually; *n* = 187; for details see S5 Table).

To satisfy assumptions of normality, data on sniff-duration were log–transformed in all analyses.

#### i) Effects of scent familiarity on interest levels of the responder: The Familiarity hypothesis

To evaluate how residents responded to treatments at group-level, cumulative group-level responses (i.e. the mean of responses of individuals affiliated to that group) were analysed by testing the mean duration of sniffing events across all potential and actual responses, as well as the mean number of scent-marking events across all actual responses, against the ‘category of the donor’ (levels: own-group, neighbour, stranger).

Responses to AGS from neighbours at unshared borders were excluded from these analyses, as they are implicitly always ‘out-of-context’. Following the approach of Palphramand and White [44], social group, rather than individual, was treated as a random, repeated effect in these analyses.

#### ii) Effect of socio-spatial context on interest levels of the responder: The Threat-level hypothesis

To test the threat-level hypothesis, familiarity was controlled for by presenting AGS from neighbours in three different socio-spatial-contexts (levels: shared border, sett, unshared border). The mean duration of sniffing events and the mean number of scent-marking events was then tested against these socio-spatial-contexts levels. Responses to ‘own-group’ (implicitly always ‘in-context’) and ‘strangers’ (implicitly always ‘out-of-context’) were excluded. As before, social group was treated as a random, repeated effect.

#### iii) Effects of relevance of individual-specific information encoded in AGS on the interest-levels of the responder: Individual advertisement hypothesis

Akaike information criterion (AIC) model selection was applied to reference effects of individual-specific fitness-related information encoded in the AGS, against the characteristics of the responder [53, 54]. These analyses used sniff-duration per potential response, as well as number of scent-marking events per actual response, as treatment response variables. Model selection does not support missing data, because this invalidates between-model contrasts. As individuals could not be identified from their clip-marks in 46% of observations due to the camera-angle, model selection was performed using the complete dataset initially, including unidentifiable responders. This global model included the variables ‘age’ of the donor (levels: yearling, adult); ‘sex’ of the donor (levels: male, female); ‘reproductive status of the female donor’ (levels: oestrous, non-oestrous); and ‘reproductive status of the male donor’ (levels: descended, fully descended), as well as interaction terms. Trial ID was included as a random effect in these models.

Subsequently, and restricting the dataset to those observations where the responder clip-marks could be identified unambiguously (*n* = 187; for details see S5 Table), individual responses were evaluated, based on a global model that included the same variables as above, but also including the characteristics of the responder. Responder and trial ID were included as random effects in these models [55].

The AIC was derived to rank the support for each model, and the delta AIC (∆_i_) calculated in relation to the highest-ranking model and Akaike weight (*w*) [54]. Model averaging was applied to derive estimates of the coefficients (*θ*) associated with all sets of models, and their 95% confidence intervals (CI). To take into account uncertainty in model selection [53], mean coefficient values were calculated by averaging their values over all models that included the coefficient of interest, weighted by *w*. The ‘Relative Influence’ of each predictive variable was calculated as the summation of *w* across all models that included the variable of interest [53].

All statistical analyses were performed in R-v.3.0.3 (R Core Team 2013) using the ‘lme4’, ‘nlme’, and ‘MuMIn’ packages. Although statistical analyses were performed on transformed data, for visual purposes, figures are based on un-transformed data. All data are included as S5 Table.

## Results

From a total of 117 trials, 85 (*n*_Own_ = 20, *n*_Neighbour_ = 35, *n*_Stranger_ = 30) produced 351 potential responses (*n*_Own_ = 112; *n*_Neighbour_ = 125, *n*_Stranger_ = 126), including 196 actual responses involving sniffing (*n*_Own_ = 27, *n*_Neighbour_ = 81, *n*_Stranger_ = 88) and 141 involving subcaudal-scent-marking responses (*n*_Own_ = 9, *n*_Neighbour_ = 49, *n*_Stranger_ = 83). Sniff-duration could not be determined accurately for 13 responses. There was no potential response in 32 trials, which were excluded from all analyses. Badgers never responded to blank control vials.

When badgers sniffed AGS, their noses generally made direct contact with the sample (*n* = 188/196). AGS from strangers was dug out more often (26.7%, *n* = 8/30), compared to neighbours at unshared borders (15.4%, *n* = 2/13) and at setts (7.6%, *n* = 1/13), whereas OG- and neighbour-AGS at shared borders was never interfered with; there were, however, insufficient data to analyse these digging responses systematically.

### i) Effects of scent familiarity on interest levels of the responder: The Familiarity hypothesis

**Fig 1:**
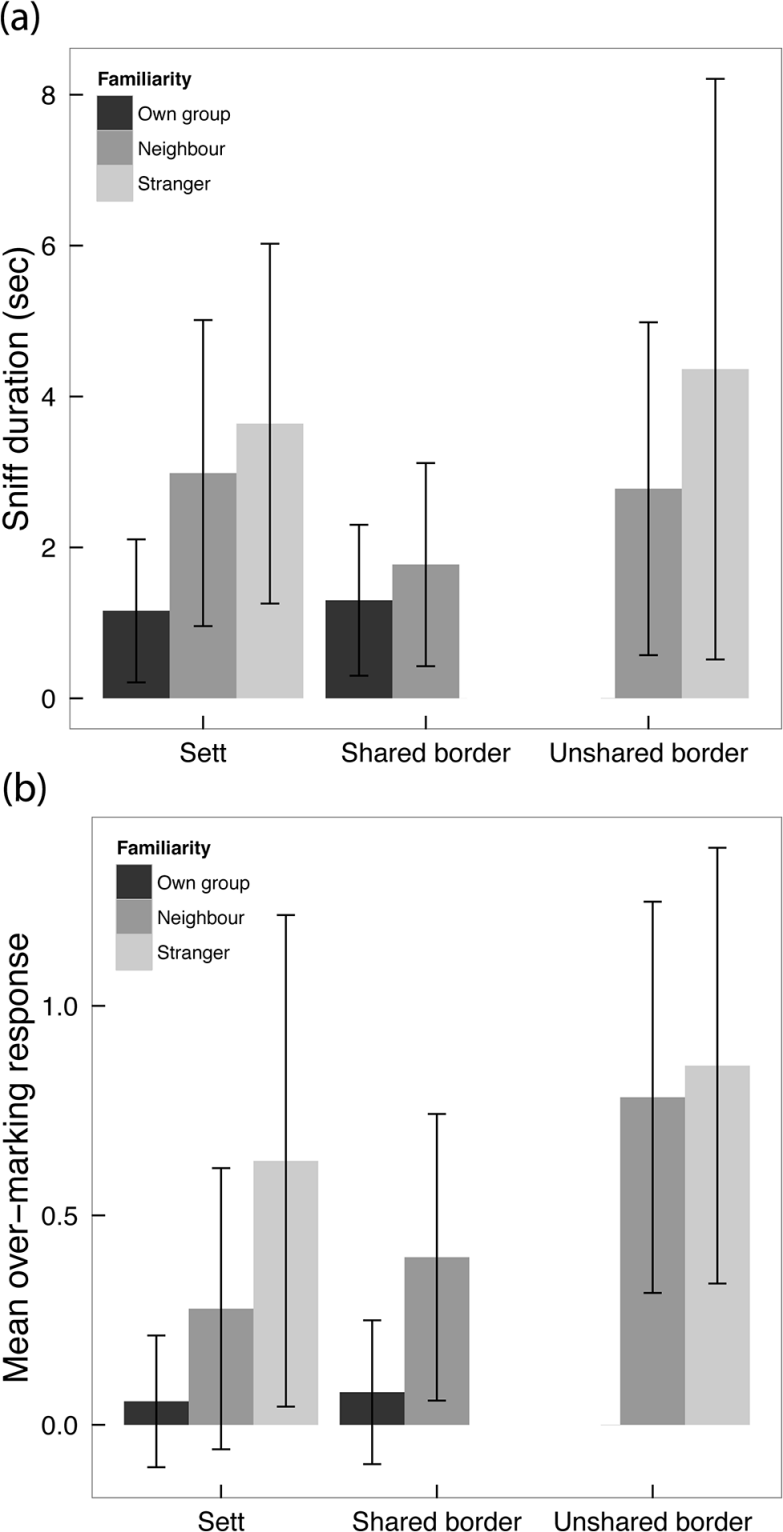
Group-level variation in responses to AGS from donors of different levels of familiarity. Mean (±SD) group level (a) duration of sniffing responses in seconds; and (b) number of subcaudal-marking responses per potential response to AGS from own–group (*n* = 20 trials), neighbours (*n* = 35) and strangers (*n* = 30) presented at either the responding group’s sett, or a border (shared or unshared) between the responding group and the scent donor.

Cumulative group-responses to introduced scent were stronger when AGS was less familiar (weakest to OG-AGS, with intermediate responses to neighbouring-group samples, and strongest to stranger-AGS). The duration of sniffing responses at the group-level differed significantly between all levels of familiarity (ANOVA: *F*_2,67_ = 14.17, *P*< 0.0001; Tukey *post hoc* test: all *P*-values < 0.027; Fig 1A), and were longer in response to AGS from both strangers ($\bar{x}$ = 4.29s ± 2.60 SD, *n* = 32), and neighbours ($\bar{x}$ = 2.71s ± 2.56 SD, *n* = 33), than to OG ($\bar{x}$ = 1.44s ± 2.58 SD, *n* = 19). Similarly, over-marking responses differed significantly between all levels of familiarity (ANOVA: *F*_2,67_ = 15.45, *P* < 0.0001; Tukey *post hoc* test: all *P*-values < 0.03; Fig 1B), and were more frequent to AGS from both strangers ($\bar{x}$ = 0.77 ± 0.51 SD, *n* = 32), and neighbours ($\bar{x}$ = 0.48 ± 0.50 SD, *n* = 33), compared to OG-AGS ($\bar{x}$ = 0.07 ± 0.50 SD, *n* = 19).

### ii) Effect of socio-spatial context on interest levels of the responder: The Threat Level hypothesis

Across the complete dataset including all levels of familiarity, sniff-duration differed significantly between locations (ANOVA: *F*_2,67_ = 4.73, *P* = 0.012). Neighbour-AGS provided out-of-context, at either the sett or at an unshared border, however, received no greater interest from the responder (ANOVA: *F*_1,18_ = 2.88, *P* = 0.11) than at an in-context location (shared border).

This pattern was also apparent in subcaudal-over-marking responses that, across all levels of familiarity, were affected significantly by location (*F*_2,67_ = 12.08, *P* < 0.0001); although neighbour-AGS presented out-of-context was not over-marked more often than at an expected location (ANOVA: *F*_2,18_ = 0.73, *P* = 0.41).

### iii) Effects of relevance of encoded individual-specific information content on the interest levels of the responder: Individual advertisement hypothesis

**Fig 2:**
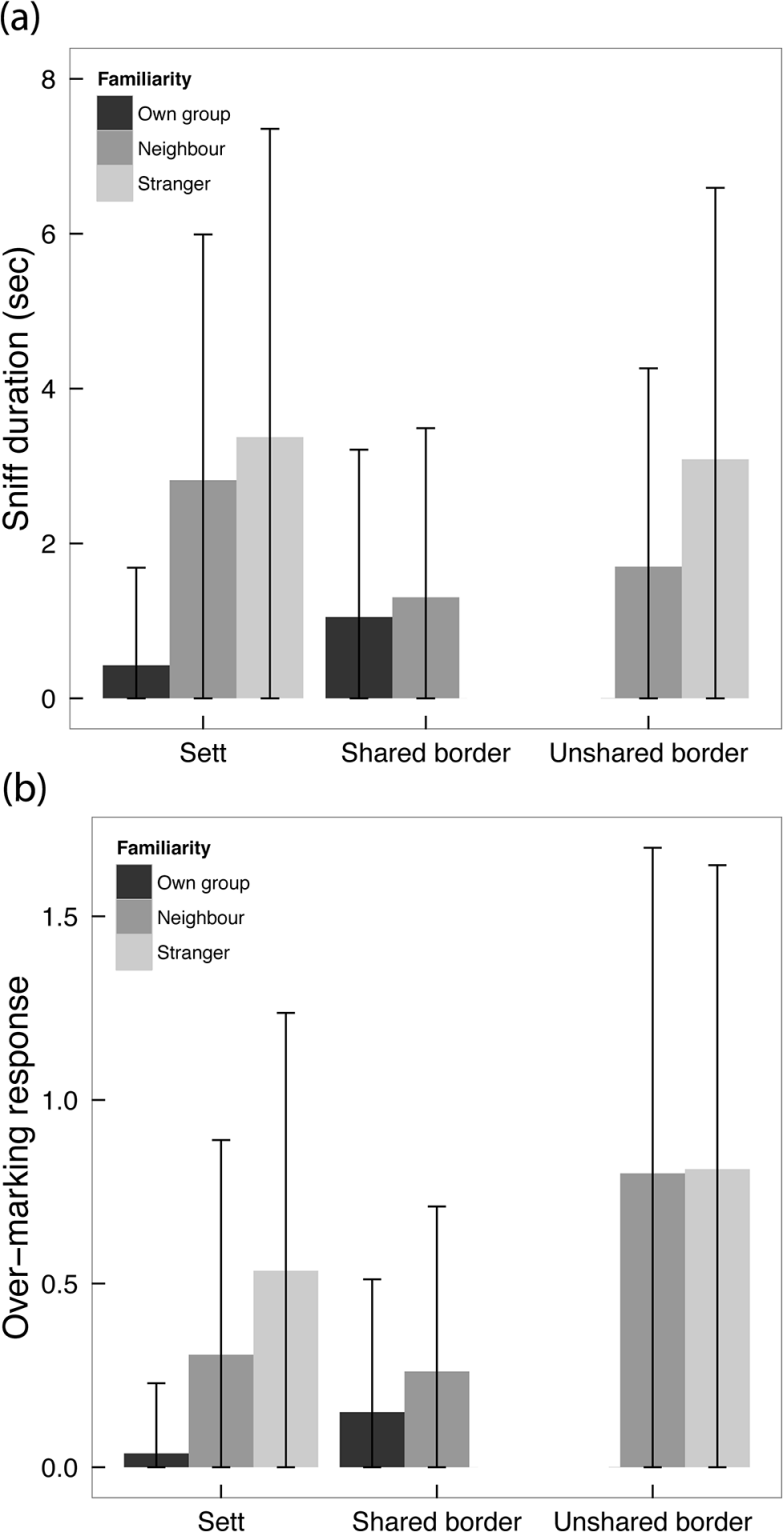
Individual-level variation in responses to AGS from donors of different familiarity levels. Individual level (±SD) (a) duration of sniffing responses; and (b) number of subcaudal-marking responses per potential response to AGS from own–group (*n* = 20 trials), neighbours (*n* = 35) and strangers (*n* = 30) presented at either the responder’s sett, or a border (shared or unshared) between the responder and the scent donor.

In general, individual-level responses followed the same overarching patterns as those at the group-level: sniff-duration differed significantly with familiarity (two-way ANOVA: *F*_2,330_ = 37.70, *P* < 0.0001), where the duration of responses decreased with greater familiarity (Fig 2A) with no significant effect of AGS-location on sniff-duration (*F*_3,330_ = 1.96, *P* = 0.12). Conversely, subcaudal-marking responses differed significantly between levels of familiarity (two-way ANOVA: *F*_2,343_ = 19.76, *P* < 0.0001), as well as location (*F*_3,343_ = 3.05, *P* = 0.029; Fig 2B).

Responding individuals could be identified in 54% of observations (*n* = 187; Total number of identifiable individuals = 45 of which *n*_Male Adult_ = 14, *n*_Male Yearling_ = 3, *n*_Female Adult_ = 25, *n*_Female Yearling_ = 3). In 12.9% of trials (*n* = 11) an identifiable individual returned a second, and in one case also a third time, to respond to the same AGS sample. In 63.6% (*n* = 7) of these instances, the donor was a stranger (*n*_Male_ = 4, *n*_Female_ = 3), a neighbour at an unshared location in 27.3% (*n* = 3, *n*_Male_ = 1, *n*_Female_ = 2), while in only one case the scent donor was a young female from the responding female’s own group.

When examined in more detail, individual responses were moderated by the relevance of individual-specific information encoded in the donor AGS to the physiological state of the responder.

**Fig 3:**
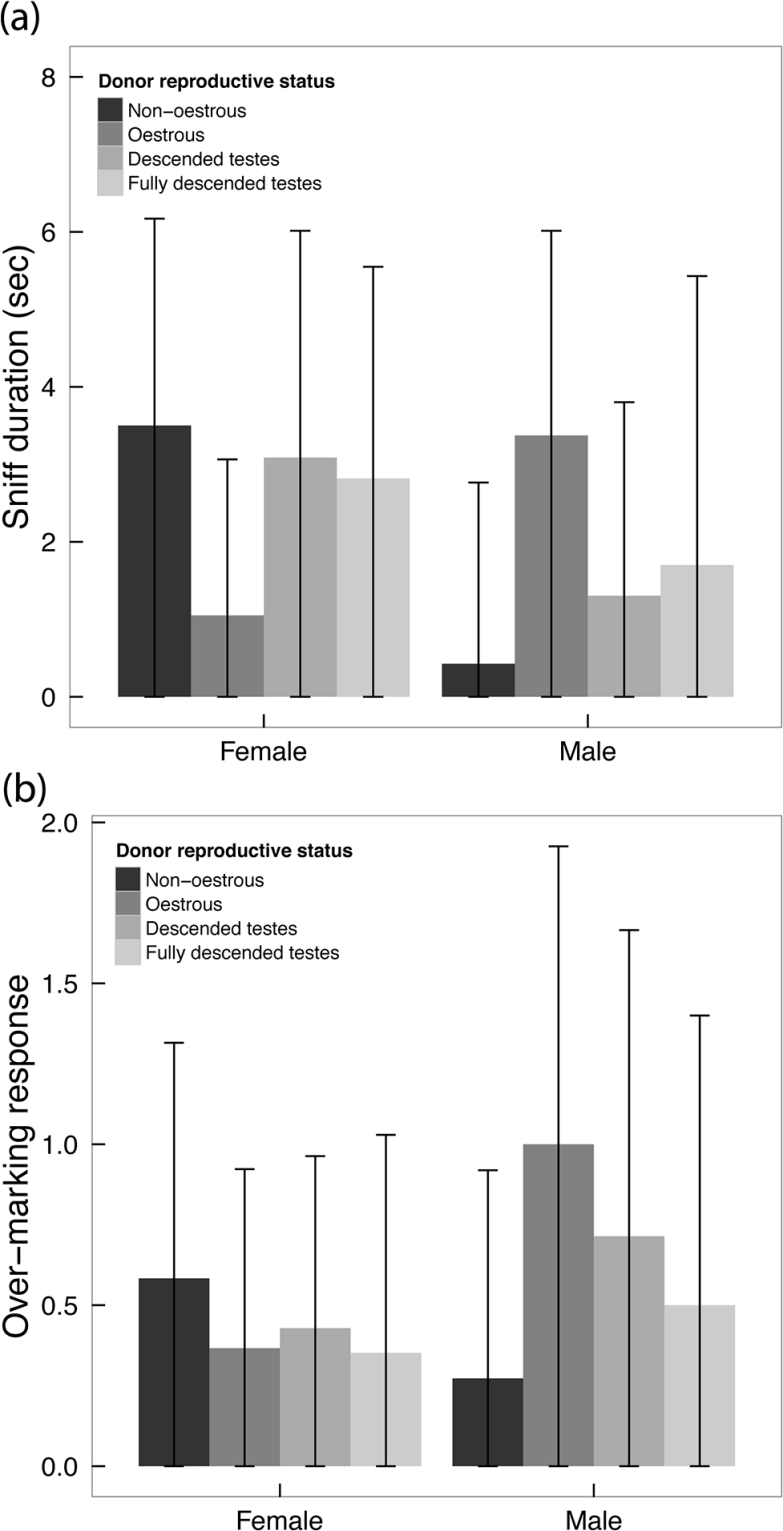
Variation in responses to AGS according to the reproductive status of the donor. Individual male and female (±SD) (a) duration of sniffing responses; and (b) number of subcaudal-marking responses per potential response to AGS in relation to the reproductive status of female (levels: oestrous, non-oestrous) and male (levels: descended testes, fully descended testes) scent.

Overall, males tended to sniff for longer than did females ($\bar{x}$ = 2.38s ± 3.08 SD; $\bar{x}$ = 1.73s ± 3.12 SD respectively), bordering significance (ANOVA: *F*_1,79_ = 3.83, *P* = 0.054), but there were no sex-related differences in the frequency of over-marking responses (ANOVA: *F*_1,80_ = 1.30, *P* = 0.26; Fig 3A). Similarly, there were no age-class related differences in sniff-duration or over-marking responses (ANOVA: *F*_1,72_ = 1.31, *P* = 0.26; *F*_1,72_ = 0.13, *P* = 0.72 respectively).

Interestingly, animals in reproductive condition sniffed AGS significantly longer and subcaudal over-marked it more often, although the latter effect was not significant (ANOVA: *F*_1,59_ = 3.99, *P* = 0.012; *F*_1,59_ = 2.52, *P* = 0.066 respectively). Whereas males tended to over-mark oestrous female AGS more than non-oestrous female AGS (Fig 3B; bordering significance: ANOVA: *F*_1,17_ = 4.09, *P* = 0.059), females exhibited no noticeable difference in inter-individual over-marking responses in relation to donor’s reproductive status. Subcaudal-proximity-marking occurred significantly more often than direct over-marking (Sign test: *P* < 0.0001, *n* = 141; 84.4% vs. 15.6% respectively), but without any significant effect of familiarity (ANOVA: *F*_2,136_ = 1.04, *P* = 0.36), location (*F*_1,136_ = 0.520, *P* = 0.47), or (donor-) sex (*F*_1,136_ = 0.45, *P* = 0.51; Table 2).

**Table 2:** Subcaudal over-marking (i.e., marking on top of the introduced AGS), and proximity-marking (i.e., within 50cm) in response to different levels of familiarity (own group, neighbour, stranger), spatial-context (sett, shared border, unshared border) and sex of donor (*n* = 141).

	Treatment	Neighbour location	N_Total_	N_Female Adult_	N_Female Yearling_	N_Male Adult_	N_Male Yearling_
**Over-mark**	Own-group		-	-	-	-	-
**(*n* = 22)**	Neighbour	Sett	-	-	-	-	-
		Shared border	2	-	2	-	-
		Unshared border	5	2	-	3	-
	Stranger		15	7	-	8	-
**Proximity-mark**	Own-group		9	5	3	1	-
**(*n* = 119)**	Neighbour	Sett	-	-	-	-	-
		Shared border	7	1	2	3	1
		Unshared border	35	16	3	16	-
	Stranger		68	33	8	18	9

#### Sniffing

While familiarity and location were the most supported parameters determining the time spent investigating introduced AGS (S1 Table), the influence of age and sex of the scent donor were also important (Table 3). Donor reproductive status as well as the interaction terms, however, had relatively little influence. Indeed, the most supported model for sniff-duration included level of familiarity, location at which the sample was presented, donor sex and age, and interaction terms between these (*w* = 0.118). The model that excluded individual characteristics (i.e., including only status: familiarity and location) had little biological significance (Δ_i_ = 4.15, *w* = 0.006) indicating the importance of considering the individual-specific information encoded in scent when predicting responses.

**Table 3:** Model averaging for the parameters linking sniffing and over-marking responses of badgers for a) the complete dataset (*n* = 351). The Relative Influence of each parameter (based on Akaike weights) is presented along with model-averaged estimated values of their coefficients (*θ*), and 95% confidence intervals (CI).

	Sniff-duration				Over-marking response			
Metric	Relative Importance	*θ*	95% CI		Relative Importance	*θ*	95% CI	
Familiarity	1.00	-0.192	-0.384	-0.001	1.00	0.256	-0.035	0.548
Location	0.98	-0.173	-0.345	-0.001	1.00	0.316	0.001	0.632
Donor Age	0.95	0.125	0.000	0.250	0.90	0.249	0.001	0.498
Donor Sex	0.89	0.036	0.000	0.072	0.88	0.108	0.000	0.216
Donor Sex*Familiarity	0.55	0.003	-0.094	0.100	0.13	0.157	0.001	0.313
Location*Donor Sex	0.50	-0.080	-0.249	0.090	0.42	-0.171	-0.656	0.313
Donor Age*Donor Sex	0.45	-0.155	-0.310	-0.001	0.29	-0.149	-0.298	0.000
Donor Age*Familiarity	0.42	0.003	-0.094	0.100	0.14	0.100	0.000	0.200
Location*Donor Age	0.39	0.035	-0.114	0.184	0.42	0.099	-0.223	0.420
Donor Reproductive Status	0.30	0.027	-0.001	0.055	0.99	0.264	0.001	0.527
Location*Familiarity	0.22	0.154	0.000	0.308	0.30	-0.637	-1.271	-0.002
Donor Age*Donor Reproductive Status	0.05	-0.019	-0.136	0.098	0.09	0.004	-0.122	0.131
Location*Donor Reproductive Status	0.05	-0.011	-0.178	0.125	0.99	-0.335	-1.072	0.402
Donor Reproductive Status*Familiarity	0.01	-0.012	-0.113	0.089	0.07	-0.208	-0.599	0.184

Asterisks (*) denote interaction terms

When just data from observations with identifiable responders were included in the models (S2 Table), familiarity was the most influential parameter, whereas sample location became relatively un-important. The responder’s reproductive status had a strong effect on sniff-duration, with the responder’s age being of intermediate importance (Table 4). Indeed, the most supported model predictive of sniff-duration included only familiarity, and the responder’s reproductive status (*w* = 0.097). The model that excluded individual characteristics again had little biological significance (Δ_i_ = 4.37, *w* = 0.011).

#### Over-marking

Familiarity and location were also the most influential parameters affecting subcaudal-marking responses when analysing the complete dataset. Unlike sniffing responses, however, the reproductive status of the scent-donor, and its interaction with location were highly influential. The influence of donor age and sex were of moderate importance, while the interaction terms had relatively little influence (Table 3). The model that excluded individual characteristics had little biological significance (Δ_i_ = 16.77, *w* = 0.00), while the most supported models all included these characteristics and their interaction with location (S3 Table).

When models based on the restricted dataset (S4 Table) were ranked, familiarity and location remained the most influential parameters, with the responder’s reproductive status being highly influential (Table 4). Unlike sniffing responses, the most supported model predictive of subcaudal-marking response included familiarity as well as location of the AGS sample in addition to the responder’s reproductive status, (*w* = 0.113).

**Table 4:** Model averaging for the parameters linking sniffing and over-marking responses of badgers for the restricted dataset (*n* = 187) including responder characteristics within the linear model. The Relative Influence of each parameter (based on Akaike weights) is presented along with model-averaged estimated values of their coefficients (*θ*), and 95% confidence intervals (CI). Asterisks (*) denote interaction terms.

Metric	Relative Importance	*θ*	95% CI		Relative Importance	*θ*	95% CI	
Familiarity	1.00	-0.104	-0.207	-0.001	1.00	0.083	-0.233	0.398
Responder Reproductive Status	0.74	-0.043	-0.156	0.070	0.68	-0.160	-0.452	0.132
Responder Age	0.51	0.185	0.001	0.370	0.27	-0.059	-0.118	0.000
Responder Age*Familiarity	0.38	-0.161	-0.374	0.052	0.01	-0.006	-0.054	0.041
Location	0.33	-0.142	-0.284	-0.001	0.990	0.177	-0.039	0.392
Responder Sex	0.30	-0.078	-0.155	-0.001	0.34	0.183	0.001	0.365
Donor Age	0.28	0.103	0.001	0.205	0.14	0.034	0.000	0.067
Responder Reproductive Status*Familiarity	0.27	0.029	-0.167	0.226	0.01	0.080	-0.176	0.336
Donor Sex	0.25	0.077	0.000	0.154	0.36	0.116	0.001	0.232
Location*Familiarity	0.14	-0.274	-0.274	-0.002	0.13	-0.185	-0.369	-0.002
Responder Sex*Familiarity	0.06	0.022	-0.118	0.163	0.05	0.321	0.002	0.640
Location*Donor Sex	0.05	0.064	-0.152	0.280	0.19	0.153	-0.245	0.551
Responder Age*Responder Reproductive Status	0.05	-0.319	-0.635	-0.003	0.08	-0.865	-1.722	-0.007
Donor Sex*Familiarity	0.04	-0.207	-0.413	-0.002	0.02	-0.242	-0.483	-0.002
Responder Age*Donor Age	0.02	-0.472	-0.940	-0.003	0.01	0.665	0.005	1.324
Responder Reproductive Status*Donor Sex	0.01	-0.092	-0.183	-0.001	0.01	-0.200	-0.398	-0.002
Donor Age*Familiarity	0.01	-0.032	-0.063	0.000	0.01	0.201	0.002	0.401
Responder Sex*Donor Sex	0.01	-0.198	-0.395	-0.002	0.00	-0.081	-0.162	-0.001
Responder Reproductive Status*Donor Age	0.01	-0.033	-0.080	0.014	0.00	-0.243	-0.484	-0.002
Location*Responder Sex	0.01	0.021	-0.211	0.254	0.14	-0.283	-0.721	0.154
Responder Age*Responder Sex	0.01	-0.067	-0.134	-0.001	0.01	-0.485	-0.965	-0.004
Responder Sex*Donor Age	0.01	0.100	0.001	0.199	0.00	-0.328	-0.653	-0.003
Location*Responder Reproductive Status	0.01	0.133	-0.201	0.468	0.04	-0.012	-0.278	0.255
Donor Age*Donor Sex	0.00	-0.173	-0.344	-0.001	0.00	-0.397	-0.792	-0.003
Location*Responder Age	0.00	-0.135	-0.268	-0.001	0.01	0.060	0.000	0.120
Location*Donor Age	0.00	-0.184	-0.366	-0.002	0.01	-0.348	-0.694	-0.003
Responder Age*Donor Sex	0.00	0.086	0.001	0.171	0.01	0.665	0.005	1.324

## Discussion

Sun Tzu (6^th^ Century BCE) wrote: “If you know your enemies and know yourself, you will not be imperilled in a hundred battles… if you do not know your enemies nor yourself, you will be imperilled”–a pervasive philosophical concept in political and military theory.

This study evidences that, for badgers, and by extension other species with a comparable RDH-based social system, interpretations of territoriality depicting rigid and potentially agonistic discrimination between OG-members and members of foreign groups are overly-simplistic. As expected, we found no support for indiscriminate attempts at Active Territorial Defence [32]. The notion of ‘enemy’ therefore is increasingly contrary to the Schengen-type (movement without borders: [56]) badger society proposed by Macdonald, Newman & Buesching [25]. Instead, individuals and their scent-marks might well be recognised as non-residents; however, they are not treated as a threat at carte blanche level, but assessed on their individual-specific characteristics. Receiver responses to conspecifics are driven by the interplay of three factors: (i) the status (degree of familiarity) of the signaller, (ii) the characteristics of the signaller, and (iii) the relevance of the scent-signal to the responder. In the following, we evaluate this cascade of evidence through a filter of progressively more refined functions.

### Familiarity Hypothesis

Badgers were clearly able to discriminate between OG- and EG-AGS, reacting significantly less to more familiar individuals. Evidence of DEP [7] was also apparent in our study.

DEP is an evolved response to minimise costs associated with territoriality [57], and in badgers it could likely be the mechanism allowing certain animals to visit neighbouring territories. In this same population, the lifetime reproductive output of 193 females examined involved exclusively EG- paternity for 39%, OG for 36%, with the remaining 25% following a mixed strategy [58]. Similarly, over 5255 trapping events, 36% of badgers were never caught visiting a different social group, while at any one seasonal trapping 16.4% of the population was at a group differing from its primary affiliation [35].

Palphramand & White [44] found that badgers responded more strongly to faeces from strangers, but not neighbours, compared to OG-samples, when presented in the single context of main setts. Their results, however, are challenging to interpret because they deliberately selected faeces not to contain any visible glandular secretions. Faeces cannot encode information about group-membership *per se*, but only about the food consumed (as a potential cue for optimal foraging: [59]) or the donor’s endocrinological status [60]. In contrast, here residents also reacted stronger to neighbours, compared to OG-AGS,

### Threat level hypothesis

Although out-of-context signals from neighbours have been reported to evoke greater responses in a variety of species [7], there was little support that scent-location moderated reaction in this badger population. Instead, badgers appeared to modify their response according to the relevance of encoded individual-specific scent-donor information relative to their own characteristics and status. This indicates that badgers did not assess the level of threat posed by apparent interlopers by the positioning of their scent-marks, but rather by individual characteristics and familiarity. Notably, some responses to strangers were very intense: Stranger-AGS was repeatedly dug up, and evoked significantly elevated responsive subcaudal-marking compared with OG- and neighbour-AGS. As observed by Palphramand & White [44], longer sniff-duration was associated with less familiar AGS, likely connected to time required to attempt to identify the individual donor and their implied threat-level [61] and/or decode individual-specific information [27].

### Individual advertisement

Due to the large sample size of individually-known AGS donors and responders in this present study, the individual-specific mechanisms underscoring these group-level effects became apparent.

Mustelid-AGS encodes a variety of fitness-related individual-specific information such as sex, age and reproductive condition [62, 63]. Although Davies, Lachno & Roper [64] report limited variability in AGS-profiles of badgers, new techniques indicate that the chemical composition of badger-AGS does differ between males and females, and varies with age, reproductive status and individuality [65]. Field observations confirm that badgers can decipher this individual-specific information; mirroring responses to chemical variation in subcaudal gland secretions [52]. Nevertheless, although responsive subcaudal marking rates by males and females were similar, their motivation was likely different: in mammals females typically sequester (and thus scent-mark) food resources necessary to raise offspring, whereas males sequester access to females [66]. From their more limited data, Palphramand & White [44] reported no evidence of differences in the responses of male or female badgers to faeces deposited by an individual of unknown sex and age. Aided by knowing the sex of both, the donor and the receiver, the present study highlighted that males investigated AGS more thoroughly than did females, and sniffed and over-marked AGS from oestrous females significantly longer than AGS from non-oestrous females. This is in strong contrast to previous studies [44], and suggests an epigamic role of the female AGS signal triggering male interest [51]. Interestingly, complementary work in this same study population found that badgers, instead of simply defecating at the section of boundary closest to their current foraging site often traverse the entire group-range to place their faeces at a latrine chosen very deliberately, again implying self-advertisement [67].

### Conclusions

In overview, this study demonstrates that badgers have a thorough operational knowledge of individuals well beyond their own social group, and the apparent ability to ascertain quickly the characteristics (sex, age etc) of unfamiliar individuals. Our findings give support to the familiarity hypothesis as well as individual advertisement. The results highlight that (a) AGS encodes information pertaining to fitness-relevant parameters, and (b) that individuals have a diverse and adaptable knowledge of their neighbouring conspecifics, as well as the ability to distinguish strangers from OG and neighbours. The presented observations of how social communication is mediated in badger society reconcile well with mounting evidence of more complex inter-group interactions than realised previously [25], from both genetic pedigree (e.g. [39, 40]) and high-resolution tracking [36], which highlight that individual heterogeneities govern socio-spatial interactions.

The ability to exchange information between all conspecifics sharing a space is crucial for the maintenance of stable socio-spatial landscapes [68]. If plotted on a map, scent-sites, such as latrines, which are used for communication amongst neighbours, can easily generate the–potentially false—appearance of co-ordinated group territories, although they might not fulfil a role in central control or explicit defence [29]. Nevertheless, the resulting extent of social network connectivity affects how social and/or genetic information [69], as well as infection [70], can flow between individuals.

## Supporting Information

S1 Table Statistical summary of the models linking the duration of sniffing responses (sec.) to a global model. The global model included the variables; ‘age’ of the donor (levels: yearling, adult); ‘sex’ of the donor (levels: male, female); ‘reproductive status of the female donor’ (levels: oestrous, non-oestrous); and ‘reproductive status of the male donor’ (levels: descended, fully descended), as well as interaction terms (n = 351). Trial ID was included as a random effect in these models. This table is the basis of the model averaging, for which results are presented in Table 3 of the main text. The support for each model, based on Akaike criterion, is presented in the first three columns. The fourth column presents the degrees of freedom associated with each model. Subsequent columns present coefficient estimates of the parameters included in each model.
(PDF)

S2 Table Statistical summary of the models linking subcaudal over-marking responses to a global model. The global model included the variables; ‘age’ of the donor (levels: yearling, adult); ‘sex’ of the donor (levels: male, female); ‘reproductive status of the female donor’ (levels: oestrous, non-oestrous); and ‘reproductive status of the male donor’ (levels: descended, fully descended), as well as interaction terms (n = 351). Trial ID was included as a random effect in these models. This table is the basis of the model averaging, for which results are presented in Table 2 of the main text. The support for each model, based on Akaike criterion, is presented in the first three columns. The fourth column presents the degrees of freedom associated with each model. Subsequent columns present coefficient estimates of the parameters included in each model.
(PDF)

S3 Table Statistical summary of the models linking the duration of sniffing responses (sec.) to a global model. The global model included the variables; ‘age’ of the donor and responder (levels: yearling, adult); ‘sex’ of the donor and the responder (levels: male, female); ‘reproductive status of the female’ donor and responder (levels: oestrous, non-oestrous); and ‘reproductive status of the male’ donor and responder (levels: descended, fully descended), as well as interaction terms, as factors in these models. Responder and trial ID were included as random effects in these models. This table is the basis of the model averaging, for which results are presented in Table 4 of the main text. The support for each model, based on Akaike criterion, is presented in the first three columns. The fourth column presents the degrees of freedom associated with each model. Subsequent columns present coefficient estimates of the parameters included in each model.
(PDF)

S4 Table Statistical summary of the models linking subcaudal over-marking responses to a global model. The global model included the variables; ‘age’ of the donor and responder (levels: yearling, adult); ‘sex’ of the donor and the responder (levels: male, female); ‘reproductive status of the female’ donor and responder (levels: oestrous, non-oestrous); and ‘reproductive status of the male’ donor and responder (levels: descended, fully descended), as well as interaction terms, as factors in these models. Responder and trial ID were included as random effects in these models. This table is the basis of the model averaging, for which results are presented in Table 4 of the main text. The support for each model, based on Akaike criterion, is presented in the first three columns. The fourth column presents the degrees of freedom associated with each model. Subsequent columns present coefficient estimates of the parameters included in each model.
(PDF)

S5 Table Raw data on which all analyses are based. (DOCX)
