# Supplementary material for: Will Trespassers Be Prosecuted or Assessed According to Their Merits? A Consilient Interpretation of Territoriality in a Group-Living Carnivore, the European Badger (Meles meles)
Source: PLoS One. 2015 Jul 6;10(7):e0132432. doi: 10.1371/journal.pone.0132432 (PMC4493095; doi:10.1371/journal.pone.0132432)
Supplement: S1 Table — The global model included the variables; ‘age’ of the donor (levels: yearling, adult); ‘sex’ of the donor (levels: male, female); ‘reproductive status of the female donor’ (levels: oestrous, non-oestrous); and ‘reproductive status of the male donor’ (levels: descended, fully descended), as well as interaction terms (n = 351). Trial ID was included as a random effect in these models. This table is the basis of the model averaging, for which results are presented in Table 3 of the main text. The support for each model, based on Akaike criterion, is presented in the first three columns. The fourth column presents the degrees of freedom associated with each model. Subsequent columns present coefficient estimates of the parameters included in each model. (PDF) [file pone.0132432.s001.pdf]

Table S1

AIC	ΔAIC	*w*	d.f.	Loc.	Don. Age	Don. Sex	Fam.	Loc. Don. Sex	Don. Age*Don. Sex	Fam.*Don. Sex	Loc.*Don. Age	Fam.*Don. Age	Fam.*Loc.	Don. Rep.	Don. Age*Don. Rep.	Loc.*Don. Rep.	Fam.*Don. Rep.
198.586	0	0.044	15	-0.190	0.182	0.056	-0.164	-0.063	-0.167	0.010	-	-	-	-	-	-	-
198.996	0.410	0.035	12	-0.239	0.181	0.008	-0.170	-	-0.179	0.010	-	-	-	-	-	-	-
199.158	0.572	0.033	13	-0.277	0.083	-	-0.194	-	-	-	0.032	0.119	-	-	-	-	-
199.441	0.855	0.028	16	-0.285	0.067	0.024	-0.183	-	-	-0.019	0.056	0.135	-	-	-	-	-
199.456	0.870	0.028	13	-0.165	0.182	0.091	-0.157	-0.103	-0.164	-	-	-	-	-	-	-	-
199.727	1.140	0.025	14	-0.189	0.095	-0.001	-0.169	-0.042	-	0.032	-	-	-	-	-	-	-
200.041	1.455	0.021	17	-0.170	0.134	0.086	-0.170	-0.100	-0.175	-0.010	-	0.082	-	-	-	-	-
200.184	1.598	0.020	14	-0.255	0.150	0.011	-0.157	-	-	-0.008	0.005	-	-	-	-	-	-
200.277	1.690	0.019	15	-0.161	0.136	0.100	-0.175	-0.122	-0.173	-	-	0.082	-	-	-	-	-
200.345	1.759	0.018	17	-0.228	0.097	0.081	-0.187	-0.076	-	-	0.030	0.129	-	-	-	-	-
200.438	1.852	0.017	19	-0.244	0.083	0.061	-0.186	-0.049	-	-0.003	0.043	0.135	-	-	-	-	-
200.474	1.888	0.017	12	-0.151	0.096	0.055	-0.148	-0.098	-	-	-	-	-	-	-	-	-
200.574	1.987	0.016	11	-0.251	0.145	-	-0.165	-	-	-	0.005	-	-	-	-	-	-
200.639	2.053	0.016	11	-0.226	0.090	-0.028	-0.165	-	-	0.019	-	-	-	-	-	-	-
200.945	2.358	0.013	14	-0.247	0.155	0.009	-0.183	-	-0.187	0.011	-	0.051	-	-	-	-	-
200.956	2.370	0.013	17	-0.288	0.093	0.034	-0.184	-	-0.079	-0.020	0.062	0.128	-	-	-	-	-
201.087	2.501	0.012	14	-0.279	0.084	0.010	-0.195	-	-	-	0.034	0.121	-	-	-	-	-
201.158	2.572	0.012	8	-0.223	0.088	-	-0.157	-	-	-	-	-	-	-	-	-	-
201.167	2.581	0.012	15	-0.261	0.181	0.026	-0.159	-	-0.114	-0.011	0.019	-	-	-	-	-	-
201.268	2.682	0.011	15	-0.283	0.186	0.085	-0.265	-0.096	-0.169	-	-	-	0.123	-	-	-	-
201.366	2.780	0.011	17	-0.244	0.162	0.014	-0.175	-0.010	-	0.029	0.002	-	-	-	-	-	-
201.414	2.828	0.011	15	-0.368	0.052	-	-0.279	-	-	-	0.060	0.148	0.092	-	-	-	-
201.444	2.858	0.010	17	-0.328	0.184	0.045	-0.297	-0.054	-0.174	0.017	-	-	0.141	-	-	-	-
201.464	2.877	0.010	16	-0.165	0.033	0.030	-0.176	-0.083	-	0.010	-	0.096	-	-	-	-	-
201.591	3.005	0.010	14	-0.143	0.033	0.064	-0.170	-0.121	-	-	-	0.099	-	-	-	-	-
201.725	3.139	0.009	13	-0.187	-	-0.006	-0.174	-0.051	-	0.017	-	-	-	-	-	-	-
201.741	3.155	0.009	10	-0.234	0.172	0.012	-0.166	-	-0.169	-	-	-	-	-	-	-	-
201.812	3.225	0.009	14	-0.401	0.184	0.005	-0.331	-	-0.189	0.012	-	-	0.174	-	-	-	-
202.029	3.443	0.008	17	-0.185	0.197	-	-0.180	-0.079	-0.188	0.012	-	-	-	0.064	-	-	-
202.103	3.517	0.008	18	-0.229	0.117	0.091	-0.187	-0.079	-0.057	-	0.033	0.123	-	-	-	-	-
202.111	3.524	0.007	20	-0.242	0.104	0.079	-0.183	-0.058	-0.066	-0.010	0.049	0.131	-	-	-	-	-
202.164	3.578	0.007	17	-0.322	0.124	0.094	-0.335	-0.118	-0.178	-	-	0.103	0.171	-	-	-	-
202.179	3.593	0.007	15	-0.207	0.164	0.068	-0.156	-0.064	-	-	0.000	-	-	-	-	-	-
202.422	3.836	0.006	14	-0.201	0.099	0.051	-0.189	-0.093	-	-	-	-	0.052	-	-	-	-
202.436	3.849	0.006	10	-0.243	-	-0.032	-0.177	-	-	0.018	-	-	-	-	-	-	-
202.543	3.957	0.006	13	-0.267	0.152	-	-0.174	-	-	-	0.000	-	0.020	-	-	-	-
202.545	3.959	0.006	11	-0.159	-	0.036	-0.163	-0.097	-	-	-	-	-	-	-	-	-
202.567	3.981	0.006	12	-0.252	0.146	0.003	-0.165	-	-	-	0.005	-	-	-	-	-	-
202.613	4.026	0.006	14	-0.251	0.187	-	-0.182	-	-0.191	0.004	-	-	-	0.009	-	-	-
202.644	4.057	0.006	18	-0.241	0.189	0.042	-0.170	-0.025	-0.098	0.017	0.013	-	-	-	-	-	-
202.680	4.094	0.006	19	-0.256	0.086	0.078	-0.207	-0.074	-	-	0.041	0.142	0.027	-	-	-	-
202.713	4.126	0.006	18	-0.305	0.053	-	-0.178	-	-	-0.048	0.068	0.140	-	0.002	-	-	-
202.718	4.132	0.006	18	-0.376	0.036	0.023	-0.270	-	-	-0.021	0.083	0.161	0.093	-	-	-	-
202.732	4.146	0.005	7	-0.241	-	-	-0.169	-	-	-	-	-	-	-	-	-	-
202.769	4.183	0.005	16	-0.260	0.096	-0.006	-0.235	-0.039	-	0.034	-	-	0.075	-	-	-	-
202.794	4.207	0.005	19	-0.344	0.122	0.076	-0.346	-0.094	-0.182	-0.003	-	0.101	0.182	-	-	-	-

202.796	4.209	0.005	15	-0.281	0.105	0.017	-0.196	-	-0.062	-	0.038	0.115	-	-	-	-	-
202.895	4.308	0.005	13	-0.234	0.048	-0.030	-0.182	-	-	0.022	-	0.067	-	-	-	-	-
202.917	4.331	0.005	15	-0.150	0.200	-	-0.164	-0.125	-0.183	-	-	-	-	0.099	-	-	-
202.924	4.338	0.005	10	-0.231	0.050	-	-0.170	-	-	-	-	0.062	-	-	-	-	-
202.938	4.352	0.005	9	-0.221	0.086	-0.017	-0.156	-	-	-	-	-	-	-	-	-	-
203.074	4.487	0.005	12	-0.243	0.152	0.013	-0.177	-	-0.181	-	-	0.046	-	-	-	-	-
203.091	4.505	0.005	10	-0.297	0.090	-	-0.223	-	-	-	-	-	0.080	-	-	-	-
203.312	4.725	0.004	12	-0.395	0.177	0.009	-0.322	-	-0.178	-	-	-	0.173	-	-	-	-
203.379	4.793	0.004	16	-0.363	0.054	0.007	-0.273	-	-	-	0.060	0.148	0.085	-	-	-	-
203.419	4.833	0.004	16	-0.262	0.154	0.010	-0.160	-	-	-0.009	0.002	-	0.010	-	-	-	-
203.509	4.922	0.004	16	-0.211	0.191	0.084	-0.159	-0.070	-0.094	-	0.011	-	-	-	-	-	-
203.638	5.052	0.003	16	-0.240	0.024	0.059	-0.264	-0.118	-	-	-	0.114	0.105	-	-	-	-
203.655	5.069	0.003	21	-0.292	0.065	0.062	-0.230	-0.051	-	-0.006	0.059	0.152	0.050	-	-	-	-
203.660	5.074	0.003	19	-0.166	0.136	-	-0.179	-0.119	-0.189	-0.023	-	0.090	-	0.075	-	-	-
203.663	5.076	0.003	16	-0.445	0.142	0.004	-0.387	-	-0.197	0.015	-	0.073	0.212	-	-	-	-
203.679	5.093	0.003	16	-0.187	0.096	-	-0.174	-0.046	-	0.033	-	-	-	0.006	-	-	-
203.748	5.161	0.003	13	-0.310	0.090	-0.031	-0.247	-	-	0.019	-	-	0.092	-	-	-	-
203.820	5.234	0.003	13	-0.257	0.173	0.015	-0.169	-	-0.099	-	0.017	-	-	-	-	-	-
203.941	5.355	0.003	16	-0.266	0.147	-	-0.161	-	-	-0.019	0.010	-	-	0.005	-	-	-
203.981	5.395	0.003	19	-0.472	0.041	0.035	-0.363	-	-0.105	-0.023	0.117	0.172	0.185	-	-	-	-
204.088	5.501	0.003	17	-0.177	0.175	0.068	-0.119	-0.065	-	-	-0.009	-	-0.029	-	-	-	-
204.109	5.523	0.003	17	-0.154	0.141	-	-0.183	-0.135	-0.183	-	-	0.087	-	0.086	-	-	-
204.184	5.598	0.003	8	-	0.102	-0.025	-0.047	-	-	0.004	-	-	-	-	-	-	-
204.209	5.623	0.003	9	-	0.169	0.002	-0.046	-	-0.130	0.000	-	-	-	-	-	-	-
204.209	5.623	0.003	19	-0.309	0.080	-	-0.183	-	-0.083	-0.047	0.077	0.135	-	0.012	-	-	-
204.246	5.660	0.003	21	-0.245	0.072	-	-0.188	-0.060	-	-0.021	0.051	0.144	-	0.045	-	-	-
204.307	5.721	0.002	17	-0.360	0.180	0.024	-0.255	-	-0.128	-0.011	0.025	-	0.106	-	-	-	-
204.332	5.746	0.002	19	-0.227	0.096	-	-0.190	-0.079	-	-	0.031	0.131	-	0.058	-	-	-
204.378	5.792	0.002	8	-0.237	-	-0.022	-0.168	-	-	-	-	-	-	-	-	-	-
204.379	5.792	0.002	14	-0.141	0.099	-	-0.146	-0.105	-	-	-	-	-	0.051	-	-	-
204.398	5.812	0.002	20	-0.315	0.090	0.086	-0.264	-0.075	-0.066	-	0.061	0.148	0.083	-	-	-	-
204.435	5.849	0.002	18	-0.276	0.022	0.024	-0.287	-0.081	-	0.013	-	0.111	0.118	-	-	-	-
204.464	5.878	0.002	19	-0.231	0.168	0.020	-0.159	-0.016	-	0.025	-0.004	-	-0.007	-	-	-	-
204.465	5.879	0.002	16	-0.262	0.154	-	-0.185	-	-0.193	-0.007	-	0.051	-	-0.002	-	-	-
204.491	5.905	0.002	5	-	0.101	-	-0.048	-	-	-	-	-	-	-	-	-	-
204.495	5.909	0.002	13	-0.233	0.087	-	-0.163	-	-	0.007	-	-	-	-0.026	-	-	-
204.542	5.956	0.002	14	-0.267	0.152	0.001	-0.173	-	-	-	0.000	-	0.018	-	-	-	-
204.651	6.065	0.002	13	-0.285	-	0.029	-0.280	-0.089	-	-	-	-	0.132	-	-	-	-
204.698	6.111	0.002	11	-0.228	0.050	-0.017	-0.170	-	-	-	-	0.061	-	-	-	-	-
204.733	6.147	0.002	17	-0.275	0.184	-	-0.170	-	-0.127	-0.020	0.025	-	-	0.020	-	-	-
204.745	6.158	0.002	15	-0.334	-	-0.020	-0.317	-0.040	-	0.026	-	-	0.150	-	-	-	-
204.773	6.187	0.002	14	-0.438	0.138	0.011	-0.373	-	-0.190	-	-	0.071	0.209	-	-	-	-
204.774	6.187	0.002	16	-0.287	0.085	-	-0.196	-	-	-	0.027	0.115	-	-0.014	-	-	-
204.791	6.205	0.002	11	-0.307	0.088	-0.020	-0.236	-	-	-	-	-	0.094	-	-	-	-
204.813	6.227	0.002	9	-0.373	-	-	-0.296	-	-	-	-	-	0.143	-	-	-	-
204.842	6.256	0.002	17	-0.264	0.201	-	-0.266	-0.115	-0.186	-	-	-	0.116	0.090	-	-	-
204.925	6.339	0.002	17	-0.438	0.060	0.015	-0.346	-	-0.083	-	0.085	0.156	0.158	-	-	-	-
204.983	6.397	0.002	19	-0.312	0.199	-	-0.301	-0.069	-0.192	0.020	-	-	0.130	0.056	-	-	-
205.000	6.414	0.002	12	-0.339	0.040	-	-0.276	-	-	-	-	0.079	0.117	-	-	-	-
205.098	6.512	0.002	17	-0.135	0.147	-	-0.161	-0.130	-	-	-	-	-	0.080	-0.029	-	-

205.126	6.540	0.002	19	-0.239	0.171	-	-0.187	-0.018	-	0.036	-0.004	-	-	0.025	-	-	-
205.223	6.637	0.002	22	-0.361	0.069	0.079	-0.296	-0.056	-0.081	-0.012	0.086	0.161	0.119	-	-	-	-
205.391	6.805	0.001	18	-0.165	0.029	-	-0.174	-0.088	-	-0.003	-	0.098	-	0.018	-	-	-
205.461	6.875	0.001	12	-0.389	-	-0.037	-0.323	-	-	0.020	-	-	0.158	-	-	-	-
205.481	6.895	0.001	18	-0.249	0.195	0.080	-0.188	-0.067	-0.095	-	0.008	-	0.040	-	-	-	-
205.496	6.910	0.001	15	-0.189	-	-	-0.168	-0.057	-	-0.003	-	-	-	-0.010	-	-	-
205.515	6.929	0.001	19	-0.169	0.169	-	-0.171	-0.093	-	0.002	-	-	-	0.063	-0.078	-	-
205.517	6.930	0.001	16	-0.397	0.189	-	-0.328	-	-0.198	0.006	-	-	0.159	0.007	-	-	-
205.582	6.996	0.001	16	-0.143	0.034	-	-0.167	-0.119	-	-	-	0.098	-	0.041	-	-	-
205.683	7.096	0.001	12	-0.239	0.174	-	-0.173	-	-0.173	-	-	-	-	0.008	-	-	-
205.720	7.133	0.001	15	-0.351	0.175	0.013	-0.256	-	-0.111	-	0.019	-	0.102	-	-	-	-
205.732	7.146	0.001	20	-0.304	0.191	0.040	-0.228	-0.023	-0.104	0.018	0.015	-	0.067	-	-	-	-
205.807	7.221	0.001	22	-0.241	0.095	-	-0.188	-0.075	-0.079	-0.029	0.060	0.142	-	0.065	-	-	-
205.887	7.300	0.001	12	-0.253	-	-	-0.166	-	-	-0.006	-	-	-	-0.037	-	-	-
205.901	7.315	0.001	17	-0.190	0.175	-	-0.157	-0.080	-	-	-0.007	-	-	0.072	-	-	-
205.948	7.362	0.001	15	-0.357	0.036	-0.035	-0.307	-	-	0.024	-	0.083	0.133	-	-	-	-
206.000	7.414	0.001	20	-0.369	0.031	-	-0.237	-	-	-0.050	0.088	0.159	0.065	0.001	-	-	-
206.014	7.428	0.001	19	-0.311	0.128	-	-0.339	-0.131	-0.188	-	-	0.107	0.168	0.081	-	-	-
206.042	7.456	0.001	20	-0.226	0.117	-	-0.193	-0.087	-0.064	-	0.037	0.128	-	0.071	-	-	-
206.086	7.500	0.001	6	-	0.099	-0.023	-0.049	-	-	-	-	-	-	-	-	-	-
206.139	7.553	0.001	22	-0.226	0.190	-	-0.196	-	-0.178	0.026	-	-	-	0.034	-	-0.026	-
206.154	7.567	0.001	20	-0.233	0.206	-	-0.186	-0.041	-0.117	0.021	0.007	-	-	0.054	-	-	-
206.313	7.726	0.001	16	-0.247	0.166	-	-0.180	-	-	0.003	-	-	-	0.003	-0.097	-	-
206.325	7.739	0.001	10	-0.384	-	-0.025	-0.310	-	-	-	-	-	0.159	-	-	-	-
206.373	7.786	0.001	16	-0.195	0.101	-	-0.188	-0.098	-	-	-	-	0.053	0.044	-	-	-
206.437	7.851	0.001	15	-0.244	0.045	-	-0.169	-	-	-0.002	-	0.063	-	-0.036	-	-	-
206.459	7.873	0.001	7	-	0.159	-0.002	-0.051	-	-0.119	-	-	-	-	-	-	-	-
206.494	7.908	0.001	21	-0.326	0.125	-	-0.341	-0.112	-0.194	-0.014	-	0.107	0.168	0.068	-	-	-
206.515	7.929	0.001	13	-0.163	-	-	-0.162	-0.092	-	-	-	-	-	0.016	-	-	-
206.532	7.946	0.001	17	-0.290	0.104	-	-0.199	-	-0.058	-	0.033	0.111	-	-0.007	-	-	-
206.555	7.969	0.001	14	-0.255	0.146	-	-0.167	-	-	-	0.006	-	-	0.000	-	-	-
206.660	8.074	0.001	21	-0.252	0.085	-	-0.206	-0.076	-	-	0.041	0.144	0.023	0.055	-	-	-
206.684	8.097	0.001	13	-0.350	0.038	-0.020	-0.289	-	-	-	-	0.079	0.131	-	-	-	-
206.692	8.105	0.001	11	-	0.155	0.003	-0.051	-	-0.139	-0.001	-	0.032	-	-	-	-	-
206.743	8.156	0.001	18	-0.255	0.097	-	-0.235	-0.042	-	0.035	-	-	0.071	0.001	-	-	-
206.755	8.169	0.001	7	-	0.076	-	-0.054	-	-	-	-	0.044	-	-	-	-	-
206.774	8.188	0.001	26	-0.354	0.059	-	-0.217	-	-	-0.002	0.076	0.139	-	-0.028	-	0.051	-
206.822	8.236	0.001	11	-0.223	0.084	-	-0.153	-	-	-	-	-	-	-0.023	-	-	-
206.840	8.254	0.001	14	-0.252	0.147	-	-0.181	-	-0.177	-	-	0.046	-	-0.008	-	-	-
206.912	8.325	0.001	10	-	0.075	-0.025	-0.054	-	-	0.004	-	0.046	-	-	-	-	-
207.018	8.432	0.001	18	-0.191	0.210	-	-0.165	-0.093	-0.111	-	0.003	-	-	0.093	-	-	-
207.029	8.443	0.001	21	-0.316	0.131	-	-0.198	-	-	-0.040	0.021	0.176	-	0.019	-0.103	-	-
207.057	8.470	0.001	18	-0.356	0.060	-	-0.259	-	-	-	0.049	0.139	0.070	-0.016	-	-	-
207.065	8.479	0.001	7	-	-	-0.027	-0.047	-	-	-0.002	-	-	-	-	-	-	-
207.102	8.515	0.001	24	-0.318	0.079	-	-0.205	-	-	-	0.058	0.130	-	-0.001	-	0.018	-
207.109	8.522	0.001	24	-0.251	0.131	-	-0.205	-	-0.174	0.004	-	0.076	-	0.002	-	-0.006	-
207.151	8.565	0.001	18	-0.256	0.152	-	-0.146	-	-	-0.022	0.006	-	-0.009	0.004	-	-	-
207.177	8.590	0.001	4	-	-	-	-0.052	-	-	-	-	-	-	-	-	-	-
207.205	8.619	0.001	20	-0.172	0.194	-	-0.168	-	-0.176	-	-	-	-	0.086	-	-0.083	-
207.264	8.677	0.001	14	-0.394	0.178	-	-0.322	-	-0.181	-	-	-	0.167	0.005	-	-	-

207.306	8.719	0.001	18	-0.439	0.142	-	-0.368	-	-0.201	-0.001	-	0.071	0.191	-0.004	-	-	-
207.313	8.727	0.001	21	-0.464	0.036	-	-0.334	-	-0.105	-0.049	0.123	0.173	0.157	0.014	-	-	-
207.383	8.796	0.001	21	-0.247	0.093	-	-0.198	-	-	0.058	-	-	-	-0.043	-	0.020	-
207.462	8.876	0.001	23	-0.275	0.060	-	-0.213	-0.063	-	-0.025	0.062	0.157	0.033	0.046	-	-	-
207.596	9.010	> 0.001	15	-0.308	0.087	-	-0.233	-	-	0.006	-	-	0.082	-0.029	-	-	-
207.619	9.033	> 0.001	21	-0.168	0.137	-	-0.181	-0.119	-	-0.023	-	0.101	-	0.077	-0.136	-	-
207.629	9.042	> 0.001	18	-0.243	0.024	-	-0.264	-0.116	-	-	-	0.113	0.107	0.036	-	-	-
207.688	9.102	> 0.001	22	-0.217	0.134	-	-0.191	-	-0.170	-	-	0.077	-	0.031	-	-0.041	-
207.756	9.170	> 0.001	15	-0.262	0.175	-	-0.175	-	-0.104	-	0.018	-	-	0.012	-	-	-
207.822	9.236	> 0.001	19	-0.251	0.160	-	-0.264	-0.120	-	-	-	-	0.116	0.077	-0.054	-	-
207.851	9.265	> 0.001	19	-0.146	0.127	-	-0.178	-0.136	-	-	-	0.072	-	0.080	-0.074	-	-
207.855	9.269	> 0.001	10	-	0.103	-	-0.044	-	-	0.012	-	-	-	-0.016	-	-	-
207.857	9.271	> 0.001	19	-0.150	0.185	-	-0.109	-0.080	-	-	-0.017	-	-0.043	0.070	-	-	-
207.921	9.334	> 0.001	19	-0.356	0.182	-	-0.247	-	-0.137	-0.020	0.029	-	0.088	0.018	-	-	-
207.942	9.356	> 0.001	10	-0.243	-	-	-0.165	-	-	-	-	-	-	-0.038	-	-	-
207.965	9.379	> 0.001	11	-	0.171	-	-0.050	-	-0.129	0.013	-	-	-	0.007	-	-	-
208.011	9.425	> 0.001	14	-0.230	0.125	-	-0.169	-	-	-	-	-	-	-0.010	-0.030	-	-
208.144	9.558	> 0.001	13	-0.235	0.046	-	-0.164	-	-	-	-	0.057	-	-0.039	-	-	-
208.236	9.650	> 0.001	21	-0.208	0.178	-	-0.152	-0.027	-	0.028	-0.011	-	-0.025	0.030	-	-	-
208.260	9.673	> 0.001	23	-0.271	0.033	-	-0.208	-	-	0.034	-	0.079	-	-0.073	-	0.038	-
208.322	9.736	> 0.001	8	-	0.075	-0.024	-0.055	-	-	-	-	0.041	-	-	-	-	-
208.326	9.740	> 0.001	22	-0.313	0.090	-	-0.271	-0.082	-0.074	-	0.065	0.154	0.084	0.068	-	-	-
208.352	9.766	> 0.001	18	-0.267	0.164	-	-0.189	-	-	-0.007	-	0.062	-	0.003	-0.162	-	-
208.375	9.789	> 0.001	9	-	0.151	0.000	-0.054	-	-0.131	-	-	0.029	-	-	-	-	-
208.380	9.794	> 0.001	19	-0.276	0.146	-	-0.163	-	-	-0.027	0.053	-	-	0.012	-0.039	-	-
208.385	9.798	> 0.001	20	-0.269	0.019	-	-0.277	-0.085	-	0.002	-	0.111	0.110	0.014	-	-	-
208.401	9.815	> 0.001	19	-0.174	0.096	-	-0.152	-	-	-	-	-	-	0.030	-	-0.057	-
208.508	9.922	> 0.001	16	-0.266	0.151	-	-0.172	-	-	-	0.000	-	0.014	-0.004	-	-	-
208.537	9.951	> 0.001	5	-	-	-0.029	-0.053	-	-	-	-	-	-	-	-	-	-
208.556	9.970	> 0.001	17	-0.321	-	-	-0.294	-0.045	-	0.006	-	-	0.135	-0.020	-	-	-
208.562	9.976	> 0.001	27	-0.346	0.075	-	-0.216	-	-0.055	-0.010	0.082	0.139	-	-0.009	-	0.038	-
208.563	9.977	> 0.001	16	-0.437	0.134	-	-0.367	-	-0.186	-	-	0.069	0.200	-0.010	-	-	-
208.581	9.995	> 0.001	15	-0.293	-	-	-0.280	-0.082	-	-	-	-	0.134	0.007	-	-	-
208.628	10.042	> 0.001	13	-0.312	0.085	-	-0.235	-	-	-	-	-	0.097	-0.027	-	-	-
208.688	10.102	> 0.001	19	-0.426	0.063	-	-0.330	-	-0.077	-	0.075	0.149	0.137	-0.007	-	-	-
208.702	10.115	> 0.001	21	-0.218	0.034	-	-0.178	-	-	-	-	0.085	-	-0.022	-	-0.018	-
208.711	10.125	> 0.001	21	-0.295	0.177	-	-0.291	-0.079	-	0.012	-	-	0.125	0.055	-0.093	-	-
208.795	10.209	> 0.001	22	-0.181	0.123	-	-0.168	-	-	-	-	-	-	0.036	-0.001	-0.053	-
208.809	10.222	> 0.001	24	-0.252	0.132	-	-0.203	-0.068	-	-0.021	0.017	0.183	-	0.065	-0.084	-	-
208.856	10.269	> 0.001	24	-0.291	0.160	-	-0.200	-	-	0.044	0.017	-	-	0.000	-	0.021	-
208.903	10.317	> 0.001	20	-0.267	-	-	-0.201	-	-	0.027	-	-	-	-0.069	-	0.029	-
208.934	10.348	> 0.001	14	-0.381	-	-	-0.293	-	-	-0.004	-	-	0.140	-0.041	-	-	-
208.935	10.348	> 0.001	24	-0.349	0.064	-	-0.290	-0.074	-0.093	-0.030	0.093	0.171	0.108	0.065	-	-	-
208.974	10.388	> 0.001	25	-0.314	0.094	-	-0.207	-	-0.042	-	0.061	0.129	-	0.010	-	0.010	-
208.978	10.392	> 0.001	22	-0.288	0.196	-	-0.270	-	-0.179	-	-	-	0.114	0.082	-	-0.080	-
209.008	10.422	> 0.001	24	-0.352	0.191	-	-0.315	-	-0.182	0.031	-	-	0.128	0.031	-	-0.024	-
209.083	10.496	> 0.001	20	-0.222	0.212	-	-0.185	-0.088	-0.110	-	0.001	-	0.030	0.087	-	-	-
209.288	10.702	> 0.001	20	-0.199	0.133	-	-0.160	-0.086	-	-	0.059	-	-	0.069	0.023	-	-
209.295	10.709	> 0.001	24	-0.224	0.147	-	-0.192	-	-	0.022	-	-	-	0.010	-0.049	-0.016	-
209.305	10.719	> 0.001	22	-0.281	0.207	-	-0.228	-0.040	-0.120	0.021	0.008	-	0.052	0.052	-	-	-

209.307	10.721	> 0.001	26	-0.347	0.068	-	-0.227	-	-	-	0.068	0.144	0.025	0.003	-	0.012	-
209.336	10.750	> 0.001	8	-	0.099	-	-0.037	-	-	-	-	-	-	-0.013	-	-	-
209.377	10.791	> 0.001	18	-0.392	0.174	-	-0.324	-	-	0.005	-	-	0.156	0.002	-0.111	-	-
209.520	10.933	> 0.001	17	-0.352	0.034	-	-0.279	-	-	0.000	-	0.078	0.118	-0.039	-	-	-
209.598	11.012	> 0.001	24	-0.359	0.122	-	-0.335	-	-0.175	-	-	0.097	0.153	0.036	-	-0.051	-
209.656	11.070	> 0.001	17	-0.350	0.177	-	-0.256	-	-0.114	-	0.020	-	0.095	0.009	-	-	-
209.692	11.106	> 0.001	23	-0.416	0.116	-	-0.294	-	-	-0.041	0.036	0.212	0.100	0.021	-0.125	-	-
209.717	11.131	> 0.001	22	-0.230	0.093	-	-0.198	-0.089	-	-	0.051	0.161	-	0.071	-0.013	-	-
209.734	11.147	> 0.001	21	-0.193	0.208	-	-0.181	-0.077	-0.198	-	-	-	-	0.077	-	-	-0.019
209.743	11.157	> 0.001	22	-0.227	0.166	-	-0.176	-0.049	-	0.009	0.036	-	-	0.050	-0.027	-	-
209.806	11.220	> 0.001	12	-0.387	-	-	-0.303	-	-	-	-	-	0.156	-0.042	-	-	-
209.834	11.248	> 0.001	22	-0.222	0.169	-	-0.162	-	-	-	0.009	-	-	0.065	-	-0.050	-
209.861	11.275	> 0.001	9	-	0.159	-	-0.042	-	-0.115	-	-	-	-	0.008	-	-	-
209.912	11.326	> 0.001	17	-0.264	0.097	-	-0.171	-	-	-	0.073	-	-	-0.009	0.029	-	-
209.929	11.343	> 0.001	28	-0.386	0.044	-	-0.245	-	-	-0.012	0.089	0.155	0.037	-0.017	-	0.039	-
209.968	11.382	> 0.001	21	-0.317	0.120	-	-0.348	-0.133	-	-	-	0.113	0.177	0.080	-0.109	-	-
209.969	11.383	> 0.001	26	-0.396	0.121	-	-0.356	-	-0.180	0.008	-	0.094	0.158	0.005	-	-0.013	-
210.041	11.454	> 0.001	25	-0.279	0.194	-	-0.198	-	-0.107	0.029	0.024	-	-	0.031	-	-0.001	-
210.115	11.529	> 0.001	15	-0.356	0.035	-	-0.283	-	-	-	-	0.075	0.131	-0.042	-	-	-
210.191	11.605	> 0.001	18	-0.252	0.199	-	-0.193	-	-0.203	-	-	-	-	-0.010	-	-	0.024
210.226	11.639	> 0.001	23	-0.353	0.128	-	-0.369	-0.111	-	-0.013	-	0.137	0.190	0.072	-0.160	-	-
210.237	11.651	> 0.001	21	-0.233	0.098	-	-0.196	-	-	-	-	-	0.052	0.027	-	-0.055	-
210.255	11.669	> 0.001	18	-0.213	-	-	-0.173	-	-	-	-	-	-	-0.018	-	-0.028	-
210.289	11.703	> 0.001	19	-0.294	0.088	-	-0.204	-	-	-	0.041	0.140	-	-0.008	-0.018	-	-
210.370	11.784	> 0.001	16	-0.386	0.141	-	-0.319	-	-	-	-	-	0.166	-0.008	-0.057	-	-
210.372	11.785	> 0.001	23	-0.320	0.093	-	-0.265	-	-	0.057	-	-	0.076	-0.042	-	0.017	-
210.479	11.893	> 0.001	12	-	0.075	-	-0.042	-	-	0.002	-	0.044	-	-0.025	-	-	-
210.483	11.896	> 0.001	13	-	0.153	-	-0.045	-	-0.133	0.001	-	0.032	-	-0.001	-	-	-
210.509	11.922	> 0.001	22	-0.310	0.053	-	-0.186	-	-	-	0.068	0.149	-	-0.004	-	-	-0.023
210.602	12.016	> 0.001	16	-0.245	0.139	-	-0.175	-	-	-	-	0.024	-	-0.013	-0.083	-	-
210.642	12.055	> 0.001	9	-	-	-	-0.034	-	-	-0.008	-	-	-	-0.028	-	-	-
210.747	12.161	> 0.001	23	-0.307	0.024	-	-0.265	-	-	-	-	0.100	0.095	-0.018	-	-0.026	-
210.929	12.343	> 0.001	20	-0.464	0.153	-	-0.393	-	-	-0.001	-	0.102	0.208	0.004	-0.186	-	-
211.080	12.493	> 0.001	23	-0.219	0.202	-	-0.169	-	-0.102	-	0.016	-	-	0.086	-	-0.062	-
211.100	12.514	> 0.001	26	-0.250	0.127	-	-0.205	-	-	0.003	-	0.077	-	0.002	-0.108	-0.007	-
211.160	12.574	> 0.001	24	-0.216	0.115	-	-0.184	-	-	-	-	0.049	-	0.012	-0.048	-0.029	-
211.162	12.575	> 0.001	27	-0.383	0.072	-	-0.269	-	-0.049	-	0.083	0.151	0.065	0.014	-	0.005	-
211.229	12.643	> 0.001	13	-	0.134	-	-0.049	-	-	0.011	-	-	-	-0.007	-0.017	-	-
211.289	12.702	> 0.001	25	-0.366	0.024	-	-0.306	-	-	0.033	-	0.094	0.106	-0.068	-	0.029	-
211.399	12.812	> 0.001	10	-	0.075	-	-0.039	-	-	-	-	0.038	-	-0.027	-	-	-
211.421	12.835	> 0.001	23	-0.171	0.140	-	-0.180	-0.121	-0.197	-	-	0.096	-	0.090	-	-	-0.041
211.455	12.869	> 0.001	26	-0.315	0.134	-	-0.261	-0.068	-	-0.021	0.016	0.209	0.062	0.067	-0.110	-	-
211.466	12.880	> 0.001	20	-0.191	0.094	-	-0.171	-0.046	-	-	-	-	-	0.018	-	-	-0.010
211.546	12.960	> 0.001	11	-	0.098	-	-0.042	-	-	-	-	-	-	-0.015	0.038	-	-
211.569	12.982	> 0.001	20	-0.263	0.163	-	-0.173	-	-	-	-0.009	-	-	-0.019	-	-	0.013
211.579	12.992	> 0.001	24	-0.289	0.139	-	-0.268	-	-	-	-	-	0.110	0.043	-0.027	-0.057	-
211.594	13.008	> 0.001	24	-0.191	0.180	-	-0.122	-	-	-	-0.001	-	-0.038	0.068	-	-0.054	-
211.606	13.019	> 0.001	25	-0.242	0.204	-	-0.204	-	-0.190	-	-	-	-	0.050	-	-0.030	-
211.659	13.073	> 0.001	29	-0.431	0.048	-	-0.298	-	-0.066	-0.017	0.110	0.165	0.090	0.001	-	0.027	-
211.694	13.108	> 0.001	24	-0.327	0.092	-	-0.288	-0.085	-	-	0.053	0.198	0.094	0.072	-0.049	-	-

211.731	13.145	> 0.001	21	-0.371	0.152	-	-0.257	-	-	-0.025	0.053	-	0.103	0.012	-0.058	-	-
211.732	13.146	> 0.001	7	-	-	-	-0.038	-	-	-	-	-	-	-0.029	-	-	-
211.755	13.168	> 0.001	11	-	0.146	-	-0.042	-	-0.122	-	-	0.028	-	-0.006	-	-	-
211.760	13.174	> 0.001	29	-0.342	0.082	-	-0.226	-	-	-0.018	0.063	0.190	-	0.013	-0.041	0.018	-
211.864	13.277	> 0.001	26	-0.265	0.167	-	-0.172	-	-	0.032	0.010	-	-0.018	0.013	-	0.006	-
211.876	13.290	> 0.001	22	-0.399	-	-	-0.326	-	-	0.034	-	-	0.134	-0.075	-	0.033	-
211.921	13.334	> 0.001	25	-0.258	0.073	-	-0.186	-0.055	-	-	0.055	0.148	-	0.069	-	-	-0.053
211.921	13.335	> 0.001	23	-0.315	0.086	-	-0.194	-	-0.091	-	0.073	0.144	-	0.006	-	-	-0.020
212.109	13.523	> 0.001	21	-0.441	0.065	-	-0.346	-	-	-	0.064	0.192	0.147	-0.005	-0.051	-	-
212.163	13.576	> 0.001	20	-0.339	-	-	-0.285	-	-	-	-	-	0.125	-0.023	-	-0.025	-
212.186	13.599	> 0.001	22	-0.262	0.151	-	-0.217	-0.081	-	-	0.049	-	0.066	0.068	-0.004	-	-
212.199	13.613	> 0.001	17	-0.231	0.086	-	-0.172	-	-	-	-	-	-	-0.052	-	-	0.027
212.210	13.624	> 0.001	21	-0.272	0.209	-	-0.184	-	-0.137	-	0.000	-	-	-0.002	-	-	0.014
212.255	13.669	> 0.001	20	-0.263	0.157	-	-0.196	-	-0.200	-	-	0.060	-	-0.015	-	-	0.011
212.340	13.753	> 0.001	29	-0.362	0.079	-	-0.226	-	-	-	0.056	0.137	-	-0.015	-	0.046	-
212.476	13.890	> 0.001	23	-0.320	0.214	-	-0.300	-0.064	-0.206	-	-	-	0.129	0.070	-	-	-0.021
212.504	13.918	> 0.001	25	-0.281	0.080	-	-0.170	-	-	-	0.113	-	-	0.009	0.066	0.007	-
212.510	13.924	> 0.001	26	-0.347	0.157	-	-0.310	-	-	0.029	-	-	0.125	0.011	-0.065	-0.015	-
212.535	13.949	> 0.001	27	-0.327	0.039	-	-0.215	-	-	-	0.103	0.164	-	0.005	0.032	0.014	-
212.561	13.975	> 0.001	18	-0.438	0.132	-	-0.368	-	-	-	-	0.069	0.201	-0.010	-0.120	-	-
212.619	14.032	> 0.001	19	-0.385	0.112	-	-0.288	-	-	-	0.069	-	0.131	-0.007	-0.002	-	-
212.691	14.104	> 0.001	27	-0.261	0.139	-	-0.214	-	-0.187	-	-	0.083	-	0.019	-	-0.013	-
212.821	14.234	> 0.001	23	-0.246	0.182	-	-0.187	-0.014	-	-	-0.011	-	-	0.039	-	-	-0.007
212.880	14.294	> 0.001	19	-0.189	-	-	-0.163	-0.062	-	-	-	-	-	0.007	-	-	-0.034
212.955	14.368	> 0.001	25	-0.252	0.205	-	-0.189	-	-0.099	-	0.013	-	0.027	0.084	-	-0.062	-
212.955	14.369	> 0.001	20	-0.394	0.205	-	-0.333	-	-0.213	-	-	-	0.155	-0.012	-	-	0.019
213.084	14.498	> 0.001	27	-0.326	0.194	-	-0.239	-	-0.109	0.024	0.025	-	0.051	0.035	-	-0.006	-
213.094	14.507	> 0.001	24	-0.249	0.094	-	-0.200	-	-	-	-	-	-	-0.028	-	0.005	-
213.108	14.522	> 0.001	24	-0.293	0.177	-	-0.236	-0.044	-	0.014	0.032	-	0.066	0.048	-0.045	-	-
213.157	14.570	> 0.001	23	-0.177	0.178	-	-0.171	-0.089	-	-	-	-	-	0.078	-0.082	-	-0.031
213.160	14.574	> 0.001	22	-0.171	0.029	-	-0.169	-0.087	-	-	-	0.091	-	0.031	-	-	-0.033
213.167	14.581	> 0.001	27	-0.306	0.120	-	-0.193	-	-	0.021	0.083	-	-	-0.006	0.013	0.031	-
213.293	14.706	> 0.001	16	-0.249	-	-	-0.174	-	-	-	-	-	-	-0.060	-	-	0.013
213.441	14.854	> 0.001	26	-0.251	0.101	-	-0.188	-0.073	-0.084	-	0.061	0.149	-	0.087	-	-	-0.054
213.498	14.912	> 0.001	26	-0.360	0.110	-	-0.337	-	-	-	-	0.093	0.157	0.030	-0.083	-0.048	-
213.697	15.111	> 0.001	24	-0.353	0.042	-	-0.226	-	-	-	0.077	0.164	0.045	-0.005	-	-	-0.028
213.731	15.145	> 0.001	24	-0.236	0.227	-	-0.188	-0.040	-0.126	-	-0.008	-	-	0.067	-	-	-0.011
213.841	15.255	> 0.001	28	-0.409	0.119	-	-0.376	-	-	0.004	-	0.117	0.175	0.016	-0.134	-0.024	-
213.877	15.291	> 0.001	27	-0.398	0.211	-	-0.348	-	-0.199	-	-	-	0.154	0.059	-	-0.038	-
213.909	15.323	> 0.001	20	-0.248	0.179	-	-0.189	-	-	-	-	-	-	-0.014	-0.103	-	0.018
214.045	15.459	> 0.001	30	-0.353	0.099	-	-0.226	-	-0.066	-	0.062	0.139	-	0.008	-	0.031	-
214.069	15.483	> 0.001	25	-0.331	0.134	-	-0.342	-0.113	-0.207	-	-	0.116	0.167	0.084	-	-	-0.039
214.070	15.484	> 0.001	26	-0.272	0.036	-	-0.208	-	-	-	-	0.077	-	-0.062	-	0.026	-
214.198	15.612	> 0.001	19	-0.242	0.043	-	-0.176	-	-	-	-	0.062	-	-0.056	-	-	0.014
214.289	15.703	> 0.001	27	-0.298	0.179	-	-0.209	-	-	-	-0.003	-	-	0.019	-	0.008	-
214.334	15.747	> 0.001	31	-0.407	0.081	-	-0.294	-	-	-0.029	0.064	0.227	0.076	0.038	-0.070	-0.006	-
214.396	15.809	> 0.001	15	-	0.143	-	-0.044	-	-	0.002	-	0.024	-	-0.006	-0.058	-	-
214.471	15.884	> 0.001	22	-0.262	0.097	-	-0.234	-0.039	-	-	-	-	0.073	0.015	-	-	-0.015
214.522	15.936	> 0.001	25	-0.326	0.125	-	-0.216	-	-	-	0.026	0.202	-	0.006	-0.098	-	0.000
214.537	15.950	> 0.001	27	-0.245	0.159	-	-0.202	-	-	-	-	-	-	0.026	-0.052	-0.017	-

214.554	15.968	> 0.001	29	-0.436	0.034	-	-0.328	-	-	-	0.108	0.210	0.113	0.023	-0.005	-0.003	-
214.565	15.978	> 0.001	23	-0.255	-	-	-0.195	-	-	-	-	-	-	-0.054	-	0.007	-
214.575	15.989	> 0.001	22	-0.225	0.178	-	-0.130	-	-	-	-0.024	-	-0.038	-0.018	-	-	0.006
214.846	16.260	> 0.001	13	-	0.123	-	-0.038	-	-	-	-	-0.007	-	-0.018	0.003	-	-
214.924	16.337	> 0.001	25	-0.450	0.052	-	-0.327	-	-0.112	-	0.109	0.179	0.139	0.007	-	-	-0.024
214.931	16.344	> 0.001	22	-0.443	0.147	-	-0.386	-	-0.213	-	-	0.084	0.197	-0.018	-	-	0.013
214.959	16.372	> 0.001	27	-0.265	0.074	-	-0.190	-0.061	-	-	0.054	0.158	0.011	0.073	-	-	-0.061
214.959	16.373	> 0.001	14	-	0.100	-	-0.064	-	-	-	-	-	-	-0.062	-	-	0.047
214.969	16.383	> 0.001	15	-	0.178	-	-0.072	-	-0.138	-	-	-	-	-0.036	-	-	0.046
215.012	16.426	> 0.001	29	-0.433	0.134	-	-0.388	-	-0.197	-	-	0.103	0.181	0.037	-	-0.031	-
215.020	16.433	> 0.001	31	-0.373	0.082	-	-0.235	-	-	-	0.052	0.147	0.017	0.010	-	0.022	-
215.234	16.648	> 0.001	23	-0.325	0.216	-	-0.234	-	-0.143	-	-0.005	-	0.060	-0.003	-	-	0.008
215.293	16.706	> 0.001	19	-0.305	0.087	-	-0.242	-	-	-	-	-	0.082	-0.054	-	-	0.020
215.348	16.761	> 0.001	28	-0.287	0.221	-	-0.209	-	-0.116	-	0.001	-	-	0.053	-	-0.011	-
215.380	16.794	> 0.001	25	-0.171	0.138	-	-0.182	-0.124	-	-	-	0.108	-	0.090	-0.134	-	-0.039
215.508	16.921	> 0.001	27	-0.357	0.100	-	-0.246	-	-	-	0.101	-	0.084	0.019	0.037	-0.002	-
215.635	17.048	> 0.001	25	-0.187	0.202	-	-0.128	-0.027	-	-	-0.030	-	-0.051	0.048	-	-	-0.018
215.658	17.072	> 0.001	26	-0.355	0.098	-	-0.293	-	-	-	-	-	0.103	-0.019	-	-0.005	-
215.952	17.365	> 0.001	23	-0.274	0.174	-	-0.176	-	-	-	0.027	-	-	-0.003	-0.047	-	0.003
216.023	17.436	> 0.001	21	-0.323	-	-	-0.291	-0.046	-	-	-	-	0.135	-0.003	-	-	-0.032
216.147	17.561	> 0.001	22	-0.268	0.163	-	-0.201	-	-	-	-	0.074	-	-0.012	-0.162	-	0.014
216.160	17.573	> 0.001	24	-0.271	0.022	-	-0.271	-0.083	-	-	-	0.107	0.107	0.026	-	-	-0.034
216.170	17.584	> 0.001	25	-0.305	0.192	-	-0.290	-0.073	-	-	-	-	0.125	0.071	-0.102	-	-0.029
216.382	17.795	> 0.001	28	-0.260	0.119	-	-0.206	-0.077	-	-	0.031	0.206	-	0.086	-0.075	-	-0.038
216.386	17.800	> 0.001	28	-0.335	0.083	-	-0.269	-0.074	-0.098	-	0.081	0.173	0.086	0.089	-	-	-0.059
216.421	17.835	> 0.001	18	-0.380	-	-	-0.304	-	-	-	-	-	0.144	-0.063	-	-	0.010
216.563	17.977	> 0.001	29	-0.387	0.131	-	-0.272	-	-	0.024	0.078	-	0.085	-0.002	-0.006	0.027	-
216.596	18.010	> 0.001	26	-0.258	0.238	-	-0.206	-0.042	-0.128	-	-0.017	-	0.027	0.068	-	-	-0.018
216.658	18.072	> 0.001	29	-0.269	0.200	-	-0.176	-	-	-	-0.022	-	-0.023	0.047	-	-0.018	-
216.659	18.072	> 0.001	29	-0.260	0.133	-	-0.213	-	-	-	-	0.082	-	0.017	-0.105	-0.012	-
216.667	18.081	> 0.001	32	-0.423	0.089	-	-0.294	-	-0.076	-	0.074	0.159	0.075	0.032	-	0.009	-
216.759	18.173	> 0.001	28	-0.393	0.029	-	-0.329	-	-	-	-	0.093	0.127	-0.046	-	0.009	-
216.830	18.244	> 0.001	22	-0.390	0.191	-	-0.329	-	-	-	-	-	0.154	-0.014	-0.123	-	0.016
216.960	18.374	> 0.001	27	-0.407	0.130	-	-0.296	-	-	-	0.022	0.234	0.083	0.006	-0.128	-	-0.001
217.172	18.585	> 0.001	13	-	-	-	-0.055	-	-	-	-	-	-	-0.073	-	-	0.034
217.194	18.608	> 0.001	32	-0.351	0.079	-	-0.237	-	-	-	0.066	0.212	-	0.027	-0.035	0.017	-
217.242	18.655	> 0.001	29	-0.395	0.176	-	-0.343	-	-	-	-	-	0.149	0.038	-0.077	-0.026	-
217.297	18.711	> 0.001	21	-0.352	0.033	-	-0.290	-	-	-	-	0.079	0.121	-0.060	-	-	0.013
217.302	18.716	> 0.001	26	-0.235	0.183	-	-0.177	-0.045	-	-	0.026	-	-	0.068	-0.029	-	-0.027
217.311	18.724	> 0.001	25	-0.418	-	-	-0.348	-	-	-	-	-	0.160	-0.053	-	0.004	-
217.606	19.020	> 0.001	16	-	0.071	-	-0.060	-	-	-	-	0.043	-	-0.065	-	-	0.036
217.627	19.041	> 0.001	17	-	0.151	-	-0.066	-	-0.138	-	-	0.038	-	-0.039	-	-	0.034
217.743	19.156	> 0.001	30	-0.333	0.233	-	-0.247	-	-0.119	-	-0.010	-	0.048	0.072	-	-0.030	-
217.820	19.234	> 0.001	27	-0.353	0.133	-	-0.370	-0.117	-	-	-	0.147	0.188	0.085	-0.162	-	-0.033
218.279	19.693	> 0.001	17	-	0.145	-	-0.068	-	-	-	-	-	-	-0.042	-0.034	-	0.037
218.421	19.834	> 0.001	30	-0.316	0.146	-	-0.205	-	-	-	0.060	-	-	0.015	0.007	0.021	-
218.536	19.949	> 0.001	24	-0.471	0.155	-	-0.415	-	-	-	-	0.119	0.216	-0.016	-0.190	-	0.020
218.773	20.187	> 0.001	30	-0.299	0.143	-	-0.245	-0.079	-	-	0.009	0.228	0.042	0.088	-0.109	-	-0.040
218.943	20.357	> 0.001	31	-0.439	0.131	-	-0.401	-	-	-	-	0.122	0.191	0.044	-0.138	-0.038	-
219.155	20.569	> 0.001	25	-0.337	0.194	-	-0.241	-	-	-	0.013	-	0.072	-0.004	-0.071	-	0.002

219.288	20.701	> 0.001	34	-0.386	0.115	-	-0.278	-	-	-	0.033	0.234	0.048	0.068	-0.078	-0.021	-
220.446	21.860	> 0.001	28	-0.274	0.208	-	-0.215	-0.042	-	-	0.008	-	0.043	0.068	-0.053	-	-0.027
221.325	22.739	> 0.001	32	-0.386	0.175	-	-0.276	-	-	-	0.039	-	0.079	0.038	-0.023	0.000	-
221.562	22.976	> 0.001	19	-	0.146	-	-0.064	-	-	-	-	0.027	-	-0.041	-0.071	-	0.031
262.530	63.944	> 0.001	12	-0.071	0.126	-	-	-0.083	-	-	-	-	-	0.054	-	-	-
263.595	65.008	> 0.001	13	-0.067	0.184	-	-	-0.093	-0.103	-	-	-	-	0.081	-	-	-
263.682	65.095	> 0.001	9	-0.123	0.108	-	-	-	-	-	-	-	-	-0.018	-	-	-
264.337	65.751	> 0.001	3	-	0.123	-	-	-	-	-	-	-	-	-	-	-	-
264.385	65.798	> 0.001	6	-	0.121	-	-	-	-	-	-	-	-	-0.004	-	-	-
264.408	65.822	> 0.001	6	-0.112	0.115	-	-	-	-	-	-	-	-	-	-	-	-
264.497	65.911	> 0.001	15	-0.093	0.218	-	-	-0.068	-	-	-0.044	-	-	0.079	-	-	-
264.500	65.914	> 0.001	9	-0.121	0.197	-	-	-	-	-	-0.041	-	-	-	-	-	-
264.585	65.999	> 0.001	12	-0.130	0.189	-	-	-	-	-	-0.042	-	-	0.005	-	-	-
264.901	66.315	> 0.001	7	-	0.187	-	-	-	-0.125	-	-	-	-	0.018	-	-	-
264.918	66.331	> 0.001	10	-0.122	0.157	-	-	-	-0.092	-	-	-	-	-0.001	-	-	-
265.040	66.454	> 0.001	10	-0.058	0.124	0.048	-	-0.087	-	-	-	-	-	-	-	-	-
265.280	66.694	> 0.001	4	-	0.119	-0.040	-	-	-	-	-	-	-	-	-	-	-
265.581	66.994	> 0.001	5	-	0.187	-0.016	-	-	-0.133	-	-	-	-	-	-	-	-
265.732	67.146	> 0.001	11	-0.062	0.187	0.074	-	-0.091	-0.119	-	-	-	-	-	-	-	-
266.010	67.424	> 0.001	17	-0.094	0.125	-	-	-	-	-	-	-	-	0.028	-	-0.055	-
266.056	67.470	> 0.001	7	-0.109	0.113	-0.024	-	-	-	-	-	-	-	-	-	-	-
266.063	67.476	> 0.001	8	-0.135	-	-	-	-	-	-	-	-	-	-0.037	-	-	-
266.149	67.563	> 0.001	11	-0.080	-	-	-	-0.066	-	-	-	-	-	0.009	-	-	-
266.491	67.905	> 0.001	16	-0.094	0.215	-	-	-0.067	0.010	-	-0.045	-	-	0.077	-	-	-
266.494	67.908	> 0.001	10	-0.121	0.196	-0.003	-	-	-	-	-0.041	-	-	-	-	-	-
266.570	67.984	> 0.001	13	-0.130	0.184	-	-	-	0.015	-	-0.044	-	-	0.003	-	-	-
266.676	68.090	> 0.001	8	-0.111	0.176	-0.003	-	-	-0.122	-	-	-	-	-	-	-	-
266.896	68.309	> 0.001	13	-0.088	0.215	0.065	-	-0.065	-	-	-0.045	-	-	-	-	-	-
267.128	68.542	> 0.001	18	-0.084	0.182	-	-	-	-0.100	-	-	-	-	0.061	-	-0.070	-
267.144	68.558	> 0.001	15	-0.070	0.206	-	-	-0.091	-	-	-	-	-	0.091	-0.133	-	-
267.519	68.933	> 0.001	5	-0.119	-	-	-	-	-	-	-	-	-	-	-	-	-
267.927	69.341	> 0.001	5	-	-	-	-	-	-	-	-	-	-	-0.024	-	-	-
267.941	69.354	> 0.001	20	-0.107	0.218	-	-	-	-	-	-0.032	-	-	0.074	-	-0.066	-
268.124	69.538	> 0.001	2	-	-	-	-	-	-	-	-	-	-	-	-	-	-
268.431	69.845	> 0.001	9	-	0.206	-	-	-	-	-	-	-	-	0.028	-0.147	-	-
268.463	69.876	> 0.001	12	-0.123	0.179	-	-	-	-	-	-	-	-	0.010	-0.126	-	-
268.480	69.893	> 0.001	11	-0.121	0.200	-0.001	-	-	-0.015	-	-0.039	-	-	-	-	-	-
268.657	70.070	> 0.001	9	-0.049	-	0.024	-	-0.087	-	-	-	-	-	-	-	-	-
268.664	70.078	> 0.001	3	-	-	-0.048	-	-	-	-	-	-	-	-	-	-	-
268.889	70.303	> 0.001	14	-0.088	0.218	0.067	-	-0.065	-0.010	-	-0.043	-	-	-	-	-	-
268.960	70.374	> 0.001	6	-0.115	-	-0.030	-	-	-	-	-	-	-	-	-	-	-
269.591	71.004	> 0.001	16	-0.117	-	-	-	-	-	-	-	-	-	-0.034	-	-0.019	-
269.918	71.332	> 0.001	18	-0.085	0.260	-	-	-0.072	-	-	-0.075	-	-	0.092	-0.064	-	-
269.929	71.343	> 0.001	21	-0.108	0.213	-	-	-	0.014	-	-0.033	-	-	0.071	-	-0.064	-
270.081	71.495	> 0.001	15	-0.126	0.224	-	-	-	-	-	-0.070	-	-	0.015	-0.056	-	-
270.382	71.795	> 0.001	20	-0.079	0.208	-	-	-	-	-	-	-	-	0.078	-0.147	-0.078	-
273.128	74.542	> 0.001	23	-0.075	0.276	-	-	-	-	-	-0.079	-	-	0.111	-0.088	-0.098	-

Table S1:

Statistical summary of the models linking the duration of sniffing responses (sec.) to a global model that included the variables; ‘age’ of the donor (levels: yearling, adult); ‘sex’ of the donor (levels: male, female); ‘reproductive status of the female donor’ (levels: oestrous, non-oestrous); and ‘reproductive status of the male donor’ (levels: descended, fully descended), as well as interaction terms (n = 351). Trial ID was included as a random effect in these models. This table is the basis of the model averaging, for which results are presented in Table 2a of the main text.

The support for each model, based on Akaike criterion, is presented in the first three columns. The fourth column presents the degrees of freedom associated with each model. Subsequent columns present coefficient estimates of the parameters included in each model.
