# Supplementary material for: Will Trespassers Be Prosecuted or Assessed According to Their Merits? A Consilient Interpretation of Territoriality in a Group-Living Carnivore, the European Badger (Meles meles)
Source: PLoS One. 2015 Jul 6;10(7):e0132432. doi: 10.1371/journal.pone.0132432 (PMC4493095; doi:10.1371/journal.pone.0132432)
Supplement: S2 Table — The global model included the variables; ‘age’ of the donor (levels: yearling, adult); ‘sex’ of the donor (levels: male, female); ‘reproductive status of the female donor’ (levels: oestrous, non-oestrous); and ‘reproductive status of the male donor’ (levels: descended, fully descended), as well as interaction terms (n = 351). Trial ID was included as a random effect in these models. This table is the basis of the model averaging, for which results are presented in Table 2 of the main text. The support for each model, based on Akaike criterion, is presented in the first three columns. The fourth column presents the degrees of freedom associated with each model. Subsequent columns present coefficient estimates of the parameters included in each model. (PDF) [file pone.0132432.s002.pdf]

Table S2

AIC	ΔAIC	*w*	d.f.	Loc.	Don. Age	Don. Rep.	Fam.	Loc.*Don. Age	Loc.*Don. Rep.	Don. Sex	Loc.*Don. Sex	Don. Age*Don. Sex	Fam.*Loc.	Fam.*Don. Age	Fam.*Don. Sex	Don. Age*Don. Rep.	Fam.*Don. Rep.
603.970	0	0.023	22	0.087	0.275	0.297	0.083	0.111	-0.321	-	-	-	-	-	-	-	-
603.986	0.017	0.022	20	0.193	0.306	0.325	0.081	-	-0.397	-	-	-0.260	-	-	-	-	-
604.142	0.172	0.021	19	0.190	0.161	0.241	0.105	-	-0.358	-	-	-	-	-	-	-	-
604.573	0.603	0.017	24	0.829	0.347	0.346	0.771	0.036	-0.366	-	-	-	-0.743	-	-	-	-
605.445	1.475	0.011	18	0.125	-	0.161	0.069	-	-0.310	-	-	-	-	-	-	-	-
605.926	1.957	0.008	21	0.715	0.175	0.259	0.615	-	-0.370	-	-	-	-0.551	-	-	-	-
605.936	1.966	0.008	23	0.089	0.288	0.305	0.081	0.114	-0.325	-	-	-0.039	-	-	-	-	-
606.180	2.210	0.007	22	0.642	0.304	0.333	0.517	-	-0.403	-	-	-0.236	-0.469	-	-	-	-
606.281	2.311	0.007	24	0.000	0.101	0.262	0.023	0.233	-0.282	-	-	-	-	0.210	-	-	-
606.459	2.489	0.006	25	0.875	0.328	0.334	0.822	0.025	-0.361	-	-	0.075	-0.792	-	-	-	-
607.177	3.207	0.005	26	0.786	0.349	0.281	0.779	0.014	-0.301	-	-	-	-0.771	0.030	-	-	-
607.319	3.349	0.004	21	0.065	0.171	0.106	-0.018	-	-0.224	-	-	-	-	-	0.206	-	-
607.351	3.381	0.004	24	-0.017	0.282	0.180	-0.022	0.108	-0.207	-	-	-	-	-	0.177	-	-
607.472	3.502	0.004	22	0.093	0.304	0.211	-0.014	-	-0.287	-	-	-0.243	-	-	0.162	-	-
607.645	3.675	0.004	22	0.187	0.256	0.290	0.081	-	-0.376	-	-	-	-	-	-	-0.086	-
607.766	3.796	0.003	22	0.187	0.260	0.313	0.064	-	-0.394	-	-	-0.257	-	0.063	-	-	-
607.770	3.800	0.003	20	0.527	-	0.171	0.455	-	-0.316	-	-	-	-0.422	-	-	-	-
607.859	3.889	0.003	21	0.185	0.109	0.232	0.084	-	-0.359	-	-	-	-	0.075	-	-	-
608.278	4.308	0.003	26	0.706	0.343	0.263	0.655	0.042	-0.284	-	-	-	-0.692	-	0.110	-	-
608.279	4.309	0.003	25	-0.001	0.098	0.260	0.023	0.233	-0.281	-	-	0.009	-	0.210	-	-	-
608.770	4.800	0.002	27	0.896	0.337	0.247	0.906	-0.029	-0.280	-	-	0.147	-0.892	0.010	-	-	-
608.976	5.007	0.002	20	0.028	-	0.057	-0.022	-	-0.207	-	-	-	-	-	0.147	-	-
609.209	5.239	0.002	24	0.106	0.162	0.208	0.002	-	-0.366	-	-	-	-	-	-	-	-0.069
609.344	5.374	0.002	25	-0.016	0.288	0.185	-0.021	0.109	-0.210	-	-	-0.018	-	-	0.174	-	-
609.345	5.375	0.002	23	0.546	0.180	0.142	0.450	-	-0.253	-	-	-	-0.484	-	0.166	-	-
609.387	5.417	0.002	25	0.041	0.193	0.245	0.080	0.187	-0.273	-	-	-	-	-	-	0.106	-
609.428	5.459	0.001	24	0.640	0.229	0.280	0.520	-	-0.372	-	-	-	-0.474	-	-	-0.034	-
609.523	5.553	0.001	27	-0.002	0.271	0.275	-0.011	0.121	-0.329	-	-	-	-	-	-	-	-0.100
609.721	5.752	0.001	23	0.685	0.160	0.231	0.602	-	-0.345	-	-	-	-0.545	0.018	-	-	-
609.757	5.788	0.001	24	0.507	0.302	0.233	0.388	-	-0.305	-	-	-0.226	-0.419	-	0.134	-	-
609.837	5.868	0.001	25	0.114	0.286	0.296	-0.003	-	-0.405	-	-	-0.215	-	-	-	-	-0.102
609.897	5.928	0.001	27	0.756	0.213	0.262	0.755	0.121	-0.296	-	-	-	-0.725	-	-	0.179	-
609.995	6.025	0.001	24	0.615	0.291	0.304	0.508	-	-0.378	-	-	-0.235	-0.466	0.014	-	-	-
610.006	6.037	0.001	26	-0.065	0.137	0.180	-0.041	0.207	-0.203	-	-	-	-	0.177	0.122	-	-
610.151	6.181	0.001	27	0.751	0.323	0.247	0.704	0.030	-0.275	-	-	0.080	-0.742	-	0.116	-	-
610.363	6.393	0.001	23	0.096	-	0.163	0.009	-	-0.363	-	-	-	-	-	-	-	-0.096
610.905	6.935	0.001	13	0.213	0.272	-	0.043	-	-	0.157	-0.203	-0.259	-	-	-	-	-
610.977	7.007	0.001	28	0.702	0.365	0.211	0.699	0.005	-0.233	-	-	-	-0.742	0.011	0.095	-	-
611.131	7.162	0.001	12	0.236	0.135	-	0.057	-	-	0.100	-0.195	-	-	-	-	-	-
611.165	7.196	0.001	23	0.062	0.140	0.100	-0.022	-	-0.224	-	-	-	-	0.043	0.194	-	-
611.174	7.204	0.001	24	0.095	0.256	0.185	-0.010	-	-0.276	-	-	-	-	-	0.158	-0.076	-
611.320	7.350	0.001	22	0.395	-	0.078	0.331	-	-0.223	-	-	-	-0.371	-	0.121	-	-
611.339	7.369	0.001	24	0.089	0.275	0.204	-0.019	-	-0.286	-	-	-0.242	-	0.039	0.151	-	-
611.350	7.380	0.001	29	0.666	0.331	0.335	0.617	0.056	-0.378	-	-	-	-0.641	-	-	-	-0.126
611.516	7.546	0.001	11	0.225	-	-	0.037	-	-	0.073	-0.194	-	-	-	-	-	-
611.520	7.550	0.001	28	-0.003	0.267	0.271	-0.011	0.120	-0.327	-	-	0.011	-	-	-	-	-0.099

611.594	7.625	> 0.001	24	0.189	0.236	0.295	0.070	-	-0.385	-	-	-	-	0.042	-	-0.096	-
611.629	7.659	> 0.001	27	-0.022	0.003	0.258	0.003	0.300	-0.282	-	-	-	-	0.295	-	0.115	-
611.911	7.941	> 0.001	26	0.536	0.167	0.225	0.419	-	-0.377	-	-	-	-0.427	-	-	-	-0.068
611.983	8.014	> 0.001	14	0.864	0.151	-	0.675	-	-	0.131	-0.223	-	-0.661	-	-	-	-
611.996	8.026	> 0.001	27	-0.069	0.130	0.172	-0.042	0.205	-0.197	-	-	0.023	-	0.177	0.125	-	-
612.338	8.369	> 0.001	15	0.755	0.267	-	0.573	-	-	0.177	-0.226	-0.225	-0.567	-	-	-	-
612.512	8.542	> 0.001	29	-0.047	0.169	0.258	-0.026	0.187	-0.309	-	-	-	-	0.130	-	-	-0.124
612.541	8.571	> 0.001	29	0.807	0.356	0.168	0.822	-0.044	-0.204	-	-	0.153	-0.866	-0.013	0.107	-	-
612.657	8.687	> 0.001	29	0.889	0.340	0.260	0.890	-0.040	-0.293	-	-	-	-0.885	0.047	-	0.098	-
612.766	8.796	> 0.001	27	0.493	0.279	0.302	0.364	-	-0.410	-	-	-0.198	-0.376	-	-	-	-0.096
612.851	8.882	> 0.001	27	-0.051	0.196	0.137	-0.015	0.180	-0.171	-	-	-	-	-	0.166	0.120	-
612.902	8.933	> 0.001	13	0.736	-	-	0.535	-	-	0.098	-0.217	-	-0.539	-	-	-	-
613.004	9.034	> 0.001	17	0.930	0.295	-	0.808	0.007	-	0.164	-0.179	-	-0.816	-	-	-	-
613.088	9.118	> 0.001	26	0.083	0.165	0.182	0.004	-	-0.342	-	-	-	-	-0.009	-	-	-0.066
613.114	9.144	> 0.001	26	0.514	0.230	0.191	0.396	-	-0.285	-	-	-	-0.424	-	0.130	-0.031	-
613.162	9.192	> 0.001	30	0.718	0.303	0.315	0.675	0.045	-0.368	-	-	0.097	-0.699	-	-	-	-0.120
613.166	9.196	> 0.001	25	0.429	-	0.166	0.326	-	-0.363	-	-	-	-0.329	-	-	-	-0.079
613.170	9.200	> 0.001	25	0.524	0.180	0.112	0.455	-	-0.224	-	-	-	-0.490	-0.005	0.166	-	-
613.202	9.232	> 0.001	15	0.135	0.229	-	0.043	0.078	-	0.117	-0.134	-	-	-	-	-	-
613.336	9.366	> 0.001	26	0.656	0.264	0.259	0.569	-	-0.345	-	-	-	-0.512	-0.063	-	-0.032	-
613.539	9.569	> 0.001	27	0.111	0.246	0.275	-0.001	-	-0.395	-	-	-	-	-	-	-0.085	-0.111
613.596	9.626	> 0.001	26	0.485	0.301	0.204	0.393	-	-0.277	-	-	-0.225	-0.425	-0.005	0.133	-	-
613.645	9.675	> 0.001	29	0.642	0.213	0.182	0.643	0.121	-0.219	-	-	-	-0.678	-	0.116	0.182	-
613.749	9.779	> 0.001	27	0.097	0.283	0.275	-0.003	-	-0.386	-	-	-0.213	-	-0.001	-	-	-0.101
613.977	10.007	> 0.001	15	0.266	0.294	0.175	0.070	-	-	-	-0.235	-0.268	-	-	-	-	-
614.168	10.198	> 0.001	14	0.278	0.147	0.105	0.097	-	-	-	-0.206	-	-	-	-	-	-
614.215	10.246	> 0.001	15	0.222	0.210	-	0.022	-	-	0.166	-0.227	-0.263	-	0.099	-	-	-
614.241	10.272	> 0.001	31	0.668	0.362	0.284	0.665	0.012	-0.327	-	-	-	-0.693	-0.002	-	-	-0.128
614.343	10.373	> 0.001	15	0.127	0.269	-	-0.036	-	-	0.023	-0.096	-0.249	-	-	0.123	-	-
614.380	10.410	> 0.001	14	0.128	0.139	-	-0.043	-	-	-0.063	-0.065	-	-	-	0.155	-	-
614.456	10.486	> 0.001	14	0.250	0.054	-	0.030	-	-	0.111	-0.225	-	-	0.125	-	-	-
614.460	10.491	> 0.001	30	-0.054	0.153	0.241	-0.026	0.183	-0.297	-	-	0.050	-	0.128	-	-	-0.118
614.845	10.875	> 0.001	13	0.246	-	0.053	0.072	-	-	-	-0.187	-	-	-	-	-	-
614.885	10.915	> 0.001	13	0.130	-	-	-0.051	-	-	-0.070	-0.079	-	-	-	0.134	-	-
614.934	10.964	> 0.001	17	0.090	0.046	-	-0.013	0.200	-	0.134	-0.149	-	-	0.245	-	-	-
614.940	10.970	> 0.001	19	0.980	0.331	-	0.845	-0.051	-	0.178	-0.192	-	-0.869	0.037	-	-	-
614.943	10.973	> 0.001	16	0.131	0.259	-	0.040	0.089	-	0.135	-0.140	-0.108	-	-	-	-	-
614.995	11.025	> 0.001	18	0.946	0.290	-	0.823	0.003	-	0.161	-0.178	0.022	-0.832	-	-	-	-
615.120	11.151	> 0.001	30	-0.037	0.180	0.226	-0.006	0.189	-0.287	-	-	-	-	-	-	0.113	-0.106
615.163	11.193	> 0.001	26	0.092	0.253	0.184	-0.012	-	-0.276	-	-	-	-	0.012	0.155	-0.083	-
615.184	11.215	> 0.001	16	0.874	0.163	0.135	0.673	-	-	-	-0.240	-	-0.626	-	-	-	-
615.293	11.323	> 0.001	16	0.734	0.151	-	0.553	-	-	0.002	-0.116	-	-0.613	-	0.115	-	-
615.426	11.457	> 0.001	17	0.786	0.293	0.195	0.572	-	-	-	-0.262	-0.242	-0.543	-	-	-	-
615.436	11.466	> 0.001	29	-0.076	0.041	0.187	-0.049	0.266	-0.214	-	-	-	-	0.264	0.104	0.109	-
615.521	11.551	> 0.001	16	0.845	0.115	-	0.639	-	-	0.135	-0.235	-	-0.637	0.058	-	-	-
615.676	11.706	> 0.001	28	0.512	0.192	0.181	0.439	-	-0.332	-	-	-	-0.442	-0.045	-	-	-0.057
615.704	11.734	> 0.001	17	0.648	0.264	-	0.474	-	-	0.068	-0.136	-0.222	-0.529	-	0.093	-	-
615.713	11.743	> 0.001	32	0.779	0.347	0.235	0.795	-0.036	-0.297	-	-	0.169	-0.822	-0.029	-	-	-0.115
615.723	11.753	> 0.001	17	0.737	0.247	-	0.545	-	-	0.182	-0.236	-0.237	-0.548	0.042	-	-	-
616.061	12.092	> 0.001	19	0.987	0.324	0.188	0.824	-0.009	-	-	-0.220	-	-0.811	-	-	-	-

616.184	12.214	> 0.001	15	0.618	-	-	0.425	-	-	-0.021	-0.118	-	-0.496	-	0.103	-	-
616.244	12.275	> 0.001	17	0.191	0.250	0.138	0.076	0.066	-	-	-0.161	-	-	-	-	-	-
616.268	12.298	> 0.001	29	0.498	0.224	0.269	0.373	-	-0.392	-	-	-	-0.385	-	-	-0.047	-0.101
616.404	12.434	> 0.001	19	0.814	0.291	-	0.701	0.012	-	0.052	-0.085	-	-0.772	-	0.097	-	-
616.410	12.440	> 0.001	15	0.718	-	0.075	0.525	-	-	-	-0.214	-	-0.496	-	-	-	-
616.424	12.454	> 0.001	31	0.824	0.377	0.189	0.827	-0.071	-0.223	-	-	-	-0.877	0.020	0.101	0.086	-
616.480	12.510	> 0.001	17	0.032	0.232	-	-0.053	0.075	-	-0.040	-0.008	-	-	-	0.149	-	-
616.590	12.621	> 0.001	29	0.472	0.295	0.263	0.381	-	-0.371	-	-	-0.193	-0.390	-0.034	-	-	-0.087
616.745	12.775	> 0.001	16	0.133	0.163	-0.031	-0.058	-	-	-	-0.021	-	-	-	0.257	-	-
616.797	12.828	> 0.001	32	0.627	0.206	0.257	0.625	0.127	-0.317	-	-	-	-0.646	-	-	0.168	-0.114
616.823	12.853	> 0.001	17	0.136	0.299	0.047	-0.067	-	-	-	-0.067	-0.252	-	-	0.228	-	-
616.832	12.862	> 0.001	20	1.048	0.325	-	0.909	-0.074	-	0.169	-0.190	0.075	-0.933	0.030	-	-	-
616.845	12.876	> 0.001	18	0.089	0.069	-	-0.014	0.204	-	0.145	-0.153	-0.064	-	0.239	-	-	-
616.956	12.986	> 0.001	28	0.523	0.277	0.154	0.449	-	-0.240	-	-	-	-0.469	-0.087	0.146	-0.023	-
617.452	13.482	> 0.001	17	0.272	0.223	0.173	0.046	-	-	-	-0.256	-0.265	-	0.104	-	-	-
617.455	13.486	> 0.001	29	0.086	0.280	0.249	0.006	-	-0.365	-	-	-	-	-0.062	-	-0.085	-0.103
617.591	13.622	> 0.001	16	0.288	0.068	0.108	0.069	-	-	-	-0.233	-	-	0.119	-	-	-
617.593	13.624	> 0.001	17	0.279	0.248	0.158	0.074	-	-	-	-0.240	-	-	-	-	-0.093	-
617.635	13.665	> 0.001	20	0.051	0.141	0.173	-0.023	-	-	-	-0.030	-	-	-	-	-	-0.001
617.804	13.835	> 0.001	25	0.791	0.288	0.316	0.793	0.094	-	-	-0.065	-	-0.863	-	-	-	-0.071
617.820	13.850	> 0.001	17	0.146	0.220	-	-0.042	-	-	0.051	-0.132	-0.255	-	0.081	0.104	-	-
617.906	13.936	> 0.001	22	0.732	0.148	0.228	0.639	-	-	-	-0.112	-	-0.684	-	-	-	-0.026
617.911	13.941	> 0.001	16	0.154	0.072	-	-0.051	-	-	-0.031	-0.107	-	-	0.102	0.133	-	-
617.926	13.956	> 0.001	19	0.054	-	0.156	-0.011	-	-	-	-0.054	-	-	-	-	-	-0.034
617.942	13.973	> 0.001	15	0.129	-	-0.058	-0.048	-	-	-	-0.041	-	-	-	0.194	-	-
617.963	13.994	> 0.001	21	0.049	0.280	0.244	-0.034	-	-	-	-0.069	-0.242	-	-	-	-	-0.024
617.990	14.020	> 0.001	18	0.189	0.285	0.159	0.069	0.076	-	-	-0.174	-0.110	-	-	-	-	-
618.049	14.079	> 0.001	18	0.664	0.171	0.012	0.458	-	-	-	-0.073	-	-0.537	-	0.214	-	-
618.060	14.091	> 0.001	20	0.992	0.322	0.186	0.829	-0.010	-	-	-0.219	0.007	-0.816	-	-	-	-
618.242	14.273	> 0.001	19	0.136	0.059	0.137	0.016	0.197	-	-	-0.170	-	-	0.244	-	-	-
618.243	14.273	> 0.001	32	-0.061	0.075	0.247	-0.033	0.241	-0.301	-	-	-	-	0.201	-	0.100	-0.124
618.296	14.326	> 0.001	18	0.035	0.258	-	-0.049	0.085	-	-0.013	-0.022	-0.092	-	-	0.138	-	-
618.389	14.420	> 0.001	21	0.638	-	0.201	0.553	-	-	-	-0.124	-	-0.589	-	-	-	-0.048
618.393	14.423	> 0.001	20	0.830	0.286	-	0.716	0.007	-	0.047	-0.084	0.023	-0.789	-	0.099	-	-
618.399	14.429	> 0.001	19	0.594	0.295	0.079	0.377	-	-	-	-0.107	-0.234	-0.465	-	0.195	-	-
618.430	14.460	> 0.001	21	0.987	0.329	0.166	0.832	-0.042	-	-	-0.213	-	-0.835	0.047	-	-	-
618.532	14.562	> 0.001	19	0.806	0.220	0.171	0.573	-	-	-	-0.271	-	-0.543	-	-	-0.036	-
618.546	14.577	> 0.001	21	0.895	0.342	-	0.767	-0.057	-	0.082	-0.112	-	-0.842	0.020	0.084	-	-
618.561	14.591	> 0.001	23	-0.093	0.213	0.238	-0.039	0.178	-	-	0.054	-	-	-	-	-	-0.038
618.604	14.634	> 0.001	19	0.027	0.072	-	-0.072	0.181	-	0.028	-0.064	-	-	0.218	0.099	-	-
618.668	14.698	> 0.001	23	0.673	0.267	0.285	0.572	-	-	-	-0.138	-0.209	-0.626	-	-	-	-0.041
618.773	14.803	> 0.001	19	0.050	0.272	0.001	-0.081	0.052	-	-	0.022	-	-	-	0.260	-	-
618.825	14.856	> 0.001	18	0.846	0.123	0.126	0.635	-	-	-	-0.246	-	-0.599	0.058	-	-	-
618.944	14.974	> 0.001	18	0.731	0.125	-	0.538	-	-	0.015	-0.133	-	-0.601	0.041	0.106	-	-
619.016	15.046	> 0.001	21	0.787	0.329	0.070	0.618	-0.009	-	-	-0.063	-	-0.726	-	0.202	-	-
619.036	15.067	> 0.001	19	0.758	0.259	0.184	0.538	-	-	-	-0.266	-0.244	-0.519	0.050	-	-	-
619.204	15.234	> 0.001	19	0.643	0.253	-	0.463	-	-	0.082	-0.150	-0.234	-0.519	0.029	0.085	-	-
619.535	15.565	> 0.001	17	0.547	-	-0.026	0.354	-	-	-	-0.080	-	-0.425	-	0.163	-	-
619.620	15.650	> 0.001	34	0.846	0.414	0.256	0.847	-0.102	-0.315	-	-	-	-0.878	-0.026	-	0.065	-0.118
619.715	15.745	> 0.001	26	0.829	0.269	0.306	0.835	0.087	-	-	-0.057	0.068	-0.905	-	-	-	-0.069

619.926	15.956	> 0.001	31	0.501	0.292	0.217	0.441	-	-0.332	-	-	-	-0.437	-0.135	-	-0.036	-0.082
620.058	16.089	> 0.001	27	0.782	0.243	0.334	0.806	0.107	-	-	-0.089	-	-0.866	0.086	-	-	-0.112
620.155	16.185	> 0.001	20	0.137	0.080	0.151	0.013	0.203	-	-	-0.178	-0.065	-	0.241	-	-	-
620.342	16.372	> 0.001	22	1.046	0.324	0.154	0.893	-0.064	-	-	-0.207	0.070	-0.893	0.038	-	-	-
620.426	16.456	> 0.001	25	-0.111	-0.013	0.291	-0.053	0.337	-	-	-0.020	-	-	0.270	-	-	-0.103
620.434	16.464	> 0.001	22	0.960	0.338	-	0.829	-0.083	-	0.066	-0.106	0.077	-0.907	0.011	0.090	-	-
620.478	16.508	> 0.001	18	0.153	0.106	-0.016	-0.063	-	-	-	-0.057	-	-	0.083	0.232	-	-
620.513	16.543	> 0.001	24	-0.089	0.231	0.249	-0.039	0.179	-	-	0.044	-0.048	-	-	-	-	-0.041
620.536	16.566	> 0.001	20	0.029	0.090	-	-0.069	0.186	-	0.043	-0.071	-0.056	-	0.214	0.093	-	-
620.564	16.595	> 0.001	19	0.153	0.248	0.059	-0.069	-	-	-	-0.098	-0.252	-	0.073	0.205	-	-
620.606	16.636	> 0.001	11	0.023	0.196	-	0.018	0.082	-	-	-	-	-	-	-	-	-
620.621	16.651	> 0.001	19	0.155	0.263	0.043	-0.056	-	-	-	-0.082	-	-	-	0.217	-0.098	-
620.640	16.670	> 0.001	20	0.054	0.296	0.021	-0.080	0.060	-	-	0.006	-0.080	-	-	0.249	-	-
620.738	16.768	> 0.001	7	0.066	-	-	0.019	-	-	-	-	-	-	-	-	-	-
621.011	17.041	> 0.001	22	0.797	0.325	0.067	0.628	-0.012	-	-	-0.061	0.016	-0.736	-	0.203	-	-
621.040	17.071	> 0.001	8	0.089	0.113	-	0.034	-	-	-	-	-	-	-	-	-	-
621.269	17.299	> 0.001	13	0.685	0.240	-	0.673	0.034	-	-	-	-	-0.703	-	-	-	-
621.270	17.301	> 0.001	22	0.064	0.096	0.181	-0.020	-	-	-	-0.058	-	-	0.062	-	-	-0.020
621.345	17.376	> 0.001	19	0.283	0.203	0.166	0.051	-	-	-	-0.259	-	-	0.096	-	-0.115	-
621.410	17.440	> 0.001	22	0.927	0.225	0.157	0.776	0.065	-	-	-0.209	-	-0.757	-	-	0.123	-
621.460	17.490	> 0.001	21	0.040	0.120	0.032	-0.090	0.154	-	-	-0.029	-	-	0.192	0.196	-	-
621.467	17.497	> 0.001	24	0.726	0.145	0.225	0.656	-	-	-	-0.109	-	-0.687	-0.004	-	-	-0.040
621.509	17.539	> 0.001	23	0.072	0.235	0.240	-0.019	-	-	-	-0.087	-	-	-	-	-0.045	-0.035
621.529	17.560	> 0.001	20	0.184	0.213	0.134	0.074	0.126	-	-	-0.167	-	-	-	-	0.035	-
621.543	17.573	> 0.001	8	0.078	-	-	0.022	-	-	-0.074	-	-	-	-	-	-	-
621.551	17.581	> 0.001	10	0.077	0.248	-	0.019	-	-	-0.021	-	-0.277	-	-	-	-	-
621.604	17.634	> 0.001	23	0.064	0.232	0.253	-0.033	-	-	-	-0.099	-0.242	-	0.068	-	-	-0.042
621.721	17.751	> 0.001	28	0.881	0.231	0.311	0.916	0.068	-	-	-0.070	0.137	-0.972	0.065	-	-	-0.109
621.736	17.766	> 0.001	23	0.849	0.363	0.069	0.689	-0.063	-	-	-0.082	-	-0.786	0.014	0.169	-	-
621.836	17.866	> 0.001	21	0.632	0.235	0.073	0.397	-	-	-	-0.134	-	-0.471	-	0.177	-0.048	-
621.839	17.869	> 0.001	25	0.695	0.196	0.279	0.579	-	-	-	-0.164	-	-0.615	-	-	0.017	-0.056
621.871	17.901	> 0.001	20	0.660	0.148	0.012	0.452	-	-	-	-0.086	-	-0.529	0.032	0.199	-	-
622.022	18.052	> 0.001	9	0.099	0.107	-	0.036	-	-	-0.068	-	-	-	-	-	-	-
622.185	18.215	> 0.001	21	0.591	0.277	0.078	0.374	-	-	-	-0.119	-0.237	-0.458	0.027	0.179	-	-
622.259	18.289	> 0.001	25	0.668	0.257	0.283	0.585	-	-	-	-0.139	-0.207	-0.627	0.005	-	-	-0.056
622.389	18.419	> 0.001	12	0.029	0.187	-	0.020	0.081	-	-0.032	-	-	-	-	-	-	-
622.421	18.451	> 0.001	9	0.530	-	-	0.481	-	-	-	-	-	-0.501	-	-	-	-
622.425	18.455	> 0.001	26	-0.110	-0.011	0.293	-0.053	0.338	-	-	-0.022	-0.008	-	0.270	-	-	-0.103
622.440	18.471	> 0.001	10	0.634	0.123	-	0.579	-	-	-	-	-	-0.586	-	-	-	-
622.508	18.538	> 0.001	21	0.805	0.232	0.167	0.581	-	-	-	-0.265	-	-0.549	-0.010	-	-0.047	-
622.528	18.558	> 0.001	13	-0.029	0.018	-	-0.036	0.202	-	-	-	-	-	0.234	-	-	-
622.553	18.583	> 0.001	28	0.745	0.135	0.295	0.782	0.193	-	-	-0.056	-	-0.823	-	-	0.240	-0.090
623.219	19.249	> 0.001	14	0.675	0.235	-	0.661	0.034	-	-0.015	-	-	-0.689	-	-	-	-
623.413	19.444	> 0.001	22	0.043	0.134	0.044	-0.090	0.160	-	-	-0.038	-0.048	-	0.191	0.191	-	-
623.449	19.480	> 0.001	15	0.697	0.252	-	0.680	-0.002	-	-	-	-	-0.727	0.052	-	-	-
623.500	19.531	> 0.001	10	0.503	-	-	0.445	-	-	-0.065	-	-	-0.460	-	-	-	-
623.544	19.574	> 0.001	26	-0.086	0.132	0.245	-0.013	0.250	-	-	0.034	-	-	-	-	0.146	-0.065
623.633	19.663	> 0.001	24	0.910	0.360	0.053	0.752	-0.089	-	-	-0.073	0.076	-0.848	0.002	0.174	-	-
623.674	19.704	> 0.001	22	0.129	0.009	0.148	0.002	0.251	-	-	-0.183	-	-	0.314	-	0.041	-
623.716	19.747	> 0.001	11	0.605	0.117	-	0.543	-	-	-0.058	-	-	-0.546	-	-	-	-

623.760	19.790	> 0.001	12	0.482	0.241	-	0.423	-	-	-0.017	-	-0.250	-0.435	-	-	-	-
623.939	19.969	> 0.001	13	0.022	0.225	-	0.015	0.097	-	-0.015	-	-0.143	-	-	-	-	-
624.271	20.301	> 0.001	24	1.043	0.332	0.156	0.889	-0.077	-	-	-0.209	-	-0.892	0.063	-	0.042	-
624.322	20.352	> 0.001	12	0.068	0.253	-	-0.014	-	-	-0.057	-	-0.281	-	-	0.056	-	-
624.389	20.420	> 0.001	22	0.061	0.245	0.012	-0.068	0.095	-	-	-0.001	-	-	-	0.238	0.036	-
624.410	20.440	> 0.001	14	-0.023	0.016	-	-0.034	0.198	-	-0.024	-	-	-	0.229	-	-	-
624.466	20.496	> 0.001	10	0.068	-	-	-0.021	-	-	-0.120	-	-	-	-	0.069	-	-
624.495	20.525	> 0.001	21	0.164	0.230	0.055	-0.065	-	-	-	-0.105	-	-	0.069	0.204	-0.116	-
624.545	20.575	> 0.001	10	0.094	-	-0.087	0.060	-	-	-	-	-	-	-	-	-	-
624.633	20.663	> 0.001	24	0.762	0.250	0.055	0.602	0.046	-	-	-0.068	-	-0.695	-	0.189	0.112	-
624.737	20.767	> 0.001	28	-0.117	-0.195	0.305	-0.066	0.471	-	-	-0.067	-	-	0.440	-	0.176	-0.112
624.744	20.775	> 0.001	10	0.083	0.075	-	0.021	-	-	-	-	-	-	0.061	-	-	-
624.860	20.891	> 0.001	11	0.089	0.110	-	-0.007	-	-	-0.115	-	-	-	-	0.070	-	-
625.032	21.063	> 0.001	30	0.735	0.088	0.314	0.767	0.181	-	-	-0.089	-	-0.836	0.206	-	0.183	-0.106
625.140	21.170	> 0.001	12	0.071	0.234	-	0.012	-	-	-0.020	-	-0.286	-	0.033	-	-	-
625.190	21.220	> 0.001	11	0.118	0.103	-0.069	0.074	-	-	-	-	-	-	-	-	-	-
625.190	21.220	> 0.001	15	0.645	0.243	-	0.632	0.041	-	-0.011	-	-0.039	-0.660	-	-	-	-
625.240	21.271	> 0.001	12	0.095	0.236	-0.023	0.046	-	-	-	-	-0.255	-	-	-	-	-
625.280	21.311	> 0.001	25	0.086	0.193	0.251	-0.032	-	-	-	-0.122	-	-	0.093	-	-0.071	-0.041
625.434	21.465	> 0.001	16	0.691	0.249	-	0.672	-0.001	-	-0.008	-	-	-0.718	0.052	-	-	-
625.441	21.471	> 0.001	14	0.027	0.192	-	0.015	0.074	-	-0.039	-	-	-	-	0.012	-	-
625.683	21.713	> 0.001	16	0.677	0.238	-	0.663	0.032	-	-0.016	-	-	-0.695	-	0.004	-	-
625.728	21.758	> 0.001	11	0.093	0.072	-	0.024	-	-	-0.068	-	-	-	0.056	-	-	-
625.766	21.796	> 0.001	27	0.693	0.215	0.276	0.596	-	-	-	-0.151	-	-0.626	-0.021	-	-0.001	-0.059
625.813	21.844	> 0.001	23	0.637	0.252	0.068	0.414	-	-	-	-0.125	-	-0.484	-0.023	0.177	-0.054	-
625.878	21.909	> 0.001	12	0.486	-	-	0.396	-	-	-0.107	-	-	-0.453	-	0.064	-	-
625.951	21.982	> 0.001	14	0.465	0.247	-	0.380	-	-	-0.049	-	-0.258	-0.428	-	0.052	-	-
625.960	21.990	> 0.001	14	0.050	0.182	-0.030	0.050	0.079	-	-	-	-	-	-	-	-	-
626.040	22.070	> 0.001	13	0.589	0.118	-	0.496	-	-	-0.099	-	-	-0.540	-	0.063	-	-
626.125	22.155	> 0.001	12	0.638	0.127	-	0.586	-	-	-	-	-	-0.593	-0.003	-	-	-
626.190	22.221	> 0.001	15	-0.026	0.051	-	-0.035	0.205	-	-0.012	-	-0.101	-	0.218	-	-	-
626.674	22.704	> 0.001	12	0.485	-	-0.080	0.448	-	-	-	-	-	-0.427	-	-	-	-
626.921	22.952	> 0.001	15	0.019	0.233	-	0.011	0.093	-	-0.018	-	-0.154	-	-	0.008	-	-
626.949	22.980	> 0.001	24	0.035	0.092	0.045	-0.101	0.180	-	-	-0.043	-	-	0.265	0.189	0.020	-
627.013	23.043	> 0.001	16	0.660	0.230	-0.014	0.651	0.036	-	-	-	-	-0.657	-	-	-	-
627.096	23.126	> 0.001	13	0.583	0.112	-0.061	0.537	-	-	-	-	-	-0.505	-	-	-	-
627.231	23.261	> 0.001	14	0.486	0.261	-	0.432	-	-	-0.015	-	-0.264	-0.444	-0.015	-	-	-
627.391	23.421	> 0.001	13	0.608	0.121	-	0.549	-	-	-0.058	-	-	-0.553	-0.003	-	-	-
627.433	23.463	> 0.001	17	0.698	0.248	-	0.680	-0.004	-	-0.009	-	0.008	-0.725	0.051	-	-	-
627.470	23.500	> 0.001	26	0.943	0.414	0.055	0.782	-0.147	-	-	-0.069	-	-0.887	0.020	0.180	0.013	-
627.519	23.549	> 0.001	14	0.478	0.230	-0.020	0.426	-	-	-	-	-0.231	-0.414	-	-	-	-
627.589	23.619	> 0.001	16	-0.027	0.005	-	-0.028	0.212	-	-0.016	-	-	-	0.238	-0.009	-	-
627.622	23.652	> 0.001	17	0.635	0.249	-	0.622	0.042	-	-0.010	-	-0.056	-0.653	-	0.004	-	-
627.645	23.676	> 0.001	15	0.041	0.217	-0.015	0.041	0.094	-	-	-	-0.122	-	-	-	-	-
627.723	23.753	> 0.001	12	0.085	-	-0.104	0.023	-	-	-	-	-	-	-	0.054	-	-
627.970	24.000	> 0.001	16	-0.004	0.032	-0.039	-0.001	0.175	-	-	-	-	-	0.207	-	-	-
628.009	24.039	> 0.001	14	0.062	0.231	-	-0.025	-	-	-0.057	-	-0.287	-	0.041	0.057	-	-
628.117	24.148	> 0.001	14	0.085	0.247	-0.041	0.000	-	-	-	-	-0.266	-	-	0.067	-	-
628.131	24.162	> 0.001	18	0.681	0.239	-	0.664	0.009	-	-0.008	-	-	-0.713	0.059	0.002	-	-
628.236	24.266	> 0.001	13	0.110	0.108	-0.090	0.028	-	-	-	-	-	-	-	0.071	-	-

628.610	24.641	> 0.001	13	0.083	0.068	-	-0.023	-	-	-0.117	-	-	-	0.066	0.073	-	-
628.737	24.768	> 0.001	14	0.085	0.220	-0.037	0.041	-	-	-	-	-0.259	-	0.027	-	-	-
628.740	24.770	> 0.001	13	0.109	0.074	-0.082	0.066	-	-	-	-	-	-	0.044	-	-	-
628.914	24.944	> 0.001	14	0.102	0.195	-0.039	0.049	-	-	-	-	-	-	-	-	-0.096	-
629.001	25.031	> 0.001	17	0.641	0.235	-0.011	0.632	0.040	-	-	-	-0.026	-0.639	-	-	-	-
629.060	25.090	> 0.001	18	0.690	0.258	-0.034	0.686	-0.020	-	-	-	-	-0.704	0.034	-	-	-
629.150	25.180	> 0.001	16	0.045	0.188	-0.035	0.036	0.071	-	-	-	-	-	-	0.016	-	-
629.311	25.341	> 0.001	17	-0.030	0.043	-	-0.029	0.220	-	-0.002	-	-0.114	-	0.227	-0.010	-	-
629.325	25.355	> 0.001	14	0.475	-	-0.094	0.410	-	-	-	-	-	-0.427	-	0.048	-	-
629.559	25.589	> 0.001	16	0.466	0.261	-	0.387	-	-	-0.046	-	-0.270	-0.433	-0.008	0.051	-	-
629.594	25.624	> 0.001	18	0.666	0.234	-0.015	0.654	0.032	-	-	-	-	-0.674	-	0.008	-	-
629.639	25.669	> 0.001	15	0.572	0.115	-0.078	0.490	-	-	-	-	-	-0.504	-	0.063	-	-
629.816	25.846	> 0.001	15	0.587	0.117	-	0.496	-	-	-0.099	-	-	-0.541	0.005	0.063	-	-
629.827	25.857	> 0.001	16	0.460	0.243	-0.034	0.372	-	-	-	-	-0.248	-0.408	-	0.063	-	-
629.859	25.889	> 0.001	17	-0.007	0.055	-0.029	-0.005	0.183	-	-	-	-0.074	-	0.202	-	-	-
629.940	25.970	> 0.001	16	0.005	-	-0.018	0.019	-	-	-	-	-	-	-	-	-	-0.005
630.129	26.159	> 0.001	19	0.669	0.240	-	0.653	0.013	-	-0.007	-	-0.012	-0.702	0.061	0.002	-	-
630.283	26.313	> 0.001	22	0.694	0.210	0.100	0.813	0.110	-	-	-	-	-0.838	-	-	-	-0.057
630.404	26.435	> 0.001	18	0.526	-	-0.005	0.545	-	-	-	-	-	-0.578	-	-	-	0.013
630.545	26.575	> 0.001	15	0.587	0.119	-0.076	0.552	-	-	-	-	-	-0.517	-0.011	-	-	-
630.754	26.785	> 0.001	17	0.035	0.227	-0.018	0.027	0.087	-	-	-	-0.137	-	-	0.015	-	-
630.763	26.793	> 0.001	16	0.491	0.165	-0.042	0.430	-	-	-	-	-	-0.416	-	-	-0.042	-
630.813	26.843	> 0.001	18	-0.003	0.246	0.045	-0.006	-	-	-	-	-0.269	-	-	-	-	-0.006
630.837	26.867	> 0.001	17	0.025	0.095	-0.010	0.021	-	-	-	-	-	-	-	-	-	0.005
630.867	26.897	> 0.001	16	0.483	0.247	-0.035	0.444	-	-	-	-	-0.239	-0.428	-0.019	-	-	-
631.029	27.059	> 0.001	19	0.728	0.256	-0.040	0.724	-0.034	-	-	-	0.042	-0.740	0.028	-	-	-
631.124	27.154	> 0.001	19	0.615	0.104	0.006	0.620	-	-	-	-	-	-0.652	-	-	-	0.020
631.149	27.179	> 0.001	17	0.041	0.142	-0.037	0.046	0.145	-	-	-	-	-	-	-	0.029	-
631.178	27.208	> 0.001	20	-0.069	0.147	0.074	0.045	0.161	-	-	-	-	-	-	-	-	-0.070
631.275	27.306	> 0.001	18	-0.014	0.015	-0.031	0.004	0.196	-	-	-	-	-	0.221	-0.020	-	-
631.552	27.582	> 0.001	19	0.632	0.245	-0.011	0.619	0.040	-	-	-	-0.047	-0.640	-	0.008	-	-
631.627	27.657	> 0.001	20	0.518	0.232	0.052	0.520	-	-	-	-	-0.233	-0.572	-	-	-	0.012
631.779	27.809	> 0.001	16	0.077	0.223	-0.049	-0.002	-	-	-	-	-0.267	-	0.037	0.058	-	-
631.883	27.914	> 0.001	20	0.681	0.242	-0.027	0.688	0.001	-	-	-	-	-0.707	0.047	-0.016	-	-
631.915	27.945	> 0.001	15	0.102	0.072	-0.097	0.020	-	-	-	-	-	-	0.054	0.065	-	-
632.036	28.066	> 0.001	16	0.089	0.225	-0.048	0.003	-	-	-	-	-	-	-	0.066	-0.138	-
632.205	28.236	> 0.001	19	0.580	0.129	-0.038	0.577	0.121	-	-	-	-	-0.577	-	-	0.113	-
632.280	28.310	> 0.001	23	0.685	0.213	0.101	0.804	0.112	-	-	-	-0.013	-0.829	-	-	-	-0.057
632.306	28.336	> 0.001	24	0.607	0.116	0.112	0.764	0.171	-	-	-	-	-0.793	0.134	-	-	-0.092
632.426	28.456	> 0.001	22	-0.164	-0.129	0.102	-0.003	0.379	-	-	-	-	-	0.322	-	-	-0.112
632.675	28.705	> 0.001	16	0.091	0.212	-0.042	0.047	-	-	-	-	-	-	0.008	-	-0.136	-
632.811	28.841	> 0.001	21	-0.078	0.192	0.091	0.035	0.169	-	-	-	-0.133	-	-	-	-	-0.071
632.856	28.886	> 0.001	7	-	0.265	-	-0.049	-	-	-0.054	-	-0.348	-	-	-	-	-
633.108	29.139	> 0.001	19	-0.019	0.044	-0.020	-0.002	0.206	-	-	-	-0.090	-	0.215	-0.019	-	-
633.281	29.311	> 0.001	17	0.574	0.116	-0.087	0.508	-	-	-	-	-	-0.513	-0.003	0.050	-	-
633.395	29.425	> 0.001	18	0.465	0.252	-0.043	0.396	-	-	-	-	-0.253	-0.421	-0.011	0.048	-	-
633.497	29.527	> 0.001	18	0.470	0.197	-0.049	0.376	-	-	-	-	-	-0.409	-	0.061	-0.088	-
633.520	29.550	> 0.001	19	-0.017	0.005	-0.034	-0.015	0.213	-	-	-	-	-	0.265	-	0.012	-
633.766	29.797	> 0.001	5	-	-	-	-0.046	-	-	-0.122	-	-	-	-	-	-	-
633.879	29.909	> 0.001	21	0.695	0.241	-0.029	0.703	-0.005	-	-	-	0.016	-0.721	0.045	-0.016	-	-

634.265	30.295	> 0.001	25	0.648	0.111	0.107	0.807	0.157	-	-	-	0.048	-0.833	0.128	-	-	-0.093
634.295	30.326	> 0.001	23	-0.167	-0.100	0.112	-0.009	0.383	-	-	-	-0.080	-	0.318	-	-	-0.111
634.381	30.411	> 0.001	18	0.542	0.232	-0.048	0.502	-	-	-	-	-	-0.474	-0.087	-	-0.068	-
634.453	30.484	> 0.001	19	0.016	0.061	-0.013	0.019	-	-	-	-	-	-	0.049	-	-	-0.003
634.455	30.485	> 0.001	20	-0.012	0.213	0.042	-0.008	-	-	-	-	-0.267	-	0.047	-	-	-0.014
634.568	30.598	> 0.001	19	0.035	0.178	-0.030	0.036	0.122	-	-	-	-	-	-	0.007	-0.015	-
634.578	30.608	> 0.001	21	0.628	0.118	0.008	0.664	-	-	-	-	-	-0.672	-0.032	-	-	-0.001
634.607	30.637	> 0.001	20	0.001	0.223	0.035	-0.002	-	-	-	-	-	-	-	-	-0.103	-0.004
634.774	30.804	> 0.001	6	-	0.088	-	-0.043	-	-	-0.116	-	-	-	-	-	-	-
634.970	31.000	> 0.001	4	-	-	-	-0.043	-	-	-	-	-	-	-	-	-	-
634.973	31.003	> 0.001	21	0.725	0.264	-0.038	0.721	-0.046	-	-	-	-	-0.741	0.051	-	0.023	-
635.114	31.145	> 0.001	22	0.529	0.242	0.053	0.560	-	-	-	-	-0.230	-0.590	-0.026	-	-	-0.008
635.197	31.228	> 0.001	21	0.591	0.168	-0.027	0.589	0.099	-	-	-	-	-0.597	-	-0.003	0.064	-
635.244	31.274	> 0.001	22	0.508	0.186	0.037	0.506	-	-	-	-	-	-0.551	-	-	-0.043	0.012
635.476	31.506	> 0.001	25	0.617	0.110	0.090	0.759	0.193	-	-	-	-	-0.762	-	-	0.158	-0.070
635.498	31.528	> 0.001	9	-	0.272	-	-0.102	-	-	-0.119	-	-0.351	-	-	0.097	-	-
635.696	31.726	> 0.001	5	-	0.100	-	-0.039	-	-	-	-	-	-	-	-	-	-
635.778	31.808	> 0.001	18	0.076	0.224	-0.048	-0.003	-	-	-	-	-	-	0.041	0.058	-0.183	-
636.188	32.218	> 0.001	23	-0.081	0.124	0.080	0.051	0.217	-	-	-	-	-	-	-	0.056	-0.082
636.201	32.232	> 0.001	9	-	0.192	-	-0.069	-	-	-0.053	-	-0.347	-	0.117	-	-	-
636.356	32.386	> 0.001	25	-0.211	-0.230	0.104	-0.047	0.469	-	-	-	-	-	0.496	-	0.086	-0.089
636.595	32.625	> 0.001	7	-	-	-	-0.104	-	-	-0.193	-	-	-	-	0.103	-	-
636.614	32.644	> 0.001	21	-0.030	0.040	-0.019	-0.018	0.193	-	-	-	-	-	0.287	-0.015	-0.046	-
636.749	32.779	> 0.001	9	-	0.274	-0.019	-0.058	-	-	-	-	-0.359	-	-	-	-	-
637.211	33.241	> 0.001	27	0.502	0.004	0.102	0.662	0.235	-	-	-	-	-0.717	0.286	-	0.103	-0.074
637.235	33.265	> 0.001	20	0.503	0.245	-0.050	0.435	-	-	-	-	-	-0.450	-0.053	0.046	-0.119	-
637.489	33.520	> 0.001	8	-	0.093	-	-0.103	-	-	-0.191	-	-	-	-	0.109	-	-
637.625	33.656	> 0.001	23	0.752	0.320	-0.022	0.754	-0.087	-	-	-	-	-0.783	0.063	-0.010	-0.044	-
637.700	33.730	> 0.001	7	-	-	-0.099	-0.043	-	-	-	-	-	-	-	-	-	-
638.033	34.063	> 0.001	8	-	-0.009	-	-0.070	-	-	-0.115	-	-	-	0.151	-	-	-
638.216	34.246	> 0.001	22	-0.017	0.194	0.035	-0.022	-	-	-	-	-	-	0.089	-	-0.151	0.001
638.619	34.650	> 0.001	15	-	0.280	0.126	-0.044	-	-	-	-	-0.343	-	-	-	-	-0.014
638.764	34.794	> 0.001	8	-	0.086	-0.084	-0.042	-	-	-	-	-	-	-	-	-	-
638.870	34.901	> 0.001	11	-	0.189	-	-0.127	-	-	-0.122	-	-0.347	-	0.127	0.101	-	-
638.876	34.906	> 0.001	7	-	-0.005	-	-0.069	-	-	-	-	-	-	0.163	-	-	-
638.973	35.004	> 0.001	24	0.524	0.217	0.043	0.549	-	-	-	-	-	-0.577	-0.030	-	-0.079	-0.004
638.994	35.024	> 0.001	13	-	-	0.052	-0.019	-	-	-	-	-	-	-	-	-	-0.007
639.151	35.181	> 0.001	11	-	0.290	-0.051	-0.133	-	-	-	-	-0.371	-	-	0.123	-	-
640.093	36.123	> 0.001	14	-	0.087	0.062	-0.027	-	-	-	-	-	-	-	-	-	0.001
640.135	36.165	> 0.001	11	-	0.196	-0.026	-0.078	-	-	-	-	-0.354	-	0.118	-	-	-
640.369	36.399	> 0.001	11	-	0.230	-0.035	-0.059	-	-	-	-	-	-	-	-	-0.166	-
640.591	36.622	> 0.001	9	-	-	-0.128	-0.105	-	-	-	-	-	-	-	0.102	-	-
640.699	36.729	> 0.001	10	-	-0.011	-	-0.137	-	-	-0.193	-	-	-	0.161	0.115	-	-
641.449	37.479	> 0.001	10	-	0.096	-0.118	-0.114	-	-	-	-	-	-	-	0.120	-	-
642.011	38.042	> 0.001	10	-	-0.010	-0.087	-0.068	-	-	-	-	-	-	0.150	-	-	-
642.119	38.150	> 0.001	17	-	0.204	0.117	-0.068	-	-	-	-	-0.340	-	0.117	-	-	-0.007
642.258	38.289	> 0.001	17	-	0.243	0.112	-0.041	-	-	-	-	-	-	-	-	-0.122	-0.014
642.581	38.611	> 0.001	13	-	0.201	-0.055	-0.158	-	-	-	-	-0.363	-	0.132	0.126	-	-
643.046	39.077	> 0.001	13	-	0.265	-0.059	-0.132	-	-	-	-	-	-	-	0.122	-0.208	-
643.518	39.548	> 0.001	16	-	0.005	0.052	-0.054	-	-	-	-	-	-	0.130	-	-	0.009

644.072	40.102	> 0.001	13	-	0.182	-0.033	-0.076	-	-	-	-	-	-	0.104	-	-0.190	-
644.651	40.681	> 0.001	12	-	-0.010	-0.118	-0.150	-	-	-	-	-	-	0.164	0.128	-	-
645.676	41.706	> 0.001	19	-	0.168	0.103	-0.080	-	-	-	-	-	-	0.160	-	-0.147	0.011
646.561	42.591	> 0.001	15	-	0.196	-0.056	-0.160	-	-	-	-	-	-	0.143	0.128	-0.233	-
647.490	43.520	> 0.001	20	-0.097	0.317	0.286	-	0.060	-0.299	-	-	-	-	-	-	-	-
648.027	44.058	> 0.001	17	-0.042	0.159	0.208	-	-	-0.313	-	-	-	-	-	-	-	-
648.713	44.743	> 0.001	18	-0.020	0.282	0.279	-	-	-0.347	-	-	-0.216	-	-	-	-	-
648.932	44.962	> 0.001	16	-0.071	-	0.130	-	-	-0.268	-	-	-	-	-	-	-	-
649.399	45.430	> 0.001	21	-0.103	0.295	0.272	-	0.056	-0.291	-	-	0.068	-	-	-	-	-
651.118	47.148	> 0.001	20	-0.001	0.388	0.354	-	-	-0.388	-	-	-	-	-	-	-0.376	-
651.524	47.554	> 0.001	23	-0.002	0.485	0.395	-	-0.090	-0.399	-	-	-	-	-	-	-0.189	-
657.357	53.387	> 0.001	10	0.088	0.144	-	-	-	-	0.067	-0.158	-	-	-	-	-	-
657.721	53.752	> 0.001	9	0.097	-	-	-	-	-	0.039	-0.158	-	-	-	-	-	-
657.743	53.773	> 0.001	11	0.079	0.270	-	-	-	-	0.119	-0.166	-0.237	-	-	-	-	-
657.923	53.953	> 0.001	12	0.083	0.151	0.094	-	-	-	-	-0.164	-	-	-	-	-	-
658.433	54.464	> 0.001	11	0.072	-	0.040	-	-	-	-	-0.144	-	-	-	-	-	-
658.459	54.490	> 0.001	13	0.093	0.281	0.155	-	-	-	-	-0.189	-0.232	-	-	-	-	-
658.759	54.789	> 0.001	13	0.019	0.285	-	-	0.011	-	0.094	-0.107	-	-	-	-	-	-
659.301	55.331	> 0.001	15	0.036	0.303	0.140	-	0.004	-	-	-0.132	-	-	-	-	-	-
660.754	56.784	> 0.001	14	0.019	0.290	-	-	0.013	-	0.096	-0.108	-0.016	-	-	-	-	-
660.879	56.909	> 0.001	15	0.079	0.373	0.194	-	-	-	-	-0.179	-	-	-	-	-0.374	-
661.301	57.331	> 0.001	16	0.036	0.305	0.141	-	0.004	-	-	-0.132	-0.004	-	-	-	-	-
663.434	59.464	> 0.001	9	-0.056	0.260	-	-	0.011	-	-	-	-	-	-	-	-	-
663.521	59.551	> 0.001	18	0.062	0.448	0.191	-	-0.094	-	-	-0.148	-	-	-	-	-0.220	-
664.123	60.153	> 0.001	5	-0.028	-	-	-	-	-	-	-	-	-	-	-	-	-
664.208	60.239	> 0.001	6	-0.020	0.128	-	-	-	-	-	-	-	-	-	-	-	-
664.814	60.844	> 0.001	6	-0.017	-	-	-	-	-	-0.083	-	-	-	-	-	-	-
665.104	61.134	> 0.001	7	-0.010	0.121	-	-	-	-	-0.076	-	-	-	-	-	-	-
665.201	61.231	> 0.001	10	-0.050	0.250	-	-	0.009	-	-0.036	-	-	-	-	-	-	-
665.261	61.291	> 0.001	8	-0.015	0.253	-	-	-	-	-0.033	-	-0.255	-	-	-	-	-
665.399	61.429	> 0.001	8	-0.042	-	-0.077	-	-	-	-	-	-	-	-	-	-	-
665.936	61.967	> 0.001	9	-0.029	0.113	-0.056	-	-	-	-	-	-	-	-	-	-	-
666.424	62.454	> 0.001	12	-0.060	0.243	-0.011	-	0.008	-	-	-	-	-	-	-	-	-
666.617	62.647	> 0.001	10	-0.027	0.231	-0.017	-	-	-	-	-	-0.220	-	-	-	-	-
667.140	63.170	> 0.001	11	-0.051	0.266	-	-	0.015	-	-0.029	-	-0.056	-	-	-	-	-
668.416	64.446	> 0.001	13	-0.060	0.249	-0.008	-	0.010	-	-	-	-0.021	-	-	-	-	-
669.075	65.105	> 0.001	12	-0.031	0.320	0.022	-	-	-	-	-	-	-	-	-	-0.366	-
670.832	66.862	> 0.001	15	-0.045	0.382	0.031	-	-0.079	-	-	-	-	-	-	-	-0.221	-
683.309	79.339	> 0.001	5	-	0.314	-	-	-	-	-0.080	-	-0.377	-	-	-	-	-
684.853	80.884	> 0.001	3	-	-	-	-	-	-	-0.156	-	-	-	-	-	-	-
685.243	81.273	> 0.001	4	-	0.121	-	-	-	-	-0.149	-	-	-	-	-	-	-
685.894	81.924	> 0.001	7	-	0.325	0.002	-	-	-	-	-	-0.381	-	-	-	-	-
687.308	83.338	> 0.001	3	-	0.137	-	-	-	-	-	-	-	-	-	-	-	-
687.343	83.373	> 0.001	2	-	-	-	-	-	-	-	-	-	-	-	-	-	-
687.508	83.538	> 0.001	5	-	-	-0.086	-	-	-	-	-	-	-	-	-	-	-
687.807	83.837	> 0.001	6	-	0.126	-0.066	-	-	-	-	-	-	-	-	-	-	-
688.190	84.220	> 0.001	9	-	0.419	0.043	-	-	-	-	-	-	-	-	-	-0.492	-

Table S2:

Statistical summary of the models linking subcaudal over-marking responses to a global model that included the variables; ‘age’ of the donor (levels: yearling, adult); ‘sex’ of the donor (levels: male, female); ‘reproductive status of the female donor’ (levels: oestrous, non-oestrous); and ‘reproductive status of the male donor’ (levels: descended, fully descended), as well as interaction terms (n = 351). Trial ID was included as a random effect in these models. This table is the basis of the model averaging, for which results are presented in Table 2a of the main text.

The support for each model, based on Akaike criterion, is presented in the first three columns. The fourth column presents the degrees of freedom associated with each model. Subsequent columns present coefficient estimates of the parameters included in each model.
