# Supplementary material for: Will Trespassers Be Prosecuted or Assessed According to Their Merits? A Consilient Interpretation of Territoriality in a Group-Living Carnivore, the European Badger (Meles meles)
Source: PLoS One. 2015 Jul 6;10(7):e0132432. doi: 10.1371/journal.pone.0132432 (PMC4493095; doi:10.1371/journal.pone.0132432)
Supplement: S3 Table — The global model included the variables; ‘age’ of the donor and responder (levels: yearling, adult); ‘sex’ of the donor and the responder (levels: male, female); ‘reproductive status of the female’ donor and responder (levels: oestrous, non-oestrous); and ‘reproductive status of the male’ donor and responder (levels: descended, fully descended), as well as interaction terms, as factors in these models. Responder and trial ID were included as random effects in these models. This table is the basis of the model averaging, for which results are presented in Table 4 of the main text. The support for each model, based on Akaike criterion, is presented in the first three columns. The fourth column presents the degrees of freedom associated with each model. Subsequent columns present coefficient estimates of the parameters included in each model. (PDF) [file pone.0132432.s003.pdf]

Table S3

AIC	ΔAIC	*w*	d.f.	Resp. Age	Resp. Rep.	Fam.	Fam.*Resp. Age	Fam.*Resp. Rep.	Don. Age	Resp. Age*Resp. Rep.	Loc.	Don. Sex	Loc.*Don. Sex	Resp. Sex	Fam.*Don. Sex	Fam.*Loc.	Fam.*Resp. Sex	Resp. Age*Don. Age	Loc.*Resp. Rep.	Fam.*Don. Age
83.045	0	0.062	16	0.203	-0.063	-0.061	-0.178	-0.020	-	-	-	-	-	-	-	-	-	-	-	-
83.263	0.218	0.056	10	0.204	-0.064	-0.077	-0.165	-	-	-	-	-	-	-	-	-	-	-	-	-
83.895	0.850	0.041	11	0.181	-0.056	-0.080	-0.126	-	0.086	-	-	-	-	-	-	-	-	-	-	-
83.931	0.887	0.040	12	0.343	-0.047	-0.074	-0.277	-	-	-0.334	-	-	-	-	-	-	-	-	-	-
83.943	0.899	0.040	14	-	-0.111	-0.184	-	-	-	-	-0.316	0.155	0.063	-	-	-	-	-	-	-
84.145	1.100	0.036	13	0.204	-0.051	-0.174	-0.126	-	-	-	-0.197	-	-	-	-	-	-	-	-	-
84.645	1.600	0.028	11	0.202	0.090	-0.075	-0.167	-	-	-	-	-	-	-0.238	-	-	-	-	-	-
85.024	1.980	0.023	13	-	-0.095	-0.125	-	0.027	-	-	-	-	-	-	-	-	-	-	-	-
85.028	1.984	0.023	14	-	-0.101	-0.138	-	0.048	0.102	-	-	-	-	-	-	-	-	-	-	-
85.089	2.044	0.022	11	0.197	-0.073	-0.076	-0.171	-	-	-	-	0.023	-	-	-	-	-	-	-	-
85.525	2.480	0.018	16	-	-0.169	-0.051	-	0.110	-	-	-	0.210	-	-	-0.237	-	-	-	-	-
85.791	2.747	0.016	11	0.222	-	-0.063	-0.084	-	-	-	-0.151	-	-	-	-	-0.225	-	-	-	-
86.166	3.121	0.013	11	-	-0.032	-0.048	-	-	-	-	-0.076	-	-	-	-	-0.262	-	-	-	-
86.220	3.175	0.013	14	-	0.085	-0.119	-	0.020	-	-	-	-	-	-0.269	-	-	-	-	-	-
86.220	3.175	0.013	14	-	0.085	-0.119	-	0.020	-	-	-	-	-	-0.269	-	-	-	-	-	-
86.220	3.175	0.013	14	-	0.085	-0.119	-	0.020	-	-	-	-	-	-0.269	-	-	-	-	-	-
86.254	3.209	0.012	9	0.430	-	-0.079	-0.287	-	0.142	-	-	-	-	-	-	-	-	-0.566	-	-
86.255	3.211	0.012	7	-	-0.063	-0.109	-	-	-	-	-	-	-	-	-	-	-	-	-	-
86.299	3.254	0.012	15	-	0.069	-0.132	-	0.042	0.100	-	-	-	-	-0.255	-	-	-	-	-	-
86.437	3.392	0.011	11	0.233	-	-0.190	-0.095	-	-	-	-0.264	-	-	0.091	-	-	-	-	-	-
86.535	3.490	0.011	8	-	-0.054	-0.107	-	-	0.098	-	-	-	-	-	-	-	-	-	-	-
86.608	3.563	0.010	14	-	-0.107	-0.127	-	0.028	-	-	-	0.033	-	-	-	-	-	-	-	-
86.638	3.593	0.010	9	-	-	-0.030	-	-	-	-	-0.109	-	-	0.102	-	-0.302	-	-	-	-
86.693	3.649	0.010	15	-	-0.112	-0.140	-	0.049	0.100	-	-	0.030	-	-	-	-	-	-	-	-
86.739	3.695	0.010	12	-	-0.025	-0.033	-	-	0.088	-	-0.053	-	-	-	-	-0.271	-	-	-	-
86.901	3.856	0.009	10	0.222	-	-0.197	-0.063	-	-	-	-0.264	-	-	-	-	-	-	-	-	-
86.947	3.902	0.009	15	0.020	-0.098	-0.138	-	0.047	0.103	-	-	-	-	-	-	-	-	-	-	-
86.971	3.926	0.009	10	-	-	-0.015	-	-	0.096	-	-0.082	-	-	0.108	-	-0.310	-	-	-	-
86.975	3.931	0.009	14	0.016	-0.092	-0.125	-	0.026	-	-	-	-	-	-	-	-	-	-	-	-
87.133	4.088	0.008	12	-	-0.048	-0.049	-	-	-	-	-0.086	0.054	-	-	-	-0.276	-	-	-	-
87.363	4.319	0.007	11	-	-	-0.027	-	-	-	-	-0.100	-	-	0.104	-	-0.300	0.029	-	-	-
87.439	4.394	0.007	8	0.253	-	-0.063	-0.155	-	-	-	-	-	-	0.091	-	-	-	-	-	-
87.449	4.404	0.007	9	0.222	-	-0.068	-0.108	-	0.106	-	-	-	-	0.095	-	-	-	-	-	-
87.496	4.451	0.007	10	-	-0.002	-0.106	-	-	-	-	-	-	-	-0.095	-	-	0.019	-	-	-
87.496	4.451	0.007	10	-	-0.002	-0.106	-	-	-	-	-	-	-	-0.095	-	-	0.019	-	-	-
87.538	4.493	0.007	17	-	-0.082	-0.101	-	0.036	-	-	-0.061	-	-	-	-	-0.212	-	-	-	-
87.624	4.579	0.006	10	0.069	-	-0.040	-	-	-	-	-0.115	-	-	0.093	-	-0.293	-	-	-	-
87.675	4.630	0.006	15	-	0.086	-0.121	-	0.021	-	-	-	0.038	-	-0.291	-	-	-	-	-	-
87.733	4.688	0.006	8	-	-	-0.041	-	-	-	-	-0.114	-	-	-	-	-0.284	-	-	-	-
87.824	4.779	0.006	7	0.245	-	-0.072	-0.125	-	-	-	-	-	-	-	-	-	-	-	-	-
87.886	4.841	0.006	11	-	0.002	-0.108	-	-	0.094	-	-	-	-	-0.097	-	-	0.033	-	-	-
87.886	4.841	0.006	8	-	0.059	-0.108	-	-	-	-	-	-	-	-0.189	-	-	-	-	-	-
87.886	4.841	0.006	8	-	0.059	-0.108	-	-	-	-	-	-	-	-0.189	-	-	-	-	-	-
87.887	4.842	0.006	11	0.202	-	-0.193	-0.032	-	0.076	-	-0.249	-	-	-	-	-	-	-	-	-
88.024	4.979	0.005	12	0.027	-0.029	-0.051	-	-	-	-	-0.080	-	-	-	-	-0.261	-	-	-	-
88.053	5.008	0.005	12	-	0.034	-0.049	-	-	-	-	-0.076	-	-	-0.102	-	-0.259	-	-	-	-

88.069	5.024	0.005	22	-	-0.342	-0.302	-	-	-	-	-0.313	-	-	-	-	-	-	-	0.141	-
88.090	5.045	0.005	8	0.216	-	-0.077	-0.079	-	0.100	-	-	-	-	-	-	-	-	-	-	-
88.127	5.082	0.005	8	-	-0.070	-0.109	-	-	-	-	-	0.019	-	-	-	-	-	-	-	-
88.128	5.083	0.005	10	-	-0.078	-0.012	-	-	-	-	-	0.167	-	-	-0.200	-	-	-	-	-
88.136	5.092	0.005	12	-	-	-0.040	-	-	-	-	-0.249	0.101	0.080	-	-	-0.267	-	-	-	-
88.196	5.151	0.005	9	0.085	-	-0.052	-	-	-	-	-0.121	-	-	-	-	-0.276	-	-	-	-
88.200	5.155	0.005	15	0.010	0.084	-0.119	-	0.020	-	-	-	-	-	-0.266	-	-	-	-	-	-
88.209	5.164	0.005	9	-	0.061	-0.106	-	-	0.096	-	-	-	-	-0.177	-	-	-	-	-	-
88.209	5.164	0.005	9	-	0.061	-0.106	-	-	0.096	-	-	-	-	-0.177	-	-	-	-	-	-
88.237	5.192	0.005	8	0.010	-0.062	-0.109	-	-	-	-	-	-	-	-	-	-	-	-	-	-
88.332	5.287	0.004	10	-	-	-0.031	-	-	-	-	-0.117	0.029	-	0.098	-	-0.310	-	-	-	-
88.339	5.294	0.004	16	-	-0.082	-0.207	-	0.015	-	-	-0.156	-	-	-	-	-	-	-	-	-
88.367	5.322	0.004	10	-	-0.050	-0.200	-	-	-	-	-0.196	-	-	-	-	-	-	-	-	-
88.446	5.401	0.004	9	-	-0.059	-0.108	-	-	0.097	-	-	0.016	-	-	-	-	-	-	-	-
88.473	5.428	0.004	9	-	-	-0.028	-	-	0.084	-	-0.091	-	-	-	-	-0.291	-	-	-	-
88.486	5.442	0.004	9	0.016	-0.052	-0.108	-	-	0.099	-	-	-	-	-	-	-	-	-	-	-
88.608	5.563	0.004	15	0.000	-0.107	-0.127	-	0.028	-	-	-	0.033	-	-	-	-	-	-	-	-
88.685	5.640	0.004	16	-	-0.094	-0.142	-	0.050	0.140	-	-	-	-	-	-	-	-	-	-	-
88.763	5.718	0.004	11	-	-0.068	-0.017	-	-	0.087	-	-	0.154	-	-	-0.185	-	-	-	-	-
88.810	5.766	0.003	10	0.089	-	-0.039	-	-	0.088	-	-0.097	-	-	-	-	-0.282	-	-	-	-
88.812	5.767	0.003	12	-	-	-0.168	-	-	0.153	-	-0.332	0.143	0.032	-	-	-	-	-	-	-
88.835	5.790	0.003	17	-	-0.093	-0.211	-	0.039	0.090	-	-0.132	-	-	-	-	-	-	-	-	-
88.874	5.829	0.003	11	0.220	-	-0.198	-0.065	-	-	-	-0.267	0.009	-	-	-	-	-	-	-	-
88.938	5.893	0.003	16	-	-0.103	-0.133	-	0.048	0.134	-	-	-	-	-	-	-	-	-	-	-0.047
88.955	5.910	0.003	12	-	-	-0.031	-	-	-	-	-0.129	-	-	0.046	-	-0.301	-	-	-	-
88.984	5.939	0.003	10	0.256	-	-0.055	-0.161	-	-	-	-	-	-	0.116	-	-	-0.009	-	-	-
89.071	6.026	0.003	10	-	0.035	-0.099	-	-	-	-	-	0.067	-	-0.033	-	-	-	-	-	-
89.125	6.080	0.003	9	-	-	-0.041	-	-	-	-	-0.125	0.041	-	-	-	-0.297	-	-	-	-
89.168	6.124	0.003	9	0.258	-	-0.064	-0.148	-	-	-	-	-0.028	-	0.094	-	-	-	-	-	-
89.212	6.167	0.003	11	-	-0.003	-0.105	-	-	-	-	-	0.028	-	-0.104	-	-	0.013	-	-	-
89.233	6.188	0.003	11	-	-0.044	-0.191	-	-	0.079	-	-0.179	-	-	-	-	-	-	-	-	-
89.305	6.260	0.003	9	0.265	-	-0.062	-0.146	-	-	-	-	-	-	0.102	-	-	-	-	-	-
89.314	6.269	0.003	17	-	-0.102	-0.220	-	0.018	-	-	-0.170	0.053	-	-	-	-	-	-	-	-
89.431	6.386	0.003	10	-	-0.061	-0.114	-	-	0.165	-	-	0.035	-	-	-	-	-	-	-	-
89.457	6.412	0.003	12	0.125	-	-0.195	-	-	-	-	-0.379	0.087	0.075	-	-	-	-	-	-	-
89.463	6.418	0.003	11	0.013	-0.004	-0.105	-	-	-	-	-	-	-	-0.087	-	-	0.015	-	-	-
89.484	6.439	0.002	6	-	-	-0.089	-	-	0.113	-	-	-	-	0.096	-	-	-	-	-	-
89.551	6.506	0.002	12	-	-0.057	-0.002	-	-	-	-	-	0.224	-	-	-0.207	-	-	-	-	-
89.646	6.602	0.002	12	0.401	-	-0.174	-0.205	-	-	-	-0.226	-	-	-	-	-	-	-	-	-
89.650	6.605	0.002	10	-	-0.050	-0.101	-	-	-	-	-	0.064	-	-	-	-	-	-	-	-
89.691	6.646	0.002	5	-	-	-0.090	-	-	-	-	-	-	-	0.089	-	-	-	-	-	-
89.695	6.650	0.002	8	0.248	-	-0.073	-0.119	-	-	-	-	-0.019	-	-	-	-	-	-	-	-
89.711	6.667	0.002	9	-	0.060	-0.108	-	-	-	-	-	0.022	-	-0.201	-	-	-	-	-	-
89.711	6.667	0.002	9	-	0.060	-0.108	-	-	-	-	-	0.022	-	-0.201	-	-	-	-	-	-
89.747	6.702	0.002	11	-	-0.064	-0.207	-	-	-	-	-0.209	0.042	-	-	-	-	-	-	-	-
89.748	6.704	0.002	17	-	0.071	-0.200	-	0.009	-	-	-0.151	-	-	-0.230	-	-	-	-	-	-
89.762	6.717	0.002	16	-	-0.105	-0.128	-	0.036	-	-	-	0.043	-	-	-	-	-	-	-	-
89.811	6.766	0.002	8	-	-	-0.088	-	-	0.111	-	-	-	-	0.100	-	-	0.029	-	-	-
89.859	6.814	0.002	11	-	0.026	-0.013	-	-	-	-	-	0.168	-	-0.161	-0.198	-	-	-	-	-
89.869	6.824	0.002	16	0.031	-0.084	-0.125	-	0.018	-	-0.158	-	-	-	-	-	-	-	-	-	-

89.878	6.833	0.002	9	0.007	0.059	-0.108	-	-	-	-	-	-	-	-0.187	-	-	-	-	-	-
89.878	6.833	0.002	9	0.007	0.059	-0.108	-	-	-	-	-	-	-	-0.187	-	-	-	-	-	-
89.910	6.865	0.002	9	0.219	-	-0.079	-0.072	-	0.102	-	-	-0.023	-	-	-	-	-	-	-	-
89.932	6.887	0.002	4	-	-	-0.094	-	-	-	-	-	-	-	-	-	-	-	-	-	-
89.936	6.891	0.002	10	-	-	-0.028	-	-	0.082	-	-0.101	0.039	-	-	-	-0.302	-	-	-	-
89.963	6.918	0.002	7	-	-	-0.085	-	-	-	-	-	-	-	0.104	-	-	0.013	-	-	-
89.976	6.931	0.002	10	0.077	-	-0.051	-	-	-	-	-0.127	0.026	-	-	-	-0.285	-	-	-	-
89.979	6.934	0.002	10	-	0.059	-0.103	-	-	0.081	-	-	-	-	-0.181	-	-	-	-	-	-
89.987	6.942	0.002	11	0.085	-0.028	-0.114	-	-	0.124	-0.304	-	-	-	-	-	-	-	-	-	-
90.082	7.037	0.002	10	-	0.061	-0.107	-	-	0.095	-	-	0.019	-	-0.188	-	-	-	-	-	-
90.095	7.051	0.002	11	0.014	-0.075	-0.012	-	-	-	-	-	0.165	-	-	-0.201	-	-	-	-	-
90.103	7.059	0.002	10	0.033	-0.051	-0.115	-	-	0.115	-	-	-	-	-	-	-	-	-0.153	-	-
90.108	7.063	0.002	17	0.035	-0.077	-0.211	-	0.014	-	-	-0.160	-	-	-	-	-	-	-	-	-
90.109	7.064	0.002	5	-	-	-0.093	-	-	0.103	-	-	-	-	-	-	-	-	-	-	-
90.125	7.080	0.002	11	-	0.048	-0.198	-	-	-	-	-0.194	-	-	-0.152	-	-	-	-	-	-
90.125	7.080	0.002	11	-	0.048	-0.198	-	-	-	-	-0.194	-	-	-0.152	-	-	-	-	-	-
90.127	7.082	0.002	9	0.001	-0.069	-0.109	-	-	-	-	-	0.019	-	-	-	-	-	-	-	-
90.139	7.094	0.002	11	-	-0.039	-0.100	-	-	0.092	-	-	0.067	-	-	-	-	-	-	-	-
90.150	7.105	0.002	10	-	-0.053	-0.107	-	-	0.109	-	-	-	-	-	-	-	-	-	-	-
90.178	7.133	0.002	10	0.013	0.059	-0.106	-	-	0.097	-	-	-	-	-0.173	-	-	-	-	-	-
90.201	7.156	0.002	11	0.030	-0.046	-0.203	-	-	-	-	-0.201	-	-	-	-	-	-	-	-	-
90.206	7.161	0.002	8	-	-	-0.205	-	-	-	-	-0.256	-	-	0.091	-	-	-	-	-	-
90.247	7.202	0.002	13	-	-0.009	-0.189	-	-	-	-	-0.180	-	-	-0.060	-	-	0.008	-	-	-
90.336	7.291	0.002	10	-	-0.055	-0.100	-	-	0.130	-	-	-	-	-	-	-	-	-	-	-0.040
90.431	7.386	0.002	10	0.009	-0.057	-0.108	-	-	0.097	-	-	0.013	-	-	-	-	-	-	-	-
90.434	7.389	0.002	12	-	-	-0.190	-	-	-	-	-0.379	0.075	0.090	0.085	-	-	-	-	-	-
90.443	7.399	0.002	11	-	-	-0.031	-	-	0.207	-	-0.068	-	-	-	-	-0.307	-	-	-	-
90.453	7.409	0.002	7	0.071	-	-0.093	-	-	0.116	-	-	-	-	0.087	-	-	-	-	-	-
90.463	7.418	0.002	13	-	-0.069	-0.116	-	-	-	-	-0.203	0.177	-	-	-0.187	-	-	-	-	-
90.520	7.475	0.001	13	-	-0.038	-0.207	-	-	-	-	-0.224	0.114	-	-	-	-	-	-	-	-
90.560	7.516	0.001	11	-	-	-0.191	-	-	-	-	-0.377	0.100	0.074	-	-	-	-	-	-	-
90.565	7.520	0.001	9	0.078	-	-0.088	-	-	0.113	-	-	-	-	0.103	-	-	0.011	-	-	-
90.601	7.557	0.001	5	0.081	-	-0.099	-	-	-	-	-	-	-	-	-	-	-	-	-	-
90.608	7.563	0.001	6	0.086	-	-0.098	-	-	0.107	-	-	-	-	-	-	-	-	-	-	-
90.623	7.578	0.001	10	0.070	-0.041	-0.113	-	-	-	-0.242	-	-	-	-	-	-	-	-	-	-
90.627	7.582	0.001	7	-	-	-0.206	-	-	-	-	-0.253	-	-	-	-	-	-	-	-	-
90.728	7.683	0.001	12	-	-0.056	-0.198	-	-	0.075	-	-0.191	0.038	-	-	-	-	-	-	-	-
90.765	7.721	0.001	10	-	-	-0.203	-	-	-	-	-0.268	0.069	-	0.212	-	-	-	-	-	-
90.780	7.735	0.001	6	0.067	-	-0.094	-	-	-	-	-	-	-	0.081	-	-	-	-	-	-
90.813	7.768	0.001	8	0.076	-	-0.085	-	-	-	-	-	-	-	0.107	-	-	-0.004	-	-	-
90.841	7.796	0.001	8	0.093	-	-0.213	-	-	-	-	-0.256	-	-	-	-	-	-	-	-	-
90.891	7.847	0.001	9	-	-	-0.196	-	-	0.087	-	-0.235	-	-	0.097	-	-	-	-	-	-
90.908	7.864	0.001	9	0.080	-	-0.211	-	-	-	-	-0.258	-	-	0.082	-	-	-	-	-	-
90.932	7.887	0.001	7	-	-	-0.082	-	-	-	-	-	0.035	-	0.202	-	-	-	-	-	-
90.942	7.898	0.001	9	0.163	-	-0.074	-0.148	-	-	-	-	-0.036	-	-	-	-	-	-	-	-
90.970	7.925	0.001	10	-	-	-0.198	-	-	-	-	-0.246	-	-	0.104	-	-	0.014	-	-	-
90.997	7.952	0.001	8	-	-	-0.082	-	-	0.105	-	-	0.030	-	0.202	-	-	-	-	-	-
91.010	7.965	0.001	12	-	0.050	-0.189	-	-	0.079	-	-0.176	-	-	-0.145	-	-	-	-	-	-
91.023	7.978	0.001	12	0.033	-0.040	-0.194	-	-	0.081	-	-0.183	-	-	-	-	-	-	-	-	-
91.026	7.981	0.001	13	-	-	-0.244	-	-	-	-	-0.326	-	-	-0.233	-	-	0.219	-	-	-

91.052	8.007	0.001	11	-	0.108	-0.100	-	-	-	-	-	0.075	-	-0.242	-	-	-	-	-	-
91.191	8.147	0.001	7	-	-	-0.085	-	-	0.095	-	-	-	-	0.086	-	-	-	-	-	-
91.404	8.359	0.001	7	-	-	-0.089	-	-	0.114	-	-	-0.015	-	0.098	-	-	-	-	-	-
91.433	8.388	0.001	12	-	0.048	-0.205	-	-	-	-	-0.207	0.045	-	-0.173	-	-	-	-	-	-
91.448	8.403	0.001	11	0.086	-	-0.199	-	-	-	-	-0.248	-	-	0.109	-	-	-0.007	-	-	-
91.493	8.448	0.001	7	0.110	-	-0.112	-	-	0.135	-	-	-	-	-	-	-	-	-0.264	-	-
91.502	8.457	0.001	10	0.082	-	-0.202	-	-	0.090	-	-0.237	-	-	0.087	-	-	-	-	-	-
91.504	8.459	0.001	10	0.231	-	-0.001	-0.076	-	-	-	-	0.096	-	-	-0.170	-	-	-	-	-
91.614	8.569	0.001	8	-	-	-0.197	-	-	0.077	-	-0.234	-	-	-	-	-	-	-	-	-
91.614	8.569	0.001	10	0.034	0.067	-0.104	-	-	-	-	-	-	-	-0.181	-	-	-	-	-	-
91.621	8.577	0.001	8	0.093	-	-0.105	-	-	0.139	-	-	-	-	0.082	-	-	-	-0.228	-	-
91.632	8.587	0.001	6	-	-	-0.090	-	-	-	-	-	-0.013	-	0.091	-	-	-	-	-	-
91.641	8.596	0.001	11	0.008	-0.048	-0.101	-	-	-	-	-	0.060	-	-	-	-	-	-	-	-
91.661	8.616	0.001	11	-	-	-0.191	-	-	0.086	-	-0.225	-	-	0.101	-	-	0.025	-	-	-
91.701	8.656	0.001	9	0.096	-	-0.204	-	-	0.081	-	-0.236	-	-	-	-	-	-	-	-	-
91.707	8.662	0.001	10	-0.005	0.061	-0.108	-	-	-	-	-	0.024	-	-0.204	-	-	-	-	-	-
91.723	8.678	0.001	12	0.012	-0.061	-0.207	-	-	-	-	-0.210	0.039	-	-	-	-	-	-	-	-
91.746	8.702	0.001	9	-	-	-0.088	-	-	0.103	-	-	-	-	0.091	-	-	0.033	-	-	-
91.768	8.723	0.001	9	-	-	-0.089	-	-	0.111	-	-	-0.011	-	0.100	-	-	0.030	-	-	-
91.823	8.778	0.001	11	-	0.062	-0.106	-	-	0.106	-	-	-	-	-0.177	-	-	-	-	-	-
91.823	8.778	0.001	8	0.076	-	-0.087	-	-	-	-	-	0.020	-	0.194	-	-	-	-	-	-
91.852	8.807	0.001	11	-	-	0.011	-	-	-	-	-0.131	0.129	-	-	-0.128	-0.287	-	-	-	-
91.931	8.886	0.001	5	-	-	-0.094	-	-	-	-	-	-0.001	-	-	-	-	-	-	-	-
91.938	8.893	0.001	8	-	-	-0.085	-	-	-	-	-	-0.008	-	0.104	-	-	0.014	-	-	-
91.963	8.918	0.001	9	-	-	0.004	-	-	-	-	-	0.163	-	0.213	-0.175	-	-	-	-	-
91.977	8.932	0.001	10	0.224	-	-0.082	-0.086	-	0.071	-	-	-	-	-	-	-	-	-	-	0.037
91.991	8.946	0.001	12	0.027	0.045	-0.200	-	-	-	-	-0.198	-	-	-0.142	-	-	-	-	-	-
92.005	8.960	0.001	11	-	-0.059	-0.108	-	-	0.110	-	-	0.020	-	-	-	-	-	-	-	-
92.010	8.965	0.001	11	-	0.059	-0.099	-	-	0.131	-	-	-	-	-0.177	-	-	-	-	-	-0.043
92.024	8.979	0.001	11	0.041	-	-0.049	-	-	-	-	-0.120	-	-	-	-	-0.285	-	-	-	-
92.039	8.994	0.001	8	-	-	-0.097	-	-	0.194	-	-	0.009	-	0.091	-	-	-	-	-	-
92.043	8.998	0.001	11	0.024	-0.049	-0.108	-	-	0.112	-	-	-	-	-	-	-	-	-	-	-
92.058	9.013	0.001	8	0.104	-	-0.089	-	-	0.114	-	-	-	-	0.107	-	-	-	-	-	-
92.067	9.022	0.001	8	0.075	-	-0.089	-	-	0.096	-	-	-	-	0.076	-	-	-	-	-	-
92.107	9.062	0.001	6	-	-	-0.093	-	-	0.103	-	-	-0.002	-	-	-	-	-	-	-	-
92.121	9.076	0.001	10	0.010	-0.070	-0.109	-	-	-	-	-	0.021	-	-	-	-	-	-	-	-
92.130	9.086	0.001	9	-	-	-0.208	-	-	-	-	-0.262	0.015	-	0.089	-	-	-	-	-	-
92.135	9.090	0.001	8	0.082	-	-0.094	-	-	0.117	-	-	-0.031	-	0.091	-	-	-	-	-	-
92.148	9.103	0.001	14	-	0.035	-0.199	-	-	-	-	-0.215	-	-	-0.155	-	-	-	-	-	-
92.267	9.222	0.001	11	-	-0.060	-0.101	-	-	0.127	-	-	0.014	-	-	-	-	-	-	-	-0.037
92.301	9.257	0.001	7	0.103	-	-0.090	-	-	-	-	-	-	-	0.103	-	-	-	-	-	-
92.304	9.259	0.001	11	0.013	-0.054	-0.101	-	-	0.127	-	-	-	-	-	-	-	-	-	-	-0.034
92.325	9.280	0.001	11	0.066	0.068	-0.112	-	-	-	-0.237	-	-	-	-0.170	-	-	-	-	-	-
92.357	9.312	0.001	7	-	-	-0.102	-	-	0.195	-	-	0.023	-	-	-	-	-	-	-	-
92.385	9.340	0.001	8	-	-	-0.210	-	-	-	-	-0.264	0.026	-	-	-	-	-	-	-	-
92.438	9.393	0.001	11	-	-	-0.024	-	-	0.095	-	-0.088	-	-	-	-	-0.293	-	-	-	-0.018
92.450	9.405	0.001	9	-	-	-0.084	-	-	-	-	-	0.027	-	0.178	-	-	0.025	-	-	-
92.453	9.408	0.001	7	0.094	-	-0.099	-	-	0.108	-	-	-0.021	-	-	-	-	-	-	-	-
92.478	9.433	0.001	6	0.088	-	-0.099	-	-	-	-	-	-0.019	-	-	-	-	-	-	-	-
92.526	9.481	0.001	7	0.077	-	-0.095	-	-	-	-	-	-0.028	-	0.084	-	-	-	-	-	-

92.546	9.501	0.001	10	0.121	-	-0.216	-	-	0.109	-	-0.235	-	-	-	-	-	-	-0.265	-	-
92.607	9.562	0.001	10	-	-	-0.192	-	-	0.070	-	-0.235	-	-	0.087	-	-	-	-	-	-
92.618	9.573	0.001	11	-	-	-0.205	-	-	-	-	-0.277	-	-	0.045	-	-	-	-	-	-
92.619	9.574	0.001	11	0.068	-0.043	-0.113	-	-	-	-0.239	-	0.004	-	-	-	-	-	-	-	-
92.619	9.574	0.001	9	0.084	-	-0.086	-	-	-	-	-	-0.024	-	0.109	-	-	-0.003	-	-	-
92.630	9.585	0.001	13	-	-0.037	-0.198	-	-	0.148	-	-0.173	-	-	-	-	-	-	-	-	-
92.702	9.658	> 0.001	9	0.092	-	-0.085	-	-	-	-	-	-	-	0.112	-	-	0.003	-	-	-
92.771	9.727	> 0.001	10	0.099	-	-0.208	-	-	-	-	-0.255	-	-	0.094	-	-	-	-	-	-
92.817	9.772	> 0.001	9	0.091	-	-0.214	-	-	-	-	-0.259	0.008	-	-	-	-	-	-	-	-
92.841	9.797	> 0.001	9	-	-	-0.013	-	-	0.108	-	-	0.094	-	0.106	-0.148	-	-	-	-	-
92.850	9.805	> 0.001	10	-	-	-0.198	-	-	0.086	-	-0.240	0.011	-	0.095	-	-	-	-	-	-
92.858	9.813	> 0.001	8	-	-	-0.007	-	-	-	-	-	0.106	-	0.099	-0.164	-	-	-	-	-
92.863	9.819	> 0.001	11	-	-	-0.200	-	-	-	-	-0.253	0.017	-	0.103	-	-	0.012	-	-	-
92.874	9.829	> 0.001	12	0.095	-	-0.213	-	-	-	-	-0.282	-	-	0.056	-	-	-	-	-	-
92.896	9.851	> 0.001	13	0.076	-0.032	-0.200	-	-	-	-0.217	-0.191	-	-	-	-	-	-	-	-	-
92.908	9.864	> 0.001	10	0.080	-	-0.211	-	-	-	-	-0.258	0.000	-	0.082	-	-	-	-	-	-
92.983	9.938	> 0.001	13	-	-0.046	-0.190	-	-	0.077	-	-0.179	-	-	-	-	-	-	-	-	-
93.004	9.959	> 0.001	13	-	-0.046	-0.183	-	-	0.113	-	-0.175	-	-	-	-	-	-	-	-	-0.041
93.018	9.973	> 0.001	8	0.084	-	-0.106	-	-	0.191	-	-	0.004	-	-	-	-	-	-	-	-
93.133	10.088	> 0.001	8	-	-	-0.085	-	-	0.096	-	-	-0.013	-	0.088	-	-	-	-	-	-
93.224	10.179	> 0.001	9	0.093	-	-0.005	-	-	-	-	-	0.101	-	0.092	-0.181	-	-	-	-	-
93.253	10.208	> 0.001	8	0.121	-	-0.113	-	-	0.138	-	-	-0.027	-	-	-	-	-	-0.275	-	-
93.417	10.373	> 0.001	9	-	-	-0.202	-	-	0.075	-	-0.244	0.024	-	-	-	-	-	-	-	-
93.430	10.385	> 0.001	8	-	-	-0.087	-	-	0.121	-	-	-	-	0.097	-	-	-	-	-	-0.007
93.452	10.407	> 0.001	12	-	-	-0.196	-	-	0.081	-	-0.254	-	-	0.054	-	-	-	-	-	-
93.480	10.435	> 0.001	11	-	-	-0.206	-	-	0.190	-	-0.220	-	-	0.097	-	-	-	-	-	-
93.570	10.526	> 0.001	8	0.105	-	-0.015	-	-	-	-	-	0.104	-	-	-0.173	-	-	-	-	-
93.614	10.569	> 0.001	7	-	-	-0.018	-	-	-	-	-	0.110	-	-	-0.153	-	-	-	-	-
93.635	10.590	> 0.001	10	-	-	-0.012	-	-	-	-	-	0.106	-	0.099	-0.161	-	0.031	-	-	-
93.693	10.648	> 0.001	10	0.095	-	-0.205	-	-	0.081	-	-0.238	0.005	-	-	-	-	-	-	-	-
93.770	10.725	> 0.001	9	0.109	-	-0.020	-	-	0.102	-	-	0.092	-	-	-0.158	-	-	-	-	-
93.777	10.732	> 0.001	12	-	-0.051	-0.099	-	-	0.174	-	-	-	-	-	-	-	-	-	-	-0.056
93.801	10.756	> 0.001	10	-	-	-0.086	-	-	0.121	-	-	-	-	0.099	-	-	0.028	-	-	-0.016
93.827	10.783	> 0.001	11	-	-	-0.131	-	-	-	-	-0.260	0.125	-	0.096	-0.156	-	-	-	-	-
93.831	10.786	> 0.001	10	-	-	-0.219	-	-	0.168	-	-0.255	0.047	-	-	-	-	-	-	-	-
93.856	10.811	> 0.001	9	-	-	-0.030	-	-	0.199	-	-	0.132	-	-	-0.143	-	-	-	-	-
93.953	10.908	> 0.001	13	0.090	-0.043	-0.200	-	-	-	-	-0.194	-	-	-	-	-	-	-	-	-
93.998	10.953	> 0.001	8	0.115	-	-0.090	-	-	-	-	-	-0.030	-	0.107	-	-	-	-	-	-
94.002	10.957	> 0.001	8	-	-	-0.024	-	-	0.097	-	-	0.099	-	-	-0.138	-	-	-	-	-
94.054	11.009	> 0.001	7	-	-	-0.088	-	-	0.127	-	-	-	-	-	-	-	-	-	-	-0.032
94.142	11.097	> 0.001	11	0.101	-	-0.215	-	-	0.190	-	-0.219	-	-	-	-	-	-	-	-	-
94.201	11.156	> 0.001	8	0.030	-	-0.102	-	-	0.111	-	-	-0.031	-	-	-	-	-	-	-	-
94.204	11.159	> 0.001	10	-	-	-0.208	-	-	0.180	-	-0.222	-	-	-	-	-	-	-	-	-
94.332	11.288	> 0.001	7	0.040	-	-0.101	-	-	-	-	-	-0.027	-	-	-	-	-	-	-	-
94.334	11.289	> 0.001	8	0.022	-	-0.097	-	-	-	-	-	-0.036	-	0.085	-	-	-	-	-	-
94.369	11.324	> 0.001	9	0.074	-	-0.095	-	-	0.103	-	-	-	-	0.089	-	-	-	-	-	0.024
94.410	11.365	> 0.001	12	-	-	-0.209	-	-	-	-	-0.288	0.024	-	0.036	-	-	-	-	-	-
94.445	11.400	> 0.001	11	0.103	-	-0.136	-	-	-	-	-0.257	0.121	-	-	-0.162	-	-	-	-	-
94.487	11.442	> 0.001	10	-	-	-0.140	-	-	-	-	-0.262	0.129	-	-	-0.145	-	-	-	-	-
94.502	11.457	> 0.001	10	0.021	-	-0.218	-	-	-	-	-0.261	-0.002	-	-	-	-	-	-	-	-

94.556	11.511	> 0.001	13	-	-	-0.187	-	-	-	-	-0.372	0.110	0.066	-	-0.011	-	-	-	-	-
94.556	11.512	> 0.001	18	-	0.015	-0.054	-	-	-	-	-0.279	-	-	-	-	-0.255	-	-	0.044	-
94.577	11.532	> 0.001	8	0.087	-	-0.098	-	-	0.105	-	-	-	-	-	-	-	-	-	-	0.007
94.741	11.696	> 0.001	11	0.141	-	-0.209	-	-	-	-	-0.249	-	-	0.086	-	-	-	-	-	-
94.749	11.704	> 0.001	10	0.118	-	-0.213	-	-	-	-	-0.249	-	-	-	-	-	-	-	-	-
94.820	11.775	> 0.001	11	-	-	-0.195	-	-	0.090	-	-0.235	-	-	0.098	-	-	-	-	-	0.002
95.102	12.057	> 0.001	9	-	-	-0.087	-	-	0.080	-	-	-	-	0.087	-	-	-	-	-	0.025
95.186	12.141	> 0.001	9	0.110	-	-0.101	-	-	0.210	-	-	-	-	-	-	-	-	-0.331	-	-0.093
95.319	12.275	> 0.001	9	0.042	-	-0.017	-	-	-	-	-	0.095	-	-	-0.174	-	-	-	-	-
95.338	12.293	> 0.001	9	-	-	-0.086	-	-	0.125	-	-	-0.016	-	0.099	-	-	-	-	-	-0.011
95.457	12.412	> 0.001	11	0.135	-	-0.203	-	-	0.087	-	-0.224	-	-	-	-	-	-	-	-	-
95.554	12.509	> 0.001	11	-	-	-0.219	-	-	0.195	-	-0.242	0.045	-	-	-	-	-	-	-	-
95.569	12.524	> 0.001	10	-	-	-0.193	-	-	0.095	-	-0.233	-	-	-	-	-	-	-	-	-0.022
95.641	12.596	> 0.001	11	0.098	-	-0.206	-	-	0.072	-	-0.237	-	-	-	-	-	-	-	-	0.018
95.664	12.619	> 0.001	11	-	-	-0.136	-	-	0.069	-	-0.244	0.120	-	-	-0.134	-	-	-	-	-
95.729	12.685	> 0.001	9	-	-	-0.089	-	-	0.289	-	-	0.025	-	-	-	-	-	-	-	-0.089
95.752	12.707	> 0.001	13	-	-0.134	-	-	-	0.231	-	-0.124	0.233	-0.031	-	-	-	-	-	-	-
96.049	13.005	> 0.001	8	-	-	-0.088	-	-	0.128	-	-	-0.004	-	-	-	-	-	-	-	-0.033
96.414	13.369	> 0.001	9	0.095	-	-0.099	-	-	0.109	-	-	-0.022	-	-	-	-	-	-	-	0.004
96.543	13.498	> 0.001	12	-	-	-0.170	-	-	0.468	-	-0.181	-	-	-	-	-	-	-	-	-0.248
96.609	13.564	> 0.001	17	-	0.004	-0.201	-	-	-	-	-0.419	-	-	-	-	-	-	-	0.045	-
96.729	13.684	> 0.001	11	0.116	-	-0.214	-	-	-	-	-0.252	0.008	-	-	-	-	-	-	-	-
96.883	13.838	> 0.001	18	-	0.032	-0.190	-	-	0.100	-	-0.392	-	-	-	-	-	-	-	0.013	-
97.387	14.343	> 0.001	11	-	-	-0.198	-	-	0.088	-	-0.243	0.023	-	-	-	-	-	-	-	-0.016
97.986	14.941	> 0.001	10	-	-	-0.022	-	-	0.104	-	-	0.098	-	-	-0.137	-	-	-	-	-0.008
97.991	14.946	> 0.001	18	-	-0.017	-0.208	-	-	-	-	-0.430	0.042	-	-	-	-	-	-	0.054	-
98.155	15.110	> 0.001	18	-	0.141	-0.198	-	-	-	-	-0.416	-	-	-0.205	-	-	-	-	0.041	-
98.382	15.338	> 0.001	18	0.036	0.010	-0.204	-	-	-	-	-0.422	-	-	-	-	-	-	-	0.044	-
102.229	19.184	> 0.001	14	-	-0.113	-	-	-	-	-	-0.168	0.239	0.025	-	-	-	-	-	-	-
102.257	19.212	> 0.001	12	-	-0.142	-	-	-	-	-	-0.159	0.171	0.028	-	-	-	-	-	-	-
103.127	20.082	> 0.001	11	0.127	-	-	-	-	0.241	-	-0.187	0.157	-0.019	-	-	-	-	-	-	-
103.643	20.599	> 0.001	13	-	0.019	-	-	-	-	-	-0.157	0.174	0.028	-0.249	-	-	-	-	-	-
103.956	20.912	> 0.001	10	-	-	-	-	-	0.223	-	-0.190	0.165	-0.015	-	-	-	-	-	-	-
104.193	21.148	> 0.001	11	-	-	-	-	-	0.232	-	-0.194	0.144	-0.002	0.081	-	-	-	-	-	-
104.256	21.211	> 0.001	13	-0.002	-0.143	-	-	-	-	-	-0.159	0.172	0.028	-	-	-	-	-	-	-
105.551	22.506	> 0.001	11	-	-	-	-	-	0.273	-	-0.182	0.179	-0.021	-	-	-	-	-	-	-
106.924	23.880	> 0.001	19	-	-0.039	-	-	-	-	-	-0.467	0.123	0.141	-	-	-	-	-	-0.007	-
107.002	23.958	> 0.001	11	-	-	-	-	-	-	-	-0.239	0.153	0.054	0.228	-	-	-	-	-	-
107.529	24.484	> 0.001	12	-	-	-	-	-	0.281	-	-0.175	0.182	-0.026	-	-	-	-	-	-	-
108.535	25.490	> 0.001	6	-	-0.070	-	-	-	0.142	-	-	-	-	-	-	-	-	-	-	-
109.260	26.215	> 0.001	9	-	-	-	-	-	-	-	-0.225	0.102	0.044	-	-	-	-	-	-	-
109.431	26.387	> 0.001	9	-	-0.075	-	-	-	0.126	-	-0.010	-	-	-	-	-	-	-	-	-
109.443	26.399	> 0.001	7	-	0.157	-	-	-	0.139	-	-	-	-	-0.350	-	-	-	-	-	-
109.443	26.399	> 0.001	7	-	0.157	-	-	-	0.139	-	-	-	-	-0.350	-	-	-	-	-	-
109.590	26.545	> 0.001	10	0.099	-	-	-	-	-	-	-0.225	0.092	0.044	-	-	-	-	-	-	-
109.636	26.591	> 0.001	5	-	-0.085	-	-	-	-	-	-	-	-	-	-	-	-	-	-	-
109.792	26.747	> 0.001	9	-	0.119	-	-	-	0.130	-	-	0.023	-	-0.121	-	-	-	-	-	-
109.904	26.859	> 0.001	8	-	-0.085	-	-	-	-	-	-0.025	-	-	-	-	-	-	-	-	-
109.952	26.907	> 0.001	7	-0.059	-0.077	-	-	-	0.137	-	-	-	-	-	-	-	-	-	-	-
110.060	27.015	> 0.001	7	-	-0.057	-	-	-	0.144	-	-	-0.039	-	-	-	-	-	-	-	-

110.080	27.035	> 0.001	10	-	-	-	-	-	-	-	-0.229	0.083	0.056	0.068	-	-	-	-	-	-
110.409	27.364	> 0.001	6	-	0.158	-	-	-	-	-	-	-	-	-0.374	-	-	-	-	-	-
110.409	27.364	> 0.001	6	-	0.158	-	-	-	-	-	-	-	-	-0.374	-	-	-	-	-	-
110.443	27.399	> 0.001	8	-	0.119	-	-	-	-	-	-	0.029	-	-0.133	-	-	-	-	-	-
110.443	27.399	> 0.001	8	-	0.119	-	-	-	-	-	-	0.029	-	-0.133	-	-	-	-	-	-
110.635	27.590	> 0.001	8	-	0.151	-	-	-	0.107	-	-	-	-	-0.355	-	-	-	-	-	-
110.635	27.590	> 0.001	8	-	0.151	-	-	-	0.107	-	-	-	-	-0.355	-	-	-	-	-	-
110.648	27.603	> 0.001	10	-	0.115	-	-	-	0.124	-	-0.009	-	-	-0.293	-	-	-	-	-	-
110.648	27.603	> 0.001	10	-	0.115	-	-	-	0.124	-	-0.009	-	-	-0.293	-	-	-	-	-	-
110.704	27.659	> 0.001	11	0.090	-	-	-	-	-	-	-0.228	0.076	0.054	0.059	-	-	-	-	-	-
110.761	27.716	> 0.001	8	-0.064	0.159	-	-	-	0.133	-	-	-	-	-0.365	-	-	-	-	-	-
110.761	27.716	> 0.001	8	-0.064	0.159	-	-	-	0.133	-	-	-	-	-0.365	-	-	-	-	-	-
110.820	27.775	> 0.001	6	-0.070	-0.093	-	-	-	-	-	-	-	-	-	-	-	-	-	-	-
110.977	27.932	> 0.001	11	-	0.079	-	-	-	-	-	-0.038	0.062	-	-0.080	-	-	-	-	-	-
110.999	27.954	> 0.001	9	-	-0.035	-	-	-	0.135	-	-	0.016	-	-	-	-	-	-	-	-
111.040	27.995	> 0.001	9	-	0.116	-	-	-	-	-	-0.023	-	-	-0.309	-	-	-	-	-	-
111.040	27.995	> 0.001	9	-	0.116	-	-	-	-	-	-0.023	-	-	-0.309	-	-	-	-	-	-
111.081	28.037	> 0.001	11	0.007	-	-	-	-	-	-	-0.225	0.081	0.044	-	-	-	-	-	-	-
111.114	28.069	> 0.001	8	-	0.154	-	-	-	0.140	-	-	-0.033	-	-0.327	-	-	-	-	-	-
111.114	28.069	> 0.001	8	-	0.154	-	-	-	0.140	-	-	-0.033	-	-0.327	-	-	-	-	-	-
111.138	28.093	> 0.001	13	-	-	-	-	-	-	-	-0.281	0.099	0.091	0.012	-	-	-	-	-	-
111.224	28.180	> 0.001	6	-	-0.072	-	-	-	-	-	-	-0.037	-	-	-	-	-	-	-	-
111.274	28.229	> 0.001	8	-	-0.057	-	-	-	0.209	-	-	-0.020	-	-	-	-	-	-	-	-
111.294	28.250	> 0.001	10	-0.029	-0.078	-	-	-	0.124	-	-0.009	-	-	-	-	-	-	-	-	-
111.399	28.354	> 0.001	10	-	-0.071	-	-	-	0.127	-	-0.009	-0.010	-	-	-	-	-	-	-	-
111.474	28.429	> 0.001	3	-	-	-	-	-	0.152	-	-	-	-	-	-	-	-	-	-	-
111.475	28.430	> 0.001	7	-0.075	0.161	-	-	-	-	-	-	-	-	-0.391	-	-	-	-	-	-
111.475	28.430	> 0.001	7	-0.075	0.161	-	-	-	-	-	-	-	-	-0.391	-	-	-	-	-	-
111.572	28.527	> 0.001	8	-	-0.073	-	-	-	0.141	-	-	-	-	-	-	-	-	-	-	-
111.684	28.639	> 0.001	9	-0.036	-0.090	-	-	-	-	-	-0.023	-	-	-	-	-	-	-	-	-
111.761	28.716	> 0.001	10	-	0.208	-	-	-	0.131	-	-	0.034	-	-0.374	-	-	-	-	-	-
111.766	28.722	> 0.001	8	-0.045	-0.066	-	-	-	0.139	-	-	-0.026	-	-	-	-	-	-	-	-
111.793	28.748	> 0.001	8	-	-0.051	-	-	-	-	-	-	0.010	-	-	-	-	-	-	-	-
111.815	28.770	> 0.001	6	-	-	-	-	-	0.150	-	-	-0.016	-	0.208	-	-	-	-	-	-
111.863	28.818	> 0.001	12	-	-0.043	-	-	-	0.119	-	-0.025	0.068	-	-	-	-	-	-	-	-
111.875	28.830	> 0.001	6	-	-	-	-	-	0.128	-	-0.071	-	-	-	-	-	-	-	-	-
111.895	28.850	> 0.001	9	-	-0.084	-	-	-	-	-	-0.025	-0.006	-	-	-	-	-	-	-	-
111.930	28.885	> 0.001	9	-0.060	0.124	-	-	-	-	-	-	0.045	-	-0.164	-	-	-	-	-	-
111.951	28.907	> 0.001	8	-0.059	-0.077	-	-	-	0.137	-	-	-	-	-	-	-	-	0.002	-	-
111.997	28.952	> 0.001	10	-	-0.034	-	-	-	0.208	-	-	0.043	-	-	-	-	-	-	-	-
112.012	28.968	> 0.001	11	-	0.112	-	-	-	0.096	-	-0.014	-	-	-0.299	-	-	-	-	-	-
112.063	29.018	> 0.001	11	-	-0.055	-	-	-	-	-	-0.040	0.067	-	-	-	-	-	-	-	-
112.098	29.053	> 0.001	9	-0.057	0.154	-	-	-	0.105	-	-	-	-	-0.368	-	-	-	-	-	-
112.116	29.071	> 0.001	4	-	-	-	-	-	0.161	-	-	-	-	0.075	-	-	-	-	-	-
112.132	29.087	> 0.001	7	-	-	-	-	-	0.244	-	-	0.017	-	0.208	-	-	-	-	-	-
112.137	29.093	> 0.001	7	-	0.156	-	-	-	-	-	-	-0.030	-	-0.354	-	-	-	-	-	-
112.137	29.093	> 0.001	7	-	0.156	-	-	-	-	-	-	-0.030	-	-0.354	-	-	-	-	-	-
112.191	29.146	> 0.001	9	-	-	-	-	-	0.125	-	-0.077	0.023	-	0.223	-	-	-	-	-	-
112.272	29.228	> 0.001	9	-	0.159	-	-	-	0.207	-	-	-0.013	-	-0.336	-	-	-	-	-	-
112.312	29.267	> 0.001	5	-	-	-	-	-	-	-	-0.090	-	-	-	-	-	-	-	-	-

112.372	29.327	> 0.001	7	-	-	-	-	-	0.137	-	-0.074	-	-	0.078	-	-	-	-	-	-
112.374	29.329	> 0.001	9	-	0.148	-	-	-	0.110	-	-	-0.029	-	-0.335	-	-	-	-	-	-
112.427	29.382	> 0.001	4	-	-	-	-	-	0.151	-	-	-0.057	-	-	-	-	-	-	-	-
112.443	29.398	> 0.001	9	-	0.206	-	-	-	-	-	-	0.029	-	-0.394	-	-	-	-	-	-
112.443	29.398	> 0.001	9	-	0.206	-	-	-	-	-	-	0.029	-	-0.394	-	-	-	-	-	-
112.443	29.398	> 0.001	9	-	0.206	-	-	-	-	-	-	0.029	-	-0.394	-	-	-	-	-	-
112.458	29.413	> 0.001	11	-0.034	0.118	-	-	-	0.121	-	-0.007	-	-	-0.303	-	-	-	-	-	-
112.458	29.413	> 0.001	9	-0.032	0.168	-	-	-	0.131	-	-	-	-	-0.358	-	-	-	-	-	-
112.471	29.426	> 0.001	9	-	0.154	-	-	-	0.135	-	-	-	-	-0.350	-	-	-	-	-	-
112.471	29.426	> 0.001	9	-	0.154	-	-	-	0.135	-	-	-	-	-0.350	-	-	-	-	-	-
112.471	29.426	> 0.001	9	-	0.154	-	-	-	0.135	-	-	-	-	-0.350	-	-	-	-	-	-
112.569	29.524	> 0.001	8	-	-	-	-	-	-	-	-0.096	0.031	-	0.224	-	-	-	-	-	-
112.601	29.556	> 0.001	5	-	-	-	-	-	0.161	-	-	-0.069	-	0.088	-	-	-	-	-	-
112.639	29.594	> 0.001	11	-	0.115	-	-	-	0.124	-	-0.008	-0.005	-	-0.289	-	-	-	-	-	-
112.651	29.606	> 0.001	12	-0.035	-	-	-	-	-	-	-0.229	0.106	0.025	-	-	-	-	-	-	-
112.686	29.642	> 0.001	9	-0.055	0.157	-	-	-	0.134	-	-	-0.017	-	-0.351	-	-	-	-	-	-
112.691	29.647	> 0.001	9	0.004	-0.054	-	-	-	0.154	-0.232	-	-	-	-	-	-	-	-	-	-
112.715	29.670	> 0.001	7	-0.060	-0.085	-	-	-	-	-	-	-0.020	-	-	-	-	-	-	-	-
112.752	29.707	> 0.001	10	-0.042	0.119	-	-	-	-	-	-0.020	-	-	-0.322	-	-	-	-	-	-
112.752	29.707	> 0.001	10	-0.042	0.119	-	-	-	-	-	-0.020	-	-	-0.322	-	-	-	-	-	-
112.760	29.715	> 0.001	9	-0.064	0.159	-	-	-	0.132	-	-	-	-	-0.365	-	-	-	0.008	-	-
112.766	29.721	> 0.001	10	-0.043	-0.042	-	-	-	0.132	-	-	0.039	-	-	-	-	-	-	-	-
112.831	29.786	> 0.001	11	-	-0.082	-	-	-	0.101	-	-0.015	-	-	-	-	-	-	-	-	-
112.858	29.813	> 0.001	2	-	-	-	-	-	-	-	-	-	-	-	-	-	-	-	-	-
112.865	29.820	> 0.001	5	-	-	-	-	-	0.244	-	-	-0.030	-	-	-	-	-	-	-	-
112.874	29.829	> 0.001	12	-	0.182	-	-	-	-	-	-0.040	0.083	-	-0.363	-	-	-	-	-	-
112.893	29.848	> 0.001	9	-0.052	-0.068	-	-	-	0.207	-	-	-0.005	-	-	-	-	-	-	-	-
113.002	29.958	> 0.001	13	-	0.089	-	-	-	0.117	-	-0.024	-	-	-0.249	-	-	-	-	-	-
113.040	29.995	> 0.001	10	-	0.116	-	-	-	-	-	-0.023	0.000	-	-0.309	-	-	-	-	-	-
113.040	29.995	> 0.001	10	-	0.116	-	-	-	-	-	-0.023	0.000	-	-0.309	-	-	-	-	-	-
113.088	30.044	> 0.001	8	-0.038	0.171	-	-	-	-	-	-	-	-	-0.383	-	-	-	-	-	-
113.088	30.044	> 0.001	8	-0.038	0.171	-	-	-	-	-	-	-	-	-0.383	-	-	-	-	-	-
113.132	30.087	> 0.001	11	-	-0.070	-	-	-	0.174	-	0.002	-	-	-	-	-	-	-	-	-
113.160	30.115	> 0.001	6	-	-	-	-	-	-	-	-0.093	-	-	0.069	-	-	-	-	-	-
113.166	30.121	> 0.001	12	-	0.086	-	-	-	-	-	-0.044	-	-	-0.266	-	-	-	-	-	-
113.166	30.121	> 0.001	12	-	0.086	-	-	-	-	-	-0.044	-	-	-0.266	-	-	-	-	-	-
113.192	30.147	> 0.001	9	-0.048	-0.079	-	-	-	0.134	-	-	-	-	-	-	-	-	-	-	-
113.207	30.162	> 0.001	5	-	-	-	-	-	-	-	-	-0.011	-	0.207	-	-	-	-	-	-
113.237	30.192	> 0.001	9	-	-0.062	-	-	-	0.139	-	-	-0.033	-	-	-	-	-	-	-	-
113.292	30.247	> 0.001	11	-0.027	-0.077	-	-	-	0.124	-	-0.008	-0.003	-	-	-	-	-	-	-	-
113.292	30.247	> 0.001	11	-0.027	-0.078	-	-	-	0.126	-	-0.008	-	-	-	-	-	-	-0.013	-	-
113.301	30.256	> 0.001	5	-	-	-	-	-	0.128	-	-	-	-	0.057	-	-	-	-	-	-
113.316	30.272	> 0.001	6	-	-	-	-	-	0.244	-	-	-0.044	-	0.081	-	-	-	-	-	-
113.344	30.299	> 0.001	11	-	-0.071	-	-	-	0.145	-	-0.007	-0.006	-	-	-	-	-	-	-	-
113.418	30.373	> 0.001	4	0.018	-	-	-	-	0.153	-	-	-	-	-	-	-	-	-	-	-
113.446	30.401	> 0.001	7	-	-	-	-	-	0.129	-	-	-0.015	-	0.190	-	-	-	-	-	-
113.450	30.405	> 0.001	8	-0.070	0.160	-	-	-	-	-	-	-0.010	-	-0.383	-	-	-	-	-	-
113.450	30.405	> 0.001	8	-0.070	0.160	-	-	-	-	-	-	-0.010	-	-0.383	-	-	-	-	-	-
113.460	30.415	> 0.001	9	-0.052	-0.059	-	-	-	-	-	-	0.037	-	-	-	-	-	-	-	-
113.503	30.458	> 0.001	7	0.046	-	-	-	-	0.131	-	-0.070	-	-	-	-	-	-	-	-	-

113.600	30.555	> 0.001	10	-0.003	0.171	-	-	-	0.149	-0.223	-	-	-	-0.349	-	-	-	-	-	-
113.608	30.563	> 0.001	8	-	-	-	-	-	0.106	-	-0.080	-	-	0.061	-	-	-	-	-	-
113.619	30.575	> 0.001	7	-	-	-	-	-	0.129	-	-0.064	-0.029	-	-	-	-	-	-	-	-
113.627	30.582	> 0.001	7	0.034	-	-	-	-	0.152	-	-	-0.023	-	0.205	-	-	-	-	-	-
113.679	30.634	> 0.001	10	-0.039	-0.091	-	-	-	-	-	-0.023	0.004	-	-	-	-	-	-	-	-
113.758	30.713	> 0.001	9	-0.033	-0.068	-	-	-	0.138	-	-	-0.024	-	-	-	-	-	-	-	-
113.764	30.719	> 0.001	9	-0.044	-0.066	-	-	-	0.141	-	-	-0.027	-	-	-	-	-	-0.013	-	-
113.783	30.739	> 0.001	3	-	-	-	-	-	-	-	-	-0.058	-	-	-	-	-	-	-	-
113.795	30.750	> 0.001	12	0.025	-0.061	-	-	-	0.147	-0.255	0.003	-	-	-	-	-	-	-	-	-
113.845	30.800	> 0.001	10	-0.070	0.221	-	-	-	-	-	-	0.067	-	-0.436	-	-	-	-	-	-
113.872	30.827	> 0.001	8	-	-	-	-	-	0.139	-	-0.064	-0.040	-	0.085	-	-	-	-	-	-
113.881	30.837	> 0.001	12	-0.038	-0.061	-	-	-	-	-	-0.039	0.086	-	-	-	-	-	-	-	-
113.893	30.848	> 0.001	3	-	-	-	-	-	-	-	-	-	-	0.064	-	-	-	-	-	-
113.959	30.915	> 0.001	6	-	-	-	-	-	0.132	-	-	-0.065	-	0.071	-	-	-	-	-	-
114.005	30.960	> 0.001	10	-0.053	0.156	-	-	-	0.126	-	-	-	-	-0.364	-	-	-	-	-	-
114.012	30.968	> 0.001	12	-	0.112	-	-	-	0.096	-	-0.014	-	-	-0.299	-	-	-	-	-	-
114.027	30.982	> 0.001	16	-	0.065	-	-	-	0.158	-	-0.205	-	-	-	-	-	-	-	-0.068	-
114.049	31.004	> 0.001	6	0.039	-	-	-	-	-	-	-0.089	-	-	-	-	-	-	-	-	-
114.096	31.051	> 0.001	6	-	-	-	-	-	-	-	-0.083	-0.026	-	-	-	-	-	-	-	-
114.105	31.061	> 0.001	8	-0.016	-0.073	-	-	-	-	-0.153	-	-	-	-	-	-	-	-	-	-
114.106	31.061	> 0.001	5	0.044	-	-	-	-	0.154	-	-	-0.067	-	-	-	-	-	-	-	-
114.112	31.067	> 0.001	5	0.005	-	-	-	-	0.161	-	-	-	-	0.074	-	-	-	-	-	-
114.178	31.133	> 0.001	8	0.033	-	-	-	-	0.138	-	-0.073	-	-	0.074	-	-	-	-	-	-
114.188	31.143	> 0.001	10	0.035	-0.032	-	-	-	0.160	-0.268	-	-0.044	-	-	-	-	-	-	-	-
114.231	31.186	> 0.001	9	0.045	-	-	-	-	-	-	-0.093	0.023	-	0.220	-	-	-	-	-	-
114.255	31.210	> 0.001	10	-	0.151	-	-	-	0.133	-	-	-0.026	-	-0.333	-	-	-	-	-	-
114.270	31.225	> 0.001	10	-0.014	-0.055	-	-	-	0.140	-0.323	-	-	-	-	-	-	-	0.229	-	-
114.289	31.244	> 0.001	11	-	-0.038	-	-	-	0.142	-	-	0.017	-	-	-	-	-	-	-	-
114.354	31.310	> 0.001	12	-	0.120	-	-	-	0.172	-	0.004	-	-	-0.291	-	-	-	-	-	-
114.418	31.374	> 0.001	6	0.033	-	-	-	-	0.163	-	-	-0.076	-	0.085	-	-	-	-	-	-
114.419	31.375	> 0.001	4	-	-	-	-	-	-	-	-	-0.069	-	0.076	-	-	-	-	-	-
114.629	31.584	> 0.001	11	-0.022	0.126	-	-	-	-	-	-0.021	-	-	-0.319	-	-	-	-	-	-
114.635	31.590	> 0.001	8	-0.023	-0.089	-	-	-	-	-	-	-0.013	-	-	-	-	-	-	-	-
114.675	31.630	> 0.001	6	0.034	-	-	-	-	0.242	-	-	-0.039	-	-	-	-	-	-	-	-
114.691	31.646	> 0.001	10	-	-0.067	-	-	-	0.177	-	-	-0.018	-	-	-	-	-	-	-	-
114.712	31.667	> 0.001	11	-0.048	0.120	-	-	-	-	-	-0.022	0.012	-	-0.331	-	-	-	-	-	-
114.745	31.700	> 0.001	17	-	0.307	-	-	-	0.156	-	-0.205	-	-	-0.364	-	-	-	-	-0.072	-
114.747	31.702	> 0.001	12	-0.023	-0.085	-	-	-	0.098	-	-0.014	-	-	-	-	-	-	-	-	-
114.752	31.707	> 0.001	7	-	-	-	-	-	-	-	-0.084	-0.037	-	0.075	-	-	-	-	-	-
114.783	31.738	> 0.001	7	-	-	-	-	-	0.214	-	-	-0.041	-	0.066	-	-	-	-	-	-
114.815	31.770	> 0.001	12	-	-0.079	-	-	-	0.101	-	-0.014	-0.007	-	-	-	-	-	-	-	-
114.835	31.790	> 0.001	9	-0.024	0.173	-	-	-	-	-0.146	-	-	-	-0.380	-	-	-	-	-	-
114.835	31.790	> 0.001	9	-0.024	0.173	-	-	-	-	-0.146	-	-	-	-0.380	-	-	-	-	-	-
114.835	31.790	> 0.001	9	-0.024	0.173	-	-	-	-	-0.146	-	-	-	-0.380	-	-	-	-	-	-
114.846	31.801	> 0.001	3	0.008	-	-	-	-	-	-	-	-	-	-	-	-	-	-	-	-
114.947	31.902	> 0.001	8	-	-	-	-	-	0.219	-	-0.047	-	-	-	-	-	-	-	-	-
114.957	31.912	> 0.001	11	0.007	-0.076	-	-	-	-	-0.176	-0.018	-	-	-	-	-	-	-	-	-
115.007	31.962	> 0.001	8	0.061	-	-	-	-	0.133	-	-0.058	-0.041	-	-	-	-	-	-	-	-
115.030	31.985	> 0.001	7	0.027	-	-	-	-	-	-	-0.092	-	-	0.065	-	-	-	-	-	-
115.047	32.002	> 0.001	12	-0.023	-0.073	-	-	-	0.170	-	0.002	-	-	-	-	-	-	-	-	-

115.048	32.003	> 0.001	10	-0.037	-0.070	-	-	-	0.134	-	-	-0.023	-	-	-	-	-	-	-	-
115.048	32.004	> 0.001	9	-0.031	0.169	-	-	-	-	-	-	-0.012	-	-0.373	-	-	-	-	-	-
115.102	32.057	> 0.001	13	-0.020	0.090	-	-	-	-	-	-0.042	-	-	-0.279	-	-	-	-	-	-
115.102	32.057	> 0.001	6	0.025	-	-	-	-	-	-	-	-0.016	-	0.205	-	-	-	-	-	-
115.114	32.069	> 0.001	12	-	-0.067	-	-	-	0.172	-	0.004	-0.008	-	-	-	-	-	-	-	-
115.148	32.103	> 0.001	13	-	0.086	-	-	-	-	-	-0.045	0.008	-	-0.272	-	-	-	-	-	-
115.177	32.132	> 0.001	5	0.029	-	-	-	-	0.167	-	-	-	-	-	-	-	-	-0.131	-	-
115.179	32.134	> 0.001	10	-0.051	-0.079	-	-	-	0.130	-	-	-	-	-	-	-	-	0.030	-	-
115.212	32.167	> 0.001	9	-	-	-	-	-	0.110	-	-0.070	-0.036	-	0.068	-	-	-	-	-	-
115.216	32.171	> 0.001	7	0.025	-	-	-	-	0.243	-	-	-0.050	-	0.079	-	-	-	-	-	-
115.231	32.186	> 0.001	8	0.058	-	-	-	-	0.146	-	-0.063	-	-	-	-	-	-	-0.139	-	-
115.238	32.193	> 0.001	12	-0.018	-0.077	-	-	-	0.122	-	-0.011	-	-	-	-	-	-	-	-	-
115.279	32.234	> 0.001	6	0.011	-	-	-	-	0.128	-	-	-	-	0.055	-	-	-	-	-	-
115.293	32.248	> 0.001	8	-	-	-	-	-	0.175	-	-0.060	-0.018	-	-	-	-	-	-	-	-
115.317	32.272	> 0.001	9	-0.022	0.161	-	-	-	-	-	-	0.000	-	-0.392	-	-	-	-	-	-
115.339	32.294	> 0.001	9	0.039	-	-	-	-	0.106	-	-0.078	-	-	0.055	-	-	-	-	-	-
115.385	32.340	> 0.001	10	-	-	-	-	-	0.131	-	-0.091	-	-	0.063	-	-	-	-	-	-
115.399	32.354	> 0.001	9	-	-	-	-	-	0.228	-	-0.049	-	-	0.079	-	-	-	-	-	-
115.449	32.404	> 0.001	9	0.051	-	-	-	-	0.142	-	-0.059	-0.050	-	0.080	-	-	-	-	-	-
115.460	32.415	> 0.001	10	-0.050	-0.059	-	-	-	-	-	-	0.038	-	-	-	-	-	-	-	-
115.479	32.434	> 0.001	11	-0.049	-0.089	-	-	-	-	-	-0.030	-	-	-	-	-	-	-	-	-
115.541	32.497	> 0.001	6	0.048	-	-	-	-	0.158	-	-	-	-	0.100	-	-	-	-	-	-
115.592	32.547	> 0.001	4	0.035	-	-	-	-	-	-	-	-0.066	-	-	-	-	-	-	-	-
115.649	32.604	> 0.001	7	0.052	-	-	-	-	-	-	-0.078	-0.037	-	-	-	-	-	-	-	-
115.665	32.620	> 0.001	9	-	-	-	-	-	0.175	-	-0.061	-0.032	-	0.082	-	-	-	-	-	-
115.676	32.631	> 0.001	11	-0.031	-0.092	-	-	-	-	-	-0.023	0.006	-	-	-	-	-	-	-	-
115.706	32.661	> 0.001	7	0.082	-	-	-	-	0.160	-	-	-0.079	-	0.113	-	-	-	-	-	-
115.725	32.680	> 0.001	7	0.038	-	-	-	-	0.133	-	-	-0.073	-	0.067	-	-	-	-	-	-
115.758	32.714	> 0.001	6	0.059	-	-	-	-	0.171	-	-	-0.069	-	-	-	-	-	-0.157	-	-
115.868	32.823	> 0.001	9	0.005	-0.059	-	-	-	-	-0.176	-	-0.031	-	-	-	-	-	-	-	-
115.889	32.844	> 0.001	15	-	0.022	-	-	-	-	-	-0.230	-	-	-	-	-	-	-	-0.021	-
115.891	32.846	> 0.001	4	-0.003	-	-	-	-	-	-	-	-	-	0.064	-	-	-	-	-	-
115.916	32.871	> 0.001	17	-	0.075	-	-	-	0.160	-	-0.203	-0.019	-	-	-	-	-	-	-0.072	-
115.922	32.877	> 0.001	17	-0.025	0.060	-	-	-	0.157	-	-0.205	-	-	-	-	-	-	-	-0.066	-
115.953	32.909	> 0.001	6	-0.009	-	-	-	-	0.156	-	-	-0.075	-	-	-	-	-	-	-	-
115.959	32.915	> 0.001	6	0.014	-	-	-	-	0.172	-	-	-	-	0.072	-	-	-	-0.104	-	-
115.975	32.930	> 0.001	9	-	-	-	-	-	-	-	-0.115	-	-	0.047	-	-	-	-	-	-
116.020	32.975	> 0.001	9	0.043	-	-	-	-	0.150	-	-0.068	-	-	0.071	-	-	-	-0.106	-	-
116.055	33.011	> 0.001	9	0.052	-	-	-	-	0.138	-	-0.073	-	-	0.086	-	-	-	-	-	-
116.083	33.038	> 0.001	12	0.000	0.125	-	-	-	-	-0.169	-0.016	-	-	-0.311	-	-	-	-	-	-
116.089	33.044	> 0.001	13	-	-0.083	-	-	-	0.141	-	0.004	-	-	-	-	-	-	-	-	-
116.098	33.053	> 0.001	11	-	-	-	-	-	-	-	-0.125	0.034	-	0.172	-	-	-	-	-	-
116.181	33.136	> 0.001	7	0.046	-	-	-	-	0.176	-	-	-0.078	-	0.082	-	-	-	-0.130	-	-
116.207	33.163	> 0.001	7	-0.029	-	-	-	-	0.166	-	-	-0.086	-	0.086	-	-	-	-	-	-
116.232	33.188	> 0.001	10	-	-	-	-	-	0.198	-	-0.048	-	-	0.059	-	-	-	-	-	-
116.293	33.249	> 0.001	11	0.003	-0.060	-	-	-	0.142	-0.203	-	-	-	-	-	-	-	-	-	-
116.324	33.279	> 0.001	5	0.024	-	-	-	-	-	-	-	-0.074	-	0.074	-	-	-	-	-	-
116.413	33.369	> 0.001	7	0.047	-	-	-	-	0.254	-	-	-0.042	-	-	-	-	-	-0.136	-	-
116.455	33.410	> 0.001	8	0.043	-	-	-	-	-	-	-0.080	-0.045	-	0.071	-	-	-	-	-	-
116.489	33.444	> 0.001	9	0.051	-	-	-	-	0.225	-	-0.043	-	-	-	-	-	-	-	-	-

116.525	33.480	> 0.001	12	-0.052	0.123	-	-	-	-	-	-0.027	-	-	-0.326	-	-	-	-	-	-
116.532	33.487	> 0.001	16	-	0.274	-	-	-	-	-	-0.230	-	-	-0.379	-	-	-	-	-0.027	-
116.532	33.487	> 0.001	16	-	0.274	-	-	-	-	-	-0.230	-	-	-0.379	-	-	-	-	-0.027	-
116.532	33.487	> 0.001	16	-	0.274	-	-	-	-	-	-0.230	-	-	-0.379	-	-	-	-	-0.027	-
116.545	33.500	> 0.001	7	-0.015	-	-	-	-	0.244	-	-	-0.046	-	-	-	-	-	-	-	-
116.646	33.601	> 0.001	9	0.077	-	-	-	-	0.151	-	-0.049	-0.045	-	-	-	-	-	-0.161	-	-
116.653	33.608	> 0.001	9	-0.019	-	-	-	-	0.137	-	-0.055	-0.054	-	-	-	-	-	-	-	-
116.734	33.689	> 0.001	10	-0.010	0.171	-	-	-	-	-0.161	-	-0.020	-	-0.363	-	-	-	-	-	-
116.746	33.701	> 0.001	18	-	0.093	-	-	-	0.260	-	-0.189	-	-	-	-	-	-	-	-0.080	-
116.797	33.752	> 0.001	9	0.055	-	-	-	-	0.170	-	-0.056	-0.032	-	-	-	-	-	-	-	-
116.829	33.784	> 0.001	7	-0.047	-	-	-	-	-	-	-	-0.025	-	0.211	-	-	-	-	-	-
116.833	33.788	> 0.001	9	-	-	-	-	-	0.212	-	-0.042	-0.020	-	-	-	-	-	-	-	-
116.833	33.788	> 0.001	9	-	-	-	-	-	0.212	-	-0.042	-0.020	-	-	-	-	-	-	-	-
116.865	33.820	> 0.001	7	0.055	-	-	-	-	-	-	-	-0.022	-	0.213	-	-	-	-	-	-
116.868	33.823	> 0.001	7	0.047	-	-	-	-	0.129	-	-	-	-	0.078	-	-	-	-	-	-
116.872	33.827	> 0.001	8	0.049	-	-	-	-	-	-	-0.093	-	-	0.079	-	-	-	-	-	-
116.889	33.845	> 0.001	11	-	-	-	-	-	0.106	-	-0.096	-	-	0.043	-	-	-	-	-	-
116.941	33.896	> 0.001	12	0.012	-0.072	-	-	-	-	-0.182	-0.017	-0.008	-	-	-	-	-	-	-	-
116.960	33.915	> 0.001	11	0.050	-	-	-	-	0.133	-	-0.090	-	-	0.069	-	-	-	-	-	-
117.041	33.996	> 0.001	11	-	-	-	-	-	0.133	-	-0.081	-0.033	-	0.075	-	-	-	-	-	-
117.070	34.025	> 0.001	10	-	-	-	-	-	0.217	-	-0.041	-0.034	-	0.086	-	-	-	-	-	-
117.132	34.087	> 0.001	10	0.039	-	-	-	-	0.232	-	-0.045	-	-	0.075	-	-	-	-	-	-
117.193	34.148	> 0.001	5	0.045	-	-	-	-	-	-	-	-	-	0.093	-	-	-	-	-	-
117.194	34.149	> 0.001	7	0.018	-	-	-	-	0.138	-	-	-	-	0.054	-	-	-	-0.078	-	-
117.224	34.179	> 0.001	7	0.068	-	-	-	-	0.173	-	-	-	-	0.100	-	-	-	-0.153	-	-
117.279	34.234	> 0.001	11	-0.035	-0.054	-	-	-	-	-0.083	-	0.029	-	-	-	-	-	-	-	-
117.379	34.334	> 0.001	9	0.022	-	-	-	-	0.134	-	-0.067	-	-	-	-	-	-	-	-	-
117.435	34.390	> 0.001	8	-0.010	-	-	-	-	-	-	-0.076	-0.047	-	-	-	-	-	-	-	-
117.464	34.419	> 0.001	12	-0.052	-0.091	-	-	-	-	-	-0.031	0.008	-	-	-	-	-	-	-	-
117.473	34.428	> 0.001	6	0.079	-	-	-	-	-	-	-	-0.077	-	0.106	-	-	-	-	-	-
117.536	34.491	> 0.001	5	0.002	-	-	-	-	-	-	-	-0.071	-	-	-	-	-	-	-	-
117.585	34.540	> 0.001	13	-0.041	-0.072	-	-	-	-	-0.345	-0.035	-	-	-	-	-	-	-	-	-
117.605	34.560	> 0.001	10	0.082	-0.064	-	-	-	-	-0.213	-	-0.019	-	-	-	-	-	-	-	-
117.637	34.593	> 0.001	10	0.045	-	-	-	-	-	-	-0.114	-	-	0.053	-	-	-	-	-	-
117.679	34.634	> 0.001	7	0.019	-	-	-	-	0.171	-	-	-0.075	-	-	-	-	-	-0.142	-	-
117.708	34.663	> 0.001	16	-0.034	0.016	-	-	-	-	-	-0.230	-	-	-	-	-	-	-	-0.020	-
117.712	34.667	> 0.001	10	-	-	-	-	-	-	-	-0.106	-0.029	-	0.058	-	-	-	-	-	-
117.847	34.802	> 0.001	18	-	0.054	-	-	-	0.135	-	-0.209	-	-	-	-	-	-	-	-0.063	-
117.857	34.812	> 0.001	16	-	0.027	-	-	-	-	-	-0.229	-0.010	-	-	-	-	-	-	-0.023	-
117.959	34.914	> 0.001	8	-0.008	-	-	-	-	-	-	-0.092	-	-	-	-	-	-	-	-	-
118.143	35.098	> 0.001	10	0.066	-	-	-	-	0.214	-	-0.033	-0.037	-	-	-	-	-	-	-	-
118.162	35.117	> 0.001	10	0.040	-	-	-	-	0.140	-	-0.070	-	-	0.073	-	-	-	-	-	-
118.199	35.154	> 0.001	9	-0.025	-	-	-	-	-	-	-0.078	-0.055	-	0.072	-	-	-	-	-	-
118.231	35.186	> 0.001	9	0.070	-	-	-	-	-	-	-0.080	-0.048	-	0.088	-	-	-	-	-	-
118.236	35.191	> 0.001	12	-	-	-	-	-	0.228	-	-0.062	-	-	0.074	-	-	-	-	-	-
118.241	35.196	> 0.001	6	-0.015	-	-	-	-	-	-	-	-0.080	-	0.075	-	-	-	-	-	-
118.271	35.226	> 0.001	17	-0.040	0.276	-	-	-	-	-	-0.230	-	-	-0.391	-	-	-	-	-0.025	-
118.452	35.407	> 0.001	10	0.055	-	-	-	-	0.225	-	-0.042	-	-	-	-	-	-	-0.057	-	-
118.526	35.482	> 0.001	17	-	0.275	-	-	-	-	-	-0.230	-0.004	-	-0.377	-	-	-	-	-0.027	-
118.770	35.726	> 0.001	18	-	0.036	-	-	-	-	-	-0.233	0.022	-	-	-	-	-	-	-0.021	-

118.820	35.775	> 0.001	10	0.034	-	-	-	-	0.137	-	-0.054	-0.044	-	-	-	-	-	-	-	-
119.001	35.956	> 0.001	9	0.007	-	-	-	-	-	-	-0.096	-	-	0.065	-	-	-	-	-	-
119.135	36.090	> 0.001	11	0.062	-	-	-	-	-	-	-0.101	-0.042	-	0.070	-	-	-	-	-	-
119.175	36.130	> 0.001	10	0.025	-	-	-	-	0.145	-	-0.065	-	-	-	-	-	-	-0.132	-	-
119.377	36.332	> 0.001	7	0.036	-	-	-	-	-	-	-	-0.084	-	0.107	-	-	-	-	-	-
119.529	36.484	> 0.001	9	0.002	-	-	-	-	-	-	-0.081	-0.039	-	-	-	-	-	-	-	-
119.580	36.535	> 0.001	11	0.057	-	-	-	-	-	-	-0.114	-	-	0.054	-	-	-	-	-	-
119.707	36.662	> 0.001	17	-0.033	0.017	-	-	-	-	-	-0.230	-0.002	-	-	-	-	-	-	-0.020	-
120.429	37.384	> 0.001	10	0.019	-	-	-	-	-	-	-0.084	-0.045	-	0.070	-	-	-	-	-	-
120.472	37.428	> 0.001	11	0.042	-	-	-	-	0.224	-	-0.043	-	-	-	-	-	-	-	-	-
120.638	37.593	> 0.001	18	0.011	0.024	-	-	-	-	-	-0.245	-	-	-	-	-	-	-	-0.020	-
120.803	37.759	> 0.001	10	0.010	-	-	-	-	-	-	-0.097	-	-	0.079	-	-	-	-	-	-
121.301	38.256	> 0.001	18	-0.016	0.019	-	-	-	-	-0.133	-0.230	-	-	-	-	-	-	-	-0.010	-
121.320	38.275	> 0.001	12	0.003	-	-	-	-	-	-	-0.132	-	-	0.048	-	-	-	-	-	-
121.399	38.354	> 0.001	10	-0.032	-	-	-	-	-	-	-0.079	-0.046	-	-	-	-	-	-	-	-

Loc.*Resp. Sex	Resp. Sex*Don. Sex	Resp. Sex*Resp. Age	Don. Sex*Don. Age	Don. Sex*Resp. Rep.	Loc.*Resp. Age	Resp. Sex*Don. Age	Loc.*Don. Age	Resp. Age*Don. Sex
-	-	-	-	-	-	-	-	-
-	-	-	-	-	-	-	-	-
-	-	-	-	-	-	-	-	-
-	-	-	-	-	-	-	-	-
-	-	-	-	-	-	-	-	-
-	-	-	-	-	-	-	-	-
-	-	-	-	-	-	-	-	-
-	-	-	-	-	-	-	-	-
-	-	-	-	-	-	-	-	-
-	-	-	-	-	-	-	-	-
-	-	-	-	-	-	-	-	-
-	-	-	-	-	-	-	-	-
-	-	-	-	-	-	-	-	-
-	-	-	-	-	-	-	-	-
-	-	-	-	-	-	-	-	-
-	-	-	-	-	-	-	-	-
-	-	-	-	-	-	-	-	-
-	-	-	-	-	-	-	-	-
-	-	-	-	-	-	-	-	-
-	-	-	-	-	-	-	-	-
-	-	-	-	-	-	-	-	-
-	-	-	-	-	-	-	-	-
-	-	-	-	-	-	-	-	-
-	-	-	-	-	-	-	-	-
-	-	-	-	-	-	-	-	-
-	-	-	-	-	-	-	-	-
-	-	-	-	-	-	-	-	-
-	-	-	-	-	-	-	-	-
-	-	-	-	-	-	-	-	-
-	-	-	-	-	-	-	-	-
-	-	-	-	-	-	-	-	-
-	-	-	-	-	-	-	-	-
-	-	-	-	-	-	-	-	-
-	-	-	-	-	-	-	-	-
-	-	-	-	-	-	-	-	-
-	-	-	-	-	-	-	-	-
-	-	-	-	-	-	-	-	-
-	-	-	-	-	-	-	-	-
-	-	-	-	-	-	-	-	-
-	-	-	-	-	-	-	-	-
-	-	-	-	-	-	-	-	-
-	-	-	-	-	-	-	-	-
-	-	-	-	-	-	-	-	-
-	-	-	-	-	-	-	-	-
-	-	-	-	-	-	-	-	-
-	-	-	-	-	-	-	-	-

-	-	-	-	-	-	-	-	-
-	-	-	-	-	-	-	-	-
-	-	-	-	-	-	-	-	-
-	-	-	-	-	-	-	-	-
-	-	-	-	-	-	-	-	-
-	-	-	-	-	-	-	-	-
-	-	-	-	-	-	-	-	-
-	-	-	-	-	-	-	-	-
-	-	-	-	-	-	-	-	-
-	-	-	-	-	-	-	-	-
-	-	-	-	-	-	-	-	-
-	-	-	-	-	-	-	-	-
-	-	-	-	-	-	-	-	-
-	-	-	-	-	-	-	-	-
-	-	-	-	-	-	-	-	-
-	-	-	-	-	-	-	-	-
-	-	-	-	-	-	-	-	-
-	-	-	-	-	-	-	-	-
-	-	-	-	-	-	-	-	-
-	-	-	-	-	-	-	-	-
-	-	-	-	-	-	-	-	-
-	-	-	-	-	-	-	-	-
-	-	-	-	-	-	-	-	-
-	-	-	-	-	-	-	-	-
-0.017	-	-	-	-	-	-	-	-
-	-	-	-	-	-	-	-	-
-	-0.204	-	-	-	-	-	-	-
-	-	-	-	-	-	-	-	-
-	-	-	-	-	-	-	-	-
-	-	-	-	-	-	-	-	-
-	-	-	-	-	-	-	-	-
-	-	-0.053	-	-	-	-	-	-
-	-	-	-	-	-	-	-	-
-	-	-	-0.151	-	-	-	-	-
-	-	-	-	-	-	-	-	-
-	-	-	-	-	-	-	-	-
-	-	-	-	-	-	-	-	-
-	-	-	-	-0.114	-	-	-	-
-	-	-	-	-	-0.209	-	-	-
-	-	-	-	-0.102	-	-	-	-
-	-	-	-	-	-	-	-	-
-	-	-	-	-	-	-	-	-
-	-	-	-	-	-	-	-	-
-	-	-	-	-	-	-	-	-
-	-	-	-	-	-	-	-	-
-	-	-	-	-	-	-	-	-
-	-	-	-	-0.036	-	-	-	-
-	-	-	-	-	-	-	-	-
-	-	-	-	-	-	-	-	-
-	-	-	-	-	-	-	-	-

-	-	-	-	-	-	-	-	-
-	-	-	-	-	-	-	-	-
-	-	-	-	-	-	-	-	-
-	-	-	-	-	-	-	-	-
-	-	-	-	-	-	-	-	-
-	-	-	-	-	-	-	-	-
-	-	-	-	-	-	-	-	-
-	-	-	-	-	-	0.098	-	-
-	-	-	-	-	-	-	-	-
-	-	-	-	-	-	-	-	-
-	-	-	-	-	-	-	-	-
-	-	-	-	-	-	-	-	-
-	-	-	-	-	-	-	-	-
-	-	-	-	-	-	-	-	-
-	-	-	-	-	-	-	-	-
-	-	-	-	-	-	-	-	-
-	-	-	-	-	-	-	-	-
-	-	-	-	-0.110	-	-	-	-
-	-	-	-	-	-	-	-	-
-	-	-	-	-	-	-	-	-
-	-	-	-	-	-	-	-	-
-	-	-	-	-	-	-	-	-
-	-	-	-	-	-	-	-	-
-	-	-	-	-	-	-	-	-
-	-	-	-	-	-	-	-	-
-	-	-	-	-	-	-	-	-
-	-	-	-	-	-	-	-0.199	-
-	-	-	-	-	-	-	-	-
-	-	-	-	-	-	-	-	-
-	-	-	-	-0.143	-	-	-	-
-	-	-	-	-	-	-	-	-
-	-	-	-	-	-	-	-	-
-	-	-	-	-	-	-	-	-
-	-	-	-	-	-	-	-	-
-	-	-	-	-	-	-	-	-
-	-	-	-	-	-	-	-	-
-	-	-	-	-	-	-	-	-
-	-0.218	-	-	-	-	-	-	-
-	-	-	-	-	-	-	-	-
-	-	-	-	-	-	-	-	-
-	-	-	-	-	-	-	-	-
-	-	-	-	-	-	-	-	-
-	-	-	-	-	-	-	-	-
-	-0.197	-	-	-	-	-	-	-
-	-	-	-	-	-	-	-	0.147
-	-	-	-	-	-	-	-	-
-	-0.186	-	-	-	-	-	-	-
-	-	-	-	-	-	-	-	-
-	-	-	-	-	-	-	-	-
0.228	-	-	-	-	-	-	-	-

-	-	-	-	-0.114	-	-	-	-
-	-	-	-	-	-	0.112	-	-
-	-	-	-	-	-	-	-	-
-	-	-	-	-	-	-	-	-
-	-	-	-	-	-	-	-	-
-	-	-	-	-	-	-	-	-
-	-	-	-	-	-	-	-	-
-	-	-	-	-	-	-	-	-
-	-	-	-	-	-	-	-	-
-	-	-0.076	-	-	-	-	-	-
-	-	-	-	-	-	-	-	-
-	-	-	-	-	-	-	-	-
-	-	-	-	-0.099	-	-	-	-
-	-	-	-	-	-	-	-	-
-	-	-	-	-	-	-	-	-
-	-	-	-	-	-	-	-	-
-	-	-	-	-	-	-	-	-
-	-	-	-	-	-	0.053	-	-
-	-	-	-	-	-	-	-	-
-	-	-	-	-	-	-	-	-
-	-0.197	-	-	-	-	-	-	-
-	-	-	-	-	-	-	-	-
-	-	-	-	-	-	-	-	-
-	-	-	-	-	-	-	-	-
-	-0.203	-	-	-	-	-	-	-
-	-	-	-	-	-	-	-	-
-	-	-	-	-	-	-	-	-
-	-	-	-	-	-	-	-	-
-	-	-	-	-	-	-	-	-
-	-	-	-	-	0.026	-	-	-
-	-	-	-0.178	-	-	-	-	-
-	-	-	-	-	-	-	-	-
-	-	-0.091	-	-	-	-	-	-
-	-	-	-	-	-	0.128	-	-
-	-	-	-	-	-	-	-	-
-	-	-	-	-	-	-	-	-0.013
-	-	-	-	-	-	-	-	-
-	-	-	-	-	-	-	-	-
-0.038	-	-	-	-	-	-	-	-
-	-	-	-	-	-	-	-	-
-	-	-0.101	-	-	-	-	-	-
-	-	-	-	-	-	-	-	-
-	-	-	-	-	-	-	-	-
-	-	-	-0.202	-	-	-	-	-
-	-	-	-	-	-	-	-	-
-	-	-	-	-	-	-	-	-
-	-0.150	-	-	-	-	-	-	-
-	-	-	-	-	-	-	-	-
-	-	-	-	-	-	-	-	-
-	-	-	-	-	-	-	-	-

-	-	-	-	-	-	-	-	-
-	-	-	-	-	-	0.109	-	-
-0.021	-	-	-	-	-	-	-	-
-	-	-	-	-	-	-	-	-
-	-	-	-	-	-	-	-	-
-	-	-	-	-	-	-	-0.116	-
-	-	-0.049	-	-	-	-	-	-
-	-	-0.054	-	-	-	-	-	-
-	-	-	-	-	-	-	-	-
-	-	-	-	-	-	-	-	-
-	-	-	-	-	-	-	-	-
-	-	-	-	-	-	-	-	-
-	-	-	-	-	-	-	-	-
-0.035	-	-	-	-	-	-	-	-
-	-	-	-	-	-	-	-	-
-	-	-	-	-	-	-	-	-
-	-	-	-	-	-	-	-	-
-	-	-	-	-	-	-	-	-
-	-	-	-0.183	-	-	-	-	-
-	-	-	-	-	-	0.108	-	-
-	-	-	-	-	-	-	-	-
-	-	-	-	-	-	-	-	-
-	-	-	-	-	-	-	-	-
-	-	-	-	-	-	-	-	-
-0.031	-	-	-	-	-	-	-	-
-	-	-	-	-	-	-	-0.177	-
-	-	-	-	-	-	-	-	-
-	-	-	-	-	-	-	-	-
-	-	-	-	-	-	-	-	-
-	-	-	-	-	-	-	-	-
-	-	-	-	-	-	-	-	-
-	-	-	-	-	-	-	-	-
-	-	-	-	-	-	-	-	-
-	-	-	-	-	-	-	-	-
-	-	-	-0.202	-	-	-	-	-
-	-	-	-0.223	-	-	-	-	-
-	-	-	-	-	-0.049	-	-	-
-	-	-0.106	-	-	-	-	-	-
-	-	-	-	-	-	-	-	-
-	-	-	-	-	-	-	-	-
-	-	-	-	-	-	-	-0.195	-
-	-	-	-	-	-	-	-	0.084
-	-	-	-	-	-	-	-0.168	-
-	-	-	-	-	-	-	-	0.064
-	-	-	-	-	-	-	-	0.073
-	-	-	-	-	-	-	-	-
-0.017	-	-	-	-	-	-	-	-
-	-	-	-	-	-	-	-	-
-	-	-	-	-	-	-	-	-
-	-	-	-	-	-	-	-	0.093

-	-	-	-	-	-	-	-	-
-	-	-	-	-	-	-	-	-
-	-	-	-	-	-	-	-	-
-	-	-	-	-	-0.074	-	-	-
-	-	-	-	-	-0.054	-	-	-
-	-	-	-	-	-	-	-	-
-	-	-	-	-	-	0.125	-	-
-	-	-	-	-	-	-	-	-
-	-	-	-	-	-	-	-	0.084
-	-	-	-	-	-	-	-	-
-	-	-	-	-	-0.089	-	-	-
-	-	-	-	-	-	-	-0.182	-
-	-	-	-	-	-	-	-	-
-	-	-	-	-	-	-	-	-
-	-	-	-	-	-	-	-	-
-	-	-	-0.251	-	-	-	-	-
-	-	-	-	-	-	-	-	-
-	-	-	-	-	-	-	-	-
-	-	-	-	-	-	-	-	-
-	-	-	-	-	-	-	-0.388	-
-	-	-	-	-	-	-	-	-
-	-	-	-	-	-0.055	-	-	-
-	-	-	-	-	-	-	-	-
-	-	-	-	-	-	-	-	-
-	-	-	-	-	-	-	-	-
-	-	-	-	-	-	-	-	-
-	-	-	-	-	-	-	-	-
-	-	-	-	-	-	-	-	-
-	-	-	-	-0.149	-	-	-	-
-	-	-	-	-	-	-	-	-
-	-	-	-	-	-	-	-	-
-	-	-	-	-	-	-	-	-
-	-	-	-	-	-	-	-	-
-	-	-	-	-	-	-	-	-
-	-	-	-	-	-	-	-	-
-	-	-	-0.104	-	-	-	-	-
-	-	-	-	-	-	-	-	-
-	-0.282	-	-	-	-	-	-	-
-	-	-	-	-	-	-	-0.077	-
-	-	-	-	-	-	-	-	-
-	-	-	-	-	-	-	-	-
-	-	-	-	-	-	-	-	-
-	-	-	-	-	-	-	-	-
-	-	-	-	-	-	-	-	-
-	-	-	-	-	-	-	-	-
-	-	-	-	-	-	-	-	-
-	-0.246	-	-	-	-	-	-	-
-	-	-	-	-	-	-	-	-
-	-	-	-	-	-	-	-	-
-	-	-	-	-	-	-	-	-

-	-	-	-	-	-	-	-	-
-	-	-	-	-	-	-	-	-
-	-	-	-	-	-	-	-	-
-	-0.261	-	-	-	-	-	-	-
-	-0.261	-	-	-	-	-	-	-
-	-	-	-	-	-	0.197	-	-
-	-	-	-	-	-	0.197	-	-
-	-	-	-	-	-	-	-	-
-	-	-	-	-	-	-	-	-
-	-	-	-	-	-	-	-	-
-	-	-	-	-	-	-	-	-
-	-	-	-	-	-	-	-	-
-	-	-	-	-	-	-	-	-
-	-0.271	-	-	-	-	-	-	-
-	-	-	-	-0.122	-	-	-	-
-	-	-	-	-	-	-	-	-
-	-	-	-	-	-	-	-	-
-	-	-	-	-	-	-	-	0.125
-	-	-	-	-	-	-	-	-
-	-	-	-	-	-	-	-	-
-0.069	-	-	-	-	-	-	-	-
-	-	-	-	-	-	-	-	-
-	-	-	-0.144	-	-	-	-	-
-	-	-	-	-	-	-	-	-
-	-	-	-	-	-	-	-	-
-	-	-	-	-	-	-	-	-
-	-	-	-	-	-	-	-	-
-	-	-	-	-	-	-	-	-
-	-	-	-	-	-	-	-	-
-	-	-	-	-	-	-	-	-
-	-	-	-	-0.139	-	-	-	-
-	-	-	-	-	-	-	-	-
-	-	-	-	-0.111	-	-	-	-
-	-0.216	-	-	-	-	-	-	-
-	-	-	-	-0.156	-	-	-	-
-	-	-	-	-	-	-	-	-
-	-	-	-	-	-	-	-	-
-	-0.255	-	-	-	-	-	-	-
-	-	-	-	-	-	-	-	-
-	-	-	-0.161	-0.131	-	-	-	-
-	-	-	-	-	-	0.173	-	-
-	-	-	-	-0.149	-	-	-	-
-	-	-	-	-	-	0.179	-	-
-	-	-	-	-	-	-	-	-
-	-0.231	-	-0.211	-	-	-	-	-
-	-	-	-	-	-	-	-	-
-	-	-	-	-	-	-	-	-
-	-0.246	-	-	-	-	-	-	-
-	-	-	-0.148	-	-	-	-	-
-	-	-	-	-	-	-	-	-

-	-	-	-	-	-	-	-	-
-	-	-	-	-	-	0.189	-	-
-	-	-	-	-	-	-	-	-
-	-	-	-	-0.130	-	-	-	-
-	-	-	-	-0.130	-	-	-	-
-	-	-	-	-0.130	-	-	-	-
-	-	-	-	-	-	-	-	-
-	-	-0.088	-	-	-	-	-	-
-	-	-	-	-	-	-	-	-
-	-	-	-	-	-	-	-	-
-	-	-	-	-	-	-	-	-
-	-0.264	-	-	-	-	-	-	-
-	-	-	-	-	-	-	-	-
-	-	-	-	-	-	-	-	-
-	-	-	-	-	0.123	-	-	-
-	-	-	-	-	-	-	-	-
-	-	-	-	-	-	-	-	-
-	-	-	-	-	-	-	-	-
-	-	-	-	-	-	-	-	-
-	-	-	-	-	-	-	-	-
-	-	-	-	-	-	-	-	-
-	-	-	-	-0.137	-	-	-	-
-	-	-	-	-	-	-	-	-
-	-	-	-	-	-	-	-	-
-	-	-	-0.205	-	-	-	-	-
-	-	-	-	-0.166	-	-	-	-
-	-	-	-0.152	-	-	-	-	-
-0.066	-	-	-	-	-	-	-	-
-	-	-	-	-	-	-	-	-
-	-	-	-	-	-	-	-	-
-	-	-0.100	-	-	-	-	-	-
-	-	-0.100	-	-	-	-	-	-
-	-	-	-	-	-	-	-0.102	-
-	-	-	-	-	-	-	-	-
-0.050	-	-	-	-	-	-	-	-
-0.050	-	-	-	-	-	-	-	-
-	-	-	-	-	-	-	-	-
-	-0.234	-	-	-	-	-	-	-
-	-	-	-	-	-	-	-	-
-	-	-	-	-	-	-	-	-
-	-	-	-	-	-	-	-	-
-	-	-	-	-	-	0.202	-	-
-	-	-	-0.186	-	-	-	-	-
-	-	-	-0.039	-	-	-	-	-
-	-	-	-	-	-	-	-	-
-	-0.206	-	-	-	-	0.136	-	-
-	-	-	-	-	-	-	-	-
-	-	-	-	-	-	-	-	-
-	-	-	-	-0.130	-	-	-	-
-	-	-	-	-	-	-	-	-

-	-	-	-	-	-	-	-	-
-	-	-	-	-	-	0.193	-	-
-	-	-	-	-	-	-	-	-
-	-0.216	-	-	-	-	-	-	-
-	-	-	-	-	-	-	-	-
-	-	-	-	-	-	-	-	-0.017
-	-	-	-	-	-	-	-	-
-	-	-	-	-	-	-	-	-
-	-	-	-	-	-	-	-	-
-	-	-	-	-0.157	-	-	-	-
-	-	-	-	-	-	-	-	-
-	-	-	-	-0.162	-	-	-	-
-	-	-	-	-	-	-	-	-
-	-	-	-	-	-	0.179	-	-
-	-	-	-	-	-	-	-	-
-	-	-	-	-	-	-	-	-
-	-	-	-	-	-	-	-	-
-	-	-	-	-	-	-	-	-
-	-	-	-	-	-	-	-	-
-	-	-	-	-	-	-	-	-
-	-	-	-	-	-	-	-	-
-	-	-	-	-	-	-	-	-
-	-	-	-	-	-	-	-	-
-	-	-	-	-	-	-	-	-
-	-0.265	-	-	-	-	-	-	-
-	-	-	-	-	-	-	-	-
-	-	-	-	-	-	-	-	-
-	-	-	-	-0.112	-	-	-	-
-	-	-	-	-	-	-	-0.100	-
-	-	-	-	-	-	-	-	-
-	-	-	-	-	-	-	-	-
-	-	-0.056	-	-	-	-	-	-
-	-	-	-	-	-	-	-	-0.052
-	-	-	-0.197	-	-	-	-	-
-	-	-	-0.135	-	-	-	-	-
-	-	-	-	-	-	-	-	-
-	-	-	-	-	-	-	-	-
-	-	-	-	-	-	-	-	-
-	-	-	-	-	-	-	-	-
-	-	-	-0.178	-	-	0.163	-	-
-	-	-	-	-	-	-	-	-
-	-	-	-	-	-	-	-	-
-	-	-	-	-	-	-	-	-
-	-	-	-	-	-	-	-	-
-	-	-	-	-	-	-	-	-
-	-	-	-	-	-	-	-0.169	-
-	-	-	-	-	-	-	-	-
-	-	-	-	-	-	-	-	-
-	-	-	-	-	-	-	-	-
-	-	-	-	-	-	-	-0.093	-

-	-	-	-	-	-	-	-	-
-	-	-0.102	-	-	-	-	-	-
-0.048	-	-	-	-	-	-	-	-
-	-0.234	-	-	-	-	-	-	-
-	-	-	-	-	-	-	-0.100	-
-0.048	-	-	-	-	-	-	-	-
-	-	-	-	-	-	-	-	-
-	-	-	-	-	-	-	-	-
-	-	-	-	-	-	0.180	-	-
-	-	-	-0.181	-	-	-	-	-
-	-	-	-	-	-	-	-	-
-	-	-	-	-	0.009	-	-	-
-	-	-	-	-	-	0.205	-	-
-	-	-	-0.098	-	-	-	-	-
-	-	-	-	-	-	-	-	-0.066
-	-	-	-	-	-	0.202	-	-
-0.052	-	-	-	-	-	-	-	-
-	-	-	-	-	-	-	-0.178	-
-	-	-	-	-	-	-	-	-
-	-	-	-	-0.130	-	-	-	-0.002
-	-	-	-	-	0.061	-	-	-
-	-	-0.119	-	-	-	-	-	-
-	-	-	-	-	-	-	-	-
-	-	-	-	-	-	-	-	-
-	-	-	-0.078	-	-	-	-	-
-	-	-	-	-	-	-	-	-0.010
-	-	-0.132	-	-	-	-	-	-
-	-	-	-	-	-	0.186	-	-
-	-	-	-	-	-	-	-	-
-	-	-	-	-	-	-	-	-
-	-	-	-	-	-	-	-	-
-	-	-	-	-	-	-	-	-
-	-	-	-	-	-	-	-	-
-	-	-	-	-	-	-	-	-
-	-	-	-	-	-	-	-	0.071
-	-	-	-	-	-	-	-	-
-0.037	-	-	-	-	-	-	-	-
-	-	-	-	-	-	-	-	-
-	-	-0.055	-	-	-	-	-	-
-	-	-	-	-	-	-	-	-
-	-	-	-	-	-	-	-0.197	-
0.007	-0.247	-	-	-	-	-	-	-
-	-	-	-	-	-	-	-	-
-	-	-	-	-	-	-	-	0.083
-	-	-	-	-	-	0.248	-0.224	-
-	-	-	-	-	-	-	-	-
-	-	-	-	-	-	-	-	-
-	-	-	-0.191	-	-	-	-	-
-	-	-	-	-	-	-	-	-
-	-	-	-	-	-	-	-0.183	-

-	-	-	-	-	0.060	-	-	-
-	-	-	-	-	-	-	-	-
-	-	-	-	-	-	-	-	-
-	-	-	-	-	-	-	-	-
-	-	-	-0.196	-	-	-	-	0.065
-	-	-	-	-	-	-	-	-
-	-	-	-	-	-	-	-	0.107
-	-	-	-	-	-	-	-	-
-	-	-	-	-	-	-	-0.212	-
-	-	-	-0.079	-	-	-	-	-
-	-0.243	-	-	-	-	-	-	0.095
-	-	-	-	-	-	-	-0.163	-
-	-	-	-	-	-	-	-0.163	-
-	-0.218	-0.079	-	-	-	-	-	-
-	-	-0.101	-	-	-	0.185	-	-
-	-	-0.063	-	-	-	-	-	-
-0.043	-	-	-	-	-	0.155	-	-
-	-	-	-	-	-	-	-	-
-0.060	-	-	-	-	-	-	-	-
-0.057	-	-	-	-	-	-	-	-
-	-	-	-	-	-	-	-0.168	-
-	-	-	-	-	-	-	-0.187	-
-	-	-0.133	-	-	-	-	-	-
-	-	-	-	-	-	0.197	-	-
-	-	-0.138	-	-	-	-	-	-
-	-	-	-	-0.124	-	-	-	-
-	-	-	-	-	-0.005	-	-	-
-	-	-	-	-	-	-	-	0.084
-	-	-	-	-	0.061	-	-	-
-	-	-0.146	-	-	-	-	-	-
-	-	-	-	-	-	-	-	0.043
-	-	-	-	-	0.227	-	-	-
-	-	-	-	-	-	-	-	-0.100
-0.044	-	-	-	-	-	-	-	-
-	-	-	-	-	-	-	-	0.052
-	-	-	-	-	-	-	-	-
-0.042	-	-	-	-	-	-	-	-
-	-	-	-	-	-	-	-	-
-	-	-	-	-	-	-	-	-
-	-	-	-	-	0.049	-	-	-
-	-	-	-	-	-	-	-0.175	-
-	-	-	-	-	-0.021	-	-	-
-	-	-	-	-	-	-	-	0.091
-	-	-0.076	-	-	-	-	-	-
-0.062	-	-	-	-	-	-	-0.196	-
-	-	-	-	-	-	-	-	0.053
-	-	-	-	-	-	-	-	-
-	-	-	-	-	-	-	-0.167	-
-	-	-	-	-	-	-	-	-
-	-	-	-	-0.086	-	-	-	-

-	-	-	-	-	-0.007	-	-	-
-	-	-	-	-	0.037	-	-	-
-0.053	-	-	-	-	-	-	-	-
-	-	-	-	-	0.027	-	-	-
-	-	-0.147	-	-	-	-	-	0.057
-	-	-	-	-	0.048	-	-	-
-0.040	-	-0.041	-	-	-	-	-	-
-	-	-	-	-	-	-	-	-
-	-	-	-	-	0.035	-	-	-
-	-	-	-	-	-0.003	-	-0.175	-
-	-	-	-	-	0.046	-	-	-
-	-	-0.076	-	-	0.055	-	-	-
-	-	-	-	-	-	-	-	-
-0.030	-	-	-	-	0.107	-	-	-
-	-	-	-	-	0.034	-	-	0.071

Table S3:

Statistical summary of the models linking the duration of sniffing responses (sec.) to a global model that included the variables; ‘age’ of the donor and responder (levels: yearling, adult); ‘sex’ of the donor and the responder (levels: male, female); ‘reproductive status of the female’ donor and responder (levels: oestrous, non-oestrous); and ‘reproductive status of the male’ donor and responder (levels: descended, fully descended), as well as interaction terms, as factors in these models. Responder and trial ID were included as random effects in these models. This table is the basis of the model averaging, for which results are presented in Table 2b of the main text.

The support for each model, based on Akaike criterion, is presented in the first three columns. The fourth column presents the degrees of freedom associated with each model. Subsequent columns present coefficient estimates of the parameters included in each model.
