# Supplementary material for: Will Trespassers Be Prosecuted or Assessed According to Their Merits? A Consilient Interpretation of Territoriality in a Group-Living Carnivore, the European Badger (Meles meles)
Source: PLoS One. 2015 Jul 6;10(7):e0132432. doi: 10.1371/journal.pone.0132432 (PMC4493095; doi:10.1371/journal.pone.0132432)
Supplement: S4 Table — The global model included the variables; ‘age’ of the donor and responder (levels: yearling, adult); ‘sex’ of the donor and the responder (levels: male, female); ‘reproductive status of the female’ donor and responder (levels: oestrous, non-oestrous); and ‘reproductive status of the male’ donor and responder (levels: descended, fully descended), as well as interaction terms, as factors in these models. Responder and trial ID were included as random effects in these models. This table is the basis of the model averaging, for which results are presented in Table 4 of the main text. The support for each model, based on Akaike criterion, is presented in the first three columns. The fourth column presents the degrees of freedom associated with each model. Subsequent columns present coefficient estimates of the parameters included in each model. (PDF) [file pone.0132432.s004.pdf]

Table S4

AIC	ΔAIC	*w*	d.f.	Loc.	Resp. Rep.	Don. Sex	Fam.	Loc.*Don. Sex	Resp. Sex	Loc.*Resp. Sex	Resp. Age	Resp. Age*Resp. Rep.	Fam.*Loc.	Don. Age	Fam.*Don. Sex	Loc.*Resp. Rep.	Fam.*Resp. Sex	Resp. Sex*Resp. Age	Resp. Age*Don. Sex
305.373	0	0.160	14	0.192	-0.235	0.153	0.089	0.150	-	-	-	-	-	-	-	-	-	-	-
306.560	1.187	0.089	14	0.343	-0.275	-	0.070	-	0.467	-0.293	-	-	-	-	-	-	-	-	-
306.763	1.390	0.080	13	0.424	-0.058	-	0.116	-	-	-	0.108	-0.870	-	-	-	-	-	-	-
308.088	2.715	0.041	10	0.347	-0.138	-	0.069	-	-	-	-	-	-	-	-	-	-	-	-
308.738	3.366	0.030	11	0.371	-0.161	-	0.084	-	-	-	-0.173	-	-	-	-	-	-	-	-
309.275	3.902	0.023	12	0.337	-0.214	0.141	0.067	-	-	-	-0.236	-	-	-	-	-	-	-	-
309.369	3.996	0.022	7	0.233	-	-	0.057	-	-	-	-	-	-	-	-	-	-	-	-
309.524	4.151	0.020	11	0.322	-0.164	0.083	0.056	-	-	-	-	-	-	-	-	-	-	-	-
309.729	4.356	0.018	11	0.420	-0.127	-	0.162	-	-	-	-	-	-0.159	-	-	-	-	-	-
309.784	4.411	0.018	8	0.228	-	-	0.058	-	0.152	-	-	-	-	-	-	-	-	-	-
309.958	4.585	0.016	11	0.235	-	-	0.056	-	0.451	-0.382	-	-	-	-	-	-	-	-	-
310.047	4.674	0.015	11	0.349	-0.056	-	0.071	-	-0.128	-	-	-	-	-	-	-	-	-	-
310.084	4.711	0.015	11	0.349	-0.138	-	0.070	-	-	-	-	-	-	0.009	-	-	-	-	-
310.351	4.978	0.013	12	0.446	-0.150	-	0.180	-	-	-	-0.175	-	-0.164	-	-	-	-	-	-
310.463	5.090	0.013	13	0.340	-0.174	0.309	0.218	-	-	-	-	-	-	-	-0.305	-	-	-	-
310.625	5.252	0.012	17	0.307	-0.009	-	0.056	-	-	-	-	-	-	-	-	-0.016	-	-	-
310.644	5.271	0.011	12	0.375	-0.036	-	0.087	-	-0.195	-	-0.177	-	-	-	-	-	-	-	-
310.717	5.344	0.011	13	0.084	-	-	-0.086	-	-0.238	0.172	-	-	-	-	-	-	0.632	-	-
310.738	5.365	0.011	12	0.371	-0.161	-	0.084	-	-	-	-0.173	-	-	0.001	-	-	-	-	-
310.833	5.461	0.010	8	0.328	-	-	0.170	-	-	-	-	-	-0.195	-	-	-	-	-	-
310.998	5.625	0.010	10	0.245	-	-	0.018	-	0.026	-	-	-	-	-	-	-	0.236	-	-
311.060	5.687	0.009	12	0.402	-0.154	0.091	0.160	-	-	-	-	-	-0.181	-	-	-	-	-	-
311.084	5.712	0.009	13	0.371	-0.121	-	0.043	-	-0.133	-	-	-	-	-	-	-	0.192	-	-
311.087	5.715	0.009	9	0.335	-	-	0.186	-	0.160	-	-	-	-0.221	-	-	-	-	-	-
311.223	5.850	0.009	10	0.257	-	-	0.089	-	0.262	-	0.108	-	-	-	-	-	-	-0.468	-
311.239	5.866	0.009	11	0.092	-	0.035	0.073	0.155	-	-	-	-	-	-	-	-	-	-	-
311.256	5.883	0.008	8	0.218	-	0.037	0.050	-	-	-	-	-	-	-	-	-	-	-	-
311.299	5.926	0.008	10	0.202	-	-0.030	0.025	-	-	-	-0.546	-	-	-	-	-	-	-	0.672
311.331	5.959	0.008	8	0.234	-	-	0.059	-	-	-	-0.028	-	-	-	-	-	-	-	-
311.363	5.990	0.008	8	0.236	-	-	0.058	-	-	-	-	-	-	0.012	-	-	-	-	-
311.428	6.055	0.008	12	0.328	-	-	0.166	-	0.452	-0.379	-	-	-0.190	-	-	-	-	-	-
311.454	6.081	0.008	12	0.324	-0.056	0.086	0.058	-	-0.168	-	-	-	-	-	-	-	-	-	-
311.459	6.087	0.008	12	0.087	-	-0.012	0.076	0.185	0.161	-	-	-	-	-	-	-	-	-	-
311.524	6.151	0.007	12	0.322	-0.164	0.083	0.056	-	-	-	-	-	-	0.000	-	-	-	-	-
311.644	6.271	0.007	9	0.229	-	-	0.062	-	0.158	-	-0.054	-	-	-	-	-	-	-	-
311.705	6.332	0.007	12	0.420	-0.064	-	0.161	-	-0.098	-	-	-	-0.156	-	-	-	-	-	-
311.721	6.348	0.007	12	0.423	-0.126	-	0.164	-	-	-	-	-	-0.160	0.014	-	-	-	-	-
311.751	6.378	0.007	9	0.235	-	-	0.061	-	0.154	-	-	-	-	0.029	-	-	-	-	-
311.759	6.386	0.007	9	0.221	-	0.017	0.055	-	0.149	-	-	-	-	-	-	-	-	-	-
311.915	6.542	0.006	12	0.233	-	-	0.054	-	0.454	-0.386	0.031	-	-	-	-	-	-	-	-
311.925	6.552	0.006	18	0.283	-0.055	0.091	0.041	-	-	-	-	-	-	-	-	0.003	-	-	-
311.937	6.564	0.006	12	0.241	-	-	0.058	-	0.453	-0.384	-	-	-	0.022	-	-	-	-	-
311.950	6.577	0.006	12	0.230	-	0.010	0.054	-	0.447	-0.380	-	-	-	-	-	-	-	-	-
311.997	6.624	0.006	10	0.240	-	-	0.018	-	-	-	0.058	-	-	0.109	-	-	-	-	-
312.044	6.671	0.006	12	0.351	-0.055	-	0.072	-	-0.127	-	-	-	-	0.008	-	-	-	-	-
312.114	6.741	0.006	13	0.290	-0.141	0.220	0.036	-	-	-	-	-	-	-	-	-	-	-	-

312.246	6.873	0.005	11	0.356	-	-	0.148	-	0.026	-	-	-	-0.228	-	-	-	0.247	-	-
312.332	6.960	0.005	18	0.383	-0.004	-	0.136	-	-	-	-	-	-0.139	-	-	-0.017	-	-	-
312.466	7.093	0.005	18	0.310	0.154	-	0.059	-	-0.245	-	-	-	-	-	-	-0.021	-	-	-
312.481	7.108	0.005	18	0.312	-0.018	-	0.061	-	-	-	-0.057	-	-	-	-	-0.015	-	-	-
312.559	7.186	0.004	13	0.373	-0.162	-	0.094	-	-	-	-0.115	-	-	-	-	-	-	-	-
312.624	7.251	0.004	18	0.306	-0.010	-	0.055	-	-	-	-	-	-	-0.004	-	-0.015	-	-	-
312.649	7.276	0.004	9	0.315	-	0.047	0.170	-	-	-	-	-	-0.209	-	-	-	-	-	-
312.697	7.324	0.004	13	0.365	-0.161	-	0.085	-	-	-	-0.192	-	-	-	-	-	-	-	-
312.779	7.406	0.004	9	0.330	-	-	0.174	-	-	-	-0.034	-	-0.198	-	-	-	-	-	-
312.808	7.435	0.004	11	0.246	-	-	0.019	-	0.022	-	-0.063	-	-	-	-	-	0.251	-	-
312.818	7.445	0.004	12	0.174	-	0.036	0.170	0.159	-	-	-	-	-0.171	-	-	-	-	-	-
312.821	7.448	0.004	9	0.333	-	-	0.172	-	-	-	-	-	-0.196	0.017	-	-	-	-	-
312.850	7.477	0.004	12	0.122	-	0.064	0.088	0.127	-	-	-	-	-	0.102	-	-	-	-	-
312.902	7.529	0.004	10	0.341	-	-	0.195	-	0.167	-	-0.062	-	-0.229	-	-	-	-	-	-
312.943	7.570	0.004	11	0.254	-	-	0.021	-	0.025	-	-	-	-	0.036	-	-	0.241	-	-
312.976	7.603	0.004	11	0.238	-	0.016	0.016	-	0.025	-	-	-	-	-	-	-	0.234	-	-
313.022	7.649	0.004	10	0.328	-	0.028	0.186	-	0.156	-	-	-	-0.229	-	-	-	-	-	-
313.036	7.663	0.003	10	0.345	-	-	0.192	-	0.162	-	-	-	-0.225	0.035	-	-	-	-	-
313.065	7.692	0.003	13	0.351	-0.115	-	0.057	-	-	-	-	-	-	0.093	-	-	-	-	-
313.139	7.766	0.003	10	0.234	-	-	0.051	-	0.183	-	-	-	-	0.080	-	-	-	-	-
313.172	7.799	0.003	9	0.216	-	0.045	0.052	-	-	-	-0.043	-	-	-	-	-	-	-	-
313.239	7.866	0.003	12	0.092	-	0.035	0.073	0.155	-	-	-0.001	-	-	-	-	-	-	-	-
313.252	7.879	0.003	9	0.221	-	0.036	0.052	-	-	-	-	-	-	0.010	-	-	-	-	-
313.326	7.953	0.003	9	0.236	-	-	0.060	-	-	-	-0.027	-	-	0.011	-	-	-	-	-
313.382	8.009	0.003	13	0.371	-0.122	-	0.058	-	-	-	-	-	-	0.157	-	-	-	-	-
313.445	8.072	0.003	10	0.229	-	0.196	0.168	-	-	-	-	-	-	-	-0.215	-	-	-	-
313.578	8.205	0.003	10	0.218	-	0.029	0.057	-	0.155	-	-0.063	-	-	-	-	-	-	-	-
313.613	8.241	0.003	11	0.232	-	0.189	0.183	-	0.165	-	-	-	-	-	-0.234	-	-	-	-
313.615	8.242	0.003	10	0.236	-	-	0.065	-	0.160	-	-0.053	-	-	0.027	-	-	-	-	-
313.647	8.274	0.003	10	0.218	-	0.038	0.056	-	0.196	-	-	-	-	-	-	-	-	-	-
313.711	8.338	0.002	13	0.333	-0.129	-	0.040	-	-	-	-	-	-	-0.122	-	-	-	-	-
313.729	8.356	0.002	10	0.228	-	0.016	0.058	-	0.151	-	-	-	-	0.027	-	-	-	-	-
314.071	8.698	0.002	10	0.202	-	0.078	0.020	-	-	-	-	-	-	0.175	-	-	-	-	-
314.074	8.701	0.002	10	0.267	-	-	0.038	-	-	-	-	-	-	0.218	-	-	-	-	-
314.268	8.895	0.002	16	0.334	-0.209	-	-0.008	-	-	-	-	-	-	-	-	-	-	-	-
314.417	9.044	0.002	13	0.034	-	-0.140	0.028	0.307	-	-	-	-	-	-	0.137	-	-	-	-
314.441	9.068	0.002	11	0.269	-	-	0.042	-	0.155	-	-	-	-	0.234	-	-	-	-	-
314.518	9.145	0.002	10	0.317	-	0.058	0.177	-	-	-	-0.054	-	-0.217	-	-	-	-	-	-
314.640	9.267	0.002	10	0.320	-	0.047	0.172	-	-	-	-	-	-0.210	0.014	-	-	-	-	-
314.768	9.395	0.001	10	0.335	-	-	0.176	-	-	-	-0.033	-	-0.199	0.016	-	-	-	-	-
315.001	9.628	0.001	10	0.222	-	-	0.024	-	-	-	-	-	-	-0.131	-	-	-	-	-
315.014	9.641	0.001	17	0.354	-0.232	-	0.010	-	-	-	-0.169	-	-	-	-	-	-	-	-
315.143	9.770	0.001	10	0.227	-	-	0.054	-	-	-	-0.125	-	-	-	-	-	-	-	-
315.169	9.797	0.001	10	0.218	-	0.045	0.053	-	-	-	-0.043	-	-	0.007	-	-	-	-	-
315.207	9.834	0.001	11	0.290	-	0.196	0.239	-	-	-	-	-	-0.135	-	-0.207	-	-	-	-
315.228	9.855	0.001	11	0.218	-	-	0.021	-	0.162	-	-	-	-	-0.139	-	-	-	-	-
315.268	9.895	0.001	10	0.225	-	-	0.048	-	-	-	-0.070	-	-	-	-	-	-	-	-
315.358	9.985	0.001	11	0.380	-	-	0.168	-	-	-	-	-	-0.225	0.238	-	-	-	-	-
315.421	10.049	0.001	11	0.227	-	0.197	0.167	-	-	-	-0.023	-	-	-	-0.211	-	-	-	-
315.444	10.071	0.001	11	0.230	-	0.195	0.169	-	-	-	-	-	-	0.006	-0.214	-	-	-	-

315.526	10.153	0.001	17	0.310	-0.244	0.095	-0.030	-	-	-	-	-	-	-	-	-	-	-	-
315.603	10.230	0.001	11	0.226	-	-	0.060	-	0.159	-	-0.051	-	-	-	-	-	-	-	-
315.631	10.258	0.001	11	0.226	-	-	0.060	-	0.155	-	-0.084	-	-	-	-	-	-	-	-
315.666	10.293	0.001	11	0.234	-	0.074	0.020	-	-	-	-	-	-	0.241	-	-	-	-	-
315.960	10.587	0.001	17	0.401	-0.209	-	0.067	-	-	-	-	-	-0.149	-	-	-	-	-	-
315.965	10.592	0.001	22	0.285	-0.372	-	-0.125	-	-	-	-	-	-	-	-	0.095	-	-	-
316.053	10.681	0.001	11	0.266	-	-	0.039	-	-	-	-0.021	-	-	0.216	-	-	-	-	-
316.221	10.849	0.001	17	0.337	-0.118	-	-0.003	-	-0.136	-	-	-	-	-	-	-	-	-	-
316.265	10.892	0.001	17	0.336	-0.210	-	-0.008	-	-	-	-	-	-	0.008	-	-	-	-	-
316.436	11.063	0.001	11	0.322	-	-	0.140	-	-	-	-	-	-0.202	-0.131	-	-	-	-	-
316.450	11.077	0.001	11	0.330	-	-	0.184	-	-	-	-0.186	-	-0.226	-	-	-	-	-	-
316.557	11.184	0.001	10	-	-0.025	-	-0.172	-	-	-	0.079	-0.607	-	-	-	-	-	-	-
316.757	11.384	0.001	11	0.323	-	-	0.163	-	-	-	-0.070	-	-0.194	-	-	-	-	-	-
316.849	11.476	0.001	11	0.203	-	0.043	0.014	-	-	-	-	-	-	-0.143	-	-	-	-	-
316.977	11.604	> 0.001	7	-	-0.111	-	-0.198	-	-	-	-	-	-	-	-	-	-	-	-
316.993	11.620	> 0.001	11	0.223	-	-	0.026	-	-	-	-0.013	-	-	-0.128	-	-	-	-	-
317.015	11.642	> 0.001	11	0.211	-	0.041	0.049	-	-	-	-0.133	-	-	-	-	-	-	-	-
317.076	11.703	> 0.001	10	-	0.085	-	-0.160	-	-0.189	-	0.030	-	-	-	-	-	-	-0.639	-
317.118	11.745	> 0.001	11	0.209	-	0.044	0.043	-	-	-	-0.078	-	-	-	-	-	-	-	-
317.135	11.762	> 0.001	11	0.231	-	-	0.056	-	-	-	-0.122	-	-	0.014	-	-	-	-	-
317.257	11.884	> 0.001	11	0.228	-	-	0.049	-	-	-	-0.074	-	-	0.017	-	-	-	-	-
317.337	11.964	> 0.001	8	-	-0.136	-	-0.191	-	-	-	-0.198	-	-	-	-	-	-	-	-
317.818	12.445	> 0.001	11	-	-0.071	0.104	-0.172	-	-	-	0.012	-0.533	-	-	-	-	-	-	-
317.854	12.482	> 0.001	12	0.278	-	-	0.046	-	-	-	-	-	-	0.314	-	-	-	-	-
318.112	12.739	> 0.001	9	-	-0.188	0.133	-0.193	-	-	-	-0.262	-	-	-	-	-	-	-	-
318.240	12.867	> 0.001	7	-	-	-	-0.129	-	0.336	-	0.151	-	-	-	-	-	-	-0.621	-
318.486	13.113	> 0.001	11	-	0.087	-	-0.170	-	-0.173	-	0.074	-0.602	-	-	-	-	-	-	-
318.537	13.164	> 0.001	5	-	-	-	-0.161	-	0.192	-	-	-	-	-	-	-	-	-	-
318.551	13.178	> 0.001	11	-	-0.024	-	-0.172	-	-	-	0.080	-0.613	-	0.012	-	-	-	-	-
318.643	13.270	> 0.001	8	-	-0.133	0.066	-0.200	-	-	-	-	-	-	-	-	-	-	-	-
318.658	13.285	> 0.001	11	-	-0.196	0.389	-0.020	-	-	-	-0.237	-	-	-	-0.345	-	-	-	-
318.764	13.391	> 0.001	10	-	-0.148	0.347	-0.013	-	-	-	-	-	-	-	-0.371	-	-	-	-
318.842	13.470	> 0.001	10	-	-0.159	0.077	-0.204	-	-	-	-0.548	-	-	-	-	-	-	-	0.399
318.856	13.483	> 0.001	4	-	-	-	-0.170	-	-	-	-	-	-	-	-	-	-	-	-
318.910	13.538	> 0.001	8	-	0.000	-	-0.197	-	-0.172	-	-	-	-	-	-	-	-	-	-
318.972	13.599	> 0.001	8	-	-0.110	-	-0.197	-	-	-	-	-	-	0.011	-	-	-	-	-
319.087	13.715	> 0.001	12	0.216	-	-	0.046	-	-	-	-0.165	-	-	-	-	-	-	-	-
319.208	13.835	> 0.001	9	-	0.018	-	-0.190	-	-0.238	-	-0.202	-	-	-	-	-	-	-	-
319.337	13.964	> 0.001	9	-	-0.136	-	-0.191	-	-	-	-0.198	-	-	-0.002	-	-	-	-	-
319.518	14.145	> 0.001	10	-	-0.139	0.139	-0.224	-	-	-	-	-	-	0.264	-	-	-	-	-
319.592	14.219	> 0.001	11	-	-0.173	0.361	-0.202	-	-	-	-0.366	-	-	-	-	-	-	-	-
319.851	14.478	> 0.001	10	-	0.029	0.141	-0.191	-	-0.340	-	-0.272	-	-	-	-	-	-	-	-
320.103	14.730	> 0.001	10	-	-0.190	0.134	-0.194	-	-	-	-0.264	-	-	-0.015	-	-	-	-	-
320.157	14.784	> 0.001	10	-	-0.095	-	-0.207	-	-0.060	-	-	-	-	-	-	-	0.084	-	-
320.208	14.835	> 0.001	7	-	-	-	-0.172	-	0.169	-	-	-	-	-	-	-	0.092	-	-
320.215	14.842	> 0.001	8	-	-	-	-0.129	-	0.337	-	0.151	-	-	0.025	-	-	-	-0.619	-
320.240	14.867	> 0.001	8	-	-	-0.001	-0.129	-	0.336	-	0.151	-	-	-	-	-	-	-0.621	-
320.309	14.937	> 0.001	10	-	-0.133	-	-0.218	-	-	-	-0.140	-	-	0.055	-	-	-	-	-
320.320	14.947	> 0.001	6	-	-	-	-0.156	-	0.201	-	-0.070	-	-	-	-	-	-	-	-
320.459	15.086	> 0.001	12	-	-0.024	-	-0.160	-	-	-	0.162	-0.638	-	-	-	-	-	-	-

320.462	15.089	> 0.001	9	-	-	-	-0.172	-	0.225	-	0.138	-	-	-	-	-	0.199	-0.596	-
320.468	15.095	> 0.001	6	-	-	-	-0.161	-	0.195	-	-	-	-	0.042	-	-	-	-	-
320.481	15.109	> 0.001	19	0.023	0.002	0.069	-	0.335	-	-	-	-	-	-	-	-0.324	-	-	-
320.499	15.126	> 0.001	11	-	-0.065	-	-0.212	-	-0.175	-	-0.200	-	-	-	-	-	0.132	-	-
320.537	15.164	> 0.001	6	-	-	0.000	-0.161	-	0.192	-	-	-	-	-	-	-	-	-	-
320.544	15.171	> 0.001	9	-	0.002	0.069	-0.199	-	-0.210	-	-	-	-	-	-	-	-	-	-
320.641	15.268	> 0.001	9	-	-0.132	0.065	-0.200	-	-	-	-	-	-	0.007	-	-	-	-	-
320.736	15.363	> 0.001	11	-	-0.076	0.347	-0.013	-	-0.111	-	-	-	-	-	-0.369	-	-	-	-
320.759	15.386	> 0.001	11	-	-0.149	0.348	-0.012	-	-	-	-	-	-	-0.011	-0.373	-	-	-	-
320.799	15.426	> 0.001	5	-	-	-	-0.168	-	-	-	-0.036	-	-	-	-	-	-	-	-
320.808	15.436	> 0.001	5	-	-	0.024	-0.169	-	-	-	-	-	-	-	-	-	-	-	-
320.837	15.464	> 0.001	5	-	-	-	-0.170	-	-	-	-	-	-	0.023	-	-	-	-	-
320.844	15.471	> 0.001	7	-	-	0.106	-0.197	-	-	-	-	-	-	0.316	-	-	-	-	-
320.907	15.534	> 0.001	9	-	0.000	-	-0.196	-	-0.170	-	-	-	-	0.010	-	-	-	-	-
321.045	15.672	> 0.001	8	-	-	0.079	-0.188	-	0.171	-	-	-	-	0.313	-	-	-	-	-
321.175	15.802	> 0.001	10	-	-0.135	-	-0.177	-	-	-	-0.117	-	-	-	-	-	-	-	-
321.207	15.834	> 0.001	10	-	0.018	-	-0.190	-	-0.239	-	-0.203	-	-	-0.004	-	-	-	-	-
321.541	16.168	> 0.001	8	-	-	-0.056	-0.177	-	0.207	-	-0.520	-	-	-	-	-	-	-	0.591
321.693	16.320	> 0.001	11	-	-0.097	0.077	-0.205	-	-0.086	-	-	-	-	-	-	-	0.069	-	-
321.738	16.365	> 0.001	8	-	-	0.220	-0.006	-	0.210	-	-	-	-	-	-0.295	-	-	-	-
321.800	16.427	> 0.001	9	-	-	0.331	-0.047	-	-	-	-	-	-	0.331	-0.286	-	-	-	-
321.854	16.481	> 0.001	12	-	-0.126	0.484	-0.008	-	-	-	-	-	-	-	-0.398	-	-	-	-
321.869	16.496	> 0.001	10	-	-0.111	0.176	-0.205	-	-	-	-	-	-	-	-	-	-	-	-
321.892	16.519	> 0.001	11	-	-0.188	0.137	-0.175	-	-	-	-0.155	-	-	-	-	-	-	-	-
322.021	16.648	> 0.001	8	-	-	-	-0.172	-	0.166	-	-0.065	-	-	-	-	-	0.107	-	-
322.090	16.717	> 0.001	7	-	-	-	-0.169	-	0.219	-	-	-	-	0.085	-	-	-	-	-
322.143	16.771	> 0.001	8	-	-	-	-0.173	-	0.168	-	-	-	-	0.041	-	-	0.097	-	-
322.156	16.783	> 0.001	11	-	-0.094	-	-0.207	-	-0.060	-	-	-	-	0.007	-	-	0.085	-	-
322.190	16.817	> 0.001	7	-	-	-0.033	-0.187	-	-	-	-0.475	-	-	-	-	-	-	-	0.569
322.205	16.832	> 0.001	8	-	-	0.006	-0.171	-	0.169	-	-	-	-	-	-	-	0.091	-	-
322.227	16.854	> 0.001	9	-	-	-	-0.134	-	0.336	-	0.124	-	-	-	-	-	-	-0.627	-
322.259	16.886	> 0.001	7	-	-	-	-0.156	-	0.203	-	-0.069	-	-	0.040	-	-	-	-	-
322.304	16.931	> 0.001	7	-	-	0.014	-0.156	-	0.199	-	-0.075	-	-	-	-	-	-	-	-
322.348	16.975	> 0.001	10	-	0.006	-	-0.207	-	-0.156	-	-	-	-	0.061	-	-	-	-	-
322.381	17.008	> 0.001	10	-	-0.091	-	-0.210	-	-	-	-	-	-	0.083	-	-	-	-	-
322.406	17.033	> 0.001	10	-	-0.102	-	-0.230	-	-	-	-	-	-	-0.160	-	-	-	-	-
322.424	17.051	> 0.001	7	-	-	0.021	-0.158	-	0.240	-	-	-	-	-	-	-	-	-	-
322.468	17.095	> 0.001	7	-	-	-0.001	-0.161	-	0.195	-	-	-	-	0.042	-	-	-	-	-
322.478	17.105	> 0.001	7	-	-	0.228	-0.028	-	-	-	-	-	-	-	-0.270	-	-	-	-
322.523	17.150	> 0.001	8	-	-	-	-0.193	-	0.185	-	-0.001	-	-	0.112	-	-	-	-	-
322.536	17.163	> 0.001	10	-	-0.001	0.074	-0.198	-	-0.189	-	-	-	-	-	-	-	-	-	-
322.543	17.170	> 0.001	11	-	-0.122	-	-0.203	-	-	-	-0.213	-	-	0.053	-	-	-	-	-
322.543	17.170	> 0.001	10	-	0.002	0.069	-0.199	-	-0.209	-	-	-	-	0.005	-	-	-	-	-
322.570	17.197	> 0.001	8	-	-	0.125	-0.193	-	-	-	-0.081	-	-	0.320	-	-	-	-	-
322.628	17.255	> 0.001	7	-	-	-	-0.208	-	-	-	0.038	-	-	0.104	-	-	-	-	-
322.713	17.340	> 0.001	6	-	-	0.034	-0.167	-	-	-	-0.048	-	-	-	-	-	-	-	-
322.783	17.410	> 0.001	6	-	-	-	-0.168	-	-	-	-0.035	-	-	0.021	-	-	-	-	-
322.790	17.417	> 0.001	6	-	-	0.024	-0.169	-	-	-	-	-	-	0.022	-	-	-	-	-
323.030	17.657	> 0.001	11	-	0.028	-	-0.175	-	-0.253	-	-0.119	-	-	-	-	-	-	-	-
323.081	17.708	> 0.001	11	-	-0.129	-	-0.213	-	-	-	-0.182	-	-	-0.113	-	-	-	-	-

323.155	17.782	> 0.001	13	-	-0.167	-	-0.287	-	-	-	-	-	-	-	-	-	-	-	-
323.171	17.798	> 0.001	11	-	-0.136	-	-0.177	-	-	-	-0.114	-	-	-0.010	-	-	-	-	-
323.369	17.996	> 0.001	16	-	-0.120	-	-0.289	-	-	-	0.074	-0.592	-	-	-	-	-	-	-
323.423	18.050	> 0.001	9	-	-	-	-0.175	-	0.229	-	-	-	-	0.099	-	-	0.063	-	-
323.461	18.088	> 0.001	9	-	-	0.166	-0.043	-	-	-	-0.481	-	-	-	-0.277	-	-	-	0.618
323.616	18.243	> 0.001	9	-	-	0.245	0.000	-	0.260	-	-	-	-	-	-0.300	-	-	-	-
323.651	18.278	> 0.001	9	-	-	0.223	-0.007	-	0.214	-	-0.046	-	-	-	-0.286	-	-	-	-
323.682	18.309	> 0.001	11	-	0.078	0.189	-0.203	-	-0.290	-	-	-	-	-	-	-	-	-	-
323.691	18.318	> 0.001	14	-	-0.197	-	-0.283	-	-	-	-0.189	-	-	-	-	-	-	-	-
323.694	18.322	> 0.001	9	-	-	0.216	-0.007	-	0.212	-	-	-	-	0.034	-0.290	-	-	-	-
323.748	18.375	> 0.001	8	-	-	-	-0.200	-	0.205	-	-	-	-	-0.162	-	-	-	-	-
323.764	18.391	> 0.001	13	0.054	-0.359	0.247	-	0.076	-	-	-0.283	-	-	-	-	-	-	-	-
323.834	18.461	> 0.001	8	-	-	-	-0.165	-	0.230	-	-0.076	-	-	0.085	-	-	-	-	-
323.849	18.476	> 0.001	11	-	-0.109	0.177	-0.204	-	-	-	-	-	-	0.023	-	-	-	-	-
323.859	18.486	> 0.001	10	-	-	0.222	-0.029	-	0.159	-	-	-	-	-	-0.296	-	0.123	-	-
323.962	18.589	> 0.001	9	-	-	-	-0.173	-	0.165	-	-0.064	-	-	0.039	-	-	0.112	-	-
323.994	18.621	> 0.001	9	-	-	0.019	-0.171	-	0.165	-	-0.071	-	-	-	-	-	0.105	-	-
324.012	18.639	> 0.001	11	-	-0.125	0.072	-0.235	-	-	-	-	-	-	-0.178	-	-	-	-	-
324.073	18.700	> 0.001	11	-	-0.111	0.064	-0.213	-	-	-	-	-	-	0.085	-	-	-	-	-
324.087	18.714	> 0.001	8	-	-	-0.006	-0.169	-	0.220	-	-	-	-	0.086	-	-	-	-	-
324.127	18.754	> 0.001	8	-	-	-0.034	-0.187	-	-	-	-0.479	-	-	0.041	-	-	-	-	0.576
324.141	18.769	> 0.001	9	-	-	0.005	-0.173	-	0.168	-	-	-	-	0.041	-	-	0.097	-	-
324.190	18.817	> 0.001	8	-	-	0.035	-0.153	-	0.248	-	-0.075	-	-	-	-	-	-	-	-
324.205	18.832	> 0.001	9	-	-	0.005	-0.171	-	0.167	-	-	-	-	-	-	-	0.090	-	-
324.224	18.851	> 0.001	15	-	-0.259	0.144	-0.292	-	-	-	-0.257	-	-	-	-	-	-	-	-
324.246	18.873	> 0.001	8	-	-	0.013	-0.156	-	0.201	-	-0.073	-	-	0.039	-	-	-	-	-
324.273	18.900	> 0.001	8	-	-	-	-0.147	-	0.204	-	-0.013	-	-	-	-	-	-	-	-
324.328	18.955	> 0.001	11	-	0.008	-	-0.209	-	-0.153	-	-	-	-	0.080	-	-	-	-	-
324.354	18.981	> 0.001	7	-	-	-	-0.203	-	-	-	-	-	-	-0.149	-	-	-	-	-
324.354	18.982	> 0.001	11	-	-0.004	-	-0.228	-	-0.151	-	-	-	-	-0.159	-	-	-	-	-
324.367	18.994	> 0.001	8	-	-	0.019	-0.158	-	0.241	-	-	-	-	0.039	-	-	-	-	-
324.371	18.998	> 0.001	9	-	-	0.111	-0.207	-	-	-	-	-	-	0.287	-	-	-	-	-
324.463	19.090	> 0.001	8	-	-	0.229	-0.028	-	-	-	-0.019	-	-	-	-0.267	-	-	-	-
324.473	19.100	> 0.001	8	-	-	0.227	-0.029	-	-	-	-	-	-	0.012	-0.269	-	-	-	-
324.543	19.170	> 0.001	16	-	-0.302	0.380	-0.160	-	-	-	-	-	-	-	-0.389	-	-	-	-
324.601	19.228	> 0.001	8	-	-	0.019	-0.207	-	-	-	0.030	-	-	0.102	-	-	-	-	-
324.640	19.267	> 0.001	14	-	-0.196	0.081	-0.293	-	-	-	-	-	-	-	-	-	-	-	-
324.698	19.326	> 0.001	7	-	-	0.034	-0.166	-	-	-	-0.047	-	-	0.019	-	-	-	-	-
324.735	19.362	> 0.001	12	0.027	-0.308	0.193	-	0.084	-	-	-	-	-	-	-	-	-	-	-
324.794	19.421	> 0.001	7	-	-	-	-0.167	-	-	-	-0.032	-	-	-	-	-	-	-	-
325.012	19.639	> 0.001	13	0.060	-0.300	0.251	-	0.029	-	-	-	-	-	0.219	-	-	-	-	-
325.071	19.699	> 0.001	14	-	-0.040	-	-0.283	-	-0.190	-	-	-	-	-	-	-	-	-	-
325.145	19.772	> 0.001	14	-	-0.168	-	-0.289	-	-	-	-	-	-	0.016	-	-	-	-	-
325.450	20.077	> 0.001	12	0.200	-0.225	-	-	-	0.325	-0.325	-	-	-	-	-	-	-	-	-
325.460	20.087	> 0.001	10	-	-	-	-0.213	-	0.175	-	-	-	-	-0.160	-	-	0.099	-	-
325.529	20.156	> 0.001	15	-	-0.021	-	-0.277	-	-0.264	-	-0.194	-	-	-	-	-	-	-	-
325.616	20.243	> 0.001	9	-	-	-	-0.200	-	0.219	-	-	-	-	-0.106	-	-	-	-	-
325.651	20.279	> 0.001	9	-	-	-	-0.194	-	0.210	-	-0.047	-	-	-0.150	-	-	-	-	-
325.689	20.316	> 0.001	15	-	-0.197	-	-0.284	-	-	-	-0.188	-	-	0.008	-	-	-	-	-
325.744	20.371	> 0.001	9	-	-	0.006	-0.200	-	0.204	-	-	-	-	-0.163	-	-	-	-	-

325.759	20.387	> 0.001	9	-	-	-	-0.172	-	-	-	0.379	-	-	0.102	-	-	-	-	-
325.934	20.561	> 0.001	10	-	-	-	-0.163	-	0.169	-	-0.016	-	-	-	-	-	0.109	-	-
325.999	20.626	> 0.001	9	-	-	-0.034	-0.169	-	-	-	-0.390	-	-	-	-	-	-	-	0.605
326.110	20.737	> 0.001	12	-	-0.092	-	-0.230	-	-	-	-	-	-	-0.079	-	-	-	-	-
326.227	20.854	> 0.001	9	-	-	-	-0.149	-	0.205	-	-0.024	-	-	0.035	-	-	-	-	-
326.231	20.858	> 0.001	13	0.213	-0.189	-	-	-	0.214	-0.305	-0.175	-	-	-	-	-	-	-	-
326.255	20.882	> 0.001	9	-	-	0.016	-0.146	-	0.202	-	-0.016	-	-	-	-	-	-	-	-
326.274	20.901	> 0.001	8	-	-	0.032	-0.204	-	-	-	-	-	-	-0.156	-	-	-	-	-
326.343	20.970	> 0.001	8	-	-	-	-0.201	-	-	-	-0.016	-	-	-0.145	-	-	-	-	-
326.458	21.085	> 0.001	9	-	-	0.228	-0.029	-	-	-	-0.019	-	-	0.011	-0.265	-	-	-	-
326.509	21.136	> 0.001	15	-	-0.038	0.085	-0.288	-	-0.239	-	-	-	-	-	-	-	-	-	-
326.513	21.140	> 0.001	13	0.030	-0.106	0.196	-	0.084	-0.312	-	-	-	-	-	-	-	-	-	-
326.623	21.250	> 0.001	9	-	-	-	-0.208	-	-	-	0.038	-	-	0.104	-	-	-	-	-
326.636	21.263	> 0.001	15	-	-0.196	0.080	-0.294	-	-	-	-	-	-	0.010	-	-	-	-	-
326.708	21.335	> 0.001	8	-	-	0.034	-0.165	-	-	-	-0.037	-	-	-	-	-	-	-	-
326.777	21.404	> 0.001	8	-	-	-	-0.168	-	-	-	-0.038	-	-	0.022	-	-	-	-	-
327.063	21.690	> 0.001	15	-	-0.042	-	-0.285	-	-0.188	-	-	-	-	0.014	-	-	-	-	-
327.347	21.974	> 0.001	9	0.216	-0.246	-	-	-	-	-	-0.280	-	-	-	-	-	-	-	-
327.387	22.014	> 0.001	13	0.207	-0.224	-	-	-	0.331	-0.330	-	-	-	0.040	-	-	-	-	-
327.391	22.018	> 0.001	13	0.193	-0.224	0.028	-	-	0.306	-0.318	-	-	-	-	-	-	-	-	-
327.449	22.076	> 0.001	14	-0.027	-0.272	0.330	-	0.141	-	-	-	-	-	-	-	-	-	-	-
327.551	22.178	> 0.001	16	-	-0.188	-	-0.269	-	-	-	-0.131	-	-	-	-	-	-	-	-
327.598	22.226	> 0.001	16	-	-0.181	0.173	-0.300	-	-	-	-	-	-	-	-	-	-	-	-
327.694	22.321	> 0.001	10	-	-	0.252	-0.061	-	-	-	-	-	-	-0.208	-0.290	-	-	-	-
327.774	22.401	> 0.001	11	0.232	-0.163	-	-	-	-	-	-0.047	-0.727	-	-	-	-	-	-	-
328.007	22.634	> 0.001	16	-	-0.133	-	-0.301	-	-	-	-	-	-	0.111	-	-	-	-	-
328.240	22.867	> 0.001	9	-	-	0.037	-0.201	-	-	-	-0.029	-	-	-0.150	-	-	-	-	-
328.376	23.003	> 0.001	10	-	-	0.232	-0.033	-	-	-	-0.067	-	-	-	-0.270	-	-	-	-
328.448	23.075	> 0.001	8	0.198	-0.214	-	-	-	-	-	-	-	-	-	-	-	-	-	-
328.537	23.164	> 0.001	12	0.183	-0.257	0.414	-	-	-	-	-0.456	-	-	-	-	-	-	-	-
328.543	23.170	> 0.001	13	0.161	-0.152	-	-	-	-	-	-0.466	-1.175	-	-	-	-	-	-	-
328.556	23.183	> 0.001	11	0.201	-0.243	0.023	-	-	-	-	-0.710	-	-	-	-	-	-	-	0.540
328.694	23.322	> 0.001	9	-	-	0.034	-0.166	-	-	-	-0.042	-	-	0.019	-	-	-	-	-
328.738	23.365	> 0.001	10	0.204	-0.284	0.098	-	-	-	-	-0.326	-	-	-	-	-	-	-	-
328.829	23.456	> 0.001	16	-	-0.160	-	-0.310	-	-	-	-	-	-	-0.116	-	-	-	-	-
328.862	23.489	> 0.001	10	0.220	0.060	-	-	-	-0.472	-	-0.287	-	-	-	-	-	-	-	-
328.902	23.530	> 0.001	15	0.220	0.035	-	-	-	-	-	-	-	-	-	-	-0.164	-	-	-
329.048	23.675	> 0.001	11	0.215	0.110	-	-	-	-0.449	-	-0.133	-	-	-	-	-	-	-0.439	-
329.260	23.887	> 0.001	10	0.222	-0.242	-	-	-	-	-	-0.277	-	-	0.048	-	-	-	-	-
329.379	24.006	> 0.001	12	0.235	0.109	-	-	-	-0.422	-	-0.057	-0.717	-	-	-	-	-	-	-
329.433	24.061	> 0.001	12	0.245	-0.153	-	-	-	-	-	-0.035	-0.779	-	0.097	-	-	-	-	-
329.607	24.234	> 0.001	12	0.224	-0.186	0.052	-	-	-	-	-0.081	-0.687	-	-	-	-	-	-	-
329.780	24.407	> 0.001	16	0.221	0.008	-	-	-	-	-	-0.169	-	-	-	-	-0.155	-	-	-
329.893	24.520	> 0.001	5	0.069	-	-	-	-	-	-	-	-	-	-	-	-	-	-	-
329.958	24.585	> 0.001	11	0.245	-0.238	-	-	-	-	-	-0.213	-	-	0.116	-	-	-	-	-
330.087	24.714	> 0.001	11	0.207	0.068	0.111	-	-	-0.551	-	-0.341	-	-	-	-	-	-	-	-
330.130	24.757	> 0.001	9	0.200	0.037	-	-	-	-0.386	-	-	-	-	-	-	-	-	-	-
330.204	24.832	> 0.001	16	0.220	0.401	-	-	-	-0.548	-	-	-	-	-	-	-0.171	-	-	-
330.221	24.848	> 0.001	11	-0.083	-	-0.020	-	0.107	-	-	-0.612	-	-	-	-	-	-	-	0.755
330.284	24.911	> 0.001	9	0.206	-0.208	-	-	-	-	-	-	-	-	0.067	-	-	-	-	-

330.326	24.953	> 0.001	10	-	-	-	-0.198	-	-	-	0.013	-	-	-0.152	-	-	-	-	-
330.436	25.063	> 0.001	9	0.196	-0.218	0.013	-	-	-	-	-	-	-	-	-	-	-	-	-
330.638	25.266	> 0.001	16	0.233	0.058	-	-	-	-	-	-	-	-	0.084	-	-0.189	-	-	-
330.678	25.305	> 0.001	9	-0.083	-	0.042	-	0.104	-	-	-	-	-	-	-	-	-	-	-
330.684	25.311	> 0.001	11	0.209	-0.280	0.095	-	-	-	-	-0.322	-	-	0.038	-	-	-	-	-
330.720	25.347	> 0.001	11	0.185	-0.254	-	-	-	-	-	-0.517	-	-	-	-	-	-	-	-
330.790	25.417	> 0.001	11	0.225	0.059	-	-	-	-0.465	-	-0.285	-	-	0.044	-	-	-	-	-
330.828	25.455	> 0.001	9	0.074	-	-	-	-	0.457	-0.419	-	-	-	-	-	-	-	-	-
330.895	25.522	> 0.001	16	0.219	0.030	0.010	-	-	-	-	-	-	-	-	-	-0.162	-	-	-
330.941	25.568	> 0.001	17	0.221	0.407	-	-	-	-0.600	-	-0.179	-	-	-	-	-0.163	-	-	-
331.249	25.876	> 0.001	10	-0.051	-	0.100	-	0.050	-	-	-	-	-	0.207	-	-	-	-	-
331.267	25.894	> 0.001	8	0.088	-	-0.106	-	-	-	-	-0.661	-	-	-	-	-	-	-	0.737
331.304	25.931	> 0.001	6	0.066	-	-	-	-	-	-	-0.118	-	-	-	-	-	-	-	-
331.324	25.951	> 0.001	6	0.064	-	-	-	-	0.099	-	-	-	-	-	-	-	-	-	-
331.398	26.025	> 0.001	13	-0.134	-	-0.102	-	0.279	0.494	-0.447	-	-	-	-	-	-	-	-	-
331.544	26.171	> 0.001	17	0.214	-0.025	0.059	-	-	-	-	-0.197	-	-	-	-	-0.144	-	-	-
331.570	26.197	> 0.001	17	0.232	0.029	-	-	-	-	-	-0.165	-	-	0.075	-	-0.177	-	-	-
331.660	26.287	> 0.001	6	0.081	-	-	-	-	-	-	-	-	-	0.081	-	-	-	-	-
331.742	26.369	> 0.001	6	0.081	-	-0.045	-	-	-	-	-	-	-	-	-	-	-	-	-
331.892	26.519	> 0.001	10	-0.091	-	0.008	-	0.125	0.115	-	-	-	-	-	-	-	-	-	-
331.959	26.587	> 0.001	17	0.233	0.418	-	-	-	-0.541	-	-	-	-	0.081	-	-0.195	-	-	-
331.979	26.606	> 0.001	10	0.208	0.037	-	-	-	-0.377	-	-	-	-	0.064	-	-	-	-	-
332.097	26.724	> 0.001	12	0.171	-0.291	0.099	-	-	-	-	-0.554	-	-	-	-	-	-	-	-
332.102	26.729	> 0.001	10	0.197	0.037	0.020	-	-	-0.397	-	-	-	-	-	-	-	-	-	-
332.176	26.803	> 0.001	18	0.258	0.134	-	-	-	-	-	-	-	-	0.236	-	-0.225	-	-	-
332.178	26.805	> 0.001	17	0.218	0.398	0.019	-	-	-0.558	-	-	-	-	-	-	-0.168	-	-	-
332.240	26.867	> 0.001	12	0.188	0.051	-	-	-	-0.470	-	-0.521	-	-	-	-	-	-	-	-
332.276	26.903	> 0.001	10	0.204	-0.212	0.011	-	-	-	-	-	-	-	0.066	-	-	-	-	-
332.277	26.904	> 0.001	11	-0.057	-	0.066	-	0.069	0.128	-	-	-	-	0.220	-	-	-	-	-
332.279	26.906	> 0.001	9	0.085	-	-0.121	-	-	0.130	-	-0.688	-	-	-	-	-	-	-	0.750
332.373	27.000	> 0.001	11	-0.027	-	0.143	-	0.030	-	-	-	-	-	0.361	-	-	-	-	-
332.377	27.004	> 0.001	18	0.220	0.020	-	-	-	-	-	-0.096	-0.504	-	-	-	-0.119	-	-	-
332.396	27.023	> 0.001	12	0.235	-0.227	-	-	-	-	-	-0.293	-	-	0.108	-	-	-	-	-
332.476	27.103	> 0.001	10	0.094	-	-0.069	-	-	0.482	-0.429	-	-	-	-	-	-	-	-	-
332.531	27.159	> 0.001	7	0.059	-	-	-	-	0.116	-	-0.139	-	-	-	-	-	-	-	-
332.538	27.165	> 0.001	18	0.277	0.132	-	-	-	-	-	-	-	-	0.355	-	-0.223	-	-	-
332.569	27.196	> 0.001	10	0.089	-	-	-	-	0.467	-0.428	-	-	-	0.084	-	-	-	-	-
332.573	27.200	> 0.001	10	-0.083	-	0.047	-	0.104	-	-	-0.052	-	-	-	-	-	-	-	-
332.636	27.263	> 0.001	17	0.233	0.055	0.005	-	-	-	-	-	-	-	0.083	-	-0.187	-	-	-
332.648	27.275	> 0.001	8	0.119	-	-	-	-	-	-	-0.039	-	-	0.168	-	-	-	-	-
332.686	27.313	> 0.001	12	0.190	-0.251	-	-	-	-	-	-0.509	-	-	0.031	-	-	-	-	-
332.754	27.381	> 0.001	10	0.073	-	-	-	-	0.452	-0.412	-0.043	-	-	-	-	-	-	-	-
332.817	27.444	> 0.001	12	0.247	-0.228	-	-	-	-	-	-0.266	-	-	0.180	-	-	-	-	-
332.895	27.522	> 0.001	9	0.104	-	-0.111	-	-	-	-	-0.667	-	-	0.102	-	-	-	-	0.754
332.952	27.580	> 0.001	11	0.179	-0.183	0.183	-	-	-	-	-	-	-	-	-	-	-	-	-
333.020	27.647	> 0.001	7	0.077	-	-	-	-	0.105	-	-	-	-	0.093	-	-	-	-	-
333.042	27.669	> 0.001	18	0.177	-0.024	-	-	-	-	-	-0.369	-	-	-	-	-0.115	-	-	-
333.064	27.691	> 0.001	7	0.079	-	-0.060	-	-	0.109	-	-	-	-	-	-	-	-	-	-
333.112	27.739	> 0.001	11	-0.099	-	0.066	-	0.123	0.246	-	-	-	-	-	-	-	-	-	-
333.112	27.739	> 0.001	7	0.076	-	-	-	-	-	-	-0.114	-	-	0.074	-	-	-	-	-

333.136	27.763	> 0.001	8	0.058	-	-	-	-	0.201	-	-0.005	-	-	-	-	-	-	-0.384	-
333.218	27.845	> 0.001	11	-0.052	-	0.102	-	0.051	-	-	-0.028	-	-	0.203	-	-	-	-	-
333.271	27.898	> 0.001	7	0.072	-	-0.022	-	-	-	-	-0.110	-	-	-	-	-	-	-	-
333.463	28.090	> 0.001	11	0.218	0.043	-	-	-	-0.366	-	-	-	-	0.115	-	-	-	-	-
333.500	28.127	> 0.001	7	0.093	-	-0.047	-	-	-	-	-	-	-	0.083	-	-	-	-	-
333.525	28.152	> 0.001	11	0.246	-0.191	-	-	-	-	-	-	-	-	0.232	-	-	-	-	-
333.702	28.329	> 0.001	11	-0.091	-	0.013	-	0.126	0.122	-	-0.070	-	-	-	-	-	-	-	-
333.724	28.351	> 0.001	11	0.218	-0.189	-	-	-	-	-	-	-	-	0.143	-	-	-	-	-
333.767	28.395	> 0.001	11	0.100	-	-	-	-	0.519	-0.452	-	-	-	0.149	-	-	-	-	-
333.942	28.569	> 0.001	11	0.209	-0.211	0.032	-	-	-	-	-	-	-	0.161	-	-	-	-	-
333.958	28.586	> 0.001	11	0.205	0.037	0.017	-	-	-0.387	-	-	-	-	0.063	-	-	-	-	-
334.012	28.639	> 0.001	18	0.213	0.017	0.068	-	-	-	-	-	-	-	-	-	-0.136	-	-	-
334.050	28.677	> 0.001	11	0.194	0.029	0.034	-	-	-0.343	-	-	-	-	-	-	-	-	-	-
334.079	28.706	> 0.001	9	0.112	-	-	-	-	0.100	-	-0.060	-	-	0.173	-	-	-	-	-
334.190	28.817	> 0.001	11	0.111	-	-0.071	-	-	0.493	-0.440	-	-	-	0.089	-	-	-	-	-
334.199	28.826	> 0.001	8	0.143	-	-	-	-	-	-	-	-	-	0.317	-	-	-	-	-
334.226	28.853	> 0.001	12	0.171	-	-	-	-	0.501	-0.461	-	-	-	0.376	-	-	-	-	-
334.269	28.896	> 0.001	8	0.071	-	-	-	-	0.121	-	-0.135	-	-	0.086	-	-	-	-	-
334.370	28.997	> 0.001	12	-0.022	-	0.145	-	0.026	-	-	-	-	-	0.367	-	-	-	-	-
334.395	29.022	> 0.001	8	0.039	-	-	-	-	-	-	-0.425	-	-	-	-	-	-	-	-
334.403	29.030	> 0.001	12	0.180	0.149	0.206	-	-	-0.511	-	-	-	-	-	-	-	-	-	-
334.411	29.038	> 0.001	11	0.089	-	-0.051	-	-	0.513	-0.416	-	-	-	-	-	-	-	-	-
334.448	29.075	> 0.001	8	0.068	-	-0.035	-	-	0.120	-	-0.126	-	-	-	-	-	-	-	-
334.464	29.091	> 0.001	11	0.093	-	-0.065	-	-	0.478	-0.426	-0.018	-	-	-	-	-	-	-	-
334.505	29.133	> 0.001	11	0.088	-	-	-	-	0.462	-0.422	-0.040	-	-	0.082	-	-	-	-	-
334.509	29.136	> 0.001	9	0.132	-	-0.045	-	-	-	-	-0.020	-	-	0.173	-	-	-	-	-
334.560	29.187	> 0.001	11	0.074	-	-	-	-	0.457	-0.396	0.002	-	-	-	-	-	-	-0.152	-
334.680	29.307	> 0.001	8	0.071	-	-0.018	-	-	0.202	-	-	-	-	-	-	-	-	-	-
334.693	29.320	> 0.001	12	0.190	-0.175	0.184	-	-	-	-	-	-	-	0.084	-	-	-	-	-
334.737	29.364	> 0.001	8	0.093	-	-0.062	-	-	0.116	-	-	-	-	0.096	-	-	-	-	-
334.772	29.400	> 0.001	8	0.083	-	-	-	-	0.125	-	-	-	-	0.130	-	-	-	-	-
334.809	29.436	> 0.001	8	0.104	-	-0.015	-	-	-	-	-	-	-	0.221	-	-	-	-	-
334.905	29.532	> 0.001	12	-0.102	-	0.088	-	0.050	-	-	-0.447	-	-	-	-	-	-	-	-
334.905	29.533	> 0.001	9	0.069	-	-	-	-	0.205	-	-0.004	-	-	0.080	-	-	-	-0.379	-
334.970	29.597	> 0.001	9	0.071	-	-0.049	-	-	0.210	-	0.016	-	-	-	-	-	-	-0.396	-
335.071	29.698	> 0.001	8	0.083	-	-0.024	-	-	-	-	-0.105	-	-	0.075	-	-	-	-	-
335.091	29.718	> 0.001	10	0.069	-	-0.103	-	-	-	-	-0.759	-	-	-	-	-	-	-	0.719
335.224	29.852	> 0.001	12	0.247	0.052	-	-	-	-0.374	-	-	-	-	0.229	-	-	-	-	-
335.262	29.889	> 0.001	10	0.069	-	-	-	-	-	-	-0.389	-	-	0.158	-	-	-	-	-
335.441	30.068	> 0.001	12	0.220	0.047	-	-	-	-0.364	-	-	-	-	0.137	-	-	-	-	-
335.471	30.098	> 0.001	10	0.027	-	-	-	-	0.197	-	-0.381	-	-	-	-	-	-	-0.541	-
335.481	30.108	> 0.001	12	0.242	-0.199	0.026	-	-	-	-	-	-	-	0.239	-	-	-	-	-
335.531	30.158	> 0.001	9	0.141	-	-	-	-	0.107	-	-	-	-	0.330	-	-	-	-	-
335.721	30.348	> 0.001	12	0.217	-0.191	0.006	-	-	-	-	-	-	-	0.142	-	-	-	-	-
335.725	30.352	> 0.001	9	0.134	-	-	-	-	-	-	-0.107	-	-	0.305	-	-	-	-	-
335.827	30.454	> 0.001	10	0.139	-	-	-	-	-	-	-0.048	-	-	0.312	-	-	-	-	-
335.879	30.506	> 0.001	9	0.033	-	-	-	-	0.097	-	-0.403	-	-	-	-	-	-	-	-
335.921	30.548	> 0.001	12	0.024	-	-	-	-	0.419	-0.374	-0.331	-	-	-	-	-	-	-	-
335.938	30.565	> 0.001	9	0.077	-	-	-	-	0.145	-	-0.143	-	-	0.128	-	-	-	-	-
336.076	30.703	> 0.001	9	0.061	-	0.006	-	-	0.212	-	-0.125	-	-	-	-	-	-	-	-

336.164	30.792	> 0.001	9	0.103	-	-0.033	-	-	0.107	-	-	-	-	0.221	-	-	-	-	-
336.169	30.796	> 0.001	9	0.081	-	-0.038	-	-	0.126	-	-0.122	-	-	0.089	-	-	-	-	-
336.171	30.799	> 0.001	9	0.147	-	-0.020	-	-	-	-	-	-	-	0.311	-	-	-	-	-
336.179	30.806	> 0.001	9	0.094	-	0.016	-	-	-	-	-0.129	-	-	0.232	-	-	-	-	-
336.241	30.868	> 0.001	9	0.052	-	-	-	-	-	-	-0.410	-	-	0.067	-	-	-	-	-
336.335	30.962	> 0.001	9	0.048	-	-0.030	-	-	-	-	-0.418	-	-	-	-	-	-	-	-
336.410	31.037	> 0.001	9	0.084	-	-0.023	-	-	0.201	-	-	-	-	0.088	-	-	-	-	-
336.434	31.061	> 0.001	9	0.101	-	-0.069	-	-	0.139	-	-	-	-	0.137	-	-	-	-	-
336.878	31.505	> 0.001	10	0.130	-	-	-	-	0.121	-	-0.127	-	-	0.317	-	-	-	-	-
337.013	31.640	> 0.001	13	0.251	-0.191	-	-	-	-	-	-	-	-	0.251	-	-	-	-	-
337.403	32.031	> 0.001	10	0.140	-	-	-	-	0.121	-	-	-	-	0.350	-	-	-	-	-
337.430	32.058	> 0.001	10	0.149	-	-0.039	-	-	0.114	-	-	-	-	0.317	-	-	-	-	-
337.682	32.309	> 0.001	10	0.047	-	-	-	-	0.102	-	-0.385	-	-	0.076	-	-	-	-	-
337.721	32.348	> 0.001	10	0.132	-	0.008	-	-	-	-	-0.110	-	-	0.307	-	-	-	-	-
337.780	32.407	> 0.001	10	0.044	-	-0.038	-	-	0.102	-	-0.393	-	-	-	-	-	-	-	-
338.170	32.798	> 0.001	10	0.062	-	-0.032	-	-	-	-	-0.401	-	-	0.069	-	-	-	-	-
339.118	33.745	> 0.001	11	0.105	-	-	-	-	-	-	-0.363	-	-	0.276	-	-	-	-	-
339.924	34.552	> 0.001	8	-	0.294	-	-	-	-0.628	-	-0.137	-	-	-	-	-	-	-0.667	-
340.658	35.285	> 0.001	8	-	-0.108	-	-	-	-	-	-0.112	-0.427	-	-	-	-	-	-	-
340.666	35.293	> 0.001	6	-	-0.212	-	-	-	-	-	-0.372	-	-	-	-	-	-	-	-
341.764	36.391	> 0.001	7	-	0.232	-	-	-	-0.683	-	-0.381	-	-	-	-	-	-	-	-
341.779	36.406	> 0.001	9	-	0.299	0.050	-	-	-0.668	-	-0.167	-	-	-	-	-	-	-0.659	-
341.799	36.426	> 0.001	9	-	0.293	-	-	-	-0.616	-	-0.134	-	-	0.061	-	-	-	-0.662	-
341.884	36.511	> 0.001	9	-	0.296	-	-	-	-0.625	-	-0.125	-0.415	-	-	-	-	-	-	-
342.432	37.059	> 0.001	7	-	-0.202	-	-	-	-	-	-0.366	-	-	0.084	-	-	-	-	-
342.447	37.074	> 0.001	9	-	-0.098	-	-	-	-	-	-0.102	-0.469	-	0.081	-	-	-	-	-
342.537	37.164	> 0.001	7	-	-0.231	0.047	-	-	-	-	-0.397	-	-	-	-	-	-	-	-
342.616	37.243	> 0.001	9	-	-0.121	0.027	-	-	-	-	-0.130	-0.407	-	-	-	-	-	-	-
343.039	37.666	> 0.001	9	-	-0.228	0.133	-	-	-	-	-0.414	-	-	0.362	-	-	-	-	-
343.511	38.138	> 0.001	8	-	0.239	0.066	-	-	-0.735	-	-0.415	-	-	-	-	-	-	-	-
343.570	38.197	> 0.001	8	-	0.231	-	-	-	-0.668	-	-0.374	-	-	0.076	-	-	-	-	-
343.664	38.291	> 0.001	5	-	-0.171	-	-	-	-	-	-	-	-	-	-	-	-	-	-
343.681	38.308	> 0.001	10	-	-0.093	-	-	-	-	-	-0.049	-0.207	-	0.119	-	-	-	-	-
343.711	38.338	> 0.001	10	-	0.295	-	-	-	-0.610	-	-0.115	-0.452	-	0.073	-	-	-	-	-
343.763	38.390	> 0.001	10	-	-0.100	-0.017	-	-	-	-	-0.426	-0.267	-	-	-	-	-	-	0.380
343.764	38.391	> 0.001	10	-	0.300	0.046	-	-	-0.664	-	-0.157	-0.380	-	-	-	-	-	-	-
343.847	38.474	> 0.001	8	-	-0.206	0.001	-	-	-	-	-0.628	-	-	-	-	-	-	-	0.322
344.105	38.733	> 0.001	9	-	-0.203	0.310	-	-	-	-	-0.500	-	-	-	-	-	-	-	-
344.275	38.902	> 0.001	8	-	-0.199	-	-	-	-	-	-0.342	-	-	0.108	-	-	-	-	-
344.323	38.950	> 0.001	8	-	-0.220	0.043	-	-	-	-	-0.388	-	-	0.080	-	-	-	-	-
344.333	38.960	> 0.001	10	-	0.425	0.377	-	-	-0.974	-	-0.541	-	-	-	-	-	-	-	-
344.417	39.044	> 0.001	5	-	-	-	-	-	0.306	-	0.022	-	-	-	-	-	-	-0.672	-
344.424	39.051	> 0.001	10	-	-0.108	0.020	-	-	-	-	-0.116	-0.452	-	0.078	-	-	-	-	-
344.928	39.555	> 0.001	9	-	0.237	0.023	-	-	-0.697	-	-0.627	-	-	-	-	-	-	-	0.296
344.996	39.623	> 0.001	6	-	0.217	-	-	-	-0.597	-	-	-	-	-	-	-	-	-	-
345.104	39.732	> 0.001	2	-	-	-	-	-	-	-	-	-	-	-	-	-	-	-	-
345.223	39.850	> 0.001	6	-	-0.159	-	-	-	-	-	-	-	-	0.116	-	-	-	-	-
345.345	39.972	> 0.001	9	-	0.238	0.063	-	-	-0.719	-	-0.408	-	-	0.071	-	-	-	-	-
345.347	39.974	> 0.001	9	-	0.238	-	-	-	-0.664	-	-0.383	-	-	0.111	-	-	-	-	-
345.368	39.995	> 0.001	9	-	0.223	0.090	-	-	-0.640	-	-0.411	-	-	-	-	-	-	-	-

345.399	40.027	> 0.001	6	-	-0.149	-0.063	-	-	-	-	-	-	-	-	-	-	-	-	-
345.419	40.046	> 0.001	11	-	-0.127	0.164	-	-	-	-	-0.223	-0.384	-	-	-	-	-	-	-
345.427	40.054	> 0.001	9	-	0.230	-	-	-	-0.662	-	-0.352	-	-	0.099	-	-	-	-	-
345.431	40.058	> 0.001	7	-	-	-0.176	-	-	0.336	-	-0.362	-	-	-	-	-	-	-0.702	0.576
345.532	40.159	> 0.001	9	-	-0.191	-0.007	-	-	-	-	-0.635	-	-	0.098	-	-	-	-	0.347
345.647	40.274	> 0.001	11	-	-0.068	-	-	-	-	-	-0.092	-0.531	-	0.155	-	-	-	-	-
345.671	40.298	> 0.001	6	-	-	-0.108	-	-	0.324	-	0.069	-	-	-	-	-	-	-0.691	-
345.706	40.334	> 0.001	3	-	-	-	-	-	-	-	-0.192	-	-	-	-	-	-	-	-
345.766	40.393	> 0.001	10	-	-0.190	0.311	-	-	-	-	-0.494	-	-	0.101	-	-	-	-	-
345.834	40.461	> 0.001	10	-	-0.185	0.263	-	-	-	-	-0.640	-	-	-	-	-	-	-	0.211
345.906	40.533	> 0.001	6	-	-	-	-	-	0.311	-	0.025	-	-	0.125	-	-	-	-0.661	-
346.175	40.802	> 0.001	5	-	-	-0.020	-	-	-	-	-	-	-	0.438	-	-	-	-	-
346.195	40.822	> 0.001	9	-	-0.195	-	-	-	-	-	-0.376	-	-	0.110	-	-	-	-	-
346.197	40.824	> 0.001	9	-	-0.215	0.037	-	-	-	-	-0.364	-	-	0.103	-	-	-	-	-
346.208	40.835	> 0.001	3	-	-	-	-	-	0.132	-	-	-	-	-	-	-	-	-	-
346.283	40.911	> 0.001	7	-	-	-	-	-	0.314	-	0.124	-	-	0.200	-	-	-	-0.753	-
346.298	40.925	> 0.001	3	-	-	-0.108	-	-	-	-	-	-	-	-	-	-	-	-	-
346.369	40.996	> 0.001	8	-	-0.138	0.009	-	-	-	-	-	-	-	0.374	-	-	-	-	-
346.389	41.016	> 0.001	4	-	-	-	-	-	0.161	-	-0.221	-	-	-	-	-	-	-	-
346.505	41.133	> 0.001	3	-	-	-	-	-	-	-	-	-	-	0.138	-	-	-	-	-
346.598	41.225	> 0.001	7	-	0.216	-	-	-	-0.577	-	-	-	-	0.110	-	-	-	-	-
346.816	41.443	> 0.001	7	-	0.212	-0.053	-	-	-0.561	-	-	-	-	-	-	-	-	-	-
346.878	41.505	> 0.001	6	-	-	0.028	-	-	-	-	-0.192	-	-	0.444	-	-	-	-	-
346.934	41.561	> 0.001	7	-	-	-0.036	-	-	-	-	-0.597	-	-	0.458	-	-	-	-	0.540
346.942	41.569	> 0.001	7	-	-0.136	-0.065	-	-	-	-	-	-	-	0.118	-	-	-	-	-
347.071	41.698	> 0.001	4	-	-	-0.130	-	-	0.156	-	-	-	-	-	-	-	-	-	-
347.132	41.759	> 0.001	6	-	-	-0.044	-	-	0.143	-	-	-	-	0.438	-	-	-	-	-
347.140	41.767	> 0.001	7	-	-	-0.109	-	-	0.330	-	0.072	-	-	0.128	-	-	-	-0.680	-
347.218	41.845	> 0.001	4	-	-	-	-	-	-	-	-0.184	-	-	0.124	-	-	-	-	-
347.336	41.963	> 0.001	10	-	0.236	-	-	-	-0.667	-	-0.385	-	-	0.095	-	-	-	-	-
347.375	42.002	> 0.001	4	-	-	-0.072	-	-	-	-	-0.163	-	-	-	-	-	-	-	-
347.460	42.087	> 0.001	4	-	-	-	-	-	0.142	-	-	-	-	0.154	-	-	-	-	-
347.513	42.140	> 0.001	5	-	-	-0.134	-	-	-	-	-0.566	-	-	-	-	-	-	-	0.534
347.569	42.197	> 0.001	7	-	-	0.006	-	-	0.160	-	-0.211	-	-	0.445	-	-	-	-	-
347.617	42.244	> 0.001	7	-	-	-	-	-	0.341	-	0.025	-	-	0.166	-	-	-	-0.685	-
347.666	42.293	> 0.001	7	-	-	-0.103	-	-	0.334	-	0.067	-	-	-	-	-	-	-0.685	-
347.714	42.342	> 0.001	4	-	-	-0.107	-	-	-	-	-	-	-	0.136	-	-	-	-	-
347.750	42.378	> 0.001	9	-	0.230	0.021	-	-	-0.573	-	-	-	-	0.371	-	-	-	-	-
347.753	42.380	> 0.001	5	-	-	-	-	-	0.170	-	-0.214	-	-	0.142	-	-	-	-	-
347.828	42.455	> 0.001	6	-	-	-0.157	-	-	0.182	-	-0.607	-	-	-	-	-	-	-	0.557
347.865	42.492	> 0.001	5	-	-	-0.091	-	-	0.173	-	-0.186	-	-	-	-	-	-	-	-
347.989	42.616	> 0.001	10	-	-0.191	-	-	-	-	-	-0.351	-	-	0.137	-	-	-	-	-
348.098	42.725	> 0.001	10	-	-0.211	0.041	-	-	-	-	-0.397	-	-	0.109	-	-	-	-	-
348.159	42.786	> 0.001	7	-	-	0.016	-	-	-	-	-0.146	-	-	0.487	-	-	-	-	-
348.314	42.942	> 0.001	5	-	-	-0.130	-	-	0.166	-	-	-	-	0.154	-	-	-	-	-
348.375	43.002	> 0.001	5	-	-	-	-	-	-	-	-0.138	-	-	0.180	-	-	-	-	-
348.402	43.029	> 0.001	8	-	0.211	-0.055	-	-	-0.540	-	-	-	-	0.113	-	-	-	-	-
348.547	43.175	> 0.001	8	-	0.191	-0.018	-	-	-0.432	-	-	-	-	-	-	-	-	-	-
348.552	43.179	> 0.001	8	-	0.219	-	-	-	-0.574	-	-	-	-	0.127	-	-	-	-	-
348.653	43.280	> 0.001	7	-	-	0.007	-	-	0.250	-	-	-	-	0.439	-	-	-	-	-

348.724	43.351	> 0.001	5	-	-	-0.089	-	-	0.248	-	-	-	-	-	-	-	-	-	-
348.823	43.450	> 0.001	8	-	-0.121	0.048	-	-	-	-	-	-	-	-	-	-	-	-	-
348.842	43.469	> 0.001	6	-	-	-0.138	-	-	-	-	-0.576	-	-	0.145	-	-	-	-	0.560
348.880	43.507	> 0.001	5	-	-	-0.073	-	-	-	-	-0.155	-	-	0.125	-	-	-	-	-
348.945	43.572	> 0.001	7	-	-	-0.162	-	-	0.193	-	-0.621	-	-	0.166	-	-	-	-	0.587
349.000	43.627	> 0.001	7	-	-	-0.047	-	-	0.159	-	-	-	-	0.470	-	-	-	-	-
349.088	43.715	> 0.001	6	-	-	-	-	-	0.159	-	-0.171	-	-	0.190	-	-	-	-	-
349.110	43.738	> 0.001	8	-	-0.145	-	-	-	-	-	-	-	-	0.170	-	-	-	-	-
349.207	43.834	> 0.001	6	-	-	-0.093	-	-	0.182	-	-0.179	-	-	0.144	-	-	-	-	-
349.227	43.854	> 0.001	7	-	-	-0.108	-	-	0.304	-	-0.635	-	-	-	-	-	-	-	0.595
349.439	44.066	> 0.001	5	-	-	-	-	-	0.149	-	-	-	-	0.166	-	-	-	-	-
349.482	44.109	> 0.001	10	-	-0.103	0.151	-	-	-	-	-	-	-	0.396	-	-	-	-	-
349.524	44.151	> 0.001	6	-	-	-0.051	-	-	0.265	-	-0.186	-	-	-	-	-	-	-	-
349.682	44.309	> 0.001	6	-	-	-	-	-	0.182	-	-0.217	-	-	0.162	-	-	-	-	-
349.789	44.416	> 0.001	10	-	-0.149	0.011	-	-	-	-	-	-	-	0.360	-	-	-	-	-
349.937	44.564	> 0.001	6	-	-	-0.083	-	-	-	-	-0.103	-	-	0.185	-	-	-	-	-
350.038	44.665	> 0.001	6	-	-	-0.093	-	-	0.249	-	-	-	-	0.147	-	-	-	-	-
350.052	44.679	> 0.001	9	-	0.302	0.079	-	-	-0.650	-	-	-	-	-	-	-	-	-	-
350.176	44.803	> 0.001	9	-	0.191	-0.023	-	-	-0.422	-	-	-	-	0.107	-	-	-	-	-
350.256	44.883	> 0.001	6	-	-	-0.133	-	-	0.177	-	-	-	-	0.173	-	-	-	-	-
350.302	44.929	> 0.001	9	-	-0.106	0.054	-	-	-	-	-	-	-	0.127	-	-	-	-	-
350.335	44.962	> 0.001	7	-	-	-0.139	-	-	-	-	-0.495	-	-	0.187	-	-	-	-	0.505
350.340	44.967	> 0.001	9	-	0.214	-0.057	-	-	-0.535	-	-	-	-	0.132	-	-	-	-	-
350.445	45.073	> 0.001	7	-	-	-0.101	-	-	0.172	-	-0.130	-	-	0.196	-	-	-	-	-
350.504	45.131	> 0.001	9	-	0.223	-	-	-	-0.569	-	-	-	-	0.159	-	-	-	-	-
350.839	45.466	> 0.001	9	-	-0.124	-0.065	-	-	-	-	-	-	-	0.165	-	-	-	-	-
350.931	45.558	> 0.001	7	-	-	-0.057	-	-	0.265	-	-0.178	-	-	0.137	-	-	-	-	-
350.958	45.585	> 0.001	7	-	-	-	-	-	0.175	-	-0.174	-	-	0.221	-	-	-	-	-
351.107	45.734	> 0.001	7	-	-	-0.096	-	-	0.197	-	-0.182	-	-	0.168	-	-	-	-	-
351.572	46.199	> 0.001	10	-	0.305	0.084	-	-	-0.632	-	-	-	-	0.122	-	-	-	-	-
351.943	46.570	> 0.001	7	-	-	-0.094	-	-	0.269	-	-	-	-	0.171	-	-	-	-	-
352.313	46.940	> 0.001	10	-	0.217	-0.055	-	-	-0.532	-	-	-	-	0.155	-	-	-	-	-
354.166	48.793	> 0.001	11	-	-0.091	0.056	-	-	-	-	-	-	-	0.174	-	-	-	-	-

Resp. Age*Don. Age	Don. Sex*Resp. Rep.	Fam.*Resp. Age	Loc.*Resp. Age	Resp. Sex*Don. Sex	Loc.*Don. Age	Fam.*Don. Age	Don. Sex*Don. Age	Fam.*Resp. Rep.
-	-	-	-	-	-	-	-	-
-	-	-	-	-	-	-	-	-
-	-	-	-	-	-	-	-	-
-	-	-	-	-	-	-	-	-
-	-	-	-	-	-	-	-	-
-	-	-	-	-	-	-	-	-
-	-	-	-	-	-	-	-	-
-	-	-	-	-	-	-	-	-
-	-	-	-	-	-	-	-	-
-	-	-	-	-	-	-	-	-
-	-	-	-	-	-	-	-	-
-	-	-	-	-	-	-	-	-
-	-	-	-	-	-	-	-	-
-	-	-	-	-	-	-	-	-
-	-	-	-	-	-	-	-	-
-	-	-	-	-	-	-	-	-
-	-	-	-	-	-	-	-	-
-	-	-	-	-	-	-	-	-
-	-	-	-	-	-	-	-	-
-	-	-	-	-	-	-	-	-
-	-	-	-	-	-	-	-	-
-	-	-	-	-	-	-	-	-
-	-	-	-	-	-	-	-	-
-	-	-	-	-	-	-	-	-
-	-	-	-	-	-	-	-	-
-	-	-	-	-	-	-	-	-
-	-	-	-	-	-	-	-	-
-	-	-	-	-	-	-	-	-
-	-	-	-	-	-	-	-	-
-	-	-	-	-	-	-	-	-
-	-	-	-	-	-	-	-	-
-	-	-	-	-	-	-	-	-
-	-	-	-	-	-	-	-	-
-	-	-	-	-	-	-	-	-
-	-	-	-	-	-	-	-	-
-	-	-	-	-	-	-	-	-
-	-	-	-	-	-	-	-	-
-	-	-	-	-	-	-	-	-
-	-	-	-	-	-	-	-	-
-	-	-	-	-	-	-	-	-
-	-	-	-	-	-	-	-	-
-	-	-	-	-	-	-	-	-
-	-	-	-	-	-	-	-	-
-0.931	-	-	-	-	-	-	-	-
-	-	-	-	-	-	-	-	-
-	-0.198	-	-	-	-	-	-	-

-	-	-	-	-	-	-	-	-
-	-	-	-	-	-	-	-	-
-	-	-	-	-	-	-	-	-
-	-	-	-	-	-	-	-	-
-	-	-0.049	-	-	-	-	-	-
-	-	-	-	-	-	-	-	-
-	-	-	-	-	-	-	-	-
-	-	-	0.059	-	-	-	-	-
-	-	-	-	-	-	-	-	-
-	-	-	-	-	-	-	-	-
-	-	-	-	-	-	-	-	-
-	-	-	-	-	-	-	-	-
-	-	-	-	-	-	-	-	-
-	-	-	-	-	-	-	-	-
-	-	-	-	-	-	-	-	-
-	-	-	-	-	-	-	-	-
-	-	-	-	-	-	-	-	-
-	-	-	-	-	-	-	-	-
-	-	-	-	-	-	-	-	-
-	-	-	-	-	-	-	-	-
-	-	-	-	-	-	-	-	-
-	-	-	-	-	-	-	-	-
-	-	-	-	-	-	-	-	-
-	-	-	-	-	-	-	-	-
-	-	-	-	-	-0.290	-	-	-
-	-	-	-	-	-	-	-	-
-	-	-	-	-	-	-	-	-
-	-	-	-	-	-	-	-	-
-	-	-	-	-	-	-	-	-
-	-	-	-	-0.082	-	-	-	-
-	-	-	-	-	-	0.199	-	-
-	-	-	-	-	-	-	-	-
-	-	-	-	-	-	-	-0.360	-
-	-	-	-	-	-0.359	-	-	-
-	-	-	-	-	-	-	-	0.054
-	-	-	-	-	-	-	-	-
-	-	-	-	-	-0.374	-	-	-
-	-	-	-	-	-	-	-	-
-	-	-	-	-	-	-	-	-
-	-	-	-	-	-	-	-	-
-	-	-	-	-	-	0.208	-	-
-	-	-	-	-	-	-	-	0.058
-	-	-	0.058	-	-	-	-	-
-	-	-	-	-	-	-	-	-
-	-	-	-	-	-	-	-	-
-	-	-	-	-	-	0.248	-	-
-	-	0.070	-	-	-	-	-	-
-	-	-	-	-	-0.382	-	-	-
-	-	-	-	-	-	-	-	-
-	-	-	-	-	-	-	-	-

-	-	-	-	-	-	-	-	0.060
-	-	0.013	-	-	-	-	-	-
-	-	-	0.024	-	-	-	-	-
-	-	-	-	-	-0.383	-	-	-
-	-	-	-	-	-	-	-	0.069
-	-	-	-	-	-	-	-	-
-	-	-	-	-	-0.354	-	-	-
-	-	-	-	-	-	-	-	0.051
-	-	-	-	-	-	-	-	0.056
-	-	-	-	-	-	0.211	-	-
-	-	-	0.122	-	-	-	-	-
-	-	-	-	-	-	-	-	-
-	-	0.052	-	-	-	-	-	-
-	-	-	-	-	-	0.220	-	-
-	-	-	-	-	-	-	-	-
-	-	-	-	-	-	0.202	-	-
-	-	-	0.058	-	-	-	-	-
-	-	-	-	-	-	-	-	-
-	-	0.060	-	-	-	-	-	-
-	-	-	0.053	-	-	-	-	-
-	-	0.076	-	-	-	-	-	-
-	-	-	-	-	-	-	-	-
-	-	-	-	-	-	-	-	-
-	-	-	-	-	-0.447	-0.046	-	-
-	-	-	-	-	-	-	-	-
-	-	-	-	-	-	-	-	-
-	-	-	-	-	-	-	-	-
-	-	-	-	-	-	-	-	-
-	-	-	-	-	-	-	-	-
-	-	-	-	-	-	-	-	-
-	-	-	-	-	-	-	-	-
-	-	-	-	-	-	-	-	-
-	-	-	-	-	-	-	-	-
-	-	-	-	-	-	-	-	-
-	-	-	-	-	-	-	-	-
-	-	-	-	-	-	-	-	-
-	-	0.054	0.084	-	-	-	-	-
-	-	-	-	-	-	-	-	-
-	-	-	-	-	-	-	-	-
-	-	-	-	-	-	-	-0.567	-
-	-0.306	-	-	-	-	-	-	-
-	-	-	-	-	-	-	-	-
-	-	-	-	-	-	-	-	-
-	-	-	-	-	-	-	-	-
-	-	-	-	-	-	-	-	-
-	-	-	-	-	-	-	-	-
-	-	-	-	-	-	-	-	-
-0.535	-	-	-	-	-	-	-	-
-	-	-	-	-	-	-	-	-
-	-	-0.098	-	-	-	-	-	-

-	-	-	-	-	-	-	-	-
-	-	-	-	-	-	-	-	-
-	-	-	-	-	-	-	-	-
-	-	-	-	-	-	-	-	-
-	-	-	-	-	-	-	-	-
-	-	-	-	-	-	-	-	-
-	-	-	-	-	-	-	-	-
-	-	-	-	-	-	-	-	-
-	-	-	-	-	-	-	-	-
-	-	-	-	-	-	-	-	-
-	-	-	-	-	-	-	-	-
-	-	-	-	-	-	-	-	-
-	-	-	-	-	-	-	-0.645	-
-	-	-	-	-	-	-	-	-
-	-	-	-	-	-	-	-0.600	-
-	-	-0.086	-	-	-	-	-	-
-	-	-	-	-	-	-	-	-
-	-	-	-	-	-	-	-	-
-	-	-	-	-	-	-	-	-
-	-	-	-	-	-	-	-	-
-	-	-	-	-	-	-	-0.700	-
-	-0.185	-	-	-	-	-	-	-
-	-0.173	-	-	-	-	-	-	-
-	-	-0.125	-	-	-	-	-	-
-	-	-	-	-	-	-	-	-
-	-	-	-	-	-	-	-	-
-	-	-	-	-	-	-	-	-
-	-	-	-	-	-	-	-	-
-	-	-	-	-	-	-	-	-
-	-	-	-	-	-	-	-	-
-	-	0.035	-	-	-	-	-	-
-	-	-	-	-	-	-	-	-
-	-	-	-	-	-	-	-	-
-	-	-	-	-	-	-	-	-
-	-	-	-	-	-	-	-	-
-	-	-	-	-	-	0.259	-	-
-	-	-	-	-0.086	-	-	-	-
-	-	-	-	-	-	-	-	-
-	-	-	-	-	-	-	-	-
-0.705	-	-	-	-	-	-	-	-
-	-	-	-	-0.025	-	-	-	-
-	-	-	-	-	-	-	-	-
-	-	-	-	-	-	-	-	-
-	-	-	-	-	-	-	-0.663	-
-0.787	-	-	-	-	-	-	-	-
-	-	-	-	-	-	-	-	-
-	-	-	-	-	-	-	-	-
-	-	-	-	-	-	-	-	-
-	-	-0.088	-	-	-	-	-	-
-	-	-	-	-	-	0.173	-	-

-	-	-	-	-	-	-	-	0.011
-	-	-0.090	-	-	-	-	-	-
-	-	-	-	-	-	-	-	0.057
-	-	-	-	-	-	-	-	-
-	-	-	-	-	-	-	-	-
-	-	-	-	-0.089	-	-	-	-
-	-	-	-	-	-	-	-	-
-	-0.187	-	-	-	-	-	-	-
-	-	-	-	-	-	-	-	0.020
-	-	-	-	-	-	-	-	-
-	-	-	-	-	-	0.301	-	-
-	-	-	-	-	-	-	-	-
-	-	-	-	-	-	-	-	-
-	-0.175	-	-	-	-	-	-	-
-	-	-	-	-	-	-	-	-
-	-	-	-	-	-	-	-	-
-	-	-	-	-	-	-	-	-
-	-	-	-	-	-	0.276	-	-
-	-	-	-	-	-	-	-	-
-	-	-	-	-	-	-	-	-
-	-	-	-	-	-	-	-	-
-	-	-	-	-	-	-	-	-
-	-	-	-	-0.087	-	-	-	-
-	-	-	-	0.005	-	-	-	-
-	-	-	-	-	-	-	-	0.028
-	-	-	-	-	-	-	-	-
-	-	-0.067	-	-	-	-	-	-
-	-	-	-	-	-	-	-	-
-	-	-	-	-	-	0.248	-	-
-	-	-	-	-	-	0.256	-	-
-	-	-	-	-0.082	-	-	-	-
-	-	-	-	-	-	0.103	-0.693	-
-	-	-	-	-	-	-	-	-
-	-	-	-	-	-	-	-	-
-	-	-	-	-	-	-	-	0.148
-0.779	-	-	-	-	-	-	-	-
-	-	-	-	-	-	-	-	0.014
-	-	-	-	-	-	-	-	-
-	-	-	-	-	-	-	-	-
-	-	0.000	-	-	-	-	-	-
-	-	-	-	-	-	-	-	-
-	-	-	-	-	-	-	-	0.007
-	-	-	-	-	-	-	-	0.015
-	-	-	-	-	-	-	-	-
-	-	-	-	-	-	0.287	-	-
-	-	-	-	-	-	-	-	0.014
-	-	-	-	-	-	0.258	-	-
-	-	-	-	-	-	0.281	-	-
-	-	-	-	-	-	-	-	0.021
-	-	-	-	-	-	0.303	-	-

-1.102	-	-0.394	-	-	-	-	-	-
-	-	-0.082	-	-	-	-	-	-
-	-	-0.131	-	-	-	-	-	-
-	-	-	-	-	-	0.196	-	-
-	-	-0.052	-	-	-	-	-	-
-	-	-	-	-	-	-	-	-
-	-	-0.072	-	-	-	-	-	-
-	-	-	-	-	-	0.257	-	-
-	-	-	-	-	-	0.240	-	-
-	-	-	-	-	-	-	-	-
-	-	-	-	-	-	-	-	0.009
-	-	-	-	-	-	-	-	-
-0.788	-	-	-	-	-	0.002	-	-
-	-	-	-	-	-	-	-	0.016
-	-	-0.010	-	-	-	-	-	-
-	-	0.010	-	-	-	-	-	-
-	-	-	-	-	-	-	-	0.010
-	-	-	-	-	-	-	-	-
-	-	-	-	-	-	-	-	-
-	-	-	-	-	-	-	-	-
-	-0.251	-	-	-	-	-	-	-
-	-	-0.097	-	-	-	-	-	0.011
-	-0.123	-	-	-	-	-	-	0.010
-	-	-	-	-	-	0.314	-	-
-	-	-	-	-	-	-	-	-
-	-	-	-	-	-	-	-	0.006
-	-	-	-	-	-	0.245	-	-
-	-	0.042	-	-	-	-	-	-
-	-	-	-	-	-	-	-	-
-	-0.421	-	-	-	-	-	-	-
-	-	-	0.897	-	-	-	-	-
-	-	-	-	-	-	-	-	-
-	-	-0.001	-	-	-	-	-	-
-	-	-	-	-	-	-	-	-
-	-	-	-	-	-	0.195	-	0.015
-	-	-	-	-	-	-	-	-
-	-	-	-	-	-	-	-	-
-	-	-	-	-	-	-	-	-
-	-	-	-	-	-	-	-	-
-	-	-	-	-	-	-	-	-
-	-	-	-	-	-	-	-	-
-	-	-	-	-	-	-	-	-
-	-	-	-	-	-	-	-	-
-	-	-	-	-	-	-	-	-
-0.614	-	-	-	-	-	-	-	-
-	-	-	-	-	-	-	-	-
-	-	-	-	-	-	-	-	-
-	-	-	-	-	-	-	-	-
-	-	-	-	-	-	-	-	-
-	-	-	-	-	-	-	-	-

-	-	-0.030	-	-	-	0.246	-	-
-	-	-	-	-	-	-	-	-
-	-	-	-	-	-	-	-	-
-	-	-	-	-	-	-	-	-
-	-	-	-	-	-	-	-	-
-	-	-	0.347	-	-	-	-	-
-	-	-	-	-	-	-	-	-
-	-	-	-	-	-	-	-	-
-	-	-	-	-	-	-	-	-
-	-	-	-	-	-	-	-	-
-	-	-	-	-	-	-	-	-
-	-	-	-	-	-	-	-	-
-	-	-	-	-	-	-	-	-
-	-	-	-	-	-	-	-	-
-	-	-	-	-	-	-	-	-
-	-	-	-	-	-	-	-	-
-	-	-	-	-	-	-	-	-
-	-	-	-	-	-	-	-	-
-	-	-	-	-	-	-	-	-
-	-	-	-	-	-	-	-	-
-	-	-	-	-	-	-	-	-
-	-	-	-	-	-	-	-	-
-	-	-	0.354	-	-	-	-	-
-	-	-	-	-	-	-	-	-
-	-	-	-	-	-	-	-	-
-	-	-	-	-	-	-	-	-
-	-	-	0.347	-	-	-	-	-
-	-	-	-	-	-	-	-	-
-	-	-	-	-	-	-	-	-
-	-	-	-	-	-	-	-	-
-	-	-	-	-	-	-	-0.323	-
-	-	-	-	-	-	-	-	-
-	-	-	-	-	-	-	-	-
-	-	-	-	-	-	-	-	-
-	-	-	-	-	-	-	-	-
-	-	-	-	-	-0.548	-	-	-
-	-	-	-	-	-	-	-	-
-	-	-	-	-	-	-	-	-
-	-	-	-	-	-	-	-	-
-0.858	-	-	-	-	-	-	-	-
-	-	-	0.334	-	-	-	-	-
-	-	-	-	-	-	-	-	-
-	-	-	-	-	-0.226	-	-	-
-	-	-	-	-	-	-	-	-
-	-0.262	-	-	-	-	-	-	-
-	-	-	-	-	-	-	-	-
-	-	-	0.370	-	-	-	-	-
-	-	-	-	-	-	-	-	-
-	-	-	-	-0.231	-	-	-	-
-	-	-	-	-	-	-	-	-

-	-	-	-	-	-	-	-	-
-	-	-	-	-	-	-	-	-
-	-	-	-	-	-	-	-	-
-	-	-	-	-	-	-	-	-
-	-	-	-	-	-	-	-	-
-	-	-	-	-	-0.330	-	-	-
-	-	-	-	-	-	-	-	-
-	-	-	-	-	-	-	-	-
-	-	-	-	-	-	-	-	-
-	-	-	-	-	-	-	-0.201	-
-	-	-	-	-	-	-	-	-
-	-0.060	-	-	-	-	-	-	-
-	-	-	-	-0.063	-	-	-	-
-0.812	-	-	-	-	-	-	-	-
-	-	-	-	-	-	-	-	-
-	-	-	-	-	-0.429	-	-	-
-	-	-	-	-	-0.545	-	-	-
-	-	-	-	-	-	-	-	-
-	-	-	-	-	-0.181	-	-	-
-	-	-	0.334	-	-	-	-	-
-	-0.285	-	-	-	-	-	-	-
-	-	-	-	-0.067	-	-	-	-
-	-	-	-	-	-	-	-	-
-	-	-	-	-	-	-	-	-
-	-	-	-	-	-	-	-	-
-0.880	-	-	-	-	-	-	-	-
-	-	-	-	-	-	-	-	-
-	-	-	-	-0.165	-	-	-	-
-	-0.266	-	-	-	-	-	-	-
-	-	-	-	-	-	-	-	-
-	-	-	-	-	-	-	-	-
-	-	-	-	-	-	-	-0.294	-
-	-	-	0.411	-	-	-	-	-
-	-	-	-	-	-	-	-	-
-	-	-	-	-	-	-	-	-
-	-	-	-	-	-	-	-	-
-	-	-	0.189	-	-	-	-	-
-	-	-	-	-	-0.327	-	-	-
-1.031	-	-	0.554	-	-	-	-	-
-	-	-	-	-	-	-	-	-
-	-	-	0.440	-	-	-	-	-
-	-	-	-	-	-0.336	-	-	-
-	-	-	-	-	-0.440	-	-	-
-	-	-	-	-	-	-	-	-
-	-	-	-	-	-0.401	-	-	-
-0.832	-	-	-	-	-0.162	-	-	-
-	-	-	0.316	-	-	-	-	-
-	-	-	0.400	-	-	-	-	-
-	-	-	-	-	-	-	-	-
-	-	-	-	-0.162	-	-	-	-

-	-	-	-	-	-	-	-0.268	-
-	-	-	-	-	-	-	-	-
-	-	-	-	-	-0.423	-	-	-
-	-	-	-	-	-	-	-0.337	-
-	-	-	0.307	-	-	-	-	-
-	-	-	0.333	-	-	-	-	-
-	-	-	-	-0.153	-	-	-	-
-	-	-	-	-	-	-	-	-
-	-	-	-	-	-0.408	-	-	-
-	-	-	-	-	-0.376	-	-	-
-	-	-	-	-	-0.409	-	-	-
-	-	-	-	-	-0.429	-	-	-
-	-	-	0.284	-	-	-	-	-
-	-	-	-	-	-0.402	-	-	-
-	-	-	0.314	-	-	-	-	-
-	-	-	0.305	-	-	-	-	-
-	-	-	0.290	-	-0.369	-	-	-
-	-	-	-	-	-	-	-	-
-	-	-	-	-	-	-	-	-
-	-	-	-	-	-	-	-	-
-	-	-	-	-	-	-	-	-
-	-	-	-	-	-	-	-	-
-	-	-	-	-	-	-	-	-
-	-	-	-	-	-	-	-	-
-	-	-	-	-	-	-	-	-
-	-	-	-	-	-	-	-	-
-	-	-	-	-	-	-	-	-
-	-	-	-	-	-	-	-	-
-	-	-	-	-	-	-	-0.630	-
-	-	-	-	-	-	-	-	-
-	-	-	-	-	-	-	-	-
-	-	-	-	-	-	-	-	-
-0.658	-	-	-	-	-	-	-	-
-	-	-	-	-	-	-	-	-
-	-	-	-	-	-	-	-	-
-	-	-	-	-	-	-	-	-
-	-	-	-	-	-	-	-	-
-	-0.373	-	-	-	-	-	-	-
-0.223	-	-	-	-	-	-	-	-
-	-	-	-	-	-	-	-	-
-	-0.434	-	-	-	-	-	-	-
-	-	-	-	-	-	-	-	-
-	-	-	-	-	-	-	-	-
-	-	-	-	-	-	-	-	-
-	-	-	-	-	-	-	-	-
-	-	-	-	-	-	-	-	-
-	-	-	-	-	-	-	-	-
-	-	-	-	-	-	-	-	-
-	-	-	-	-	-	-	-	-
-	-	-	-	-0.110	-	-	-	-

-	-	-	-	-	-	-	-	-
-	-0.158	-	-	-	-	-	-	-
-0.213	-	-	-	-	-	-	-	-
-	-	-	-	-	-	-	-	-
-	-	-	-	-	-	-	-	-
-	-	-	-	-	-	-	-	-
-	-	-	-	-	-	-	-	-
-	-	-	-	-	-	-	-	-
-	-0.379	-	-	-	-	-	-	-
-	-0.356	-	-	-	-	-	-	-
-	-	-	-	-	-	-	-	-
-	-	-	-	-	-	-	-0.667	-
-	-	-	-	-	-	-	-	-
-0.202	-	-	-	-	-	-	-	-
-	-	-	-	-	-	-	-	-
-0.739	-	-	-	-	-	-	-	-
-	-	-	-	-	-	-	-	-
-	-	-	-	-	-	-	-0.565	-
-	-	-	-	-	-	-	-	-
-	-	-	-	-	-	-	-	-
-	-	-	-	-	-	-	-	-
-	-	-	-	-	-	-	-	-
-	-	-	-	-	-	-	-0.711	-
-	-	-	-	-	-	-	-0.698	-
-	-	-	-	-	-	-	-	-
-	-	-	-	-	-	-	-	-
-	-	-	-	-	-	-	-0.633	-
-	-	-	-	-	-	-	-	-
-	-	-	-	-	-	-	-	-
-	-	-	-	-	-	-	-	-
-	-	-	-	-	-	-	-	-
-	-	-	-	-	-	-	-	-
-	-	-	-	-	-	-	-	-
-	-	-	-	-	-	-	-0.677	-
-	-	-	-	-	-	-	-	-
-	-	-	-	-0.020	-	-	-	-
-	-	-	-	-	-	-	-	-
-	-	-	-	-	-	-	-0.572	-
-	-	-	-	-	-	-	-	-
-	-	-	-	-	-	-	-	-
-	-	-	-	-	-	-	-	-
-0.258	-	-	-	-	-	-	-	-
-	-	-	-	-	-	-	-	-
-0.484	-	-	-	-	-	-	-0.691	-
-	-	-	-	-	-	-	-	-
-0.528	-	-	-	-	-	-	-	-
-	-	-	-	-	-	-	-	-
-	-	-	-	-0.153	-	-	-	-
-	-	-	-	-	-	-	-	-
-	-	-	-	-0.194	-	-	-0.654	-

-	-	-	-	-0.166	-	-	-	-
-	-0.193	-	-	-	-	-	-	-
-	-	-	-	-	-	-	-	-
-	-	-	-	-	-	-	-	-
-	-	-	-	-	-	-	-	-
-	-	-	-	-	-	-	-0.641	-
-0.469	-	-	-	-	-	-	-	-
-	-	-	-	-	-	-	-	-
-	-	-	-	-	-	-	-	-
-	-	-	-	-0.218	-	-	-	-
-	-	-	-	-	-	-	-	-
-	-0.238	-	-	-	-	-	-0.592	-
-	-	-	-	-0.164	-	-	-	-
-	-	-	-	-	-	-	-	-
-	-	-	-	-	-	-	-0.694	-
-0.560	-	-	-	-	-	-	-	-
-	-	-	-	-0.148	-	-	-	-
-	-0.224	-	-	-	-	-	-	-
-	-	-	-	-0.140	-	-	-	-
-	-	-	-	-	-	-	-	-
-	-0.203	-	-	-	-	-	-	-
-0.416	-	-	-	-	-	-	-	-
-	-	-	-	-	-	-	-	-
-0.502	-	-	-	-	-	-	-	-
-	-	-	-	-	-	-	-	-
-	-	-	-	-	-	-	-	-
-	-	-	-	-0.148	-	-	-	-
-0.492	-	-	-	-	-	-	-	-
-	-	-	-	-	-	-	-	-
-	-0.232	-	-	-	-	-	-	-
-	-	-	-	-0.159	-	-	-	-
-	-	-	-	-	-	-	-	-
-	-0.209	-	-	-	-	-	-	-

Table S4:

Statistical summary of the models linking subcaudal over-marking responses to a global model that included the variables; ‘age’ of the donor and responder (levels: yearling, adult); ‘sex’ of the donor and the responder (levels: male, female); ‘reproductive status of the female’ donor and responder (levels: oestrous, non-oestrous); and ‘reproductive status of the male’ donor and responder (levels: descended, fully descended), as well as interaction terms, as factors in these models. Responder and trial ID were included as random effects in these models. This table is the basis of the model averaging, for which results are presented in Table 2b of the main text.

The support for each model, based on Akaike criterion, is presented in the first three columns. The fourth column presents the degrees of freedom associated with each model. Subsequent columns present coefficient estimates of the parameters included in each model.
