# Supplementary material for: Will Trespassers Be Prosecuted or Assessed According to Their Merits? A Consilient Interpretation of Territoriality in a Group-Living Carnivore, the European Badger (Meles meles)
Source: PLoS One. 2015 Jul 6;10(7):e0132432. doi: 10.1371/journal.pone.0132432 (PMC4493095; doi:10.1371/journal.pone.0132432)
Supplement: S5 Table — (DOCX) [file pone.0132432.s005.docx]

| Date | Group | Treatment | Location | ScID | ScSex | ScAge | ScRep | RespID | RespSex | RespAge | RespRep | LogSniff | nrSmark |
| --- | --- | --- | --- | --- | --- | --- | --- | --- | --- | --- | --- | --- | --- |
| 30/05/2012 | K | N | Shared | 31 | M | juvenile | fully descended | - | - | - | - | 0 | 0 |
| 30/05/2012 | K | N | Shared | 31 | M | juvenile | fully descended | - | - | - | - | - | 1 |
| 31/05/2012 | J | S | Border | 31 | M | juvenile | fully descended | 78 | M | - | - | 0.77815125 | 2 |
| 31/05/2012 | J | S | Border | 31 | M | juvenile | fully descended | 79 | M | - | - | - | 0 |
| 31/05/2012 | J | S | Border | 31 | M | juvenile | fully descended | 80 | M | - | - | - | 0 |
| 31/05/2012 | J | S | Border | 31 | M | juvenile | fully descended | 79 | M | - | - | 0.477121255 | 1 |
| 01/06/2012 | L | S | Sett | 48 | M | adult | descended | - | - | - | - | 0 | 0 |
| 01/06/2012 | J | N | Shared | 14 | M | adult | fully descended | - | - | - | - | 0.477121255 | 0 |
| 01/06/2012 | J | N | Shared | 14 | M | adult | fully descended | - | - | - | - | - | 2 |
| 01/06/2012 | J | N | Shared | 14 | M | adult | fully descended | 77 | M | - | - | 0 | 0 |
| 01/06/2012 | L | N | Unshared | 23 | M | adult | fully descended | 28 | M | adult | - | 0.954242509 | 1 |
| 01/06/2012 | K | S | Sett | 48 | M | adult | descended | 67 | F | adult | non-oestrous | 0 | 0 |
| 01/06/2012 | K | S | Sett | 48 | M | adult | descended | - | - | - | - | - | 1 |
| 01/06/2012 | K | S | Sett | 48 | M | adult | descended | - | - | - | - | 0 | 0 |
| 02/06/2012 | J | S | Border | 11 | M | adult | descended | - | - | - | - | 0.602059991 | 2 |
| 02/06/2012 | J | S | Border | 11 | M | adult | descended | 79 | M | - | - | 0.903089987 | 2 |
| 02/06/2012 | J | S | Border | 11 | M | adult | descended | 81 | M | - | - | 0.301029996 | 2 |
| 02/06/2012 | J | S | Border | 11 | M | adult | descended | 78 | M | - | - | 0.477121255 | 1 |
| 02/06/2012 | K | N | Unshared | 22 | M | adult | fully descended | - | - | - | - | - | 0 |
| 02/06/2012 | K | N | Unshared | 22 | M | adult | fully descended | - | - | - | - | 0.301029996 | 2 |
| 02/06/2012 | K | N | Unshared | 22 | M | adult | fully descended | - | - | - | - | 0 | 0 |
| 02/06/2012 | K | S | Border | 11 | M | adult | descended | - | - | - | - | 0.602059991 | 1 |
| 02/06/2012 | J | S | Sett | 9 | M | adult | fully descended | 78 | M | - | - | 0.698970004 | 0 |
| 02/06/2012 | J | S | Sett | 9 | M | adult | fully descended | 79 | M | - | - | 0.301029996 | 0 |
| 02/06/2012 | J | S | Sett | 9 | M | adult | fully descended | 77 | M | - | - | 0.301029996 | 0 |
| 02/06/2012 | J | S | Sett | 9 | M | adult | fully descended | 76 | F | - | - | 0.301029996 | 1 |
| 03/06/2012 | J | O | Sett | 8 | M | adult | fully descended | - | - | - | - | 0 | 0 |
| 03/06/2012 | K | N | Sett | 30 | M | adult | fully descended | - | - | - | - | 0.84509804 | 0 |
| 04/06/2012 | L | O | Border | 28 | M | adult | fully descended | 60 | F | adult | oestrous | 0.602059991 | 0 |
| 04/06/2012 | L | O | Border | 28 | M | adult | fully descended | - | - | - | - | 0 | 0 |
| 04/06/2012 | L | S | Border | 26 | M | adult | fully descended | - | - | - | - | 0.698970004 | 1 |
| 04/06/2012 | L | S | Border | 26 | M | adult | fully descended | - | - | - | - | 1.230448921 | 0 |
| 04/06/2012 | J | N | Sett | 49 | M | adult | descended | 79 | M | - | - | 0 | 0 |
| 04/06/2012 | J | N | Sett | 49 | M | adult | descended | 79 | M | - | - | 0.698970004 | 0 |
| 04/06/2012 | J | N | Sett | 49 | M | adult | descended | - | - | - | - | 0.903089987 | 1 |
| 04/06/2012 | J | N | Sett | 49 | M | adult | descended | - | - | - | - | 0 | 0 |
| 04/06/2012 | J | N | Sett | 49 | M | adult | descended | - | - | - | - | 0.84509804 | 0 |
| 04/06/2012 | J | N | Sett | 49 | M | adult | descended | 79 | M | - | - | 0.477121255 | 0 |
| 04/06/2012 | J | N | Sett | 49 | M | adult | descended | - | - | - | - | 0 | 0 |
| 04/06/2012 | J | N | Sett | 49 | M | adult | descended | 76 | F | - | - | 0.301029996 | 1 |
| 04/06/2012 | L | N | Shared | 16 | M | adult | fully descended | 60 | F | adult | oestrous | 0.77815125 | 1 |
| 04/06/2012 | L | N | Shared | 16 | M | adult | fully descended | - | - | - | - | 0 | 0 |
| 04/06/2012 | L | N | Shared | 16 | M | adult | fully descended | - | - | - | - | 0 | 0 |
| 04/06/2012 | L | N | Shared | 16 | M | adult | fully descended | - | - | - | - | 0 | 0 |
| 04/06/2012 | L | N | Shared | 16 | M | adult | fully descended | - | - | - | - | 0 | 0 |
| 04/06/2012 | L | N | Shared | 16 | M | adult | fully descended | - | - | - | - | 0 | 0 |
| 04/06/2012 | L | N | Shared | 16 | M | adult | fully descended | - | - | - | - | 0 | 0 |
| 04/06/2012 | L | N | Shared | 16 | M | adult | fully descended | - | - | - | - | 0 | 0 |
| 04/06/2012 | L | N | Shared | 16 | M | adult | fully descended | - | - | - | - | 0 | 0 |
| 04/06/2012 | L | N | Shared | 16 | M | adult | fully descended | - | - | - | - | 0 | 0 |
| 04/06/2012 | M | N | Sett | 49 | M | adult | descended | 16 | M | adult | fully descended | 0 | 0 |
| 05/06/2012 | M | S | Sett | 9 | M | adult | fully descended | 58 | F | adult | non-oestrous | 0.84509804 | 2 |
| 05/06/2012 | L | O | Border | 49 | M | adult | descended | - | - | - | - | 0 | 0 |
| 05/06/2012 | J | N | Unshared | 49 | M | adult | descended | - | - | - | - | 0 | 0 |
| 05/06/2012 | J | N | Unshared | 49 | M | adult | descended | - | - | - | - | 0 | 0 |
| 05/06/2012 | L | S | Sett | 11 | M | adult | descended | - | - | - | - | 0.698970004 | 1 |
| 05/06/2012 | L | O | Border | 28 | M | adult | fully descended | - | - | - | - | 0 | 0 |
| 06/06/2012 | M | O | Sett | 16 | M | adult | fully descended | 58 | F | adult | non-oestrous | 0 | 0 |
| 06/06/2012 | M | O | Sett | 16 | M | adult | fully descended | - | - | - | - | 0 | 0 |
| 06/06/2012 | L | S | Border | 9 | M | adult | fully descended | 75 | F | adult | oestrous | 0.77815125 | 0 |
| 06/06/2012 | L | S | Border | 9 | M | adult | fully descended | 68 | F | adult | non-oestrous | - | 0 |
| 06/06/2012 | L | S | Border | 9 | M | adult | fully descended | 75 | F | adult | oestrous | 0 | 0 |
| 06/06/2012 | L | S | Border | 9 | M | adult | fully descended | - | - | - | - | 0.301029996 | 2 |
| 06/06/2012 | L | S | Border | 9 | M | adult | fully descended | 68 | F | adult | non-oestrous | 0.602059991 | 1 |
| 06/06/2012 | L | S | Border | 9 | M | adult | fully descended | - | - | - | - | - | 0 |
| 06/06/2012 | L | O | Sett | 28 | M | adult | fully descended | 60 | F | adult | oestrous | 0 | 0 |
| 06/06/2012 | L | O | Sett | 28 | M | adult | fully descended | 68 | F | adult | non-oestrous | 0.84509804 | 0 |
| 06/06/2012 | L | O | Sett | 28 | M | adult | fully descended | 75 | F | adult | oestrous | 0 | 0 |
| 06/06/2012 | L | O | Sett | 28 | M | adult | fully descended | - | - | - | - | 0 | 0 |
| 06/06/2012 | L | O | Sett | 28 | M | adult | fully descended | 50 | F | adult | - | 0 | 0 |
| 06/06/2012 | L | O | Sett | 28 | M | adult | fully descended | - | - | - | - | 0 | 0 |
| 06/06/2012 | C | N | Sett | 6 | M | adult | fully descended | - | - | - | - | 0.477121255 | 0 |
| 06/06/2012 | C | N | Sett | 6 | M | adult | fully descended | 45 | F | adult | oestrous | 0 | 0 |
| 06/06/2012 | C | N | Sett | 6 | M | adult | fully descended | - | - | - | - | 0.77815125 | 0 |
| 06/06/2012 | C | N | Sett | 6 | M | adult | fully descended | - | - | - | - | 0.301029996 | 0 |
| 06/06/2012 | C | S | Border | 14 | M | adult | fully descended | - | - | - | - | 1.204119983 | 1 |
| 07/06/2012 | D | S | Border | 1 | M | adult | fully descended | - | - | - | - | 0.301029996 | 1 |
| 07/06/2012 | D | S | Border | 1 | M | adult | fully descended | - | - | - | - | 0.698970004 | 0 |
| 07/06/2012 | D | S | Border | 1 | M | adult | fully descended | - | - | - | - | 0.477121255 | 0 |
| 07/06/2012 | D | S | Border | 1 | M | adult | fully descended | 54 | F | adult | oestrous | 0.301029996 | 0 |
| 07/06/2012 | D | S | Border | 1 | M | adult | fully descended | - | - | - | - | 0 | 0 |
| 07/06/2012 | D | S | Border | 1 | M | adult | fully descended | - | - | - | - | 0.301029996 | 1 |
| 07/06/2012 | C | S | Border | 10 | F | adult | oestrous | 54 | F | adult | oestrous | 0.698970004 | 2 |
| 07/06/2012 | C | S | Sett | 49 | M | adult | descended | - | - | - | - | 1 | 0 |
| 07/06/2012 | C | S | Sett | 49 | M | adult | descended | - | - | - | - | 0.84509804 | 2 |
| 07/06/2012 | C | N | Unshared | 9 | M | adult | fully descended | 54 | F | adult | oestrous | 0.477121255 | 2 |
| 07/06/2012 | C | N | Unshared | 9 | M | adult | fully descended | 5 | M | adult | descended | 0.301029996 | 1 |
| 07/06/2012 | D | N | Sett | 5 | M | adult | fully descended | 63 | F | adult | non-oestrous | - | 0 |
| 08/06/2012 | D | N | Unshared | 5 | M | adult | fully descended | 54 | F | adult | oestrous | 0.301029996 | 1 |
| 08/06/2012 | D | N | Unshared | 5 | M | adult | fully descended | - | - | - | - | 0 | 0 |
| 08/06/2012 | D | N | Unshared | 5 | M | adult | fully descended | 63 | F | adult | non-oestrous | 0.602059991 | 3 |
| 08/06/2012 | C | N | Shared | 6 | M | adult | fully descended | 5 | M | adult | descended | 0 | 0 |
| 03/06/2013 | C | N | Sett | 47 | F | adult | non-oestrous | 45 | F | adult | oestrous | 0 | 0 |
| 03/06/2013 | C | N | Sett | 47 | F | adult | non-oestrous | - | - | - | - | 0 | 0 |
| 03/06/2013 | C | N | Sett | 47 | F | adult | non-oestrous | 45 | F | adult | oestrous | 0 | 0 |
| 04/06/2013 | C | N | Shared | 39 | F | juvenile | non-oestrous | - | - | - | - | 0.698970004 | 1 |
| 04/06/2013 | C | N | Shared | 39 | F | juvenile | non-oestrous | - | - | - | - | 0.84509804 | 1 |
| 04/06/2013 | C | N | Shared | 39 | F | juvenile | non-oestrous | - | - | - | - | 0.301029996 | 1 |
| 04/06/2013 | C | N | Shared | 39 | F | juvenile | non-oestrous | - | - | - | - | 0.77815125 | 1 |
| 05/06/2013 | C | S | Border | 25 | F | adult | oestrous | - | - | - | - | 0.602059991 | 1 |
| 05/06/2013 | C | S | Border | 25 | F | adult | oestrous | 62 | F | adult | non-oestrous | 0 | 0 |
| 05/06/2013 | C | S | Border | 25 | F | adult | oestrous | - | - | - | - | 0 | 0 |
| 05/06/2013 | C | S | Border | 25 | F | adult | oestrous | - | - | - | - | - | 1 |
| 05/06/2013 | C | S | Border | 25 | F | adult | oestrous | 62 | F | adult | non-oestrous | 0 | 0 |
| 05/06/2013 | C | S | Border | 25 | F | adult | oestrous | - | - | - | - | 0 | 0 |
| 05/06/2013 | C | S | Border | 25 | F | adult | oestrous | - | - | - | - | 0.301029996 | 1 |
| 05/06/2013 | C | S | Border | 25 | F | adult | oestrous | 29 | F | adult | non-oestrous | 0 | 0 |
| 05/06/2013 | C | N | Unshared | 4 | F | adult | non-oestrous | - | - | - | - | 0 | 0 |
| 05/06/2013 | C | N | Unshared | 4 | F | adult | non-oestrous | - | - | - | - | 0 | 0 |
| 05/06/2013 | C | N | Unshared | 4 | F | adult | non-oestrous | - | - | - | - | 0.301029996 | 1 |
| 05/06/2013 | C | N | Unshared | 4 | F | adult | non-oestrous | - | - | - | - | 0 | 0 |
| 05/06/2013 | G | O | Sett | 5 | M | adult | fully descended | 57 | F | adult | - | 0 | 0 |
| 05/06/2013 | G | O | Sett | 5 | M | adult | fully descended | - | - | - | - | 0 | 0 |
| 05/06/2013 | G | O | Sett | 5 | M | adult | fully descended | - | - | - | - | 0 | 0 |
| 05/06/2013 | G | O | Sett | 5 | M | adult | fully descended | 56 | F | adult | oestrous | 0 | 0 |
| 05/06/2013 | G | O | Sett | 5 | M | adult | fully descended | 56 | F | adult | oestrous | 0 | 0 |
| 05/06/2013 | G | O | Sett | 5 | M | adult | fully descended | 71 | M | juvenile | fully descended | 0 | 0 |
| 05/06/2013 | G | O | Sett | 5 | M | adult | fully descended | 73 | F | juvenile | non-oestrous | 0 | 0 |
| 05/06/2013 | G | O | Sett | 5 | M | adult | fully descended | 71 | M | juvenile | fully descended | 0 | 0 |
| 05/06/2013 | G | O | Sett | 5 | M | adult | fully descended | 56 | F | adult | oestrous | 0 | 0 |
| 05/06/2013 | G | O | Sett | 5 | M | adult | fully descended | 72 | M | juvenile | fully descended | 0 | 0 |
| 05/06/2013 | G | O | Sett | 5 | M | adult | fully descended | 56 | F | adult | oestrous | 0 | 0 |
| 05/06/2013 | G | O | Sett | 5 | M | adult | fully descended | 71 | M | juvenile | fully descended | 0 | 0 |
| 05/06/2013 | G | O | Sett | 5 | M | adult | fully descended | 71 | M | juvenile | fully descended | 0 | 0 |
| 05/06/2013 | G | O | Sett | 5 | M | adult | fully descended | 72 | M | juvenile | fully descended | 0 | 0 |
| 05/06/2013 | G | O | Sett | 5 | M | adult | fully descended | - | - | - | - | 0 | 0 |
| 05/06/2013 | G | O | Sett | 5 | M | adult | fully descended | 72 | M | juvenile | fully descended | 0 | 0 |
| 05/06/2013 | G | O | Sett | 5 | M | adult | fully descended | 57 | F | adult | - | 0 | 0 |
| 05/06/2013 | G | O | Sett | 5 | M | adult | fully descended | 73 | F | juvenile | non-oestrous | 0.301029996 | 1 |
| 05/06/2013 | I | N | Sett | 19 | F | adult | non-oestrous | 5 | M | adult | fully descended | 0 | 0 |
| 05/06/2013 | I | N | Sett | 19 | F | adult | non-oestrous | 5 | M | adult | fully descended | 0.77815125 | 0 |
| 05/06/2013 | I | N | Sett | 19 | F | adult | non-oestrous | 53 | M | adult | - | 0.698970004 | 0 |
| 05/06/2013 | I | N | Sett | 19 | F | adult | non-oestrous | 53 | M | adult | - | 0 | 0 |
| 05/06/2013 | I | N | Sett | 19 | F | adult | non-oestrous | - | - | - | - | 0.954242509 | 3 |
| 05/06/2013 | I | S | Border | 12 | M | adult | fully descended | - | - | - | - | 1 | 1 |
| 05/06/2013 | E | S | Sett | 41 | M | juvenile | fully descended | 74 | F | adult | - | 0.77815125 | 0 |
| 05/06/2013 | E | S | Sett | 41 | M | juvenile | fully descended | 19 | F | adult | oestrous | 0.602059991 | 1 |
| 05/06/2013 | E | S | Sett | 41 | M | juvenile | fully descended | - | - | - | - | 0 | 0 |
| 05/06/2013 | E | S | Sett | 41 | M | juvenile | fully descended | 47 | F | adult | non-oestrous | 0 | 0 |
| 06/06/2013 | C | S | Border | 24 | F | adult | oestrous | 62 | F | adult | non-oestrous | 0.301029996 | 0 |
| 06/06/2013 | C | S | Border | 24 | F | adult | oestrous | - | - | - | - | 0.698970004 | 0 |
| 06/06/2013 | C | S | Border | 24 | F | adult | oestrous | - | - | - | - | 0.698970004 | 1 |
| 06/06/2013 | C | O | Border | 45 | F | adult | oestrous | - | - | - | - | 0 | 0 |
| 06/06/2013 | C | O | Border | 45 | F | adult | oestrous | - | - | - | - | 0 | 0 |
| 06/06/2013 | C | O | Border | 45 | F | adult | oestrous | 62 | F | adult | non-oestrous | 0.477121255 | 0 |
| 06/06/2013 | C | O | Border | 45 | F | adult | oestrous | - | - | - | - | 0 | 0 |
| 06/06/2013 | G | S | Sett | 12 | M | adult | fully descended | 71 | M | juvenile | fully descended | 1.230448921 | 1 |
| 06/06/2013 | G | S | Sett | 12 | M | adult | fully descended | 56 | F | adult | oestrous | 0 | 0 |
| 06/06/2013 | G | S | Sett | 12 | M | adult | fully descended | - | - | - | - | 0 | 0 |
| 06/06/2013 | G | S | Sett | 12 | M | adult | fully descended | 57 | F | adult | - | 0 | 0 |
| 06/06/2013 | G | S | Sett | 12 | M | adult | fully descended | - | - | - | - | 1.176091259 | 1 |
| 06/06/2013 | G | S | Sett | 12 | M | adult | fully descended | - | - | - | - | 0.84509804 | 1 |
| 06/06/2013 | I | O | Sett | 39 | F | juvenile | non-oestrous | - | - | - | - | 0.477121255 | 0 |
| 06/06/2013 | I | O | Sett | 39 | F | juvenile | non-oestrous | - | - | - | - | 0.602059991 | 1 |
| 06/06/2013 | E | O | Sett | 37 | M | juvenile | fully descended | 19 | F | adult | oestrous | 0 | 0 |
| 06/06/2013 | E | O | Sett | 37 | M | juvenile | fully descended | - | - | - | - | 0.301029996 | 0 |
| 06/06/2013 | E | O | Sett | 37 | M | juvenile | fully descended | - | - | - | - | 0 | 0 |
| 06/06/2013 | E | O | Sett | 37 | M | juvenile | fully descended | - | - | - | - | 0 | 0 |
| 06/06/2013 | E | O | Sett | 37 | M | juvenile | fully descended | - | - | - | - | 0 | 0 |
| 06/06/2013 | E | O | Sett | 37 | M | juvenile | fully descended | 47 | F | adult | non-oestrous | 0 | 0 |
| 06/06/2013 | E | O | Sett | 37 | M | juvenile | fully descended | - | - | - | - | 0.84509804 | 0 |
| 06/06/2013 | E | O | Sett | 37 | M | juvenile | fully descended | 19 | F | adult | oestrous | 0 | 0 |
| 06/06/2013 | E | O | Sett | 37 | M | juvenile | fully descended | 74 | F | adult | - | 0 | 0 |
| 07/06/2013 | C | O | Border | 34 | F | juvenile | oestrous | - | - | - | - | 0.477121255 | 0 |
| 07/06/2013 | C | O | Border | 34 | F | juvenile | oestrous | - | - | - | - | 0.602059991 | 1 |
| 07/06/2013 | C | O | Border | 34 | F | juvenile | oestrous | - | - | - | - | 0.301029996 | 1 |
| 07/06/2013 | C | O | Border | 34 | F | juvenile | oestrous | 62 | F | adult | non-oestrous | 0.698970004 | 0 |
| 07/06/2013 | C | O | Border | 34 | F | juvenile | oestrous | - | - | - | - | 0 | 0 |
| 07/06/2013 | C | O | Border | 34 | F | juvenile | oestrous | 62 | F | adult | non-oestrous | 0.477121255 | 1 |
| 07/06/2013 | G | S | Sett | 40 | M | adult | fully descended | 74 | F | adult | - | 0.301029996 | 0 |
| 07/06/2013 | G | S | Sett | 40 | M | adult | fully descended | 74 | F | adult | - | 0.698970004 | 0 |
| 07/06/2013 | G | S | Sett | 40 | M | adult | fully descended | 73 | F | juvenile | non-oestrous | 0.602059991 | 1 |
| 07/06/2013 | G | S | Sett | 40 | M | adult | fully descended | 73 | F | juvenile | non-oestrous | 0 | 1 |
| 07/06/2013 | G | S | Sett | 40 | M | adult | fully descended | - | - | - | - | 0.698970004 | 0 |
| 07/06/2013 | G | S | Sett | 40 | M | adult | fully descended | - | - | - | - | 0 | 0 |
| 07/06/2013 | G | S | Sett | 40 | M | adult | fully descended | - | - | - | - | 0.602059991 | 0 |
| 07/06/2013 | G | S | Sett | 40 | M | adult | fully descended | 57 | F | adult | - | 1.176091259 | 0 |
| 07/06/2013 | G | S | Sett | 40 | M | adult | fully descended | 57 | F | adult | - | 0 | 0 |
| 07/06/2013 | I | S | Sett | 2 | F | adult | non-oestrous | 47 | F | adult | non-oestrous | 0.698970004 | 0 |
| 07/06/2013 | H | O | Border | 2 | F | adult | non-oestrous | - | - | - | - | 0 | 0 |
| 08/06/2013 | G | N | Sett | 38 | M | juvenile | fully descended | 74 | F | adult | - | 0.301029996 | 1 |
| 08/06/2013 | G | N | Sett | 38 | M | juvenile | fully descended | 73 | F | juvenile | non-oestrous | 0.698970004 | 0 |
| 08/06/2013 | G | N | Sett | 38 | M | juvenile | fully descended | 5 | M | adult | fully descended | 0.602059991 | 0 |
| 08/06/2013 | G | N | Sett | 38 | M | juvenile | fully descended | - | - | - | - | 0 | 0 |
| 08/06/2013 | G | N | Sett | 38 | M | juvenile | fully descended | 56 | F | adult | oestrous | 0 | 0 |
| 08/06/2013 | H | S | Border | 7 | F | adult | oestrous | - | - | - | - | 0 | 0 |
| 08/06/2013 | C | O | Sett | 21 | F | adult | non-oestrous | 62 | F | adult | non-oestrous | 0 | 0 |
| 08/06/2013 | C | O | Sett | 21 | F | adult | non-oestrous | - | - | - | - | 0 | 0 |
| 08/06/2013 | C | O | Sett | 21 | F | adult | non-oestrous | 45 | F | adult | oestrous | 0 | 0 |
| 08/06/2013 | C | O | Sett | 21 | F | adult | non-oestrous | - | - | - | - | 0 | 0 |
| 08/06/2013 | C | O | Sett | 21 | F | adult | non-oestrous | 51 | F | adult | oestrous | 0 | 0 |
| 08/06/2013 | C | O | Sett | 21 | F | adult | non-oestrous | 47 | F | adult | non-oestrous | 0 | 0 |
| 08/06/2013 | C | O | Sett | 21 | F | adult | non-oestrous | - | - | - | - | 0.602059991 | 0 |
| 08/06/2013 | C | O | Sett | 21 | F | adult | non-oestrous | 45 | F | adult | oestrous | 0 | 0 |
| 08/06/2013 | C | O | Sett | 21 | F | adult | non-oestrous | - | - | - | - | 0.301029996 | 0 |
| 08/06/2013 | C | O | Sett | 21 | F | adult | non-oestrous | - | - | - | - | 0 | 0 |
| 08/06/2013 | C | O | Sett | 21 | F | adult | non-oestrous | - | - | - | - | 0 | 0 |
| 08/06/2013 | C | O | Sett | 21 | F | adult | non-oestrous | - | - | - | - | 0 | 0 |
| 08/06/2013 | C | O | Sett | 21 | F | adult | non-oestrous | - | - | - | - | 0 | 0 |
| 08/06/2013 | C | O | Sett | 21 | F | adult | non-oestrous | 53 | M | adult | - | 0 | 0 |
| 08/06/2013 | F | N | Unshared | 13 | F | adult | oestrous | 52 | F | adult | non-oestrous | 0 | 0 |
| 08/06/2013 | F | N | Unshared | 13 | F | adult | oestrous | 65 | M | adult | fully descended | 0.602059991 | 2 |
| 08/06/2013 | F | N | Unshared | 13 | F | adult | oestrous | 65 | M | adult | fully descended | 0 | 1 |
| 08/06/2013 | F | N | Unshared | 13 | F | adult | oestrous | 43 | F | adult | non-oestrous | 0.301029996 | 1 |
| 09/06/2013 | C | N | Unshared | 43 | F | adult | non-oestrous | - | - | - | - | 0.77815125 | 0 |
| 09/06/2013 | C | N | Unshared | 43 | F | adult | non-oestrous | 63 | F | adult | non-oestrous | 0 | 0 |
| 09/06/2013 | C | N | Unshared | 43 | F | adult | non-oestrous | 53 | M | adult | - | 0 | 0 |
| 09/06/2013 | C | N | Unshared | 43 | F | adult | non-oestrous | - | - | - | - | 0.301029996 | 1 |
| 09/06/2013 | C | N | Unshared | 43 | F | adult | non-oestrous | 52 | F | adult | non-oestrous | 0 | 0 |
| 09/06/2013 | F | O | Sett | 17 | F | adult | oestrous | 65 | M | adult | fully descended | 0 | 0 |
| 09/06/2013 | F | O | Sett | 17 | F | adult | oestrous | 19 | F | adult | oestrous | 0 | 0 |
| 09/06/2013 | F | O | Sett | 17 | F | adult | oestrous | 19 | F | adult | oestrous | 0 | 0 |
| 09/06/2013 | F | O | Sett | 17 | F | adult | oestrous | - | - | - | - | 0 | 0 |
| 09/06/2013 | F | O | Sett | 17 | F | adult | oestrous | 47 | F | adult | non-oestrous | 0.698970004 | 0 |
| 09/06/2013 | F | O | Sett | 17 | F | adult | oestrous | 17 | F | adult | oestrous | 0 | 0 |
| 09/06/2013 | F | O | Sett | 17 | F | adult | oestrous | 17 | F | adult | oestrous | 0 | 0 |
| 09/06/2013 | F | O | Sett | 17 | F | adult | oestrous | 65 | M | adult | fully descended | 0 | 0 |
| 09/06/2013 | F | O | Sett | 17 | F | adult | oestrous | 44 | F | adult | oestrous | 0 | 0 |
| 09/06/2013 | F | S | Border | 24 | F | adult | oestrous | - | - | - | - | 0 | 0 |
| 09/06/2013 | F | S | Border | 24 | F | adult | oestrous | - | - | - | - | 0.84509804 | 1 |
| 09/06/2013 | F | S | Border | 24 | F | adult | oestrous | - | - | - | - | 0 | 0 |
| 09/06/2013 | F | S | Border | 24 | F | adult | oestrous | - | - | - | - | 0 | 0 |
| 09/06/2013 | F | S | Border | 24 | F | adult | oestrous | - | - | - | - | 0.84509804 | 2 |
| 09/06/2013 | F | S | Border | 24 | F | adult | oestrous | 44 | F | adult | oestrous | 0.77815125 | 1 |
| 09/06/2013 | F | N | Shared | 47 | F | adult | non-oestrous | 56 | F | adult | oestrous | 0 | 0 |
| 09/06/2013 | F | N | Shared | 47 | F | adult | non-oestrous | 56 | F | adult | oestrous | 0.301029996 | 1 |
| 09/06/2013 | F | N | Shared | 47 | F | adult | non-oestrous | 19 | F | adult | oestrous | 0 | 0 |
| 10/06/2013 | C | S | Border | 19 | F | adult | oestrous | 62 | F | adult | non-oestrous | 0.602059991 | 1 |
| 10/06/2013 | C | S | Border | 19 | F | adult | oestrous | 62 | F | adult | non-oestrous | 0.301029996 | 1 |
| 10/06/2013 | C | S | Border | 19 | F | adult | oestrous | 45 | F | adult | oestrous | 0.698970004 | 1 |
| 10/06/2013 | C | S | Border | 19 | F | adult | oestrous | - | - | - | - | 0.954242509 | 1 |
| 10/06/2013 | C | S | Border | 19 | F | adult | oestrous | 5 | M | adult | fully descended | 0.602059991 | 2 |
| 10/06/2013 | C | S | Border | 19 | F | adult | oestrous | 62 | F | adult | non-oestrous | 0 | 0 |
| 10/06/2013 | F | S | Sett | 7 | F | adult | oestrous | 44 | F | adult | oestrous | 0.77815125 | 1 |
| 10/06/2013 | F | S | Sett | 7 | F | adult | oestrous | 44 | F | adult | oestrous | 0 | 0 |
| 10/06/2013 | F | S | Sett | 7 | F | adult | oestrous | 43 | F | adult | non-oestrous | 0.903089987 | 1 |
| 10/06/2013 | F | S | Sett | 7 | F | adult | oestrous | 38 | M | juvenile | fully descended | 0.84509804 | 0 |
| 10/06/2013 | F | S | Sett | 7 | F | adult | oestrous | 17 | F | adult | oestrous | 0 | 0 |
| 10/06/2013 | F | S | Sett | 7 | F | adult | oestrous | 65 | M | adult | fully descended | 0.84509804 | 2 |
| 10/06/2013 | F | N | Unshared | 39 | F | juvenile | non-oestrous | 65 | M | adult | fully descended | 0.77815125 | 2 |
| 10/06/2013 | F | N | Unshared | 39 | F | juvenile | non-oestrous | - | - | - | - | 0.301029996 | 1 |
| 10/06/2013 | F | N | Unshared | 39 | F | juvenile | non-oestrous | 70 | F | juvenile | oestrous | 0 | 0 |
| 10/06/2013 | F | S | Border | 3 | F | adult | non-oestrous | 56 | F | adult | oestrous | 0 | 0 |
| 10/06/2013 | F | S | Border | 3 | F | adult | non-oestrous | 19 | F | adult | oestrous | 0.301029996 | 1 |
| 10/06/2013 | F | S | Border | 3 | F | adult | non-oestrous | 65 | M | adult | fully descended | 0.602059991 | 0 |
| 10/06/2013 | H | N | Sett | 33 | F | juvenile | non-oestrous | - | - | - | - | 0.477121255 | 0 |
| 10/06/2013 | H | N | Sett | 33 | F | juvenile | non-oestrous | - | - | - | - | 0 | 0 |
| 11/06/2013 | C | S | Border | 32 | F | juvenile | non-oestrous | 62 | F | adult | non-oestrous | 0.698970004 | 0 |
| 11/06/2013 | C | S | Border | 32 | F | juvenile | non-oestrous | 52 | F | adult | non-oestrous | 0.903089987 | 3 |
| 11/06/2013 | C | S | Border | 32 | F | juvenile | non-oestrous | 29 | F | adult | non-oestrous | 0.301029996 | 1 |
| 11/06/2013 | C | S | Border | 32 | F | juvenile | non-oestrous | 5 | M | adult | fully descended | 0.602059991 | 0 |
| 11/06/2013 | C | S | Border | 32 | F | juvenile | non-oestrous | 29 | F | adult | non-oestrous | 0.477121255 | 1 |
| 11/06/2013 | C | S | Border | 32 | F | juvenile | non-oestrous | - | - | - | - | 0 | 0 |
| 11/06/2013 | C | S | Border | 32 | F | juvenile | non-oestrous | - | - | - | - | 0.698970004 | 0 |
| 11/06/2013 | C | S | Border | 32 | F | juvenile | non-oestrous | - | - | - | - | 0.477121255 | 1 |
| 11/06/2013 | C | S | Border | 32 | F | juvenile | non-oestrous | 62 | F | adult | non-oestrous | 0.77815125 | 1 |
| 11/06/2013 | C | S | Border | 32 | F | juvenile | non-oestrous | 62 | F | adult | non-oestrous | 0.301029996 | 1 |
| 11/06/2013 | C | S | Border | 32 | F | juvenile | non-oestrous | - | - | - | - | 0 | 0 |
| 11/06/2013 | C | S | Border | 32 | F | juvenile | non-oestrous | - | - | - | - | 0.602059991 | 1 |
| 11/06/2013 | C | S | Border | 32 | F | juvenile | non-oestrous | - | - | - | - | 0.77815125 | 2 |
| 11/06/2013 | F | N | Sett | 29 | F | adult | non-oestrous | 38 | M | juvenile | fully descended | 0.84509804 | 0 |
| 11/06/2013 | F | N | Sett | 29 | F | adult | non-oestrous | 43 | F | adult | non-oestrous | 0.602059991 | 0 |
| 11/06/2013 | F | N | Sett | 29 | F | adult | non-oestrous | 44 | F | adult | oestrous | 0.77815125 | 1 |
| 11/06/2013 | F | O | Border | 43 | F | adult | non-oestrous | 63 | F | adult | non-oestrous | 0 | 0 |
| 11/06/2013 | F | O | Border | 43 | F | adult | non-oestrous | - | - | - | - | 1.113943352 | 0 |
| 11/06/2013 | F | O | Border | 43 | F | adult | non-oestrous | - | - | - | - | 0 | 0 |
| 11/06/2013 | F | O | Border | 43 | F | adult | non-oestrous | 66 | F | adult | non-oestrous | 0.477121255 | 1 |
| 11/06/2013 | F | O | Border | 43 | F | adult | non-oestrous | 65 | M | adult | fully descended | 0.301029996 | 1 |
| 11/06/2013 | F | O | Border | 44 | F | adult | oestrous | 73 | F | juvenile | non-oestrous | 0 | 0 |
| 11/06/2013 | F | O | Border | 44 | F | adult | oestrous | 43 | F | adult | non-oestrous | 0.301029996 | 0 |
| 11/06/2013 | F | O | Border | 44 | F | adult | oestrous | 66 | F | adult | non-oestrous | 0.477121255 | 1 |
| 11/06/2013 | F | O | Border | 44 | F | adult | oestrous | 56 | F | adult | oestrous | 0 | 0 |
| 11/06/2013 | F | O | Border | 44 | F | adult | oestrous | 73 | F | juvenile | non-oestrous | 0 | 0 |
| 11/06/2013 | F | O | Sett | 44 | F | adult | oestrous | 65 | M | adult | fully descended | 0.77815125 | 1 |
| 11/06/2013 | H | S | Sett | 20 | F | adult | - | - | - | - | - | 0.77815125 | 2 |
| 11/06/2013 | H | S | Sett | 20 | F | adult | - | - | - | - | - | 1.146128036 | 0 |
| 12/06/2013 | E | N | Sett | 35 | M | juvenile | descended | - | - | - | - | 0 | 0 |
| 12/06/2013 | E | N | Sett | 35 | M | juvenile | descended | - | - | - | - | 0.602059991 | 0 |
| 12/06/2013 | E | N | Sett | 35 | M | juvenile | descended | - | - | - | - | 0 | 0 |
| 12/06/2013 | E | N | Sett | 35 | M | juvenile | descended | 19 | F | adult | oestrous | 0.903089987 | 1 |
| 12/06/2013 | D | S | Sett | 46 | F | adult | non-oestrous | 52 | F | adult | non-oestrous | 0.602059991 | 2 |
| 12/06/2013 | D | S | Sett | 46 | F | adult | non-oestrous | 29 | F | adult | non-oestrous | 0.602059991 | 1 |
| 12/06/2013 | D | S | Sett | 46 | F | adult | non-oestrous | - | - | - | - | 0.698970004 | 1 |
| 12/06/2013 | D | S | Sett | 46 | F | adult | non-oestrous | 63 | F | adult | non-oestrous | 0.301029996 | 1 |
| 12/06/2013 | D | S | Sett | 46 | F | adult | non-oestrous | 63 | F | adult | non-oestrous | 0 | 0 |
| 12/06/2013 | D | S | Sett | 46 | F | adult | non-oestrous | 66 | F | adult | non-oestrous | 0.301029996 | 2 |
| 12/06/2013 | A | N | Sett | 16 | M | adult | descended | - | - | - | - | 1.041392685 | 1 |
| 12/06/2013 | A | N | Sett | 16 | M | adult | descended | - | - | - | - | 0 | 0 |
| 12/06/2013 | A | N | Sett | 16 | M | adult | descended | - | - | - | - | 0 | 0 |
| 12/06/2013 | A | N | Sett | 16 | M | adult | descended | - | - | - | - | 0.698970004 | 0 |
| 12/06/2013 | A | N | Sett | 16 | M | adult | descended | 59 | F | adult | oestrous | 0.301029996 | 0 |
| 12/06/2013 | A | N | Sett | 16 | M | adult | descended | - | - | - | - | 0.602059991 | 0 |
| 12/06/2013 | B | O | Sett | 9 | M | adult | fully descended | 51 | F | adult | oestrous | 0 | 0 |
| 12/06/2013 | B | O | Sett | 9 | M | adult | fully descended | 69 | F | juvenile | non-oestrous | 0 | 0 |
| 12/06/2013 | B | O | Sett | 9 | M | adult | fully descended | 55 | M | adult | non-oestrous | 0 | 0 |
| 12/06/2013 | B | O | Sett | 9 | M | adult | fully descended | 9 | M | adult | fully descended | 0.301029996 | 0 |
| 12/06/2013 | B | O | Sett | 9 | M | adult | fully descended | 51 | F | adult | oestrous | 0 | 0 |
| 12/06/2013 | B | O | Sett | 9 | M | adult | fully descended | - | - | - | - | 0 | 0 |
| 12/06/2013 | B | O | Sett | 9 | M | adult | fully descended | - | - | - | - | 0 | 0 |
| 12/06/2013 | B | O | Sett | 9 | M | adult | fully descended | 64 | F | adult | oestrous | 0 | 0 |
| 12/06/2013 | B | O | Sett | 9 | M | adult | fully descended | 9 | M | adult | fully descended | 0 | 0 |
| 12/06/2013 | B | O | Sett | 9 | M | adult | fully descended | 69 | F | juvenile | non-oestrous | 0.301029996 | 0 |
| 12/06/2013 | B | O | Sett | 9 | M | adult | fully descended | 69 | F | juvenile | non-oestrous | 0 | 0 |
| 12/06/2013 | B | O | Sett | 9 | M | adult | fully descended | 64 | F | adult | oestrous | 0 | 0 |
| 12/06/2013 | B | O | Sett | 9 | M | adult | fully descended | 26 | M | adult | descended | 0 | 0 |
| 13/06/2013 | D | N | Sett | 21 | F | adult | non-oestrous | 63 | F | adult | non-oestrous | 1.146128036 | 1 |
| 13/06/2013 | D | N | Sett | 21 | F | adult | non-oestrous | 66 | F | adult | non-oestrous | 0.698970004 | 1 |
| 13/06/2013 | D | N | Sett | 21 | F | adult | non-oestrous | 63 | F | adult | non-oestrous | 0.301029996 | 1 |
| 13/06/2013 | D | N | Sett | 21 | F | adult | non-oestrous | 29 | F | adult | non-oestrous | 0.602059991 | 1 |
| 13/06/2013 | D | N | Sett | 21 | F | adult | non-oestrous | 66 | F | adult | non-oestrous | 0 | 0 |
| 13/06/2013 | D | N | Sett | 21 | F | adult | non-oestrous | - | - | - | - | 1.041392685 | 1 |
| 13/06/2013 | A | O | Sett | 15 | M | adult | fully descended | - | - | - | - | - | 0 |
| 13/06/2013 | A | O | Sett | 15 | M | adult | fully descended | - | - | - | - | 0 | 0 |
| 13/06/2013 | A | O | Sett | 15 | M | adult | fully descended | - | - | - | - | 0 | 0 |
| 13/06/2013 | A | O | Sett | 15 | M | adult | fully descended | - | - | - | - | 0 | 0 |
| 13/06/2013 | A | S | Border | 36 | M | juvenile | fully descended | - | - | - | - | 1.176091259 | 1 |
| 13/06/2013 | A | S | Border | 36 | M | juvenile | fully descended | - | - | - | - | 0.301029996 | 1 |
| 13/06/2013 | B | O | Border | 9 | M | adult | fully descended | 9 | M | adult | fully descended | 0 | 0 |
| 13/06/2013 | B | O | Border | 9 | M | adult | fully descended | 26 | M | adult | descended | 0.698970004 | 0 |
| 13/06/2013 | B | O | Border | 9 | M | adult | fully descended | 69 | F | juvenile | non-oestrous | 0.602059991 | 0 |
| 13/06/2013 | B | S | Border | 30 | M | adult | fully descended | 48 | M | adult | descended | 0 | 0 |
| 13/06/2013 | B | S | Border | 30 | M | adult | fully descended | 9 | M | adult | fully descended | 0.301029996 | 3 |
| 14/06/2013 | A | N | Shared | 16 | M | adult | descended | 61 | F | adult | oestrous | 0.84509804 | 0 |
| 14/06/2013 | A | N | Shared | 16 | M | adult | descended | 61 | F | adult | oestrous | 0 | 0 |
| 14/06/2013 | B | N | Sett | 42 | M | adult | descended | 69 | F | juvenile | non-oestrous | 0.698970004 | 1 |
| 14/06/2013 | B | N | Unshared | 15 | M | adult | fully descended | 69 | F | juvenile | non-oestrous | 0.77815125 | 2 |
| 14/06/2013 | B | N | Unshared | 15 | M | adult | fully descended | 48 | M | adult | descended | 0.602059991 | 1 |
| 14/06/2013 | B | N | Unshared | 15 | M | adult | fully descended | 9 | M | adult | fully descended | 1.041392685 | 2 |
| 14/06/2013 | B | N | Unshared | 15 | M | adult | fully descended | 51 | F | adult | oestrous | 0 | 0 |
| 12/06/2013 | B | S | Border | 12 | M | adult | fully descended | 9 | M | adult | fully descended | 0.903089987 | 3 |
| 12/06/2013 | B | S | Border | 12 | M | adult | fully descended | 69 | F | juvenile | non-oestrous | 1 | 1 |
| 12/06/2013 | B | S | Border | 12 | M | adult | fully descended | - | - | - | - | 0 | 0 |
| 12/06/2013 | B | S | Border | 12 | M | adult | fully descended | - | - | - | - | 0.477121255 | 1 |
| 06/06/2012 | L | O | Sett | 28 | M | adult | fully descended | - | - | - | - | 0 | 0 |
| 06/06/2012 | L | O | Sett | 28 | M | adult | fully descended | - | - | - | - | 0 | 0 |
| 01/06/2012 | K | O | Border | 27 | M | adult | fully descended | - | - | - | - | 0 | 0 |
| 01/06/2012 | K | O | Border | 27 | M | adult | fully descended | - | - | - | - | 0 | 0 |
| 01/06/2012 | K | O | Border | 27 | M | adult | fully descended | - | - | - | - | 0 | 0 |
| 01/06/2012 | K | O | Border | 27 | M | adult | fully descended | - | - | - | - | - | 1 |
| 01/06/2012 | K | O | Border | 27 | M | adult | fully descended | - | - | - | - | 0 | 0 |
| 01/06/2012 | K | N | Border | 31 | M | juvenile | fully descended | - | - | - | - | 0.77815125 | 0 |
| 01/06/2012 | K | N | Border | 31 | M | juvenile | fully descended | - | - | - | - | 0.477121255 | 0 |
| 04/06/2012 | J | O | Border | 8 | M | adult | fully descended | - | - | - | - | 0 | 0 |
| 04/06/2012 | J | O | Border | 8 | M | adult | fully descended | 77 | M | - | - | 0 | 0 |
| 04/06/2012 | J | O | Border | 8 | M | adult | fully descended | - | - | - | - | 0 | 0 |
| 04/06/2012 | J | O | Border | 8 | M | adult | fully descended | - | - | - | - | - | 0 |
| 04/06/2012 | J | O | Border | 8 | M | adult | fully descended | - | - | - | - | 0 | 0 |
| 04/06/2012 | J | O | Border | 8 | M | adult | fully descended | - | - | - | - | 0 | 0 |
| 04/06/2012 | J | O | Border | 8 | M | adult | fully descended | - | - | - | - | 0 | 0 |
| 04/06/2012 | J | O | Border | 8 | M | adult | fully descended | - | - | - | - | 0 | 0 |
| 04/06/2012 | J | O | Border | 8 | M | adult | fully descended | - | - | - | - | 0 | 0 |
